# Enhancing Conservation Efforts in the Qinling Mountains Through Phenotypic Trait Diversity Optimization

**DOI:** 10.3390/plants14142130

**Published:** 2025-07-10

**Authors:** Sibo Chen, Xin Fu, Kexin Chen, Jinguo Hua, Qian Rao, Xuewei Feng, Wenli Ji

**Affiliations:** 1 College of Landscape Architecture and Arts, Northwest A&F University, Xianyang 712100, China; chensibo@nwafu.edu.cn (S.C.);; 2 College of the Environment and Ecology, Xiamen University, Xiamen 361102, China; 3 Haikou Jinniuling Management Office, Haikou 570206, China

**Keywords:** phenotypic traits, distribution pattern, diversity hotspot, conservation gap, conservation area

## Abstract

The establishment of conservation areas is considered one of the most effective approaches to address biodiversity loss with limited resources. Identifying hotspots of plant diversity and conservation gaps has played a crucial role in optimizing conservation areas. Utilizing diverse types of research data can effectively enhance the recognition of hotspots and conservation gaps. Phenotypic trait diversity is a functional biogeography that analyzes the geographic distribution patterns, formation, and reasons for the development of specific or multiple phenotypic traits of organisms. Flower color and fruit color phenotypic traits are primary characteristics through which plants interact with other organisms, affecting their own survival and reproduction, and that of their offspring. This study utilized data from 1923 Phenotypic Trait Diversity Species (PTDS) with flower and fruit color characteristics to optimize conservation areas in the Shaanxi Qinling Mountains. Additionally, data from 1838 endemic species (ES), 190 threatened species (TS), and 119 protected species (PS) were used for validation. The data were primarily sourced from the Catalogue of Vascular Plants in Shaanxi, supplemented by the Chinese Virtual Herbarium and the Shaanxi Digital Herbarium. The results reveal that by comparing the existing conservation area boundaries with those determined by four types of data, conservation gaps are found in 14 counties in the Qinling Mountains of Shaanxi. The existing conservation area only accounts for 13.3% of the area determined by the four types of data. There are gaps in biodiversity conservation in the Qinling Mountains of Shaanxi, and the macroscopic use of plant phenotypic trait data contributes to optimizing these conservation gaps.

## 1. Introduction

Due to the threats posed by climate change and human activities, the ongoing loss of biodiversity has emerged as one of the most serious environmental issues in recent decades [1,2,3,4]. Approximately one-fifth of the world’s plant species face the risk of extinction in the wild in the near future [5]. Biodiversity forms the cornerstone of a healthy environment, and is crucial not only for human development but also for the well-being of all life on Earth [6,7]. Sustainable ecosystems heavily rely on biodiversity, prompting a macroscopic exploration of its geographic distribution and formation mechanisms, a key focus in biogeography and ecology [8,9]. Establishing conservation areas is recognized as one of the most effective strategies to mitigate biodiversity loss, particularly under resource constraints [10,11]. Currently, China boasts over 2700 conservation areas [12]. However, many face challenges, such as an uneven distribution, decentralized management, and inadequate spatial allocation, falling short in meeting the demands of biodiversity protection and ecosystem services [13]. Furthermore, some traditional conservation areas prioritize wildlife over wild plants [14]. Thus, evaluating the effectiveness of existing conservation areas, identifying conservation gaps, optimizing plant conservation efforts, and developing tailored conservation plans are imperative steps forward.

The identification of plant diversity hotspots and conservation gaps is pivotal in planning and optimizing conservation areas [15,16,17]. Hotspots denote geographical regions where endemic or particularly significant species face threats, often characterized by a high concentration of species [18]. Researchers have undertaken various approaches to more effectively identify hotspots and furnish reliable data for prioritizing conservation areas [19,20]. Recent attention has also focused on more threatened or significant plants like medicinal plants, orchids, and magnolias [21,22]. Innovative research methods have markedly advanced hotspot and priority conservation area identification.

Using different species’ distribution data can effectively enhance the identification of hotspot areas and conservation gaps, as different methods focus on various aspects of biodiversity conservation [23]. Simultaneously utilizing multiple plant datasets can effectively identify priority conservation areas [11]. The formation of endemic species (ES) is associated with long-term evolutionary adaptation and geographic differentiation. Endemic plants, due to their high sensitivity to environmental changes, are well-suited to survive in environments with high survival pressure and low habitat competition. Threatened species (TS) and protected species (PS), however, are less capable of adapting to climate and environmental changes, making them more vulnerable to external environmental changes and potentially at risk of becoming endangered or extinct [24,25]. The distribution patterns and spatial patterns of species richness for endemic species, threatened species, and protected species are largely inconsistent [8,26]. Therefore, to identify priority conservation areas, multiple methods and the combined use of different plant category data are necessary. However, using multiple plant category data to identify conservation hotspots still falls short of the goals set by the Global Strategy for Plant Conservation (GSPC, http://www.plants2020.net/, accessed on 20 November 2024), which aims to protect at least 75% of key plant diversity areas [27]. Traditional studies using plant category data have not significantly improved the identification of hotspot areas in conservation area optimization. Consequently, there is an urgent need for new methods to provide a more comprehensive and effective selection of conservation areas.

**Figure 1:**
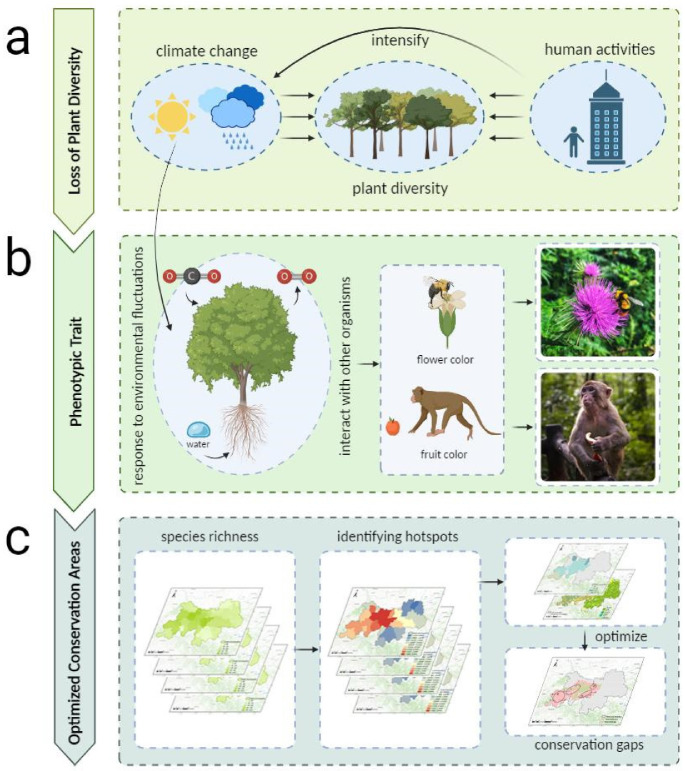
The research roadmap for optimizing conservation areas in the Qinling Mountains of Shaanxi through flower and fruit color phenotypic traits. (**a**) Climate change and human activities are the main factors causing the loss of plant diversity. (**b**) Plants respond to environmental fluctuations through phenotypic traits and interact with other organisms. The flower and fruit colors studied in this paper are major phenotypic traits influencing the survival strategies of plants themselves or their offspring. (**c**) The research pathway to achieve optimized conservation areas in this paper. This figure was created using BioRender (Version 1.0).

Phenotypic trait diversity, a functional aspect of biogeography, examines the geographic distribution patterns, formation, and developmental rationales behind various phenotypic traits across organisms [28]. Its exploration aims to elucidate the correlation between the geographic distribution patterns of biological traits and species diversity within ecosystems [29]. Phenotypic traits encompass morphology, physiology, phenology, and other organismal characteristics [30]. Plant phenotypic traits directly or indirectly shape survival strategies, growth, reproduction, and the survival of both themselves and their progeny [31]. As communities engage in complex interactions such as competition and mutualism, novel community compositions emerge. Concurrently, plants interact with regional organisms through phenotypic traits, responding to environmental fluctuations and thus impacting regional biodiversity and fostering the interplay between phenotypic traits, survival, and biodiversity [28,32]. Phenotypic trait diversity holds paramount importance in biodiversity conservation. Yet, acquiring data for one or more phenotypic traits across all species within vast geographic regions has posed challenges, impeding the application of phenotypic trait diversity in biodiversity conservation and conservation area optimization [11,21]. Thanks to photographic data compiled by scientific research teams, information on plant flower color and fruit color phenotypic traits has become widely accessible. Flower and fruit color phenotypic traits serve as primary conduits through which plants interact with other organisms, influencing their own survival and reproduction, and that of their offspring [33,34]. For instance, the selection of flower color phenotypic traits among different populations significantly impacts the richness and visitation frequency of pollinators [35]. Additionally, the size and color of plant fruits contribute to attracting or repelling herbivores or omnivores, potentially enhancing survival and reproductive prospects [36] (Figure 1).

Given the central role of flower and fruit color traits in driving key ecological interactions (e.g., pollination and seed dispersal) and shaping regional biodiversity patterns [36,37], this study innovatively proposes and applies the “Plant Flower and Fruit Color Trait Diversity” dataset as a key basis for identifying biodiversity conservation hotspots. The originality of this approach lies in (1) being the first to systematically incorporate visual trait diversity (flower and fruit color) as a core dimension for large-scale conservation priority planning. Traditional conservation planning often relies on species richness or phylogenetic diversity, whereas integrating the diversity patterns of key plant reproductive traits that directly influence ecosystem functions (e.g., pollination network stability and seed dispersal efficiency) into the conservation area identification framework represents the core innovation of this study [38]. (2) It fully utilizes emerging, easily standardized macro-trait data sources. This study overcomes the previous bottleneck in trait diversity research, which was limited by the difficulty of obtaining large-scale, multi-species detailed morphological or physiological trait data, by transforming widely shared field observation photographic records into quantifiable and comparable trait diversity information. Consequently, hotspot areas identified based on Phenotypic Trait Diversity Species (PTDS) data can more precisely protect the key interaction networks that sustain ecosystem functionality and resilience. Additionally, this data type offers unique advantages in terms of operability and universality [39].

Mountain ecosystems typically harbor rich biodiversity and are more sensitive to climate change [40]. Many species or communities in mountainous regions are more vulnerable to global climate change, leading to increased risks of endangerment or extinction [41]. As a natural barrier between temperate and subtropical climates, the Qinling Mountains serve as the convergence point of two major plant subregions in East Asia: the Sino-Japanese Forest Subregion and the Sino-Himalayan Forest Subregion. Known as the “core” of China’s ecosystems, the Qinling Mountains boast abundant species diversity and are prioritized areas for biodiversity conservation in China [3,42]. We utilized distribution data for plants across 35 counties (districts) in the Shaanxi Qinling Mountains, employing various indicators including Phenotypic Trait Diversity Species (PTDS) with flower and fruit color phenotypic traits, endemic species, threatened species, and protected species, to achieve the following objectives: (1) clarify the composition of PTDS in the Shaanxi Qinling Mountains; (2) study the geographic distribution patterns of vascular plants in the Shaanxi Qinling Mountains based on PTDS, endemic species, threatened species, and protected species richness indicators; (3) verify the potential of PTDS to fill existing conservation gaps by assessing the spatial consistency of PTDS hotspots with those of endemic species, threatened species, and protected species hotspots; and (4) provide recommendations for optimizing existing conservation areas in the Shaanxi Qinling Mountains.

## 2. Materials and Methods

### 2.1. Study Area

**Figure 2:**
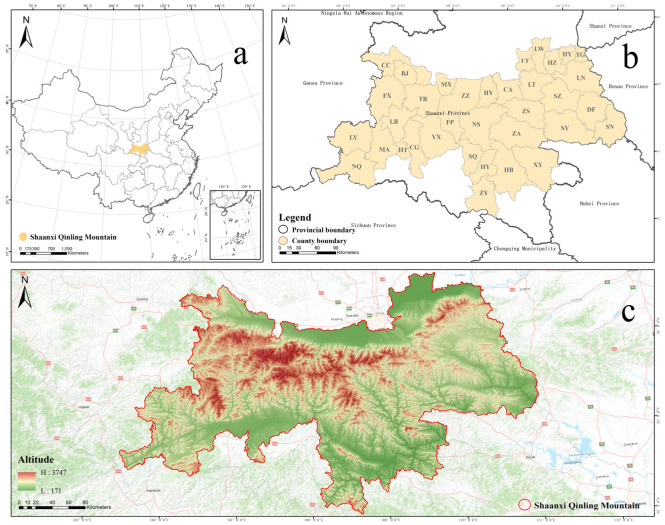
(**a**) Illustration of the position of the Qinling Mountains in Shaanxi, China. (**b**) Depiction of the positions of the 35 counties (districts) in the Qinling Mountains of Shaanxi along with their abbreviations. Abbreviations of county names: LT, Lintong; CA, Changan; HU, Huyi; LA, Lantian; ZZ, Zhouzhi; BJ, Baoji; CC, Chencang; MX, Mei; FX, Feng; TB, Taibai; LW, Linwei; HZ, Huazhou; TG, Tongguan; HY, Huayin; HT, Hantai; CG, Chenggu; YX, Yang; MA, Mian; NQ, Ningqiang; LY, Lueyang; LB, Liuba; FP, Foping; HB, Hanbin; HA, Hanyin; SQ, Shiquan; NS, Ningshan; ZY, Ziyang; XY, Xuyang; SZ, Shangzhou; LN, Luonan; DF, Danfeng; SN, Shangnan; SY, Shanyang; ZA, Zhenan; ZS, Zhashui. (**c**) Elevation map of the Qinling Mountains in Shaanxi.

The Qinling Mountains are situated in central China and are considered the natural boundary between southern and northern China. The northern part of the Qinling Mountains features a warm temperate semi-humid climate, while the southern part has a humid subtropical climate [3,43]. Its obstruction of air circulation results in dry summers in the north and warm winters in the south, earning it the nickname “Central Air Conditioning” of China [44]. Moreover, serving as the watershed between the Yangtze River and Yellow River, it is also referred to as China’s “Central Water Tower”. Due to its unique transitional nature between the north and south of China, the Qinling Mountains harbor complex and sensitive habitats [45]. The average annual temperature is 13 °C, with an average annual rainfall of 820 mm [46]. There are significant north–south differences. The northern slopes are steep with high mountains and numerous valleys, dominated by deciduous broad-leaved forests. In contrast, the southern slopes are gentle with hills covered in a mix of tropical deciduous and evergreen broad-leaved forests [47]. The elevation gradually increases from west to east, with the highest peak located in Taibai County at 3747.2 m. The broader Qinling Mountains span across Gansu, Shaanxi, and Henan Provinces, with its main portion located in Shaanxi. For the sake of data consistency, this study selected the Shaanxi section of the Qinling Mountains (32°6′–34°48′ N, 105°27′–111°3′ E). The study area encompasses 36 counties (districts) across six city-level administrative regions: Xi’an, Weinan, Baoji, Hanzhong, Shangluo, and Ankang. Both Weibin District and Jintai District are municipally administered districts with relatively few plant species. Therefore, based on the principle of merging adjacent areas with smaller sizes, these two districts were combined into a single administrative region, resulting in a total of 35 counties (districts). The eastern boundary of the study area extends to Shangnan County; the western boundary reaches Ningqiang County and Lueyang County; the northern boundary includes Chencang District and Linwei District; and the southern boundary encompasses Ziyang County (Figure 2).

### 2.2. Collection of the Species Catalog and Distribution Data

#### 2.2.1. Collection and Sources of Phenotypic Trait Diversity Species (PTDS) Data

The Phenotypic Trait Diversity Species data used in the study consist of data on plant species with flower and fruit color traits from the Qinling Mountains, Shaanxi, abbreviated as PTDS for the reader’s convenience. We established a phenotypic trait database for the 1923 species of PTDS used in our study in the Shaanxi Qinling Mountains (the species list is provided in the Supplementary Materials). PTDS data primarily originated from the Catalogue of Vascular Plants in Shaanxi. Supplemental data were obtained from the Chinese Virtual Herbarium (http://www.cvh.ac.cn/, accessed on 5 October 2024) and the Shaanxi Digital Herbarium (http://site.nsii.org.cn, accessed on 13 November 2024). County-level divisions were chosen as the geographical units for analyzing the spatial distribution patterns of plants. Plant distributions are not confined to singular locations within geographical ranges, and most plants do not occur solely within specific grid resolutions. Therefore, county-level distribution data for plants are more reliable for a spatial distribution analysis [8,48]. The lists of plants from different sources were standardized according to the Catalogue of Life China (Species 2000 China, http://col.especies.cn, accessed on 20 November 2024). The Catalogue of Life China also includes taxonomic data for some animal species in the study area (used to elucidate animal–plant interactions). Plant phenotypic traits were determined using resources such as the Chinese Plant Image Library (PPBC, http://ppbc.iplant.cn/, accessed on 26 November 2024), Flora of China, Flora of the Qinling Mountains, 1600 Species of Garden Trees, and Woody Flowers of Northern China. Each species was recorded with the following information: (i) life form, including trees, shrubs, vines, and herbs; and (ii) phenotypic traits, selecting flower color and fruit color, which influence plant survival strategies [33,35,36]. We followed the APG IV (Angiosperm Phylogeny Group IV) classification system [48].

#### 2.2.2. Collection and Sources of Endemic Species (ES), Threatened Species (TS), and Protected Species (PS) Data

Endemic species, threatened species, and key protected wild plants have been considered important criteria for identifying hotspot areas in previous studies [10,49]. Therefore, we utilized data from these three categories to identify hotspot areas and validate the effectiveness of PTDS in filling conservation gaps. Endemic species and threatened species data were sourced from the China Rare and Endangered Plants System (http://www.iplant.cn/rep/protlist, accessed on 14 November 2024). We obtained a total of 1838 endemic species and 190 threatened species. Threatened species were classified according to the IUCN (International Union for Conservation of Nature) threat levels, including Critically Endangered (CR) with 18 species, Endangered (EN) with 58 species, and Vulnerable (VU) with 114 species. Data on key protected wild plants were sourced from the List of Key Protected Wild Plants in China (published on 7 September 2021), totaling 119 species, including 12 species classified as Level I national protected wild plants and 107 species as Level II national protected wild plants. The distribution of the aforementioned species in the counties was determined using the Shaanxi Digital Herbarium (http://site.nsii.org.cn, accessed on 26 November 2024), with the species list provided in the Supplementary Materials.

### 2.3. Distribution Patterns and Analysis of Hotspot Areas

Due to its simplicity and ease of implementation, richness algorithms have become the most commonly used method for identifying plant diversity hotspots [10,20]. Therefore, we employed richness algorithms to visualize the geographic distribution data of PTDS, endemic species, threatened species, and key protected wild plants, and plotted maps of county-level richness for PTDS (DR), endemic species richness (ER), threatened species richness (TR), and key protected wild plants species richness (PR).

Using the Getis-Ord Gi* statistical method in ArcGIS software, we calculated whether the occurrence patterns of specific counties and their neighboring counties showed significant clustering or dispersion trends [50]. The Getis-Ord Gi* statistical method evaluates the spatial distribution characteristics of species richness by computing z-score statistics. This statistic assesses the spatial distribution of species richness by comparing the weighted sum of species richness in specific counties with the total sum of the sample [51]. A high z-score and low p-value indicate a high-value spatial cluster, while a low z-score and low *p*-value indicate a low-value spatial cluster. The higher (or lower) the z-score, the greater (or lower) the degree of clustering. A z-score close to 0 indicates no significant spatial clustering [42]. The calculation formula is as follows:

(1) $$G_{i}^{*}=\frac{\sum_{j=1}^{n}w_{i,j}x_{j}-\bar{X}\sum_{j=1}^{n}w_{i,j}}{S\sqrt{\frac{\left[n\sum_{j=1}^{n}w_{i,j}^{2}-{\left(\sum_{j=1}^{n}w_{i,j}\right)}^{2}\right]}{n-1}}}$$

In the equation, $x_{j}$ represents the species richness value of a specific county j, $w_{i,j}$ represents the spatial weight between specific counties i and j, n is the total number of counties, and

(2) $$\bar{X}=\frac{\sum_{j=1}^{n}x_{j}}{n}\;S=\sqrt{\frac{\sum_{j=1}^{n}x_{j}^{2}}{n}-{\left(\bar{X}\right)}^{2}}$$

The $G_{i}^{*}$ statistic represents the z-score; therefore, further calculation is unnecessary.

In particular, we utilized the Getis-Ord Gi* hotspot analysis tool with the aforementioned four richness distributions as input features. In conceptualizing spatial relationships, the inverse distance squared method was employed using the Euclidean distance as the distance metric. The distributions of PTDS hotspot areas, endemic species hotspot areas, threatened species hotspot areas, and key protected wild plant hotspot areas were extracted as output feature classes to determine the spatial distributions of z-scores and their significance [42]. We further refined the county-level distributions of each species and $G_{i}^{*}$ into 5 km × 5 km grids. This step was primarily aimed at enhancing the precision of optimizing the boundaries of natural reserves using land-use data in subsequent analyses.

### 2.4. Conservation Gap and Conservation Optimization

Attribute data editing and analysis techniques were employed to process the attribute data of hotspot areas. Through the mapping function in ArcGIS, attribute data were associated with spatial locations to conduct a spatial overlay analysis. The distribution results of the analysis of spatial congruence (ASC) provided information on the spatial relationships and degree of overlap between hotspot areas. The consistency of hotspot areas was evaluated based on spatial overlap and protection efficiency analyses. By considering both spatial overlap and protection efficiency, a preliminary plan for optimizing the conservation areas in the Qinling Mountains of Shaanxi Province was determined. Subsequently, the boundary map of the natural reserves in the Qinling Mountains of Shaanxi Province was obtained from the China Natural Reserve Specimen Resource Sharing Platform (http://cnpapc.zrbhq.cn/, accessed on 27 November 2024) to validate the rationality of the preliminary plan for optimized conservation areas. The land-use data of the Qinling Mountains in Shaanxi Province in 2020 (sourced from the Shaanxi Provincial Department of Natural Resources) were obtained and resampled into a 5 km × 5 km grid using ASC to enhance the protection range of the preliminary plan for optimized conservation areas. This involved removing intensive agricultural areas, dense urban areas, rivers, lakes, and other land features from the preliminary plan. Finally, the conservation gaps in the Qinling Mountains of Shaanxi Province were identified.

All analyses were conducted using ArcGIS 10.8 (Esri, Redlands, CA, USA). Administrative boundary maps of China were downloaded from the China Geographic Information Center (http://www.ngcc.cn/, accessed on 28 November 2024). The Digital Elevation Model (DEM) was sourced from the Geospatial Data Cloud (GDC) with a resolution of 30 arc seconds (https://www.gscloud.cn/#page1, accessed on 28 November 2024).

## 3. Results

### 3.1. Composition of Phenotypic Trait Diversity Species

In the Qinling Mountains of Shaanxi Province, a total of 1923 PTDS were identified, belonging to 138 families and 634 genera (species list in Supplementary Materials). In terms of family composition, families with over 40 species include Rosaceae, Ranunculaceae, Fabaceae, Asteraceae, Gramineae, Liliaceae, Caprifoliaceae, Cyperaceae, Umbelliferae, Compositae, and Lamiaceae, totaling 866 species. Families with 30–39 species include Cruciferae, Ericaceae, Celastraceae, Oleaceae, Orchidaceae, Polygonaceae, Berberidaceae, and Hydrangeaceae, totaling 261 species. Families with 10–29 species include 29 families with 483 species. Families with 2–9 species include 59 families with 282 species. Thirty-one families have only one species. Regarding the genus composition, genera with over 30 species include *Carex*, *Rosa*, *Rubus*, *Clematis*, and *Spiraea*, totaling 188 species. Genera with 20–29 species include *Rhododendron*, *Amygdalus*, *Euonymus*, *Lonicera*, *Saussurea*, *Berberis*, and *Ribes*, totaling 161 species. Genera with 10–19 species include 23 genera with 305 species. Genera with 2–9 species include 265 genera with 935 species. There are 334 genera with only one species.

According to the classification based on species life forms, there are 193 tree species, accounting for 10% of the total species. Among them, there are 170 deciduous tree species and 23 evergreen tree species. There are 542 shrub species, constituting 28.2% of the total species, including 419 deciduous shrub species and 123 evergreen shrub species. There are 127 vine species, representing 6.6% of the total species, with 107 deciduous vine species and 20 evergreen vine species. Finally, there are 1061 herb species, comprising 55.2% of the total species, among which there are 138 annual herb species, 51 biennial herb species, and 872 perennial herb species (Table 1).

Ultimately, 1864 species with flower color phenotypic traits were recorded, belonging to 133 families and 624 genera. Among these species, white and yellow coloration of flowers are the most common, with 1005 species, representing 53.9% of the total (Table 2). The flowering seasons primarily occur in spring and summer, with 1773 species flowering during these seasons, accounting for 95.1% of the total (Table 3). There are 457 species with both flowers and fruits, belonging to 74 families and 212 genera, accounting for 23.8% of the total. Additionally, 1332 species with fruit color phenotypic traits were identified, belonging to 118 families and 483 genera, representing 69.3% of the total. Among them, multicolor fruits and red fruits are the most abundant, totaling 980 species, accounting for 73.6% of the total (Table 4). Fruit ripening seasons are predominantly in summer and autumn (Table 5).

### 3.2. Distribution Patterns of the Four Types of Plant Data

**Figure 3:**
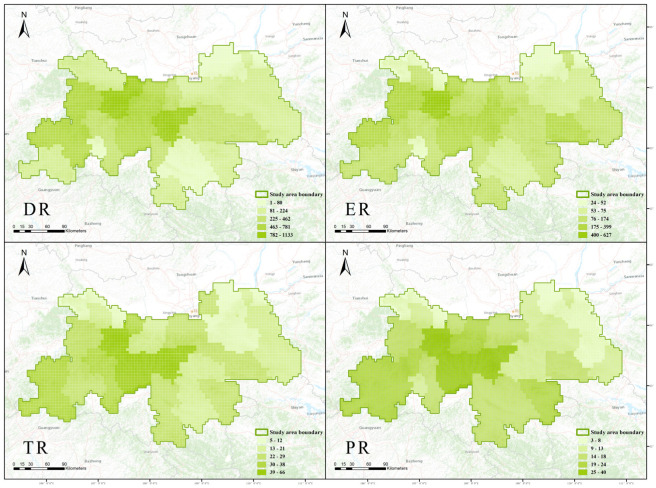
The distribution of Phenotypic Trait Diversity Species richness (DR), endemic species richness (ER), threatened species richness (TR) and protected species richness (RR) in Shaanxi Qinling. DR comprises 1923 species of PTDS (Phenotypic Trait Diversity Species) distributed across various counties and districts in the Shaanxi Qinling Mountains. ER includes 1838 ES (endemic species) distributed across various counties and districts. TR consists of 190 TS (threatened species) distributed across various counties and districts. PR encompasses 119 PS (protected species) distributed across various counties and districts. The figures illustrate the distribution quantities of different plant data across various counties and districts in the Shaanxi Qinling Mountains. The greener the color, the higher the richness of this plant data in the county or district.

Using the species richness algorithm, we identified regions with high and low species richness for the four datasets (Figure 3). The areas with the highest species richness for PTDS are Taibai and Mei, accounting for 58.9% and 47.2% of the total PTDS, respectively. Regions with relatively high species richness include Ningshan, Foping, Zhouzhi, and Yang (Figure 3; DR). Taibai has the highest richness of endemic species, accounting for 34.1% of the total. Regions with relatively high richness include Ningshan, Meixian, Foping, Huyi, Feng, Luoyang, Yang, and Shanyang (Figure 3; ER). The areas with the highest richness of threatened species are Taibai, accounting for 34.7% of the total threatened species, Foping, accounting for 31.6%, Yang, accounting for 25.8%, and Ningshan, accounting for 25.2%. Regions with relatively high richness include Luoyang, Mei, Liuba, Ningqiang, and Chenggu (Figure 3; TR). The areas with the highest richness of protected species are Taibai, accounting for 33.3% of the total protected species, Foping, accounting for 32.5%, and Ningshan, accounting for 23.7%. Regions with relatively high richness include Yang, Liuba, Luoyang, Ningqiang, and Zhouzhi (Figure 3; PR). Taibai is the area with the highest species richness in PTDS, endemic species, and protected species among the three datasets. Foping is the area with the highest richness of threatened species and protected species simultaneously.

### 3.3. Distribution and Consistency of Hotspots for Phenotypic Trait Diversity Species Compared to the Other Three Hotspots

**Figure 4:**
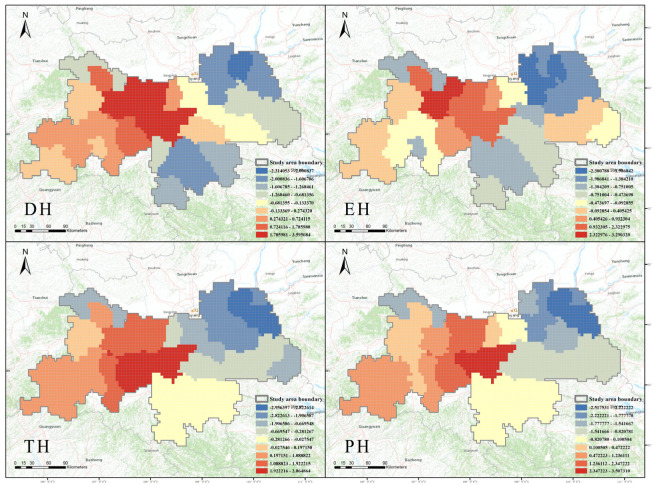
The distribution of Phenotypic Trait Diversity Species hotspots (DH), endemic species hotspots (EH), threatened species hotspots (TH) and protected species hotspots (PH) in Shaanxi Qinling. These four figures represent hotspot analysis of PTDS (Phenotypic Trait Diversity Species), ES (endemic species), TS (threatened species), and PS (protected species) using the Getis-Ord Gi* method. The numbers shown in the figures are the statistical z-scores, where higher (or lower) z-scores indicate higher (or lower) levels of clustering. Specifically, areas with redder colors represent hotspots for plant conservation, while areas with bluer colors represent coldspots for plant conservation.

The analysis using Getis-Ord Gi* revealed the hotspots for PTDS in areas such as Taibai, Mei, Foping, Zhouzhi, Ningshan, Yang, Baoji, Huayin, Chenggu, Liuba, Mian, and Luoyang (z ≥ 0.274321) (Figure 4; DH). Hotspots for endemic species were identified in Taibai, Mei, Foping, Zhouzhi, Ningshan, Baoji, Huayin, and Yang (z ≥ 0.405426) (Figure 4; EH). Areas with threatened species hotspots included Ningshan, Foping, Yang, Taibai, Mei, Zhouzhi, Chenggu, Baoji, Liuba, Mian, Luoyang, Ningqiang, and Hantai (z ≥ 0.197151) (Figure 4; TH). The hotspots for protected species were observed in Ningshan, Foping, Zhouzhi, Yang, Taibai, Mei, Chenggu, Mian, Luoyang, and Ningqiang (z ≥ 0.472223) (Figure 4; PH).

**Figure 5:**
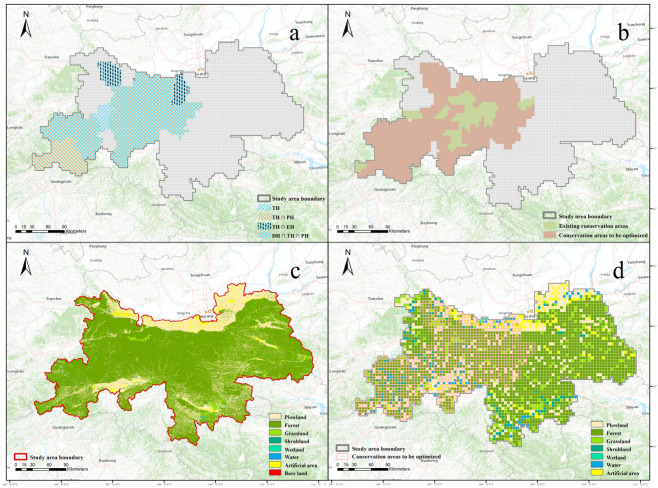
(**a**) The analysis of spatial congruence (ASC) of Phenotypic Trait Diversity Species hotspots (OH), endemic species hotspots (EH), threatened species hotspots (TH), protected species hotspots (PH) based on Getis-Ord Gi* statistics. (**b**) The comparison between the conservation areas to be optimized determined by the four types of data in figure a and the existing conservation areas. (**c**) Land-use map of the Qinling Mountains in Shaanxi Province in 2020. (**d**) The resampled land-use map and the boundaries of the areas earmarked for optimization of protection.

The results of the spatial consistency analysis (Figure 5a) indicated that there were 14 counties identified as hotspots based on the analysis of the four datasets. Among these, nine counties were identified as hotspots for all three categories (DH∩TH∩PH). Four counties were identified as hotspots for two categories simultaneously (TH∩PH, DH∩TH, DH∩EH). Only one county was identified as a hotspot for one category (TH).

The analysis based on the four datasets collectively identified fourteen counties with conservation gaps (Figure 5b), with thirteen counties identified through PTDS data analysis, seven counties through endemic species data, fourteen counties through threatened species data, and nine counties through protected species data. The area of conservation gaps identified by threatened species was the highest, covering 95.8% of the total area; followed by PTDS, covering 87.4% of the total area; then protected species, covering 83.8% of the total area; and finally endemic species, covering 56.3% of the total area. The results indicate that the area of conservation gaps identified by PTDS exceeds those identified by commonly used protected and endemic species in current conservation studies, suggesting that PTDS could serve as research data to fill in the gaps in conservation areas.

### 3.4. Priority Conservation Areas for Plant Species in the Qinling Mountains, Shaanxi

**Figure 6:**
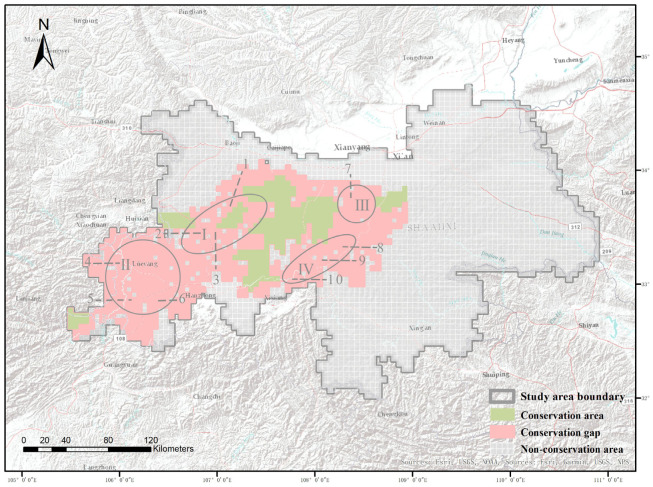
The final boundaries of the areas earmarked for the optimization of protection and the existing protected areas are as follows: Area I is located around the main peak of Mount Taibai in the Qinling Mountains, mainly covering Taibai (1), Liuba (2), and Chenggu (3) Counties. Area II is situated at the junction of the Qinling and Bashan mountains, primarily covering Lueyang (4), Ningqiang (5), and Mianxian (6) Counties. Area III is located around Mount Hua and its vicinity, mainly covering Huayin (7) County. Area IV is situated along the main ridge of the Qinling Mountains, primarily covering Ningshan (8), Foping (9), and Yang (10) Counties. A non-conservation area refers to regions with low conservation value.

We conducted a resampling of the 2020 land-use data in the Qinling Mountains of Shaanxi Province, removing areas with intensive agricultural and construction land use, as well as rivers and lakes, from the areas earmarked for optimization in conservation efforts. This process allowed us to delineate the boundaries of the areas earmarked for optimization (Figure 5c,d). Utilizing the geographical boundaries of existing conservation areas in comparison with the newly obtained areas earmarked for optimization, the analysis revealed that 94.2% of the existing conservation area falls within the boundaries of the areas marked for optimization. However, the existing conservation areas only constitute 13.3% of the total area marked for optimization (Figure 6). Furthermore, five counties with significant conservation hotspots, namely, Ningqiang, Luoyang, Mian, Baoji, and Huayin, lack designated conservation areas. The areas earmarked for optimization that are not covered by existing conservation areas are primarily concentrated in four regions: around the highest peak of the Qinling Mountains, Mount Taibai; around Mount Hua, one of the main peaks of the Qinling Mountains; along the main ridge of the Qinling Mountains; and at the intersection of the Qinling and Bashan mountains (Figure 6).

## 4. Discussion

### 4.1. Composition of Phenotypic Trait Diversity Species and Their Roles in Conservation Areas

The remarkable diversity and composition of Phenotypic Trait Diversity Species (PTDS) identified in the Qinling Mountains underscore the ecological richness and complexity of this region. With a total of 1923 PTDS identified, encompassing a wide array of families and genera, this study reveals the profound botanical diversity harbored within this mountainous landscape [43,44]. Notably, the prevalence of monotypic genera, accounting for over half of the total genera identified, highlights the unique evolutionary pathways and ecological niches occupied by various plant species. Similarly, genera with 2–5 species underscore the diverse ecological strategies employed by plant communities to thrive in the various habitats of the Qinling Mountains [52].

The observed overlap between flowering and fruiting seasons and the activity peaks of pollinators and herbivores underscores the significance of flower and fruit colors in mediating plant–animal interactions [53,54]. Flower and fruit colors serve as vital cues for attracting pollinators and seed dispersers, thereby facilitating successful reproduction and dispersal strategies among plant populations [55]. In Li et al.’s study on animal diversity in the Qinling Mountains, regions with high animal diversity closely align with the hotspots identified in this study using integrated PTDS data, elucidating the interaction between animal foraging and plant dispersal [56,57]. The co-evolutionary dynamics between plants and their animal counterparts have likely shaped the observed patterns of flower and fruit color diversity within PTDS, contributing to the resilience and persistence of plant communities in the face of environmental challenges.

Furthermore, the correlations between flower and fruit color diversity and plant survival underscore the pivotal role of phenotypic traits in shaping ecosystem structure and function [58]. Regions characterized by a higher diversity of flower and fruit colors may exhibit greater ecological stability and resilience, as evidenced by the enhanced survival chances of plant populations [59]. Consequently, the conservation and management of PTDS-rich areas in the Qinling Mountains are paramount for safeguarding the region’s botanical heritage and maintaining ecosystem integrity in the face of ongoing environmental changes and anthropogenic pressures.

### 4.2. Species Diversity, Hotspots, and Conservation Gaps in the Qinling Mountains of Shaanxi

The distributions of PTDS, ES, TS, and PS across counties and districts in the Qinling Mountains are uneven and primarily concentrated in the high-altitude regions of the central and western areas. Research by Li et al. (2022) found that higher altitudes and complex ecological environments create favorable conditions for plant growth. Conversely, lower-altitude areas experience greater human disturbance, resulting in generally lower biodiversity compared to higher-altitude regions [60]. Zhang et al. (2017), through a survey of cushion plant richness in the Qinling region, found that areas with high cushion plant richness are concentrated in the high-altitude central and western Qinling [16], corroborating our study’s findings. By integrating PTDS (Plant Flower and Fruit Color Trait Diversity Species) data for hotspot identification, this study provides a new dimension distinct from traditional conservation planning perspectives. Compared to widely used plant category indicators such as ES, TS, and PS, PTDS data directly reflect the interaction potential and spatial patterns of plants with key mutualists like pollinators and seed dispersers. Losada et al. (2023) found that regions with high plant diversity often exhibit high diversity in animals and microorganisms, which is critical for maintaining ecological stability [59]. This direct characterization of key drivers of ecosystem function, which traditional species category data struggle to capture effectively, aids in identifying core areas that sustain the stability of ecological processes [61,62,63].

As carriers of regionally unique evolutionary histories, ES are critical, TS represent urgent conservation priorities, and PS hold statutory protection status. The importance of these species categories has been well-established by numerous studies and is indispensable for conservation planning [64]. Therefore, combining PTDS, which reflects key ecological functions, with ES, TS, and PS indicators can provide a more comprehensive and resilient theoretical foundation for conservation area networks, offering robust scientific support for optimizing the layout of conservation areas in the Qinling Mountains. Hotspot areas identified by integrating multidimensional PTDS, ES, TS, and PS datasets show significant spatial consistency with existing conservation areas, further confirming that the comprehensive use of datasets reflecting different conservation values can optimize existing protected areas [65,66]. These hotspot areas provide critical spatial guidance for optimizing conservation networks, establishing new botanical gardens, and planning national parks [67]. However, a core challenge in optimizing conservation networks lies in unified planning and management across administrative boundaries. While large-scale hotspot and gap analyses (such as this study) provide a macro blueprint, conservation governance fragmented by administrative jurisdictions often leads to gaps in protecting key ecological corridors and habitats across counties, undermining ecosystem integrity and resilience [27].

To address this institutional bottleneck, China’s ongoing national park system offers a critical solution [68]. As one of the first pilot projects, the Qinling National Park aims to implement holistic protection at a supra-administrative scale based on ecosystem integrity and connectivity, rather than administrative boundaries [12,69]. This framework provides a fundamental solution for integrating the cross-county hotspots and ecological corridors (e.g., key connective zones between protected areas) identified in this study, which are currently fragmented by administrative divisions. Although the full maturation of the national park system will take time, the Qinling pilot has already taken substantial steps toward overcoming administrative fragmentation [70]. The multidimensional, spatially explicit gap identification method provided in this study can directly support boundary delineation, functional zoning, and corridor restoration priority decisions for national parks and regional conservation networks. Future conservation planning should continue to rely on such comprehensive data to dynamically optimize large-scale ecosystem management, synergistically ensuring the integrity of biodiversity representation and key ecological processes [69,71].

### 4.3. Application of Phenotypic Trait Diversity in Hotspot Identification and Recognizing Conservation Gaps

Since Diaz and Cabido (2001) proposed the concept of using species richness data to optimize ecological conservation, threatened species, endemic species, and protected species have gradually become widely used and validated datasets for identifying hotspot gaps [72,73]. For instance, Yang et al. (2021) utilized traditional datasets for identifying conservation gaps (ES, TS, and PS) in the Kanas region of southern China, confirming the foundational roles of these indicators in conservation planning [64]. The proportion of hotspot areas identified by PTDS data ranks second only to threatened species areas, marking these regions as one of the priority areas for conservation optimization. This result underscores the effectiveness of PTDS as an independent indicator in identifying high conservation value areas, with its significance lying in directly reflecting key potential areas for sustaining ecosystem functions (e.g., pollination and seed dispersal networks), providing a new dimension distinct from traditional threat or endemism indicators for conservation planning [39].

Phenotypic traits are rooted in fundamental evolutionary principles, demonstrating how individuals (genotypes) adapt and succeed through variation in specific environments [74]. Sobral et al. (2021) suggest that these traits exhibit plasticity, with genotypes producing different phenotypic characteristics in response to environmental changes, supporting this study’s hypothesis regarding trait–environment associations [36,75]. For example, individual selection for flower color may stem from variations in ultraviolet radiation, temperature fluctuations, or changes in biotic interactions due to mutualistic or competitive relationships [76]. While this study does not delve into specific causes, it emphasizes the complex relationships between phenotypic traits and environmental variables. Furthermore, phenotypic traits interact among individuals and across traits, and these interactions may vary over time and space [77].

### 4.4. Uncertainties

The data utilized in this study were obtained from field surveys conducted by other research teams and subsequently published or shared on relevant websites. However, many areas in the Qinling Mountains of Shaanxi have not been thoroughly explored, especially remote or inaccessible mountainous regions. Therefore, the data on PTDS may not be comprehensive, and there is a possibility of underestimating the biodiversity analysis based on the available data. This is a common issue in large-scale biodiversity studies, and with the ongoing supplementation of regional biodiversity inventories, this situation is expected to improve. The plant phenotypic traits selected for this study include flower color and fruit color, both of which are representative in studies of biological reproduction and survival. However, selecting additional plant phenotypic traits may be more conducive to determining the scope of the areas for optimization in conservation zones.

## 5. Conclusions

Identifying plant diversity hotspots and conservation gaps plays a crucial role in the planning and optimization of protected areas. However, the Qinling Mountains in Shaanxi still face challenges such as an uneven distribution, cross-administrative management, and unified planning. The use of PTDS fills the gap in optimizing conservation through trait diversity, achieving spatial coupling of ecological functional traits and species conservation priorities at a regional scale through plant–animal interaction potential. The conservation gaps identified by PTDS surpass those determined by the commonly used ES and PS data in current conservation studies. Integrating PTDS hotspots, endemic hotspots, threatened hotspots, and protected hotspots (DH∩EH∩TH∩PH) identifies priority protected areas for plant diversity in the Shaanxi Qinling Mountains. This approach not only overcomes the limitations of traditional indicators but also systematically reveals key functional areas and potential vulnerabilities for maintaining Qinling biodiversity. It directly supports the optimization goals of protected areas, focusing on “expansion, connectivity, and quality improvement”, providing an irreplaceable role in conservation planning oriented toward ecological function integrity. Additionally, this study established the Shaanxi Qinling Plant Conservation Database (including 1923 PTDS, 1838 ES, 190 TS, and 119 PS), providing a verifiable “function–structure” integrated dataset for biodiversity conservation in the Shaanxi Qinling region, advancing conservation planning from species inventory management to ecosystem optimization.

## 6. Suggestions and Outlook

To address the identified conservation gaps, we propose the following tiered conservation measures:

(1) Priority integration into national parks—incorporate contiguous gap areas into the boundary optimization plans of national parks, establishing peripheral buffer zones and ecological corridors to enhance habitat connectivity.
(2) Community co-governance—implement an “ecological stewardship public welfare position” mechanism in fragmented conservation gap areas, encouraging local residents to participate in monitoring and patrolling, while promoting ecological planting (e.g., fungi and medicinal plants) as an alternative to traditional resource-dependent livelihoods.
(3) Smart monitoring network—utilize GIS and remote sensing technologies to establish a biodiversity database for conservation gap areas, dynamically assessing the impacts of human disturbances and the effectiveness of ecological restoration.

The integration of hotspot identification and phenotypic trait research provides preliminary insights into the impacts of phenotypic traits on plant diversity. However, to comprehensively explore these impacts, future studies should focus on trait richness (the total breadth of phenotypic traits within a system), variation, and regularity. Future research should delve deeper into these aspects to uncover the complex dynamics governing plant diversity and ecosystem functions.

## Figures and Tables

**Table 1:** Classification and proportions of life forms of Phenotypic Trait Diversity Species (PTDS) based on flower and fruit colors in the Qinling Mountain.

Life Form		Families		Genera		Species	
		Numbers	Proportion of Total Families/%	Numbers	Proportion of Total Genera/%	Numbers	Proportion of Total Species/%
Tree	Deciduous tree	36	26.7	74	11.7	170	8.8
	Evergreen tree	12	8.7	12	1.9	23	1.2
Shrub	Deciduous shrub	40	29.0	98	15.5	419	21.8
	Evergreen shrub	27	19.6	45	7.1	123	6.4
Liana	Deciduous liana	14	10.1	27	4.3	107	5.6
	Evergreen liana	12	8.7	14	2.2	20	1.0
Herbs	Annual herbs	44	31.9	101	16.0	138	7.2
	Annual and biennial herbs	16	11.6	38	6.0	51	2.7
	Perennial herbs	87	63.0	362	57.1	872	45.3

**Table 2:** Flower color classification and proportion of Phenotypic Trait Diversity Species (PTDS) in the Shaanxi Qinling Mountains.

Color of Flowers	Families		Genera		Species	
	Numbers	Proportion of Total Families/%	Numbers	Proportion of Total Genera/%	Numbers	Proportion of Total Species/%
White	72	54.1	218	34.9	589	31.6
Yellow	81	60.9	203	32.5	423	22.7
Purple	61	45.7	143	22.9	305	16.3
Green	45	33.8	73	11.7	154	8.3
Multicolor	42	31.6	77	12.3	114	6.1
Pink	25	18.8	45	7.2	93	5.0
Red	39	29.3	66	10.6	90	4.8

**Table 3:** Flowering seasons and proportions of plants with flower color phenotypic traits in the Qinling Mountains, Shaanxi.

Season of Observing Flowers	Families		Genera		Species	
	Numbers	Proportion of Total Families/%	Numbers	Proportion of Total Genera/%	Numbers	Proportion of Total Species/%
Spring	110	82.7	363	58.2	821	44.0
Summer	96	72.2	380	60.1	953	51.1
Autumn	24	18.0	41	6.6	70	3.8
Winter	7	5.3	8	1.3	10	0.5

**Table 4:** Fruit color classification and proportion of Phenotypic Trait Diversity Species (PTDS) in the Shaanxi Qinling Mountains.

Color of Fruits	Families		Genera		Species	
	Numbers	Proportion of Total Families/%	Numbers	Proportion of Total Genera/%	Numbers	Proportion of Total Species/%
Multicolor (Fruit shape)	96	81.4	349	72.3	697	52.3
Red	33	28.0	61	12.6	283	21.2
Black	34	28.8	60	12.4	133	10.0
Brown	23	19.5	38	7.9	100	7.5
Purple	16	13.6	29	6.0	67	5.0
Yellow	20	16.9	29	6.0	52	3.9

**Table 5:** Flowering seasons and proportions of plants with fruit color phenotypic traits in the Qinling Mountains, Shaanxi.

Season of Observing Fruits	Families		Genera		Species	
	Numbers	Proportion of Total Families/%	Numbers	Proportion of Total Genera/%	Numbers	Proportion of Total Species/%
Spring	22	18.6	45	9.3	87	6.5
Summer	88	74.6	282	58.4	648	48.6
Autumn	88	74.6	262	54.2	581	43.6
Winter	6	5.1	6	1.2	6	0.0

## Data Availability

The data are not publicly available due to privacy or ethical restrictions.

## Supplementary Materials

The following supporting information can be downloaded at https://www.mdpi.com/article/10.3390/plants14142130/s1: see S1 and S2 in the attached documents.

## Abbreviations

The following abbreviations are used in this manuscript:

PTDS	Phenotypic Trait Diversity Species
ES	Endemic species
TS	Threatened species
PS	Protected species
