# Supplementary material for: Enhancing Conservation Efforts in the Qinling Mountains Through Phenotypic Trait Diversity Optimization
Source: Plants (Basel). 2025 Jul 10;14(14):2130. doi: 10.3390/plants14142130 (PMC12300060; doi:10.3390/plants14142130)
Supplement: Supplementary file 1 [file plants-14-02130-s001.zip › S1 (PTDS).pdf]

Families	Genera	Species	Life form	Ornamental parts	Color of flowers	Season of observing flowers	Color of fruits	Season of observing fruits
Acanthaceae	*Rostellularia*	*Rostellularia procumbens var. procumbens*	Annual herbs	Flower	Pink	SU、AU		
Actinidiaceae	*Clematoclethra*	*Clematoclethra scandens*	Deciduous liana	Flower	White	SU		
Actinidiaceae	*Clematoclethra*	*Clematoclethra scandens subsp. hemsleyi*	Deciduous liana	Flower	White	SU		
Actinidiaceae	*Clematoclethra*	*Clematoclethra scandens subsp. actinidioides*	Deciduous liana	Flower	White	SU		
Actinidiaceae	*Amygdalus*	*Actinidia arguta var. giraldii*	Deciduous liana	Flower	Green	SP	Brown	AU
Actinidiaceae	*Amygdalus*	*Actinidia chinensis*	Deciduous liana	Flower	White	SP	Brown	AU
Actinidiaceae	*Amygdalus*	*Actinidia polygama*	Deciduous liana	Flower	White	SP	Brown	AU
Actinidiaceae	*Amygdalus*	*Actinidia callosa var. henryi*	Deciduous liana	Flower	White	SP	Brown	AU
Actinidiaceae	*Amygdalus*	*Actinidia chinensis var. deliciosa*	Deciduous liana	Flower	White	SP	Brown	AU
Actinidiaceae	*Amygdalus*	*Actinidia kolomikta*	Deciduous liana	Flower	White	SP	Brown	AU
Actinidiaceae	*Amygdalus*	*Actinidia tetramera*	Deciduous liana	Flower	White	SP	Brown	AU
Actinidiaceae	*Amygdalus*	*Actinidia arguta*	Deciduous liana	Flower	Green	SP	Brown	AU
Actinidiaceae	*Amygdalus*	*Actinidia melanandra*	Deciduous liana	Flower	White	SP	Brown	AU
Aizoac	*Mollu*	*Mollugo stricta*	Annual	Flowe	Gree	SU	Multicol	SU、AU

eae	*go*		herbs	r	n		our(Fruit shape)	
Alismataceae	*Alisma*	*Alisma plantago-aquatica*	Perennial herbs	Flower	White	SP、SU	Multicolour(Fruit shape)	SU
Alismataceae	*Alisma*	*Alisma orientale*	Perennial herbs	Flower、Fruit	Multicolour	SP、SU	Multicolour(Fruit shape)	SU、AU
Alismataceae	*Sagittaria*	*Sagittaria pygmaea*	Annual herbs	Flower、Fruit	White	SU	Multicolour(Fruit shape)	SU、AU
Alismataceae	*Sagittaria*	*Sagittaria trifolia*	Perennial herbs	Flower、Fruit	Multicolour	SP、SU	Multicolour(Fruit shape)	SU、AU
Amaranthaceae	*Dysphania*	*Dysphania aristata*	Annual herbs	Fruit			Purple	AU
Amaranthaceae	*Amaranthus*	*Amaranthus caudatus*	Perennial herbs	Flower、Fruit	Red	SU	Multicolour(Fruit shape)	AU
Amaranthaceae	*Celosia*	*Celosia argentea*	Annual herbs	Flower	Red	SP	Multicolour(Fruit shape)	AU
Amaranthaceae	*Achyranthes*	*Achyranthes bidentata*	Perennial herbs	Flower、Fruit		SU、AU	Brown	AU
Amaranthaceae	*Amaranthus*	*Amaranthus blitum*	Annual herbs	Flower、Fruit	Green	SU	Multicolour(Fruit shape)	SU、AU
Amaranthaceae	*Amaranthus*	*Amaranthus cruentus*	Annual herbs	Flower、Fruit	Purple	SU	Multicolour(Fruit shape)	AU
Amaranthaceae	*Amaranthus*	*Amaranthus retroflexus*	Annual herbs	Flower、Fruit	White	SU	Multicolour(Fruit shape)	SU、AU
Amaranthaceae	*Amaranthus*	*Amaranthus roxburghianus*	Annual herbs	Flower、Fruit		SU	Multicolour(Fruit shape)	SU、AU
Amaranthaceae	*Amaranthus*	*Amaranthus tricolor*	Annual herbs	Flower、Fruit	Green	SP、SU	Multicolour(Fruit shape)	SU、AU
Amaryllidaceae	*Lycoris*	*Lycoris radiata*	Perennial herbs	Flower	Red	SU	Multicolour(Fruit shape)	AU

Amaryllidaceae	*Lycoris*	*Lycoris chinensis*	Perennial herbs	Flower	Yellow	SU	Multicolour(Fruit shape)	AU
Amaryllidaceae	*Lycoris*	*Lycoris aurea*	Perennial herbs	Flower	Yellow	SU	Multicolour(Fruit shape)	AU
Amaryllidaceae	*Allium*	*Allium victorialis*	Perennial herbs	Flower	White	SU	Multicolour(Fruit shape)	AU
Amaryllidaceae	*Allium*	*Allium prattii*	Perennial herbs	Flower	Purple	SU	Multicolour(Fruit shape)	AU
Amaryllidaceae	*Allium*	*Allium chrysanthum*	Perennial herbs	Flower	Yellow	SU	Multicolour(Fruit shape)	AU
Anacardiaceae	*Rhus*	*Rhus potaninii*	Deciduous tree	Flower	White	SU	Red	AU
Anacardiaceae	*Rhus*	*Rhus punjabensis var. sinica*	Deciduous tree	Flower	White	SU	Red	AU
Anacardiaceae	*Rhus*	*Rhus chinensis*	Deciduous tree	Flower	White	SU	Red	AU
Anacardiaceae	*Toxicodendron*	*Toxicodendron sylvestre*	Deciduous tree	Flower	Yellow	SP		
Anacardiaceae	*Toxicodendron*	*Toxicodendron succedaneum*	Deciduous tree	Flower	Yellow	SU		
Anacardiaceae	*Toxicodendron*	*Toxicodendron vernicifluum*	Deciduous tree	Flower	Yellow	SU		
Anacardiaceae	*Cotinus*	*Cotinus coggygria var. cinerea*	Deciduous shrub	Flower	Pink	SP		
Anacardiaceae	*Cotinus*	*Cotinus coggygria var. glaucophylla*	Deciduous shrub	Flower	Pink	SP		
Anacardiaceae	*Cotinus*	*Cotinus coggygria var. pubescens*	Deciduous shrub	Flower	Pink	SP		
Anacardiace	*Pistacia*	*Pistacia chinensis*	Deciduous tree	Flower	Yellow	SP	Red	AU

ae								
Apocynaceae	*Tylophora*	*Tylophora flexuosa*	Deciduous liana	Flower	White	SP		
Apocynaceae	*Dregea*	*Dregea yunnanensis*	Deciduous liana	Flower	White	SP		
Apocynaceae	*Dregea*	*Dregea sinensis var. corrugata*	Deciduous liana	Flower	Purple	SP		
Apocynaceae	*Dregea*	*Dregea sinensis*	Deciduous liana	Flower	Purple	SP		
Apocynaceae	*Sindechites*	*Sindechites henryi*	Deciduous liana	Flower	White	SU		
Apocynaceae	*Trachelospermum*	*Trachelospermum axillare*	Evergreen liana	Flower	Purple	SU		
Apocynaceae	*Trachelospermum*	*Trachelospermum jasminoides*	Evergreen liana	Flower	White	SP		
Apocynaceae	*Trachelospermum*	*Trachelospermum asiaticum*	Evergreen liana	Flower	Yellow	SU		
Apocynaceae	*Periploca*	*Periploca forrestii*	Deciduous liana	Flower	Yellow	SP		
Apocynaceae	*Cynanchum*	*Cynanchum chinense*	Perennial herbs	Flower 、Fruit	White	SU	Multicolour(Fruit shape)	AU
Apocynaceae	*Cynanchum*	*Cynanchum atratum*	Perennial herbs	Flower	Purple	SU	Multicolour(Fruit shape)	AU
Apocynaceae	*Cynanchum*	*Cynanchum amplexicaule*	Perennial herbs	Flower	Multicolour	SP	Multicolour(Fruit shape)	AU
Aquifoliaceae	*Ilex*	*Ilex fargesii*	Evergreen tree	Flower 、Fruit	White	SP	Red	AU
Aquifo	*Ilex*	*Ilex fargesii var.*	Evergree	Flowe	Whit	SP	Red	AU

liaceae		*angustifolia*	n tree	r、Fruit	e			
Aquifoliaceae	*Ilex*	*Ilex yunnanensis*	Evergreen shrub	Flower、Fruit	White	SU	Red	AU
Aquifoliaceae	*Ilex*	*Ilex yunnanensis var. gentilis*	Evergreen shrub	Flower、Fruit	White	SU	Red	AU
Aquifoliaceae	*Ilex*	*Ilex pedunculosa*	Evergreen shrub	Flower、Fruit	White	SU	Red	SU
Aquifoliaceae	*Ilex*	*Ilex szechwanensis*	Evergreen shrub	Flower、Fruit	White	SU	Black	AU
Aquifoliaceae	*Ilex*	*Ilex wilsonii*	Evergreen shrub	Flower、Fruit	White	SU	Red	AU
Aquifoliaceae	*Ilex*	*Ilex macrocarpa var. reevesiae*	Evergreen tree	Flower、Fruit	White	SP	Black	AU
Aquifoliaceae	*Ilex*	*Ilex corallina*	Evergreen shrub	Flower、Fruit	Green	SP	Red	AU
Aquifoliaceae	*Ilex*	*Ilex dunniana*	Evergreen tree	Flower、Fruit	Green	SP	Red	AU
Aquifoliaceae	*Ilex*	*Ilex pernyi*	Evergreen shrub	Flower、Fruit	Yellow	SP	Red	AU
Aquifoliaceae	*Ilex*	*Ilex chinensis*	Evergreen tree	Flower、Fruit	Purple	SP	Red	SU
Aquifoliaceae	*Ilex*	*Ilex macrocarpa*	Deciduous tree	Flower、Fruit	White	SP	Black	AU
Araceae	*Arisaema*	*Arisaema erubescens*	Perennial herbs	Flower、Fruit	Multicolour	SP、SU	Multicolour(Fruit shape)	AU
Araceae	*Pinellia*	*Pinellia pedatisecta*	Perennial herbs	Flower、Fruit	Multicolour	SU	Multicolour(Fruit shape)	AU
Araceae	*Acorus*	*Acorus calamus*	Perennial herbs	Flower、Fruit	Yellow	SP、SU	Red	

Araceae	*Amorphophallus*	*Amorphophallus*	Perennial herbs	Flower、Fruit	Red	SP、SU	Yellow	AU
Araceae	*Arisaema*	*Arisaema asperatum*	Perennial herbs	Flower、Fruit	Multicolour	SP、SU	Red	
Araceae	*Arisaema*	*Arisaema fargesii*	Perennial herbs	Flower	Multicolour	SP、SU		
Araceae	*Arisaema*	*Arisaema heterophyllum*	Perennial herbs	Flower、Fruit	Multicolour	SP	Red	SU、AU
Araceae	*Arisaema*	*Arisaema bockii*	Perennial herbs	Flower、Fruit	Multicolour	SP	Yellow	SU、AU
Araceae	*Pinellia*	*Pinellia ternata*	Perennial herbs	Flower、Fruit	Multicolour	SP、SU	Yellow	SU
Araliaceae	*Gamblea*	*Gamblea ciliata var. evodiifolia*	Deciduous shrub	Flower	Yellow	SU	Black	AU
Araliaceae	*Tetrapanax*	*Tetrapanax papyrifer*	Evergreen shrub	Flower	Yellow	AU	Black	WI
Araliaceae	*Hydrocotyle*	*Hydrocotyle sibthorpioides*	Perennial herbs	Flower、Fruit	Green	SP、SU	Multicolour(Fruit shape)	SU、AU
Araliaceae	*Hydrocotyle*	*Hydrocotyle nepalensis*	Perennial herbs	Flower、Fruit	White	SP、SU	Multicolour(Fruit shape)	SU、AU
Araliaceae	*Nothopanax*	*Metapanax davidii*	Evergreen shrub	Flower	White	SU	Black	AU
Araliaceae	*Macropanax*	*Macropanax rosthornii*	Evergreen shrub	Flower	White	SU		
Araliaceae	*Aralia*	*Aralia elata*	Deciduous shrub	Flower、Fruit	Yellow	SU	Black	AU
Araliaceae	*Aralia*	*Aralia continentalis*	Perennial herbs	Fruit			Purple	AU
Araliaceae	*Kalopanax*	*Kalopanax septemlobus*	Deciduous tree	Flower	White	SU		
Araliaceae	*Hedera*	*Hedera nepalensis var. sinensis*	Evergreen liana	Flower、Fruit	Yellow	AU	Red	SP
Aristol	*Asaru*	*Asarum*	Perennia	Flowe	Purpl	SP、SU		

ochiaceae	*m*	*himalaicum*	l herbs	r	e			
Aristolochiaceae	*Saruma*	*Saruma henryi*	Perennial herbs	Flower	Yellow	SP、SU		
Aristolochiaceae	*Aristolochia*	*Aristolochia mollissima*	Deciduous liana	Flower	Yellow	SP		
Aristolochiaceae	*Aristolochia*	*Aristolochia manshuriensis*	Deciduous liana	Flower	Pink	SU	Multicolour(Fruit shape)	AU
Aristolochiaceae	*Aristolochia*	*Aristolochia contorta*	Deciduous liana	Flower	Yellow	SU	Multicolour(Fruit shape)	AU
Asparagaceae	*Hosta*	*Hosta plantaginea*	Perennial herbs	Flower	White	SU、AU	Multicolour(Fruit shape)	SU
Asparagaceae	*Ophiopogon*	*Ophiopogon japonicus*	Perennial herbs	Flower、Fruit	Multicolour	SU	Purple	AU
Asparagaceae	*Asparagus*	*Asparagus schoberioides*	Annual and biennial herbs	Flower、Fruit	Green	SU	Red	AU
Asparagaceae	*Asparagus*	*Asparagus filicinus*	Perennial herbs	Flower、Fruit	Multicolour	SP、SU	Multicolour(Fruit shape)	SU、AU
Asparagaceae	*Liriope*	*Liriope spicata*	Perennial herbs	Flower、Fruit	Purple	SP、SU	Multicolour(Fruit shape)	SU
Asparagaceae	*Convallaria*	*Convallaria majalis*	Perennial herbs	Flower	White	SP、SU	Multicolour(Fruit shape)	SU
Asparagaceae	*Tupistra*	*Campylandra chinensis*	Perennial herbs	Flower、Fruit	Yellow	SP、SU	Purple	AU
Asparagaceae	*Reineckea*	*Reineckea carnea*	Perennial herbs	Flower、Fruit	Pink	SU、AU	Red	AU
Asparagaceae	*Polygonatum*	*Polygonatum sibiricum*	Perennial herbs	Flower、Fruit	White	SP	Black	SU
Asparagace	*Polygonatu*	*Polygonatum odoratum*	Perennial herbs	Flower、	White	SP	Multicolour(Frui	SU

ae	*m*			Fruit			t shape)	
Asteraceae	*Aster*	*Aster tataricus*	Perennial herbs	Flower	Purple	SU	Multicolour(Fruit shape)	AU
Asteraceae	*Aster*	*Aster scaber*	Perennial herbs	Flower、Fruit	White	SU、AU	Multicolour(Fruit shape)	AU
Asteraceae	*Aster*	*Aster ageratoides*	Perennial herbs	Flower	Purple	SU	Multicolour(Fruit shape)	AU
Asteraceae	*Aster*	*Aster pekinensis*	Perennial herbs	Flower	Purple	SU	Multicolour(Fruit shape)	AU
Asteraceae	*Aster*	*Aster lautureanus*	Perennial herbs	Flower	Purple	SU	Multicolour(Fruit shape)	AU
Asteraceae	*Aster*	*Aster indicus*	Perennial herbs	Flower、Fruit	Purple	SP、SU	Multicolour(Fruit shape)	AU
Asteraceae	*Aster*	*Aster hispidus*	Annual and biennial herbs	Flower、Fruit	White	SU、AU	Multicolour(Fruit shape)	SU
Asteraceae	*Aster*	*Aster alpinus*	Perennial herbs	Flower	Red	SU	Multicolour(Fruit shape)	SU
Asteraceae	*Eupatorium*	*Eupatorium japonicum*	Perennial herbs	Flower	White	SU		
Asteraceae	*Eupatorium*	*Eupatorium fortunei*	Perennial herbs	Flower	Purple	SU	Multicolour(Fruit shape)	AU
Asteraceae	*Eupatorium*	*Eupatorium chinense*	Perennial herbs	Flower	White	SU	Multicolour(Fruit shape)	AU
Asteraceae	*Solidago*	*Solidago decurrens*	Perennial herbs	Flower	Yellow	SP、SU	Multicolour(Fruit shape)	AU
Asteraceae	*AlliumScorzonera*	*Scorzonera austriaca*	Perennial herbs	Flower	Yellow	SP		
Asteraceae	*Inula*	*Inula japonica*	Perennial herbs	Flower	Yellow	SU	Multicolour(Fruit shape)	AU

Asteraceae	*Parasenecio*	*Parasenecio auriculatus*	Perennial herbs	Flower	White	SU		
Asteraceae	*Anaphalis*	*Anaphalis sinica*	Perennial herbs	Flower	White	SU	Multicolour(Fruit shape)	AU
Asteraceae	*Anaphalis*	*Anaphalis aureopunctata*	Perennial herbs	Flower	White	SU	Multicolour(Fruit shape)	AU
Asteraceae	*Anaphalis*	*Anaphalis margaritacea*	Perennial herbs	Flower	White	SU	Multicolour(Fruit shape)	AU
Asteraceae	*Lactuca*	*Lactuca sibirica*	Perennial herbs	Flower、Fruit	Purple	SU	Multicolour(Fruit shape)	AU
Asteraceae	*Lactuca*	*Lactuca raddeana*	Annual and biennial herbs	Flower	Yellow	SP、SU		
Asteraceae	*Lactuca*	*Lactuca indica*	Annual and biennial herbs	Flower	White	SP、SU	Multicolour(Fruit shape)	SU
Asteraceae	*Ligularia*	*Ligularia dentata*	Perennial herbs	Flower	Yellow	SU、AU		
Asteraceae	*Ligularia*	*Ligularia hodgsonii*	Perennial herbs	Flower	Yellow	SU、AU		
Asteraceae	*Ligularia*	*Ligularia przewalskii*	Perennial herbs	Flower	Yellow	SU、AU		
Asteraceae	*Ligularia*	*Ligularia fischeri*	Perennial herbs	Flower	Yellow	SU		
Asteraceae	*Ligularia*	*Ligularia veitchiana*	Perennial herbs	Flower	Yellow	SU		
Asteraceae	*Ligularia*	*Ligularia hookeri*	Perennial herbs	Flower	Yellow	SU		
Asteraceae	*Ligularia*	*Ligularia dolichobotrys*	Perennial herbs	Flower	Yellow	SU	Multicolour(Fruit shape)	SU
Asteraceae	*Syneilesis*	*Syneilesis aconitifolia*	Perennial herbs	Flower、Fruit	Red	SU、AU	Multicolour(Fruit shape)	AU
Asteraceae	*Carpesium*	*Carpesium macrocephalum*	Perennial herbs	Flower	Yellow	SP		
Astera	*Gnap*	*Pseudognaphali*	Perennia	Flowe	Yello	SP	Multicol	AU

ceae	*halium*	*um affine*	l herbs	r 、Fruit	w		our(Fruit shape)	
Asteraceae	*Achillea*	*Achillea alpina*	Perennial herbs	Flower	White	SU		
Asteraceae	*Arctium*	*Arctium lappa*	Annual and biennial herbs	Flower 、Fruit	Purple	SU、AU	Multicolour(Fruit shape)	AU
Asteraceae	*Hieracium*	*Hieracium umbellatum*	Perennial herbs	Flower	Yellow	SU	Black	AU
Asteraceae	*Gynura*	*Gynura japonica*	Perennial herbs	Flower	Yellow	SU	Brown	AU
Asteraceae	*Senecio*	*Senecio scandens*	Perennial herbs	Flower	Yellow	SP、SU		
Asteraceae	*Senecio*	*Senecio nemorensis*	Perennial herbs	Flower	Yellow	SU、AU		
Asteraceae	*Taraxacum*	*Taraxacum mongolicum*	Perennial herbs	Flower 、Fruit	Yellow	SP、SU	Multicolour(Fruit shape)	SU
Asteraceae	*Sinosenecio*	*Sinosenecio oldhamianus*	Perennial herbs	Flower	Yellow	SP、SU		
Asteraceae	*Turczaninovia*	*Turczaninovia fastigiata*	Perennial herbs	Flower	Yellow	SP、SU		
Asteraceae	*Picris*	*Picris hieracioides*	Annual and biennial herbs	Flower	Yellow	SU、AU		
Asteraceae	*Myripnois*	*Myripnois dioica*	Deciduous shrub	Flower	Multicolour	SP		
Asteraceae	*Rhaponticum*	*Rhaponticum uniflorum*	Perennial herbs	Flower	Purple	SP、SU		
Asteraceae	*Echinops*	*Echinops gmelinii*	Annual herbs	Flower	Purple	SU		
Asteraceae	*Tussilago*	*Tussilago farfara*	Perennial herbs	Flower	Yellow	SP		
Asteraceae	*Ixeris*	*Ixeris polycephala*	Annual herbs	Flower	Yellow	SP	Multicolour(Fruit shape)	SU
Asteraceae	*Sonchus*	*Sonchus wightianus*	Perennial herbs	Flower	Yellow	SP、SU	Multicolour(Frui	SU

							t shape)	
Asteraceae	*Dendranthema*	*Chrysanthemum hypargyrum*	Perennial herbs	Flower	White	AU		
Asteraceae	*Dendranthema*	*Chrysanthemum zawadskii*	Perennial herbs	Flower	White	AU		
Asteraceae	*Dendranthema*	*Chrysanthemum indicum*	Perennial herbs	Flower	Yellow	AU		
Asteraceae	*Cichorium*	*Cichorium intybus*	Perennial herbs	Flower	Purple	SP、SU	Multicolour(Fruit shape)	AU
Asteraceae	*Cirsium*	*Cirsium pendulum*	Perennial herbs	Flower	Purple	SU		
Asteraceae	*Cirsium*	*Cirsium japonicum*	Perennial herbs	Flower	Purple	SP、SU	Multicolour(Fruit shape)	AU
Asteraceae	*Cirsium*	*Cirsium arvense var. integrifolium*	Perennial herbs	Flower	Purple	SP、SU	Multicolour(Fruit shape)	AU
Asteraceae	*Cirsium*	*Cirsium lineare*	Perennial herbs	Flower	Yellow	AU		
Asteraceae	*Cirsium*	*Cirsium leo*	Perennial herbs	Flower	Purple	SU		
Asteraceae	*Leontopodium*	*Leontopodium leontopodioides*	Perennial herbs	Flower	White	SU、AU		
Asteraceae	*Leontopodium*	*Leontopodium japonicum*	Perennial herbs	Flower、Fruit	White	SU、AU	Multicolour(Fruit shape)	AU
Asteraceae	*Leontopodium*	*Leontopodium smithianum*	Perennial herbs	Flower	White	SU		
Asteraceae	*Youngia*	*Youngia japonica*	Perennial herbs	Flower	Yellow	SP		
Asteraceae	*Carthamus*	*Carthamus tinctorius*	Annual herbs	Flower	Red	SP		
Asteraceae	*Artemisia*	*Artemisia sieversiana*	Annual and biennial herbs	Flower、Fruit	White	SU、AU	Multicolour(Fruit shape)	AU
Asteraceae	*Artemisia*	*Artemisia eriopoda*	Perennial herbs	Fruit			Multicolour(Frui	AU

							t shape)	
Asteraceae	*Artemisia*	*Artemisia capillaris*	Perennial herbs	Fruit			Multicolour(Fruit shape)	AU
Asteraceae	*Bidens*	*Bidens pilosa*	Annual herbs	Flower	White	SP	Black	AU
Asteraceae	*Bidens*	*Bidens parviflora*	Annual herbs	Flower、Fruit	Yellow		Multicolour(Fruit shape)	AU
Asteraceae	*Tephroseris*	*Tephroseris kirilowii*	Perennial herbs	Flower	Yellow	SP		
Asteraceae	*Tephroseris*	*Tephroseris flammea*	Perennial herbs	Flower	Yellow	SU		
Asteraceae	*Petasites*	*Petasites japonicus*	Perennial herbs	Flower、Fruit	White	SP	Multicolour(Fruit shape)	SU
Asteraceae	*Saussurea*	*Saussurea cordifolia*	Perennial herbs	Flower	Purple	SU		
Asteraceae	*Saussurea*	*Saussurea purpurascens*	Perennial herbs	Flower	Purple	SU		
Asteraceae	*Saussurea*	*Saussurea nigrescens*	Perennial herbs	Flower	Purple	AU	Multicolour(Fruit shape)	AU
Asteraceae	*Saussurea*	*Saussurea huashanensis*	Perennial herbs	Flower	Purple	AU	Multicolour(Fruit shape)	AU
Asteraceae	*Saussurea*	*Saussurea mutabilis*	Annual herbs	Flower	White	SU		
Asteraceae	*Erigeron*	*Erigeron acris*	Perennial herbs	Flower	Purple	SU		
Asteraceae	*Artemisia*	*Artemisia deversa*	Perennial herbs	Flower	White	SU、AU	Multicolour(Fruit shape)	SU、AU
Athyriaceae	*Cystopteris*	*Cystopteris moupinensis*	Annual herbs	观叶				
Balanophoraceae	*Balanophora*	*Balanophora harlandii*	Perennial herbs	Flower	Red	AU		
Balsaminaceae	*Impatiens*	*Impatiens noli-tangere*	Annual herbs	Flower	Yellow	SU、AU	Multicolour(Fruit shape)	AU
Balsaminaceae	*Impatiens*	*Impatiens stenosepala*	Annual herbs	Flower	Purple	SU		

Balsaminaceae	*Impatiens*	*Impatiens davidii*	Annual herbs	Flower	Red	SU		
Balsaminaceae	*Impatiens*	*Impatiens fissicornis*	Annual herbs	Flower	Yellow	SU		
Balsaminaceae	*Impatiens*	*Impatiens linocentra*	Annual herbs	Flower	Pink	SU	Multicolour(Fruit shape)	AU
Balsaminaceae	*Impatiens*	*Impatiens notolopha*	Annual herbs	Flower	Yellow	SU	Multicolour(Fruit shape)	SU、AU
Balsaminaceae	*Impatiens*	*Impatiens potaninii*	Annual herbs	Flower	Yellow	SU		
Balsaminaceae	*Impatiens*	*Impatiens pterosepala*	Annual herbs	Flower	Purple	SU		
Begoniaceae	*Begonia*	*Begonia grandis subsp. sinensis*	Perennial herbs	Flower	Red	AU		
Berberidaceae	*Epimedium*	*Epimedium pubescens*	Perennial herbs	Flower	White	SP	Multicolour(Fruit shape)	SU
Berberidaceae	*Epimedium*	*Epimedium brevicornu*	Perennial herbs	Flower	White	SP	Multicolour(Fruit shape)	SU
Berberidaceae	*Berberis*	*Berberis circumserrata*	Deciduous shrub	Flower	Yellow	SP	Red	SU
Berberidaceae	*Berberis*	*Berberis vernae*	Deciduous shrub	Flower	Yellow	SP	Red	SU
Berberidaceae	*Berberis*	*Berberis oritrepha*	Evergreen shrub	Flower	Yellow	SU		
Berberidaceae	*Berberis*	*Berberis diaphana*	Deciduous shrub	Flower	Yellow	SP	Red	SU
Berberidaceae	*Berberis*	*Berberis candidula*	Evergreen shrub	Flower	Yellow	SP	Multicolour(Fruit shape)	SU
Berberidaceae	*Berberis*	*Berberis dasystachya*	Deciduous shrub	Flower	Yellow	SP	Red	SU

Berberidaceae	*Berberis*	*Berberis dictyoneura*	Deciduous shrub	Flower	Yellow	SP	Red	SU
Berberidaceae	*Berberis*	*Berberis kansuensis*	Deciduous shrub	Flower	Yellow	SP	Red	SU
Berberidaceae	*Berberis*	*Berberis amurensis*	Deciduous shrub	Flower	Yellow	SP	Red	AU
Berberidaceae	*Berberis*	*Berberis poiretii*	Deciduous shrub	Flower	Yellow	SP	Red	SU
Berberidaceae	*Berberis*	*Berberis reticulata*	Deciduous shrub	Flower	Yellow	SU	Red	SU
Berberidaceae	*Berberis*	*Berberis julianae*	Evergreen shrub	Flower	Yellow	SP	Black	AU
Berberidaceae	*Berberis*	*Berberis henryan*	Deciduous shrub	Flower	Yellow	SP	Red	SU
Berberidaceae	*Berberis*	*Berberis salicaria*	Deciduous shrub	Flower	Yellow	SP	Red	SU
Berberidaceae	*Berberis*	*Berberis brachypoda*	Deciduous shrub	Flower	Yellow	SP	Red	AU
Berberidaceae	*Berberis*	*Berberis dielsiana*	Deciduous shrub	Flower	Yellow	SP	Red	SU
Berberidaceae	*Berberis*	*Berberis feddeana*	Deciduous shrub	Flower	Yellow	SP	Red	SU
Berberidaceae	*Berberis*	*Berberis gilgiana*	Deciduous shrub	Flower	Yellow	SP	Red	SU
Berberidaceae	*Berberis*	*Berberis shensiana*	Deciduous shrub	Flower	Yellow	SU	Red	SU
Berberidaceae	*Berberis*	*Berberis souliean*	Evergreen shrub	Flower	Yellow	SP	Red	SU
Berberidace	*Berberis*	*Berberis potaninii*	Evergreen shrub	Flower	Yellow	SP	Red	SU

ae								
Berberidaceae	*Berberis*	*Berberis virgetorum*	Deciduous shrub	Flower	Yellow	SP	Red	SU
Berberidaceae	*Sinopodophyllum*	*Sinopodophyllum hexandrum*	Perennial herbs	Flower	Red	SP	Multicolour(Fruit shape)	SU
Berberidaceae	*Mahonia*	*Mahonia bealei*	Evergreen shrub	Flower	Yellow	AU	Purple	SP
Berberidaceae	*Diphylleia*	*Diphylleia sinensis*	Perennial herbs	Flower、Fruit	White	SP	Multicolour(Fruit shape)	SU
Berberidaceae	*Nandina*	*Nandina domestica*	Evergreen shrub	Flower	White	SU	Red	AU
Berberidaceae	*Caulophyllum*	*Caulophyllum robustum*	Perennial herbs	Fruit	Yellow	SP、SU	Black	SU、AU
Berberidaceae	*Dysosma*	*Dysosma versipellis*	Perennial herbs	Flower、Fruit	Red	SP、SU	Multicolour(Fruit shape)	SU、AU
Berberidaceae	*Epimedium*	*Epimedium sagittatum*	Perennial herbs	Flower、Fruit	White	SP	Multicolour(Fruit shape)	SP、SU
Bignoniaceae	*Campsis*	*Campsis grandiflora*	Deciduous liana	Flower	Red	SU		
Bignoniaceae	*Incarvillea*	*Incarvillea sinensis*	Perennial herbs	Flower、Fruit	Purple	SP、SU	Multicolour(Fruit shape)	AU
Boraginaceae	*Lithospermum*	*Lithospermum zollingeri*	Perennial herbs	Flower	Purple	SU		
Boraginaceae	*Sinojohnstonia*	*Sinojohnstonia chekiangensis*	Perennial herbs	Flower	Multicolour	SP	Multicolour(Fruit shape)	SP
Boraginaceae	*Sinojohnstonia*	*Sinojohnstonia moupinensis*	Perennial herbs	Flower	Multicolour	SP、SU	Brown	SP、SU
Boraginaceae	*Ehretia*	*Ehretia dicksonii*	Deciduous tree	Flower	White	SP	Yellow	SU

Brassicaceae	*Eruca*	*Eruca vesicaria subsp. sativa*	Annual herbs	Flower	Multicolour	SP、SU	Multicolour(Fruit shape)	SU
Buxaceae	*Sarcococca*	*Sarcococca hookeriana var. digyna*	Evergreen shrub	Flower	White	WI		
Buxaceae	*Sarcococca*	*Sarcococca ruscifolia*	Evergreen shrub	Flower	White	WI	Red	AU
Buxaceae	*Populus*	*Buxus henryi*	Evergreen shrub	Flower	Green	SP		
Buxaceae	*Populus*	*Buxus bodinieri*	Evergreen shrub	Flower	Green	WI		
Buxaceae	*Populus*	*Buxus sinica*	Evergreen shrub	Flower	Yellow	SP		
Buxaceae	*Populus*	*Buxus ichangensis*	Evergreen shrub	Flower	Yellow	SP		
Buxaceae	*Pachysandra*	*Pachysandra terminalis*	Evergreen shrub	Flower	White	SP		
Buxaceae	*Pachysandra*	*Pachysandra axillaris*	Evergreen shrub	Flower、Fruit	Pink	SP	Yellow	AU
Calycanthaceae	*Chimonanthus*	*Chimonanthus praecox*	Deciduous shrub	Flower	Yellow	WI		
Calycanthaceae	*Chimonanthus*	*Chimonanthus nitens*	Evergreen shrub	Flower	Yellow	WI		
Campanulaceae	*Adenophora*	*Adenophora petiolata subsp. hunanensis*	Perennial herbs	Flower	Purple	SU		
Campanulaceae	*Adenophora*	*Adenophora capillaris*	Perennial herbs	Flower	White	SU		
Campanulaceae	*Adenophora*	*Adenophora stricta*	Perennial herbs	Flower	Purple	SU		
Campanulaceae	*Platycodon*	*Platycodon grandiflorus*	Perennial herbs	Flower、Fruit	Purple	AU	Multicolour(Fruit shape)	WI
Campanulaceae	*Campanula*	*Campanula punctata*	Perennial herbs	Flower	Multicolour	SU	Multicolour(Fruit shape)	AU
Camp	*Codo*	*Codonopsis*	Perennia	Flowe	Gree	SU	Yellow	AU

anulaceae	*nopsis*	*pilosula subsp. tangshen*	l herbs	r、Fruit	n			
Campanulaceae	*Codonopsis*	*Codonopsis pilosula*	Perennial herbs	Flower、Fruit	Green	SU	Multicolour(Fruit shape)	AU
Campanulaceae	*Adenophora*	*Adenophora himalayana subsp. alpina*	Perennial herbs	Flower	Purple	SU、AU		
Campanulaceae	*Adenophora*	*Adenophora liliifolioides*	Perennial herbs	Flower	Multicolour	SU、AU		
Campanulaceae	*Adenophora*	*Adenophora capillaris subsp. paniculata*	Perennial herbs	Flower	Multicolour	SU、AU	Brown	SU、AU
Campanulaceae	*Adenophora*	*Adenophora petiolata*	Perennial herbs	Flower	Multicolour	SU		
Campanulaceae	*Adenophora*	*Adenophora stricta subsp. sessilifolia*	Perennial herbs	Flower	Purple	SU、AU		
Campanulaceae	*Codonopsis*	*Codonopsis tsinlingensis*	Perennial herbs	Flower	Purple	SU、AU	Multicolour(Fruit shape)	SU、AU
Caprifoliaceae	*Valeriana*	*Valeriana officinalis*	Perennial herbs	Flower	Purple	SP	Multicolour(Fruit shape)	AU
Caprifoliaceae	*Valeriana*	*Valeriana jatamansi*	Perennial herbs	Flower	White	SP	Multicolour(Fruit shape)	SU
Caprifoliaceae	*Kolkwitzia*	*Kolkwitzia amabilis*	Deciduous shrub	Flower	Pink	SU		
Caprifoliaceae	*Triosteum*	*Triosteum pinnatifidum*	Perennial herbs	Flower、Fruit	Green		Black	SU
Caprifoliaceae	*Dipelta*	*Dipelta yunnanensis*	Deciduous shrub	Flower	Pink	SU	Multicolour(Fruit shape)	AU
Caprifoliaceae	*Dipelta*	*Dipelta floribunda*	Deciduous shrub	Flower	Pink	SP	Multicolour(Fruit shape)	AU
Caprifoliaceae	*Lonicera*	*Lonicera caerulea*	Deciduous shrub	Flower、Fruit	Yellow	SU	Black	AU

Caprifoliaceae	*Lonicera*	*Lonicera hispida*	Deciduous shrub	Flower 、Fruit	White	SU	Red	SU
Caprifoliaceae	*Lonicera*	*Lonicera nervosa*	Deciduous shrub	Flower 、Fruit	Yellow	SU	Black	AU
Caprifoliaceae	*Lonicera*	*Lonicera stephanocarpa*	Deciduous shrub	Flower 、Fruit	White	SU	Black	AU
Caprifoliaceae	*Lonicera*	*Lonicera fargesii*	Deciduous shrub	Flower 、Fruit	Red	SU	Red	AU
Caprifoliaceae	*Lonicera*	*Lonicera retusa*	Deciduous shrub	Flower 、Fruit	Yellow	SU	Black	AU
Caprifoliaceae	*Lonicera*	*Lonicera webbiana*	Deciduous shrub	Flower 、Fruit	Red	SU	Black	AU
Caprifoliaceae	*Lonicera*	*Lonicera microphylla*	Deciduous shrub	Flower 、Fruit	Yellow	SU	Red	SU
Caprifoliaceae	*Lonicera*	*Lonicera trichosantha*	Deciduous shrub	Flower 、Fruit	Yellow	SU	Red	SU
Caprifoliaceae	*Lonicera*	*Lonicera tangutica*	Deciduous shrub	Flower 、Fruit	White	SU	Red	SU
Caprifoliaceae	*Lonicera*	*Lonicera elisae*	Deciduous shrub	Flower 、Fruit	White	SP	Red	SU
Caprifoliaceae	*Lonicera*	*Lonicera modesta*	Deciduous shrub	Flower 、Fruit	Yellow	SP	Red	AU
Caprifoliaceae	*Lonicera*	*Lonicera chrysantha var. koehneana*	Deciduous shrub	Flower 、Fruit	Yellow	SU	Red	SU
Caprifoliaceae	*Lonicera*	*Lonicera ferdinandi*	Deciduous shrub	Flower 、Fruit	Yellow	SU	Red	AU
Caprifoliaceae	*Lonicera*	*Lonicera gynochlamydea*	Deciduous shrub	Flower 、Fruit	White	SP	Red	AU
Capriffoliace	*Lonicera*	*Lonicera chrysantha*	Deciduous shrub	Flower 、	Yellow	SU	Red	SU

ae				Fruit				
Caprifoliaceae	*Lonicera*	*Lonicera acuminata*	Deciduous liana	Flower、Fruit	Yellow	SU	Black	AU
Caprifoliaceae	*Lonicera*	*Lonicera similis*	Deciduous liana	Flower、Fruit	Yellow	SU	Black	AU
Caprifoliaceae	*Lonicera*	*Lonicera tragophylla*	Deciduous liana	Flower、Fruit	Yellow	SU	Red	AU
Caprifoliaceae	*Lonicera*	*Lonicera fragrantissima*	Deciduous shrub	Flower、Fruit	White	SP	Red	SP
Caprifoliaceae	*Lonicera*	*Lonicera ligustrina var. yunnanensis*	Evergreen shrub	Flower、Fruit	Yellow	SP	Black	AU
Caprifoliaceae	*Lonicera*	*Lonicera japonica*	Evergreen liana	Flower、Fruit	White	SU	Black	AU
Caprifoliaceae	*Lonicera*	*Lonicera maackii*	Deciduous shrub	Flower、Fruit	Yellow	SU	Red	AU
Caprifoliaceae	*Abelia*	*Abelia macrotera*	Deciduous shrub	Flower	Purple	SU		
Caprifoliaceae	*Abelia*	*Abelia uniflora*	Deciduous shrub	Flower	Red	SU		
Caprifoliaceae	*Symphoricarpos*	*Symphoricarpos sinensis*	Deciduous shrub	Flower	White	SU	Black	AU
Caprifoliaceae	*Zabelia*	*Zabelia dielsii*	Deciduous shrub	Flower	White	SU		
Caprifoliaceae	*Weigela*	*Weigela florida*	Deciduous shrub	Flower	Red	SP		
Caprifoliaceae	*Sambucus*	*Sambucus williamsii*	Deciduous tree	Flower	White	SP	Red	AU
Caprifoliaceae	*Sambucus*	*Sambucus javanica*	Perennial herbs	Flower、Fruit	White	SP	Red	SU
Caprif	*Samb*	*Sambucus*	Perennia	Flowe	Whit	SP、SU	Red	AU

oliaceae	*ucus*	*adnata*	l herbs	r 、Fruit	e			
Caprifoliaceae	*Viburnum*	*Viburnum kansuense*	Deciduous shrub	Flower	Red	SU	Red	AU
Caprifoliaceae	*Viburnum*	*Viburnum chinshanense*	Evergreen shrub	Flower	White	SP	Black	SU
Caprifoliaceae	*Viburnum*	*Viburnum opulus subsp. calvescens*	Deciduous shrub	Flower	White	SU	Red	AU
Caprifoliaceae	*Viburnum*	*Viburnum sympodiale*	Deciduous shrub	Flower	White	SP	Black	AU
Caprifoliaceae	*Viburnum*	*Viburnum glomeratum*	Deciduous shrub	Flower	White	SP	Black	SU
Caprifoliaceae	*Viburnum*	*Viburnum plicatum var. tomentosum*	Deciduous shrub	Flower	White	SP	Black	AU
Caprifoliaceae	*Viburnum*	*Viburnum erubescens*	Deciduous shrub	Flower	White	SP	Black	SU
Caprifoliaceae	*Viburnum*	*Viburnum foetidum var. rectangulatum*	Deciduous shrub	Flower	White	SU	Red	AU
Caprifoliaceae	*Viburnum*	*Viburnum mongolicum*	Deciduous shrub	Flower	Yellow	SP	Black	AU
Caprifoliaceae	*Viburnum*	*Viburnum rhytidophyllum*	Evergreen shrub	Flower	White	SP	Black	AU
Caprifoliaceae	*Viburnum*	*Viburnum oliganthum*	Evergreen shrub	Flower	White	SP	Black	SU
Caprifoliaceae	*Viburnum*	*Viburnum buddleifolium*	Deciduous shrub	Flower	White	SP		
Caprifoliaceae	*Viburnum*	*Viburnum propinquum*	Evergreen shrub	Flower	White	SP	Black	AU
Caprifoliaceae	*Viburnum*	*Viburnum dilatatum*	Deciduous shrub	Flower	White	SU	Red	AU

Caprifoliaceae	*Viburnum*	*Viburnum cylindricum*	Evergreen shrub	Flower	White	SU	Black	AU
Caprifoliaceae	*Viburnum*	*Viburnum schensianum*	Deciduous shrub	Flower	White	SU	Black	AU
Caprifoliaceae	*Viburnum*	*Viburnum erosum*	Deciduous shrub	Flower	White	SP	Red	AU
Caprifoliaceae	*Viburnum*	*Viburnum setigerum*	Deciduous shrub	Flower	White	SP	Red	AU
Caprifoliaceae	*Viburnum*	*Viburnum utile*	Evergreen shrub	Flower	White	SP	Black	SU
Caprifoliaceae	*Dipsacus*	*Dipsacus japonicus*	Perennial herbs	Flower	Purple	SU	Multicolour(Fruit shape)	AU
Caprifoliaceae	*Patrinia*	*Patrinia scabra*	Perennial herbs	Flower	White	SP	Multicolour(Fruit shape)	SU
Caprifoliaceae	*Patrinia*	*Patrinia rupestris*	Perennial herbs	Flower	Yellow	SU	Multicolour(Fruit shape)	SU
Caryophyllaceae	*Silene*	*Silene tatarinowii*	Perennial herbs	Flower	White	SU	Multicolour(Fruit shape)	AU
Caryophyllaceae	*Silene*	*Silene fortunei*	Perennial herbs	Flower	Red	SU	Multicolour(Fruit shape)	AU
Caryophyllaceae	*Silene*	*Silene aprica*	Perennial herbs	Flower	Multicolour	SU		
Caryophyllaceae	*Arenaria*	*Arenaria serpyllifolia*	Annual and biennial herbs	Flower、Fruit	White	SU	Brown	AU
Caryophyllaceae	*Silene*	*Silene conoidea*	Annual herbs	Flower	Pink	SP、SU	Multicolour(Fruit shape)	SU
Caryophyllaceae	*Silene*	*Silene firma*	Annual and biennial herbs	Flower	White	SU	Multicolour(Fruit shape)	SU

Caryophyllaceae	*Dianthus*	*Dianthus superbus*	Perennial herbs	Flower	Multicolour	SU	Multicolour(Fruit shape)	AU
Caryophyllaceae	*Dianthus*	*Dianthus chinensis*	Perennial herbs	Flower	Multicolour	SP	Multicolour(Fruit shape)	SU
Caryophyllaceae	*Silene*	*Silene hupehensis var. pubescens*	Perennial herbs	Flower、Fruit	Pink	SU	Multicolour(Fruit shape)	SU
Caryophyllaceae	*Silene*	*Silene pterosperma*	Perennial herbs	Flower	Yellow	SU	Multicolour(Fruit shape)	SU
Caryophyllaceae	*Silene*	*Silene repens*	Perennial herbs	Flower	Multicolour	SU	Multicolour(Fruit shape)	SU、AU
Caryophyllaceae	*Vaccaria*	*Vaccaria hispanica*	Annual herbs	Flower	Red	SP	Multicolour(Fruit shape)	AU
Caryophyllaceae	*Lychnis*	*Lychnis senno*	Perennial herbs	Flower	Red	SU	Multicolour(Fruit shape)	AU
Caryophyllaceae	*Lychnis*	*Lychnis fulgens*	Perennial herbs	Flower	Pink	SU		
Caryophyllaceae	*Arenaria*	*Arenaria giraldii*	Annual herbs	Flower	White	SU	Multicolour(Fruit shape)	SU
Caryophyllaceae	*Arenaria*	*Arenaria fimbriata*	Annual herbs	Flower	White	SU	Multicolour(Fruit shape)	SU、AU
Caryophyllaceae	*Arenaria*	*Arenaria quadridentata*	Perennial herbs	Flower、Fruit	White	SU、AU	Multicolour(Fruit shape)	SU、AU
Caryophyllaceae	*Arenaria*	*Arenaria taibaishanensis*	Perennial herbs	Flower、Fruit	White	SU		
Caryophyllaceae	*Cerastium*	*Cerastium arvense*	Perennial herbs	Flower	White	SP、SU	Multicolour(Fruit shape)	SU、AU
Caryophyllaceae	*Cerastium*	*Cerastium fontanum subsp. vulgare*	Perennial herbs	Flower	White	SP、SU	Multicolour(Fruit shape)	SU
Caryophylla	*Cerastium*	*Cerastium furcatum*	Perennial herbs	Flower	White	SP、SU	Multicolour(Frui	SU、AU

ceae							t shape)	
Caryophyllaceae	*Gypsophila*	*Gypsophila huashanensis*	Perennial herbs	Flower	Multicolour	SU	Multicolour(Fruit shape)	SU、AU
Caryophyllaceae	*Gypsophila*	*Gypsophila oldhamiana*	Perennial herbs	Flower	Pink	SU、AU	Multicolour(Fruit shape)	SU、AU
Caryophyllaceae	*Myosoton*	*Myosoton aquaticum*	Annual and biennial herbs	Flower	White	SP、SU	Multicolour(Fruit shape)	SU、AU
Caryophyllaceae	*Pseudostellaria*	*Pseudostellaria davidii*	Perennial herbs	Flower	White	SP、SU	Multicolour(Fruit shape)	SU
Caryophyllaceae	*Pseudostellaria*	*Pseudostellaria heterophylla*	Perennial herbs	Flower	White	SP、SU	Multicolour(Fruit shape)	SU
Caryophyllaceae	*Pseudostellaria*	*Pseudostellaria heterantha*	Perennial herbs	Flower	White	SP、SU	Multicolour(Fruit shape)	SU
Celastraceae	*Euonymus*	*Euonymus frigidus*	Deciduous shrub	Flower	Green	SU	Red	AU
Celastraceae	*Euonymus*	*Euonymus nanus*	Deciduous shrub	Flower	Green	SU	Red	AU
Celastraceae	*Euonymus*	*Euonymus phellomanus*	Deciduous shrub	Flower	Green	SU	Red	AU
Celastraceae	*Euonymus*	*Euonymus semenovii*	Deciduous shrub	Flower	Red	SU	Red	AU
Celastraceae	*Euonymus*	*Euonymus verrucosus*	Deciduous shrub	Flower	Red	SU	Red	AU
Celastraceae	*Euonymus*	*Euonymus actinocarpus*	Deciduous shrub	Flower	Green	WI	Red	SU
Celastraceae	*Euonymus*	*Euonymus cornutus*	Deciduous shrub	Flower	Red	SP	Red	AU
Celastraceae	*Euonymus*	*Euonymus giraldii*	Deciduous shrub	Flower	Green	SU	Red	AU
Celastraceae	*Euonymus*	*Euonymus verrucosoides*	Deciduous shrub	Flower	Green	SU	Red	AU
Celastraceae	*Euonymus*	*Euonymus schensianus*	Deciduous shrub	Flower	Green	SP	Red	AU
Celastraceae	*Euonymus*	*Euonymus carnosus*	Evergreen shrub	Flower	Yellow	SU	Red	AU
Celastraceae	*Euonymus*	*Euonymus venosus*	Deciduous shrub	Flower	Yellow	SU	Red	AU

Celastraceae	*Euonymus*	*Euonymus hamiltonianus*	Deciduous tree	Flower	Green	SU	Red	AU
Celastraceae	*Euonymus*	*Euonymus sanguineus*	Deciduous shrub	Flower	Green	SU	Red	AU
Celastraceae	*Euonymus*	*Euonymus acanthocarpus*	Deciduous shrub	Flower	Green	SU	Red	AU
Celastraceae	*Euonymus*	*Euonymus maackii*	Deciduous tree	Flower	White	SP	Red	AU
Celastraceae	*Euonymus*	*Euonymus echinatus*	Evergreen shrub	Flower	Green	SP	Red	AU
Celastraceae	*Euonymus*	*Euonymus myrianthus*	Evergreen shrub	Flower	Green	SU	Red	AU
Celastraceae	*Euonymus*	*Euonymus nitidus*	Evergreen shrub	Flower	Green	SP	Red	SU
Celastraceae	*Euonymus*	*Euonymus grandiflorus*	Deciduous shrub	Flower	Yellow	SU	Red	AU
Celastraceae	*Euonymus*	*Euonymus alatus*	Deciduous shrub	Flower	Green	SU	Red	AU
Celastraceae	*Euonymus*	*Euonymus fortunei*	Evergreen liana	Flower	Green	SP	Red	AU
Celastraceae	*Euonymus*	*Euonymus microcarpus*	Deciduous shrub	Flower	Green	SP	Red	AU
Celastraceae	*Celastrus*	*Celastrus glaucophyllus*	Deciduous liana	Flower	Green	SP	Red	AU
Celastraceae	*Celastrus*	*Celastrus gemmatus*	Deciduous liana	Flower	Green	SU	Red	AU
Celastraceae	*Celastrus*	*Celastrus hypoleucus*	Deciduous liana	Flower	Green	SP	Red	AU
Celastraceae	*Celastrus*	*Celastrus orbiculatus*	Deciduous liana	Flower	Green	SU	Red	AU
Celastraceae	*Celastrus*	*Celastrus rosthornianus*	Deciduous liana	Flower	Green	SP	Red	AU
Celastraceae	*Celastrus*	*Celastrus rosthornianus var. loeseneri*	Deciduous liana	Flower	Green	SP	Red	AU
Celastraceae	*Celastrus*	*Celastrus angulatus*	Deciduous shrub	Flower	Green	SU	Yellow	AU
Celastraceae	*Parnassia*	*Parnassia brevistyla*	Perennial herbs	Flower、Fruit	White	SU	Multicolour(Fruit shape)	AU
Celastraceae	*Parnassia*	*Parnassia delavayi*	Perennial herbs	Flower、Fruit	White	SU	Multicolour(Fruit shape)	AU
Celastraceae	*Parnassia*	*Parnassia*	Perennia	Flowe	Whit	SU	Multicol	AU

raceae	*ssia*	*wightiana*	l herbs	r、Fruit	e		our(Fruit shape)	
Ceratophyllaceae	*Ceratophyllum*	*Ceratophyllum demersum*	Perennial herbs	Fruit			Multicolour(Fruit shape)	SU
Ceratophyllaceae	*Ceratophyllum*	*Ceratophyllum demersum*	Perennial herbs	Flower、Fruit		SU	Multicolour(Fruit shape)	SU、AU
Chenopodiaceae	*Chenopodium*	*Dysphania schraderiana*	Annual herbs	Flower、Fruit		SU、AU	Multicolour(Fruit shape)	AU
Chenopodiaceae	*Salsola*	*Salsola collina*	Annual herbs	Fruit		SU、AU	Multicolour(Fruit shape)	AU
Chloranthaceae	*Chloranthus*	*Chloranthus henryi var. hupehensis*	Perennial herbs	Flower、Fruit		SP、SU		
Chloranthaceae	*Chloranthus*	*Chloranthus multistachys*	Perennial herbs	Flower	White	SU	Multicolour(Fruit shape)	AU
Colchicaceae	*Disporum*	*Disporum cantoniense*	Perennial herbs	Flower	Purple	SP	Multicolour(Fruit shape)	SU
Commelinaceae	*Streptolirion*	*Streptolirion volubile*	Perennial herbs	Flower、Fruit	White	SU	Multicolour(Fruit shape)	AU
Commelinaceae	*Commelina*	*Commelina benghalensis*	Perennial herbs	Flower	Purple			
Commelinaceae	*Commelina*	*Commelina communis*	Annual herbs	Flower	Purple	SP		
Commelinaceae	*Murdannia*	*Murdannia triquetra*	Perennial herbs	Fruit			Multicolour(Fruit shape)	AU
Compositae	*Senecio*	*Senecio argunensis*	Perennial herbs	Flower	Yellow	SU		
Compositae	*Senecio*	*Senecio scandens var. incisus*	Perennial herbs	Flower	Yellow	AU、WI		
Compositae	*Sinacalia*	*Sinacalia tangutica*	Perennial herbs	Flower	Yellow	SU、AU		
Compositae	*Sinoseneci*	*Sinosenecio euosmus*	Annual herbs	Flower	Yellow	SU		

	o							
Compositae	*Ajania*	*Ajania potaninii*	Deciduous shrub	Flower	Yellow	SU	Multicolour(Fruit shape)	AU
Compositae	*Artemisia*	*Artemisia qinlingensis*	Perennial herbs	Flower、Fruit	Red	SU、AU	Multicolour(Fruit shape)	SU、AU
Compositae	*Doronicum*	*Doronicum gansuense*	Perennial herbs	Flower	Yellow	SU		
Compositae	*Ixeridium*	*Ixeridium gracile*	Perennial herbs	Flower	Yellow	SP、SU	Brown	SU、AU
Compositae	*Leontopodium*	*Leontopodium giraldii*	Perennial herbs	Flower	Purple	SU、AU		
Compositae	*Ligularia*	*Ligularia achyrotricha*	Perennial herbs	Flower	Yellow	SU	Multicolour(Fruit shape)	SU
Compositae	*Ligulariopsis*	*Ligulariopsis shichuana*	Perennial herbs	Flower	Yellow	SU	Brown	SU、AU
Compositae	*Parasenecio*	*Parasenecio ambiguus*	Perennial herbs	Flower	White	SU	Multicolour(Fruit shape)	AU
Compositae	*Parasenecio*	*Parasenecio dasythyrsus*	Perennial herbs	Flower	Yellow	SU、AU	Multicolour(Fruit shape)	SU、AU
Compositae	*Parasenecio*	*Parasenecio gansuensis*	Perennial herbs	Flower	Yellow	SU	Multicolour(Fruit shape)	AU
Compositae	*Parasenecio*	*Parasenecio pilgerianus*	Perennial herbs	Flower	Multicolour	SU	Multicolour(Fruit shape)	AU
Compositae	*Parasenecio*	*Parasenecio roborowskii*	Perennial herbs	Flower	White	SU	Multicolour(Fruit shape)	AU
Compositae	*Parasenecio*	*Parasenecio sinicus*	Perennial herbs	Flower	Multicolour	SU	Brown	AU
Compositae	*Parasenecio*	*Parasenecio tsinlingensis*	Perennial herbs	Flower	White	SU	Brown	AU
Compositae	*Petasites*	*Petasites tricholobus*	Perennial herbs	Flower、Fruit	White			

Compositae	*Picris*	*Picris japonica*	Perennial herbs	Flower	Yellow	SU、AU	Brown	SU、AU
Compositae	*Prenanthes*	*Nabalus tatarinowii*	Perennial herbs	Flower	Multicolour	SU、AU	Brown	SU、AU
Compositae	*Saussurea*	*Saussurea acromelaena*	Perennial herbs	Flower	Purple	AU	Brown	AU
Compositae	*Saussurea*	*Saussurea acrophila*	Perennial herbs	Flower	Purple	SU、AU	Brown	SU、AU
Compositae	*Saussurea*	*Saussurea acroura*	Perennial herbs	Flower	Pink	SU	Brown	SU
Compositae	*Saussurea*	*Saussurea dolichopoda*	Perennial herbs	Flower	Purple	SU、AU	Brown	SU、AU
Compositae	*Saussurea*	*Saussurea flaccida*	Perennial herbs	Flower	Yellow	SU、AU	Multicolour(Fruit shape)	SU、AU
Compositae	*Saussurea*	*Saussurea iodostegia*	Perennial herbs	Flower	Purple	SU、AU	Brown	SU、AU
Compositae	*Saussurea*	*Saussurea kungii*	Perennial herbs	Flower	Purple	AU	Multicolour(Fruit shape)	AU
Compositae	*Saussurea*	*Saussurea licentiana*	Perennial herbs	Flower	Purple	SU、AU	Brown	SU、AU
Compositae	*Saussurea*	*Saussurea macrota*	Perennial herbs	Flower	Purple	SU	Brown	SU
Compositae	*Saussurea*	*Saussurea morifolia*	Perennial herbs	Flower	Purple	SU、AU	Brown	SU、AU
Compositae	*Saussurea*	*Saussurea oligantha*	Perennial herbs	Flower	Purple	SU、AU	Multicolour(Fruit shape)	SU、AU
Compositae	*Saussurea*	*Saussurea oligocephala*	Perennial herbs	Flower	Purple	AU	Multicolour(Fruit shape)	AU
Compositae	*Saussurea*	*Saussurea paucijuga*	Perennial herbs	Flower	Purple	SU、AU	Multicolour(Fruit shape)	SU、AU
Compositae	*Saussurea*	*Saussurea pectinata*	Perennial herbs	Flower	Purple	SU、AU	Multicolour(Fruit shape)	SU、AU
Compositae	*Saussurea*	*Saussurea populifolia*	Perennial herbs	Flower	Purple	SU、AU	Brown	SU、AU
Compositae	*Saussurea*	*Saussurea saligna*	Perennial herbs	Flower	Purple	SU、AU	Multicolour(Fruit shape)	SU、AU

Compositae	*Saussurea*	*Saussurea sobarocephala*	Perennial herbs	Flower	Purple	SU	Multicolour(Fruit shape)	SU
Compositae	*Saussurea*	*Saussurea megaphylla*	Perennial herbs	Flower	Purple	SU、AU	Multicolour(Fruit shape)	SU、AU
Compositae	*Scorzonera*	*Scorzonera albicaulis*	Perennial herbs	Flower、Fruit	Yellow	SP、SU	Multicolour(Fruit shape)	SU、AU
Convolvulaceae	*Merremia*	*Merremia sibirica*	Annual herbs	Flower	Red	SU	Multicolour(Fruit shape)	AU
Convolvulaceae	*Convolvulus*	*Convolvulus arvensis*	Perennial herbs	Flower	Multicolour	SU		
Convolvulaceae	*Pharbitis*	*Ipomoea purpurea*	Annual herbs	Flower	Multicolour	SP、SU	Multicolour(Fruit shape)	AU
Convolvulaceae	*Pharbitis*	*Ipomoea nil*	Annual herbs	Flower	Multicolour	SU		
Convolvulaceae	*Calystegia*	*Calystegia hederacea*	Annual herbs	Flower	Purple	SU		
Convolvulaceae	*Calystegia*	*Calystegia sepium*	Perennial herbs	Flower、Fruit	White	SU		
Coriariaceae	*Coriaria*	*Coriaria nepalensis*	Deciduous shrub	Flower	Red	SP	Black	SU
Cornaceae	*Dendrobenthamia*	*Cornus elliptica*	Evergreen tree	Flower	White	SU	Red	AU
Cornaceae	*Swida*	*Cornus schindleri subsp. poliophylla*	Deciduous tree	Flower	White	SU	Black	AU
Cornaceae	*Swida*	*Cornus bretschneideri*	Deciduous tree	Flower	White	SU	Black	AU
Cornaceae	*Cornus*	*Cornus hemsleyi*	Deciduous shrub	Flower	White	SU	Black	AU
Cornaceae	*Cornus*	*Cornus chinensis*	Deciduous tree	Flower	Yellow	SP	Black	AU
Cornaceae	*Swida*	*Cornus bretschneideri*	Deciduous tree	Flower	White	SU	Black	AU

		var. crispa						
Cornaceae	*Cornus*	*Cornus officinalis*	Deciduous tree	Flower	Yellow	SP	Red	AU
Cornaceae	*Swida*	*Cornus ulotricha*	Deciduous tree	Flower	White	SU	Black	SU
Cornaceae	*Dendrobenthamia*	*Cornus kousa subsp. chinensis*	Deciduous tree	Flower	White	SU	Red	AU
Cornaceae	*Bothrocaryum*	*Cornus controversa*	Deciduous tree	Flower	White	SU	Black	SU
Cornaceae	*Swida*	*Cornus quinquenervis*	Deciduous shrub	Flower	White	SU	Black	AU
Cornaceae	*Swida*	*Cornus walteri*	Deciduous tree	Flower	White	SP	Black	AU
Cornaceae	*Swida*	*Cornus macrophylla*	Deciduous tree	Flower	White	SU	Black	AU
Cornaceae	*Swida*	*Cornus wilsoniana*	Deciduous tree	Flower	White	SP	Black	AU
Cornaceae	*Swida*	*Cornus alba*	Deciduous shrub	Flower	White	SU	Multicolour(Fruit shape)	SU
Cornaceae	*Alangium*	*Alangium chinense subsp. pauciflorum*	Deciduous tree	Flower、Fruit	Yellow	SU	Black	SU
Cornaceae	*Alangium*	*Alangium platanifolium var. trilobum*	Deciduous shrub	Flower、Fruit	Yellow	SP	Purple	SU
Cornaceae	*Alangium*	*Alangium chinense*	Deciduous tree	Flower、Fruit	Yellow	SU	Black	SU
Crassulaceae	*Orostachys*	*Orostachys fimbriata*	Annual and biennial herbs	Flower、Fruit	Red	SU	Multicolour(Fruit shape)	AU
Crassulaceae	*Sinocrassula*	*Sinocrassula indica*	Annual and biennial herbs	Flower	Red			
Crassulaceae	*Sinocrassula*	*Sinocrassula indica var. viridiflora*	Annual herbs	Flower、Fruit	Yellow	AU	Multicolour(Fruit shape)	AU
Crassu	*Sedu*	*Sedum*	Perennia	Flowe	Yello	SP	Multicol	SU

laceae	*m*	*sarmentosum*	l herbs	r、Fruit	w		our(Fruit shape)	
Crassulaceae	*Sedum*	*Sedum lineare*	Perennial herbs	Flower、Fruit	Yellow	SP	Multicolour(Fruit shape)	SU
Crassulaceae	*Sedum*	*Sedum elatinoides*	Annual herbs	Flower	White	SP、SU	Multicolour(Fruit shape)	SU、AU
Crassulaceae	*Sedum*	*Rhodiola kirilowii*	Perennial herbs	Flower	Yellow	SU	Multicolour(Fruit shape)	AU
Crassulaceae	*Sedum*	*Sedum majus*	Perennial herbs	Flower	White	AU		
Crassulaceae	*Sedum*	*Rhodiola dumulosa*	Perennial herbs	Flower、Fruit	Multicolour	SU	Multicolour(Fruit shape)	AU
Crassulaceae	*Sedum*	*Phedimus aizoon*	Perennial herbs	Flower、Fruit	Yellow	SU	Multicolour(Fruit shape)	AU
Crassulaceae	*Hylotelephium*	*Hylotelephium verticillatum*	Perennial herbs	Flower、Fruit	Green	SU	Multicolour(Fruit shape)	AU
Crassulaceae	*Hylotelephium*	*Hylotelephium spectabile*	Perennial herbs	Flower、Fruit	Purple	SU	Multicolour(Fruit shape)	SU、AU
Crassulaceae	*Hylotelephium*	*Hylotelephium angustum*	Perennial herbs	Flower	Pink	SU		
Crassulaceae	*Rhodiola*	*Rhodiola macrocarpa*	Perennial herbs	Flower	Multicolour	SU、AU	Multicolour(Fruit shape)	SU、AU
Crassulaceae	*Rhodiola*	*Rhodiola yunnanensis*	Perennial herbs	Flower	Red	SP、SU	Multicolour(Fruit shape)	SU
Crassulaceae	*Phedimus*	*Phedimus aizoon var. scabrus*	Perennial herbs	Flower	Yellow	SU	Multicolour(Fruit shape)	SU
Crassulaceae	*Sedum*	*Sedum oligospermum*	Annual herbs	Flower	Yellow	SU、AU	Multicolour(Fruit shape)	SU、AU
Crassulaceae	*Sedum*	*Sedum dielsii*	Perennial herbs	Flower	Yellow	AU	Multicolour(Fruit shape)	AU
Crassu	*Sedu*	*Sedum filipes*	Annual	Flowe	Purpl	SU、AU	Multicol	AU

laceae	*m*		and biennial herbs	r	e		our(Fruit shape)	
Crassulaceae	*Sedum*	*Sedum pampaninii*	Perennial herbs	Flower	Yellow	SU、AU	Multicolour(Fruit shape)	AU
Crassulaceae	*Sedum*	*Sedum planifolium*	Perennial herbs	Flower	Yellow	SU	Multicolour(Fruit shape)	SU
Crassulaceae	*Sedum*	*Sedum stellariifolium*	Annual and biennial herbs	Flower	Yellow	SU	Multicolour(Fruit shape)	SU、AU
Cruciferae	*Orychophragmus*	*Orychophragmus violaceus*	Annual and biennial herbs	Flower、Fruit	Purple	SP	Multicolour(Fruit shape)	SU
Cruciferae	*Eruca*	*Eruca vesicaria subsp. sativa*	Annual herbs	Flower、Fruit	Yellow	SP	Multicolour(Fruit shape)	SU
Cruciferae	*Erysimum*	*Erysimum amurense*	Annual and biennial herbs	Flower	Yellow	SU	Multicolour(Fruit shape)	AU
Cruciferae	*Cardamine*	*Cardamine macrophylla*	Perennial herbs	Flower	Multicolour	SP、SU	Multicolour(Fruit shape)	AU
Cruciferae	*Cardamine*	*Cardamine leucantha*	Perennial herbs	Flower	White	SP	Multicolour(Fruit shape)	SU
Cruciferae	*Isatis*	*Isatis tinctoria*	Annual and biennial herbs	Flower、Fruit	Yellow	SP	Multicolour(Fruit shape)	SU
Cruciferae	*Rorippa*	*Rorippa indica*	Annual and biennial herbs	Flower、Fruit	Yellow	SU	Brown	AU
Cruciferae	*Arabidopsis*	*Arabidopsis thaliana*	Annual herbs	Flower、Fruit	White	SP、SU		
Cruciferae	*Arabis*	*Arabis paniculata*	Annual and biennial	Flower、Fruit	White	SP、SU	Multicolour(Fruit shape)	SU、AU

			herbs					
Cruciferae	*Arabis*	*Arabis hirsuta*	Annual and biennial herbs	Flower、Fruit	White	SP、SU	Brown	SU
Cruciferae	*Arabis*	*Arabis pendula*	Annual and biennial herbs	Flower、Fruit	White	SU、AU	Brown	SU、AU
Cruciferae	*Brassica*	*Brassica rapa var. oleifera*	Annual and biennial herbs	Flower	Yellow	SP	Multicolour(Fruit shape)	SP
Cruciferae	*Brassica*	*Brassica juncea*	Annual herbs	Flower	Yellow	SP	Multicolour(Fruit shape)	SP、SU
Cruciferae	*Capsella*	*Capsella bursa-pastoris*	Annual and biennial herbs	Flower	White	SP、SU	Multicolour(Fruit shape)	SP、SU
Cruciferae	*Cardamine*	*Cardamine engleriana*	Perennial herbs	Flower	White	SP	Multicolour(Fruit shape)	SU
Cruciferae	*Cardamine*	*Cardamine flexuosa*	Annual and biennial herbs	Flower	White	SP	Multicolour(Fruit shape)	SP、SU
Cruciferae	*Cardamine*	*Cardamine hirsuta*	Annual herbs	Flower	White	SP	Multicolour(Fruit shape)	SP、SU
Cruciferae	*Cardamine*	*Cardamine impatiens*	Annual and biennial herbs	Flower	White	SP、SU	Multicolour(Fruit shape)	SP、SU
Cruciferae	*Cardamine*	*Cardamine scaposa*	Perennial herbs	Flower、Fruit	White	SP、SU	Multicolour(Fruit shape)	SU
Cruciferae	*Chorispora*	*Chorispora tenella*	Annual herbs	Flower	Purple	SP、SU	Multicolour(Fruit shape)	SP、SU
Cruciferae	*Descurainia*	*Descurainia sophia*	Annual herbs	Flower	Yellow	SP		
Cruciferae	*Dontostemo*	*Dontostemon dentatus*	Annual and	Flower	Purple	SP、SU	Multicolour(Frui	SU

	n		biennial herbs				t shape)	
Cruciferae	*Draba*	*Draba ladyginii*	Perennial herbs	Flower	White	SP、SU	Multicolour(Fruit shape)	SU
Cruciferae	*Draba*	*Draba lasiophylla*	Perennial herbs	Flower	White	SU	Multicolour(Fruit shape)	SU
Cruciferae	*Draba*	*Draba nemorosa*	Annual and biennial herbs	Flower	Yellow	SP	Multicolour(Fruit shape)	SP、SU
Cruciferae	*Draba*	*Draba oreades*	Perennial herbs	Flower	Yellow	SU		
Cruciferae	*Erysimum*	*Erysimum cheiranthoides*	Annual herbs	Flower	Yellow	SP	Multicolour(Fruit shape)	SU
Cruciferae	*Eutrema*	*Eutrema heterophyllum*	Perennial herbs	Flower	White	SU	Multicolour(Fruit shape)	AU
Cruciferae	*Eutrema*	*Eutrema yunnanense*	Perennial herbs	Flower、Fruit	White	SP	Multicolour(Fruit shape)	SP、SU
Cruciferae	*Lepidium*	*Lepidium apetalum*	Annual and biennial herbs	Flower、Fruit		SP、SU	Multicolour(Fruit shape)	SP、SU
Cruciferae	*Lepidium*	*Lepidium cuneiforme*	Annual and biennial herbs	Flower	White	SP、SU	Multicolour(Fruit shape)	SP、SU
Cruciferae	*Malcolmia*	*Malcolmia africana*	Annual and biennial herbs	Flower	Purple	SU	Multicolour(Fruit shape)	SU
Cruciferae	*Nasturtium*	*Nasturtium officinale*	Perennial herbs	Flower、Fruit	White	SP	Multicolour(Fruit shape)	SU
Cruciferae	*Raphanus*	*Raphanus sativus*	Annual and biennial herbs	Flower	Multicolour	SP	Multicolour(Fruit shape)	SP、SU
Cruciferae	*Rorippa*	*Rorippa cantoniensis*	Annual and	Flower	Yellow	SP	Multicolour(Frui	SP、SU

			biennial herbs				t shape)	
Cruciferae	*Rorippa*	*Rorippa dubia*	Annual herbs	Flower、Fruit		SP、SU	Multicolour(Fruit shape)	SU
Cucurbitaceae	*Gynostemma*	*Gynostemma pentaphyllum*	Perennial herbs	Fruit			Black	SU
Cucurbitaceae	*Bolbostemma*	*Bolbostemma paniculatum*	Perennial herbs	Fruit			Multicolour(Fruit shape)	SU
Cyperaceae	*Carex*	*Carex neurocarpa*	Perennial herbs	Flower	Green			
Cyperaceae	*Cyperus*	*Cyperus difformis*	Annual herbs	观叶				
Cyperaceae	*Cyperus*	*Cyperus microiria*	Annual herbs	观叶				
Cyperaceae	*Cyperus*	*Cyperus iria*	Annual herbs	观叶				
Cyperaceae	*Carex*	*Carex agglomerata*	Perennial herbs	Flower、Fruit		SP、SU	Yellow	SP、SU
Cyperaceae	*Carex*	*Carex atrofuscoides*	Perennial herbs	Flower、Fruit	Purple	SU	Brown	SU
Cyperaceae	*Carex*	*Carex breviaristata*	Perennial herbs	Flower、Fruit		SP、SU	Brown	SP、SU
Cyperaceae	*Carex*	*Carex breviculmis*	Perennial herbs	Flower、Fruit	White	SP、SU	Brown	SP、SU
Cyperaceae	*Carex*	*Carex brunnea*	Perennial herbs	Flower、Fruit	Yellow		Brown	
Cyperaceae	*Carex*	*Carex capilliformis*	Perennial herbs	Flower	Yellow	SU、AU	Black	SU、AU
Cyperaceae	*Carex*	*Carex cinerascens*	Perennial herbs	Flower		SP	Brown	SP
Cyperaceae	*Carex*	*Carex davidii*	Perennial herbs	Flower	Yellow	SP、SU	Multicolour(Fruit shape)	SP、SU
Cyperaceae	*Carex*	*Carex dimorpholepis*	Perennial herbs	Flower		SP、SU	Brown	SP、SU
Cyper	*Carex*	*Carex diplodon*	Perennia	Flowe	Gree	SP	Brown	SP

aceae			l herbs	r	n			
Cyperaceae	*Carex*	*Carex doniana*	Perennial herbs	Flower	Yellow	SP、SU	Yellow	SU、AU
Cyperaceae	*Carex*	*Carex gibba*	Perennial herbs	Flower		SP、SU	Yellow	SP、SU
Cyperaceae	*Carex*	*Carex giraldiana*	Perennial herbs	Flower		SP	Yellow	SP
Cyperaceae	*Carex*	*Carex grandiligulata*	Perennial herbs	Flower		SP	Multicolour(Fruit shape)	SP
Cyperaceae	*Carex*	*Carex henryi*	Perennial herbs	Flower	Yellow	SU、AU	Yellow	SU、AU
Cyperaceae	*Carex*	*Carex heterolepis*	Perennial herbs	Flower		SP、SU	Brown	SP、SU
Cyperaceae	*Carex*	*Carex heterostachya*	Perennial herbs	Flower		SP、SU	Brown	SP、SU
Cyperaceae	*Carex*	*Carex heudesii*	Perennial herbs	Flower		SP、SU	Black	SP、SU
Cyperaceae	*Carex*	*Carex japonica*	Perennial herbs	Flower	White	SP、SU	Brown	SP、SU
Cyperaceae	*Carex*	*Carex kansuensis*	Perennial herbs	Flower	Purple	SU、AU	Yellow	SU、AU
Cyperaceae	*Carex*	*Carex lanceolata*	Perennial herbs	Fruit			Multicolour(Fruit shape)	
Cyperaceae	*Carex*	*Carex lanceolata var. subpediformis*	Perennial herbs	观叶				
Cyperaceae	*Carex*	*Carex ligulata*	Perennial herbs	Flower		SP、SU	Brown	SP、SU
Cyperaceae	*Carex*	*Carex lithophila*	Perennial herbs	Flower	Green	SP、SU	Brown	SP、SU
Cyperaceae	*Carex*	*Carex luctuosa*	Perennial herbs	Flower	Red	SP、SU	Brown	SP、SU
Cyperaceae	*Carex*	*Carex meihsienica*	Perennial herbs	Fruit			Brown	
Cyperaceae	*Carex*	*Carex neurocarpa*	Perennial herbs	Flower	Yellow	SU	Brown	SU
Cyperaceae	*Carex*	*Carex nubigena*	Perennial herbs	Flower	Green	SU	Brown	SU
Cyperaceae	*Carex*	*Carex omeiensis*	Perennial herbs	Flower	Yellow	SP、SU	Brown	SP、SU
Cyperaceae	*Carex*	*Carex onoei*	Perennial herbs	Fruit			Brown	

Cyperaceae	*Carex*	*Carex parva*	Perennial herbs	Flower	Red	SP、SU	Multicolour(Fruit shape)	SP、SU
Cyperaceae	*Carex*	*Carex pediformis*	Perennial herbs	Fruit			Multicolour(Fruit shape)	
Cyperaceae	*Carex*	*Carex planiculmis*	Perennial herbs	Flower	Yellow	SP、SU	Brown	SP、SU
Cyperaceae	*Carex*	*Carex polyschoenoides*	Perennial herbs	Flower	Red	SP	Brown	SP
Cyperaceae	*Carex*	*Carex pseudodispalata*	Perennial herbs	Fruit			Yellow	
Cyperaceae	*Carex*	*Carex remotiuscula*	Perennial herbs	Flower	White	SU	Yellow	SU
Cyperaceae	*Carex*	*Carex rochebrunii*	Perennial herbs	Flower	Green	SP、SU	Yellow	SP、SU
Cyperaceae	*Carex*	*Carex scabrirostris*	Perennial herbs	Flower	Red	SU	Brown	SU
Cyperaceae	*Carex*	*Carex siderosticta*	Perennial herbs	Flower 、Fruit		SP	Multicolour(Fruit shape)	SP
Cyperaceae	*Carex*	*Carex stipitinux*	Perennial herbs	Flower	Yellow	SU、AU	Yellow	SU、AU
Cyperaceae	*Carex*	*Carex taipaishanica*	Perennial herbs	Flower	Green	SP	Brown	SP
Cyperaceae	*Carex*	*Carex tangiana*	Perennial herbs	Flower	Yellow	SP、SU	Yellow	SP、SU
Cyperaceae	*Carex*	*Carex thibetica*	Perennial herbs	观叶				
Dioscoreaceae	*Dioscorea*	*Dioscorea nipponica*	Perennial herbs	Fruit			Multicolour(Fruit shape)	SU
Ebenaceae	*Diospyros*	*Diospyros lotus var. mollissima*	Deciduous tree	Flower	Red	SP	Black	AU
Ebenaceae	*Diospyros*	*Diospyros cathayensis*	Evergreen tree	Flower	White	SP	Yellow	AU
Ebenaceae	*Diospyros*	*Diospyros kaki var. silvestris*	Deciduous tree	Flower	Yellow	SP	Yellow	AU
Ebenaceae	*Diospyros*	*Diospyros variegata*	Deciduous tree	Fruit			Red	AU
Ebenaceae	*Diospyros*	*Diospyros lotus*	Deciduous tree	Flower	Red	SP	Black	AU
Ebenaceae	*Diospyros*	*Diospyros armata*	Evergreen shrub	Flower	White	SP	Yellow	AU

Elaeagnaceae	*Elaeagnus*	*Elaeagnus wushanensis*	Deciduous shrub	Flower	White	SP	Red	AU
Elaeagnaceae	*Elaeagnus*	*Elaeagnus angustata*	Deciduous shrub	Flower	White	SP	Red	SU
Elaeagnaceae	*Elaeagnus*	*Elaeagnus mollis*	Deciduous shrub	Flower	Green	SU	Multicolour(Fruit shape)	AU
Elaeagnaceae	*Elaeagnus*	*Elaeagnus multiflora*	Deciduous shrub	Flower	White	SP	Red	SU
Elaeagnaceae	*Elaeagnus*	*Elaeagnus henryi*	Evergreen shrub	Flower	White	AU	Red	SP
Elaeagnaceae	*Elaeagnus*	*Elaeagnus lanceolata*	Evergreen shrub	Flower	Yellow	SU	Red	SP
Elaeagnaceae	*Elaeagnus*	*Elaeagnus umbellata*	Deciduous shrub	Flower	Yellow	SP	Red	SU
Elaeagnaceae	*Elaeagnus*	*Elaeagnus bockii*	Evergreen shrub	Flower	White	AU	Red	SP
Elaeagnaceae	*Elaeagnus*	*Elaeagnus pungens*	Evergreen shrub	Flower	White	AU	Red	SP
Ericaceae	*Lyonia*	*Lyonia ovalifolia var. hebecarpa*	Evergreen shrub	Flower	White	SP		
Ericaceae	*Lyonia*	*Lyonia ovalifolia*	Evergreen shrub	Flower	White	SP		
Ericaceae	*Vaccinium*	*Vaccinium vitis-idaea*	Evergreen shrub	Flower	White	SU	Red	AU
Ericaceae	*Vaccinium*	*Vaccinium japonicum var. sinicum*	Deciduous shrub	Flower	White	SU	Red	AU
Ericaceae	*Vaccinium*	*Vaccinium henryi*	Deciduous shrub	Flower	Green	SU	Purple	AU
Ericaceae	*Cassiope*	*Cassiope selaginoides*	Evergreen shrub	Flower	White	SP		
Ericaceae	*Monotropa*	*Monotropa uniflora*	Perennial herbs	Flower	White	SU	Multicolour(Fruit shape)	AU
Ericac	*Mono*	*Monotropa*	Perennia	Flowe	Whit	SU	Multicol	SU

eae	*tropa*	*hypopitys*	l herbs	r	e			our(Fruit shape)
Ericaceae	*Pieris*	*Pieris formosa*	Evergreen shrub	Flower	White	SP		
Ericaceae	*Rhododendron*	*Rhododendron taibaiense*	Evergreen shrub	Flower	Purple	SU		
Ericaceae	*Rhododendron*	*Rhododendron detersile*	Evergreen shrub	Flower	Pink	SP		
Ericaceae	*Rhododendron*	*Rhododendron capitatum*	Evergreen shrub	Flower	Purple	SP		
Ericaceae	*Rhododendron*	*Rhododendron roxieanum*	Evergreen shrub	Flower	White	SU		
Ericaceae	*Rhododendron*	*Rhododendron declivatum*	Evergreen shrub	Flower	Purple	SU		
Ericaceae	*Rhododendron*	*Rhododendron oreodoxa var. shensiense*	Evergreen shrub	Flower	Pink	SP		
Ericaceae	*Rhododendron*	*Rhododendron przewalskii*	Evergreen shrub	Flower	White	SU		
Ericaceae	*Rhododendron*	*Rhododendron calophytum*	Evergreen shrub	Flower	Pink	SP		
Ericaceae	*Rhododendron*	*Rhododendron hunnewellianum*	Evergreen shrub	Flower	Pink	SP		
Ericaceae	*Rhododendron*	*Rhododendron purdomii*	Evergreen shrub	Flower	White	SP		
Ericaceae	*Rhododendron*	*Rhododendron sutchuenense*	Evergreen shrub	Flower	Red	SP		
Ericaceae	*Rhododendron*	*Rhododendron clementinae subsp. aureodorsale*	Evergreen shrub	Flower	White	SP		
Ericaceae	*Rhododendron*	*Rhododendron fortunei*	Evergreen shrub	Flower	Pink	SP		

Ericaceae	*Rhododendron*	*Rhododendron oreodoxa var. fargesii*	Evergreen shrub	Flower	Pink	SP		
Ericaceae	*Rhododendron*	*Rhododendron mucronulatum*	Deciduous shrub	Flower	Purple	SP		
Ericaceae	*Rhododendron*	*Rhododendron discolor*	Evergreen shrub	Flower	White	SU		
Ericaceae	*Rhododendron*	*Rhododendron tsinlingense*	Evergreen shrub	Flower	Pink	SP		
Ericaceae	*Rhododendron*	*Rhododendron hypoglaucum*	Evergreen shrub	Flower	White	SP		
Ericaceae	*Rhododendron*	*Rhododendron concinnum*	Evergreen shrub	Flower	Purple	SP		
Ericaceae	*Rhododendron*	*Rhododendron rufum*	Evergreen shrub	Flower	White	SP		
Ericaceae	*Rhododendron*	*Rhododendron maculiferum*	Evergreen shrub	Flower	White	SP		
Ericaceae	*Rhododendron*	*Rhododendron augustinii*	Evergreen shrub	Flower	Purple	SP		
Ericaceae	*Rhododendron*	*Rhododendron mariesii*	Deciduous shrub	Flower	Pink	SP		
Ericaceae	*Rhododendron*	*Rhododendron stamineum*	Evergreen shrub	Flower	White	SP		
Ericaceae	*Rhododendron*	*Rhododendron simsii*	Deciduous shrub	Flower	Red	SP		
Ericaceae	*Arctous*	*Arctous alpinus*	Deciduous shrub	Flower、Fruit	Green	SP	Black	SU
Eucommiaceae	*Eucommia*	*Eucommia ulmoides*	Deciduous tree	Flower	Yellow	SP	Multicolour(Fruit shape)	AU
Euphorbiace	*Vernicia*	*Vernicia fordii*	Deciduous tree	Flower	White	SP		

ae								
Euphorbiaceae	*Mallotus*	*Mallotus tenuifolius*	Deciduous tree	Flower	Yellow	SU	Multicolour(Fruit shape)	AU
Euphorbiaceae	*Mallotus*	*Mallotus philippensis*	Evergreen tree	Flower	Yellow	SP	Red	SU
Euphorbiaceae	*Mallotus*	*Mallotus repandus var. chrysocarpus*	Deciduous shrub	Flower	Yellow	SP	Yellow	AU
Euphorbiaceae	*Mallotus*	*Mallotus tenuifolius var. paxii*	Deciduous shrub	Flower	Yellow	SU	Multicolour(Fruit shape)	AU
Euphorbiaceae	*Triadica*	*Triadica sebifera*	Deciduous tree	Flower	Yellow	SU	Black	AU
Euphorbiaceae	*Alchornea*	*Alchornea davidii*	Deciduous shrub	Flower	Green	SP		
Euphorbiaceae	*Excoecaria*	*Excoecaria acerifolia*	Evergreen shrub	Flower	Yellow	SU		
Euphorbiaceae	*Discocleidion*	*Discocleidion rufescens*	Deciduous shrub	Flower	Yellow	SP		
Euphorbiaceae	*Euphorbia*	*Euphorbia lathyris*	Annual and biennial herbs	Flower、Fruit	Yellow	SP	Multicolour(Fruit shape)	AU
Euphorbiaceae	*Euphorbia*	*Euphorbia kansui*	Perennial herbs	Flower、Fruit	Yellow	SP	Multicolour(Fruit shape)	AU
Euphorbiaceae	*Euphorbia*	*Euphorbia helioscopia*	Annual and biennial herbs	Flower	Green	SP、SU		
Euphorbiaceae	*Euphorbia*	*Euphorbia hylonoma*	Perennial herbs	Flower	Green	SU		
Euphorbiaceae	*Triadica*	*Neoshirakia japonica*	Deciduous tree	Flower	Yellow	SU		
Euphorbiace	*Euphorbia*	*Euphorbia esula*	Perennial herbs	Flower、		SP、SU	Multicolour(Frui	SU、AU

ae				Fruit			t shape)	
Euphorbiaceae	*Euphorbia*	*Euphorbia humifusa*	Annual herbs	Flower、Fruit		SU、AU	Multicolour(Fruit shape)	SU、AU
Euphorbiaceae	*Euphorbia*	*Euphorbia micractina*	Perennial herbs	Flower、Fruit		SU	Multicolour(Fruit shape)	SU
Euphorbiaceae	*Euphorbia*	*Euphorbia pekinensis*	Perennial herbs	Flower、Fruit		SP、SU	Multicolour(Fruit shape)	SU、AU
Eupteleaceae	*Euptelea*	*Euptelea pleiosperma*	Deciduous tree	Flower	Red	SP	Multicolour(Fruit shape)	AU
Fabaceae	*Wisteria*	*Wisteria sinensis*	Deciduous liana	Flower、Fruit	Purple	SP	Multicolour(Fruit shape)	SU
Fabaceae	*Cercis*	*Cercis racemosa*	Deciduous tree	Flower、Fruit	Pink	SP	Multicolour(Fruit shape)	AU
Fabaceae	*Cercis*	*Cercis glabra*	Deciduous tree	Flower、Fruit	Purple	SP	Multicolour(Fruit shape)	AU
Fabaceae	*Podocarpium*	*Hylodesmum podocarpum var. szechuenense*	Perennial herbs	Flower	Pink	AU		
Fabaceae	*Gleditsia*	*Gleditsia sinensis*	Deciduous tree	Flower	Green	SP	Multicolour(Fruit shape)	SU
Fabaceae	*Bauhinia*	*Bauhinia glauca*	Evergreen liana	Flower	White	SP	Multicolour(Fruit shape)	SU
Fabaceae	*Caesalpinia*	*Caesalpinia decapetala*	Deciduous liana	Flower	Yellow	SP	Multicolour(Fruit shape)	AU
Fabaceae	*Mucuna*	*Mucuna sempervirens*	Evergreen liana	Flower	Purple	SP	Multicolour(Fruit shape)	AU
Fabaceae	*Vicia*	*Vicia unijuga*	Perennial herbs	Flower、Fruit	Purple	SU	Multicolour(Fruit shape)	AU
Fabaceae	*Vicia*	*Vicia cracca*	Perennial herbs	Flower	Purple	SP、SU	Multicolour(Fruit shape)	SU

Fabaceae	*Vicia*	*Vicia latibracteolata*	Perennial herbs	Flower	Purple	AU		
Fabaceae	*Vicia*	*Vicia taipaica*	Perennial herbs	Flower	Red	AU		
Fabaceae	*Thermopsis*	*Thermopsis lanceolata*	Perennial herbs	Flower	Yellow	SP	Multicolour(Fruit shape)	AU
Fabaceae	*Thermopsis*	*Thermopsis chinensis*	Perennial herbs	Flower	Yellow	SP	Multicolour(Fruit shape)	SU
Fabaceae	*Thermopsis*	*Thermopsis alpina*	Perennial herbs	Flower	Yellow	SU		
Fabaceae	*Hedysarum*	*Hedysarum dentatoalatum*	Perennial herbs	Flower、Fruit	Purple	SP	Multicolour(Fruit shape)	SU
Fabaceae	*Hedysarum*	*Hedysarum taipeicum*	Perennial herbs	Flower	Yellow	SU	Multicolour(Fruit shape)	SU
Fabaceae	*Cladrastis*	*Cladrastis wilsonii*	Deciduous tree	Flower	White	SU	Multicolour(Fruit shape)	AU
Fabaceae	*Cladrastis*	*Cladrastis delavayi*	Deciduous tree	Flower	White	SU	Multicolour(Fruit shape)	AU
Fabaceae	*Apios*	*Apios fortunei*	Perennial herbs	Flower、Fruit	Green		Multicolour(Fruit shape)	AU
Fabaceae	*Desmodium*	*Desmodium elegans*	Deciduous shrub	Flower	Purple	SU	Multicolour(Fruit shape)	AU
Fabaceae	*Lathyrus*	*Lathyrus davidii*	Perennial herbs	Flower	Yellow	SP	Multicolour(Fruit shape)	SU
Fabaceae	*Chamaecrista*	*Cassia mimosoides*	Perennial herbs	Flower	Yellow	SU		
Fabaceae	*Medicago*	*Medicago ruthenica*	Perennial herbs	Flower	Yellow	SU	Multicolour(Fruit shape)	AU
Fabaceae	*Indigofera*	*Indigofera kirilowii*	Deciduous shrub	Flower	Purple	SU	Multicolour(Fruit shape)	SU
Fabac	*Indigo*	*Indigofera*	Deciduo	Flowe	Purpl	SU	Multicol	AU

eae	*fera*	*amblyantha*	us shrub	r	e		our(Fruit shape)	
Fabaceae	*Indigofera*	*Indigofera silvestrii*	Deciduous shrub	Flower	Purple	SU	Multicolour(Fruit shape)	AU
Fabaceae	*Indigofera*	*Indigofera carlesii*	Deciduous shrub	Flower	Pink	SP	Multicolour(Fruit shape)	AU
Fabaceae	*Indigofera*	*Indigofera bungeana*	Deciduous shrub	Flower	Purple	SU	Multicolour(Fruit shape)	AU
Fabaceae	*Indigofera*	*Indigofera fortunei*	Deciduous shrub	Flower	Purple	SP	Multicolour(Fruit shape)	SU
Fabaceae	*Gueldenstaedtia*	*Gueldenstaedtia verna*	Perennial herbs	Flower	Red	SP	Multicolour(Fruit shape)	SU
Fabaceae	*Maackia*	*Maackia hupehensis*	Deciduous tree	Flower	White	SU	Multicolour(Fruit shape)	AU
Fabaceae	*Maackia*	*Maackia hwashanensis*	Deciduous tree	Flower	White	SU	Multicolour(Fruit shape)	AU
Fabaceae	*Amphicarpaea*	*Amphicarpaea edgeworthii*	Annual herbs	Flower、Fruit	Multicolour	AU	Black	AU
Fabaceae	*Sphaerophysa*	*Sphaerophysa salsula*	Perennial herbs	Flower	Purple	SP	Multicolour(Fruit shape)	SU
Fabaceae	*Sophora*	*Sophora davidii*	Deciduous shrub	Flower	White	SP	Multicolour(Fruit shape)	SU
Fabaceae	*Sophora*	*Sophora flavescens*	Perennial herbs	Flower、Fruit	White	SU	Multicolour(Fruit shape)	AU
Fabaceae	*Caragana*	*Caragana arborescens*	Deciduous tree	Flower	Yellow	SU	Multicolour(Fruit shape)	AU
Fabaceae	*Caragana*	*Caragana stipitata*	Deciduous shrub	Flower	Yellow	SP	Multicolour(Fruit shape)	SU
Fabaceae	*Caragana*	*Caragana boisii*	Deciduous shrub	Flower	Yellow	SP	Multicolour(Fruit shape)	SU

Fabaceae	*Caragana*	*Caragana microphylla*	Deciduous shrub	Flower	Yellow	SU	Multicolour(Fruit shape)	SU
Fabaceae	*Caragana*	*Caragana rosea*	Deciduous shrub	Flower	Yellow	SP	Multicolour(Fruit shape)	SU
Fabaceae	*Caragana*	*Caragana leveillei*	Deciduous shrub	Flower	Yellow	SP	Multicolour(Fruit shape)	SU
Fabaceae	*Caragana*	*Caragana sinica*	Deciduous shrub	Flower	Yellow	SP	Multicolour(Fruit shape)	SU
Fabaceae	*Caragana*	*Caragana shensiensis*	Deciduous shrub	Flower	Yellow	SP		
Fabaceae	*Vigna*	*Vigna vexillata*	Perennial herbs	Flower	Purple	SU	Multicolour(Fruit shape)	AU
Fabaceae	*Oxytropis*	*Oxytropis melanocalyx*	Perennial herbs	Flower	Purple	SU	Multicolour(Fruit shape)	AU
Fabaceae	*Oxytropis*	*Oxytropis chinglingensis*	Perennial herbs	Flower	Purple	SU		
Fabaceae	*Oxytropis*	*Oxytropis sitaipaiensis*	Perennial herbs	Flower	Purple	SU		
Fabaceae	*Callerya*	*Callerya dielsiana*	Evergreen liana	Flower	Purple	SP	Multicolour(Fruit shape)	AU
Fabaceae	*Callerya*	*Callerya reticulata*	Evergreen liana	Flower	Purple	SP	Multicolour(Fruit shape)	AU
Fabaceae	*Dalbergia*	*Dalbergia mimosoides*	Deciduous shrub	Flower	White	SP		
Fabaceae	*Dalbergia*	*Dalbergia dyeriana*	Deciduous liana	Flower	Yellow	SP		
Fabaceae	*Dalbergia*	*Dalbergia hupeana*	Deciduous tree	Flower	White	SU		
Fabaceae	*Astragalus*	*Astragalus laxmannii*	Perennial herbs	Flower	Purple	SU		
Fabaceae	*Astragalus*	*Astragalus chrysopterus*	Perennial herbs	Flower	Yellow	SU		
Fabaceae	*Hedysarum*	*Hedysarum alpinum*	Perennial herbs	Flower	Purple	SU	Multicolour(Fruit shape)	SU、AU
Fabac	*Astra*	*Astragalus*	Perennia	Flowe	Yello	SU		

eae	*galus*	*havianus*	l herbs	r	w			
Fabaceae	*Astragalus*	*Astragalus membranaceus*	Perennial herbs	Flower、Fruit	Yellow	SU	Multicolour(Fruit shape)	AU
Fabaceae	*Astragalus*	*Astragalus leansanicus*	Perennial herbs	Flower	Multicolour	SU		
Fabaceae	*Astragalus*	*Astragalus sinicus*	Annual and biennial herbs	Flower	Purple	SP	Multicolour(Fruit shape)	SU
Fabaceae	*Astragalus*	*Astragalus bhotanensis*	Perennial herbs	Flower	Purple	SP	Brown	AU
Fabaceae	*Piptanthus*	*Piptanthus nepalensis*	Deciduous shrub	Flower	Yellow	SP	Multicolour(Fruit shape)	SU
Fabaceae	*Lespedeza*	*Lespedeza buergeri*	Deciduous shrub	Flower	Green	SU	Multicolour(Fruit shape)	AU
Fabaceae	*Lespedeza*	*Lespedeza thunbergii*	Deciduous shrub	Flower	Red	SU		
Fabaceae	*Lespedeza*	*Lespedeza virgata*	Deciduous shrub	Flower	White	SU		
Fabaceae	*Lespedeza*	*Lespedeza bicolor*	Deciduous shrub	Flower	Purple	SU	Multicolour(Fruit shape)	AU
Fabaceae	*Lespedeza*	*Lespedeza cyrtobotrya*	Deciduous shrub	Flower	Purple	SU	Multicolour(Fruit shape)	AU
Fabaceae	*Lespedeza*	*Lespedeza tomentosa*	Deciduous shrub	Flower	White	SU		
Fabaceae	*Lespedeza*	*Lespedeza inschanica*	Deciduous shrub	Flower	White	SU		
Fabaceae	*Lespedeza*	*Lespedeza caraganae*	Deciduous shrub	Flower	White	SU	Multicolour(Fruit shape)	AU
Fabaceae	*Lespedeza*	*Lespedeza floribunda*	Deciduous shrub	Flower	Purple	SU	Multicolour(Fruit shape)	AU
Fabaceae	*Ormosia*	*Ormosia hosiei*	Evergreen tree	Flower、Fruit	Multicolour	SP	Multicolour(Fruit shape)	AU
Fabaceae	*Albizia*	*Albizia julibrissin*	Deciduous tree	Flower、	Pink	SU	Multicolour(Frui	AU

				Fruit			t shape)	
Fabaceae	*Albizia*	*Albizia kalkora*	Deciduous tree	Flower、Fruit	White	SU	Multicolour(Fruit shape)	AU
Fabaceae	*Campylotropis*	*Campylotropis macrocarpa* var. *hupehensis*	Deciduous shrub	Flower、Fruit	Purple	SU	Multicolour(Fruit shape)	AU
Fabaceae	*Campylotropis*	*Campylotropis macrocarpa*	Deciduous shrub	Flower、Fruit	Purple	SU	Multicolour(Fruit shape)	AU
Fabaceae	*Glycyrrhiza*	*Glycyrrhiza pallidiflora*	Perennial herbs	Flower、Fruit	Purple	SU	Multicolour(Fruit shape)	AU
Fabaceae	*Melilotus*	*Melilotus albus*	Annual and biennial herbs	Flower	White	SU	Multicolour(Fruit shape)	SU、AU
Fabaceae	*Lotus*	*Lotus corniculatus*	Perennial herbs	Flower	Yellow	SP、SU	Multicolour(Fruit shape)	AU
Fagaceae	*Castanea*	*Castanea mollissima*	Deciduous tree	Flower	Yellow	SP	Multicolour(Fruit shape)	AU
Fagaceae	*Castanea*	*Castanea seguinii*	Deciduous tree	Flower	Yellow	SP	Multicolour(Fruit shape)	AU
Fagaceae	*Castanea*	*Castanea henryi*	Deciduous tree	Flower	Yellow	SP	Multicolour(Fruit shape)	AU
Flacourtiaceae	*Poliothyrsis*	*Poliothyrsis sinensis*	Deciduous tree	Flower	Yellow	SU		
Gentianaceae	*Swertia*	*Swertia bimaculata*	Annual herbs	Flower	Yellow	SU	Multicolour(Fruit shape)	SU
Gentianaceae	*Swertia*	*Swertia bifolia*	Perennial herbs	Flower	Purple	SU		
Gentianaceae	*Tripterospermum*	*Tripterospermum chinense*	Perennial herbs	Flower	Purple	AU	Multicolour(Fruit shape)	AU
Gentianaceae	*Tripterospermum*	*Tripterospermum filicaule*	Perennial herbs	Flower	Purple	AU		

Gentianaceae	*Gentiana*	*Gentiana squarrosa*	Annual herbs	Flower、Fruit	Purple	SU	Black	AU
Gentianaceae	*Gentiana*	*Gentiana spathulifolia*	Annual herbs	Flower	Purple	SU	Multicolour(Fruit shape)	SU
Gentianaceae	*Gentiana*	*Gentiana scabra*	Perennial herbs	Flower	Purple	SP、SU	Multicolour(Fruit shape)	SU
Gentianaceae	*Gentiana*	*Gentiana rhodantha*	Perennial herbs	Flower	Red	AU	Multicolour(Fruit shape)	WI
Gentianaceae	*Gentiana*	*Gentiana macrophylla*	Perennial herbs	Flower	Purple	SU	Multicolour(Fruit shape)	AU
Gentianaceae	*Gentiana*	*Gentiana arethusae*	Perennial herbs	Flower	Purple	SU	Multicolour(Fruit shape)	AU
Gentianaceae	*Gentiana*	*Gentiana apiata*	Perennial herbs	Flower	Yellow	SU		
Gentianaceae	*Lomatogonium*	*Lomatogonium bellum*	Annual herbs	Flower	Purple	SU	Multicolour(Fruit shape)	AU
Gentianaceae	*Halenia*	*Halenia elliptica*	Annual herbs	Flower	Purple	SU	Multicolour(Fruit shape)	SU
Gentianaceae	*Halenia*	*Halenia corniculata*	Annual herbs	Flower	Yellow	SU	Multicolour(Fruit shape)	SU
Gentianaceae	*Halenia*	*Halenia elliptica var. grandiflora*	Perennial herbs	Flower	Purple	SU		
Gentianaceae	*Gentianopsis*	*Gentianopsis paludosa*	Annual herbs	Flower	Purple	SU	Multicolour(Fruit shape)	AU
Gentianaceae	*Gentianopsis*	*Gentianopsis barbata*	Annual herbs	Flower	Purple	SU	Multicolour(Fruit shape)	SU
Gentianaceae	*Lomatogonium*	*Lomatogonium perenne*	Perennial herbs	Flower	Purple			
Geraniaceae	*Erodium*	*Erodium stephanianum*	Perennial herbs	Flower、	Purple	SU	Brown	AU

Geraniaceae	*Geranium*	*Geranium wilfordii*	Perennial herbs	Fruit Flower、Fruit	Multicolour	SU	Multicolour(Fruit shape)	SU、AU
Geraniaceae	*Geranium*	*Geranium nepalense*	Perennial herbs	Flower、Fruit	Purple	SP、SU	Multicolour(Fruit shape)	SU、AU
Geraniaceae	*Geranium*	*Geranium platyanthum*	Perennial herbs	Flower	Purple	SU	Multicolour(Fruit shape)	SU、AU
Geraniaceae	*Geranium*	*Geranium pylzowianum*	Perennial herbs	Flower	Purple	SU	Multicolour(Fruit shape)	AU
Geraniaceae	*Geranium*	*Geranium rosthornii*	Perennial herbs	Flower	Purple	SU	Multicolour(Fruit shape)	SU、AU
Geraniaceae	*Geranium*	*Geranium shensianum*	Perennial herbs	Flower	Multicolour	SU	Multicolour(Fruit shape)	SU
Gesneriaceae	*Boea*	*Dorcoceras hygrometrica*	Perennial herbs	Flower、Fruit	Purple	SU	Multicolour(Fruit shape)	AU
Gesneriaceae	*Petrocosmea*	*Petrocosmea sinensis*	Perennial herbs	Flower	Purple	SU		
Gesneriaceae	*Corallodiscus*	*Corallodiscus lanuginosus*	Perennial herbs	Flower、Fruit	Purple	SU	Multicolour(Fruit shape)	SU
Gesneriaceae	*Hemiboea*	*Hemiboea subcapitata*	Perennial herbs	Flower	White	AU	Multicolour(Fruit shape)	AU、WI
Gesneriaceae	*Ancylostemon*	*Ancylostemon saxatilis*	Perennial herbs	Flower	Yellow	SU		
Gesneriaceae	*Isometrum*	*Isometrum farreri*	Perennial herbs	Flower	Purple	SU		
Gesneriaceae	*Petrocosmea*	*Petrocosmea qinlingensis*	Perennial herbs	Flower	Purple	SU、AU		
Gramineae	*Setaria*	*Setaria forbesiana*	Perennial herbs	Flower		SU、AU	Multicolour(Fruit shape)	SU、AU
Grami	*Setari*	*Setaria pumila*	Annual	Flowe		SU、AU	Multicol	SU、AU

neae	*a*		herbs	r			our(Fruit shape)	
Gramineae	*Achnatherum*	*Achnatherum breviaristatum*	Perennial herbs	Flower、Fruit		SU	Multicolour(Fruit shape)	SU
Gramineae	*Achnatherum*	*Achnatherum chingii*	Perennial herbs	Flower、Fruit		SU	Multicolour(Fruit shape)	SU
Gramineae	*Achnatherum*	*Achnatherum pekinense*	Perennial herbs	Flower、Fruit		SU、AU	Multicolour(Fruit shape)	SU、AU
Gramineae	*Achnatherum*	*Achnatherum pubicalyx*	Perennial herbs	Flower、Fruit		SU、AU	Multicolour(Fruit shape)	SU、AU
Gramineae	*Agrostis*	*Agrostis clavata*	Perennial herbs	Flower		SU、AU		
Gramineae	*Alopecurus*	*Alopecurus aequalis*	Annual herbs	Flower、Fruit		SP	Multicolour(Fruit shape)	SP
Gramineae	*Alopecurus*	*Alopecurus japonicus*	Annual herbs	Flower、Fruit		SP	Multicolour(Fruit shape)	SP
Gramineae	*Arundinella*	*Arundinella hirta*	Perennial herbs	Flower、Fruit		SU、AU	Multicolour(Fruit shape)	SU、AU
Gramineae	*Avena*	*Avena chinensis*	Annual herbs	Flower、Fruit		SU、AU	Multicolour(Fruit shape)	SU、AU
Gramineae	*Avena*	*Avena fatua*	Annual herbs	Flower、Fruit		SP、SU	Multicolour(Fruit shape)	SU、AU
Gramineae	*Avena*	*Avena sativa*	Annual herbs	Flower、Fruit		SP、SU	Multicolour(Fruit shape)	SU、AU
Gramineae	*Bashania*	*Arundinaria fargesii*	Annual herbs	观叶				
Gramineae	*Beckmannia*	*Beckmannia syzigachne*	Annual herbs	Flower、Fruit	White	SP、SU	Brown	SU、AU
Gramineae	*Bromus*	*Bromus japonicus*	Annual herbs	Flower、Fruit		SP、SU	Yellow	SP、SU
Gramineae	*Bromus*	*Bromus plurinodis*	Perennial herbs	Flower、		SU	Multicolour(Frui	SU

				Fruit			t shape)	
Gramineae	*Bromus*	*Bromus remotiflorus*	Perennial herbs	Flower、Fruit		SU	Multicolour(Fruit shape)	SU
Gramineae	*Calamagrostis*	*Calamagrostis emodensis*	Perennial herbs	Flower、Fruit		SU、AU	Multicolour(Fruit shape)	SU、AU
Gramineae	*Calamagrostis*	*Calamagrostis epigeios*	Perennial herbs	Flower、Fruit		SP、SU	Multicolour(Fruit shape)	SU、AU
Gramineae	*Calamagrostis*	*Calamagrostis pseudophragmites*	Perennial herbs	Flower、Fruit		SU、AU	Multicolour(Fruit shape)	SU、AU
Gramineae	*Chloris*	*Chloris virgata*	Annual herbs	Flower、Fruit		SU、AU	Yellow	SU、AU
Gramineae	*Cynodon*	*Cynodon dactylon*	Annual herbs	Flower、Fruit		SU、AU	Multicolour(Fruit shape)	SU、AU
Gramineae	*Dactylis*	*Dactylis glomerata*	Perennial herbs	Flower	Green	SU、AU	Multicolour(Fruit shape)	SU、AU
Gramineae	*Deschampsia*	*Deschampsia littoralis*	Perennial herbs	Flower	Multicolour	SU、AU	Multicolour(Fruit shape)	SU、AU
Gramineae	*Deyeuxia*	*Deyeuxia pyramidalis*	Perennial herbs	Flower、Fruit		SU、AU	Multicolour(Fruit shape)	SU、AU
Gramineae	*Deyeuxia*	*Deyeuxia arundinacea var. borealis*	Perennial herbs	Flower、Fruit		SU、AU	Multicolour(Fruit shape)	SU、AU
Gramineae	*Deyeuxia*	*Deyeuxia effusiflora*	Perennial herbs	Flower、Fruit		SU、AU	Multicolour(Fruit shape)	SU、AU
Gramineae	*Deyeuxia*	*Deyeuxia scabrescens*	Perennial herbs	Flower、Fruit		SU、AU	Multicolour(Fruit shape)	SU、AU
Gramineae	*Deyeuxia*	*Deyeuxia sinelatior*	Perennial herbs	Flower、Fruit		AU	Multicolour(Fruit shape)	AU
Gramineae	*Digitaria*	*Digitaria ciliaris var. chrysoblephara*	Annual herbs	Flower、Fruit		SU、AU	Multicolour(Fruit shape)	SU、AU
Grami	*Digita*	*Digitaria*	Annual	Flowe		SU、AU	Multicol	SU、AU

neae	*ria*	*ischaemum*	herbs	r 、Fruit			our(Fruit shape)	
Gramineae	*Eleusine*	*Eleusine indica*	Annual herbs	Flower 、Fruit		SU、AU	Multicolour(Fruit shape)	SU、AU
Gramineae	*Elymus*	*Elymus dahuricus*	Perennial herbs	Flower 、Fruit		SU、AU	Multicolour(Fruit shape)	SU、AU
Gramineae	*Elymus*	*Elymus sibiricus*	Perennial herbs	Flower	Green	SU、AU	Multicolour(Fruit shape)	SU、AU
Gramineae	*Eragrostis*	*Eragrostis ferruginea*	Perennial herbs	Flower 、Fruit		SU、AU	Brown	SU、AU
Gramineae	*Eragrostis*	*Eragrostis nigra*	Perennial herbs	Flower	Green	SP、SU	Multicolour(Fruit shape)	SU、AU
Gramineae	*Eragrostis*	*Eragrostis pilosa*	Annual herbs	Flower 、Fruit		SU、AU	Multicolour(Fruit shape)	SU、AU
Gramineae	*Festuca*	*Festuca extremiorientalis*	Perennial herbs	Flower 、Fruit		SU	Multicolour(Fruit shape)	SU
Gramineae	*Festuca*	*Festuca japonica*	Perennial herbs	Flower 、Fruit		SU	Multicolour(Fruit shape)	SU
Gramineae	*Festuca*	*Festuca modesta*	Perennial herbs	Flower 、Fruit		SP、SU	Multicolour(Fruit shape)	SP、SU
Gramineae	*Festuca*	*Festuca ovina*	Perennial herbs	Flower 、Fruit		SU、AU	Multicolour(Fruit shape)	SU、AU
Gramineae	*Festuca*	*Festuca parvigluma*	Perennial herbs	Flower 、Fruit		SP、SU	Multicolour(Fruit shape)	SP、SU
Gramineae	*Helictotrichon*	*Helictotrichon delavayi*	Perennial herbs	Flower 、Fruit		SU	Multicolour(Fruit shape)	SU
Gramineae	*Helictotrichon*	*Helictotrichon leianthum*	Perennial herbs	Flower 、Fruit		SP、SU	Multicolour(Fruit shape)	SP、SU
Gramineae	*Heteropogon*	*Heteropogon contortus*	Perennial herbs	Flower 、Fruit		SP、SU、AU	Multicolour(Fruit shape)	SU、AU、WI

Gramineae	*Hierochloe*	*Anthoxanthum nitens*	Perennial herbs	Flower、Fruit		SU、AU	Multicolour(Fruit shape)	SU、AU
Gramineae	*Hystrix*	*Hystrix duthiei*	Perennial herbs	Flower、Fruit		SP、SU	Multicolour(Fruit shape)	SP、SU
Gramineae	*Leptochloa*	*Leptochloa chinensis*	Annual herbs	Flower、Fruit		SU、AU	Multicolour(Fruit shape)	SU、AU
Gramineae	*Melica*	*Melica onoei*	Perennial herbs	Flower、Fruit		SU、AU	Multicolour(Fruit shape)	SU、AU
Gramineae	*Melica*	*Melica przewalskyi*	Perennial herbs	Flower、Fruit		SU	Multicolour(Fruit shape)	SU
Gramineae	*Melica*	*Melica radula*	Perennial herbs	Flower	Green	SP、SU	Brown	SP、SU
Gramineae	*Melica*	*Melica scabrosa*	Perennial herbs	Flower、Fruit		SP、SU	Brown	SP、SU
Gramineae	*Milium*	*Milium effusum*	Perennial herbs	Flower、Fruit		SP、SU	Multicolour(Fruit shape)	SP、SU
Gramineae	*Muhlenbergia*	*Muhlenbergia huegelii*	Perennial herbs	Flower、Fruit		SU、AU	Multicolour(Fruit shape)	SU、AU
Gramineae	*Muhlenbergia*	*Muhlenbergia japonica*	Perennial herbs	Flower、Fruit		SU、AU	Multicolour(Fruit shape)	SU、AU
Gramineae	*Orthoraphium*	*Achnatherum coreanum*	Perennial herbs	Flower、Fruit		SU、AU	Multicolour(Fruit shape)	SU、AU
Gramineae	*Oryzopsis*	*Achnatherum chinense*	Perennial herbs	Flower、Fruit		SP、SU	Multicolour(Fruit shape)	SP、SU
Gramineae	*Oryzopsis*	*Achnatherum henryi*	Perennial herbs	Flower		SP、SU		
Gramineae	*Oryzopsis*	*Piptatherum kuoi*	Perennial herbs	Flower、Fruit		SP、SU	Multicolour(Fruit shape)	SP、SU
Gramineae	*Oryzopsis*	*Piptatherum tibeticum*	Perennial herbs	Flower、Fruit		SU	Multicolour(Fruit shape)	SU
Grami	*Paspa*	*Paspalum*	Perennia	Flowe		SP、SU	Multicol	SU、AU

neae	*lum*	*thunbergii*	l herbs	r、Fruit			our(Fruit shape)	
Gramineae	*Pennisetum*	*Pennisetum flaccidum*	Perennial herbs	Flower、Fruit		SU、AU	Multicolour(Fruit shape)	SU、AU
Gramineae	*Phleum*	*Phleum alpinum*	Perennial herbs	Flower、Fruit		SU、AU	Multicolour(Fruit shape)	SU、AU
Gramineae	*Phleum*	*Phleum paniculatum*	Annual herbs	Flower、Fruit		SP、SU	Multicolour(Fruit shape)	SP、SU
Gramineae	*Phleum*	*Phleum pratense*	Perennial herbs	Flower	Green	SU	Multicolour(Fruit shape)	SU
Gramineae	*Poa*	*Poa nemoralis*	Perennial herbs	Flower		SP、SU		
Gramineae	*Polypogon*	*Polypogon fugax*	Annual herbs	Flower、Fruit		SP、SU	Multicolour(Fruit shape)	SU、AU
Gramineae	*Psathyrostachys*	*Psathyrostachys huashanica*	Perennial herbs	Flower、Fruit		SP、SU	Multicolour(Fruit shape)	SP、SU
Gramineae	*Ptilagrostis*	*Ptilagrostis concinna*	Perennial herbs	Flower、Fruit		SU、AU	Multicolour(Fruit shape)	SU、AU
Gramineae	*Ptilagrostis*	*Ptilagrostis mongholica*	Perennial herbs	Flower、Fruit		SU	Multicolour(Fruit shape)	SU
Gramineae	*Roegneria*	*Elymus kamoji*	Perennial herbs	观叶				
Grossulariaceae	*Ribes*	*Ribes longiracemosum var. gracillimum*	Deciduous shrub	Flower、Fruit	Green	SP	Black	SU
Grossulariaceae	*Ribes*	*Ribes rubrisepalum*	Deciduous shrub	Flower、Fruit	Green	SP	Black	SU
Grossulariaceae	*Ribes*	*Ribes himalense*	Deciduous shrub	Flower、Fruit	Red	SP	Purple	SU
Grossulariaceae	*Ribes*	*Ribes himalense var. glandulosum*	Deciduous shrub	Flower、Fruit	Red	SP	Purple	SU
Grossulariac	*Ribes*	*Ribes franchetii*	Deciduous shrub	Flower、	Red	SP	Red	SU

eae				Fruit				
Grossulariaceae	*Ribes*	*Ribes moupinense var. tripartitum*	Deciduous shrub	Flower、Fruit	Green	SP	Black	SU
Grossulariaceae	*Ribes*	*Ribes vilmorinii*	Deciduous shrub	Flower、Fruit	Green	SP	Black	SU
Grossulariaceae	*Ribes*	*Ribes stenocarpum*	Deciduous shrub	Flower、Fruit	White	SP	Red	SU
Grossulariaceae	*Ribes*	*Ribes glaciale*	Deciduous shrub	Flower、Fruit	Yellow	SP	Red	SU
Grossulariaceae	*Ribes*	*Ribes burejense*	Deciduous shrub	Flower、Fruit	Pink	SP	Red	SU
Grossulariaceae	*Ribes*	*Ribes himalense var. verruculosum*	Deciduous shrub	Flower、Fruit	Red	SP	Red	SU
Grossulariaceae	*Ribes*	*Ribes mandshuricum var. subglabrum*	Deciduous shrub	Flower、Fruit	Green	SP	Red	SU
Grossulariaceae	*Ribes*	*Ribes moupinense*	Deciduous shrub	Flower、Fruit	Green	SP	Black	SU
Grossulariaceae	*Ribes*	*Ribes takare*	Deciduous shrub	Flower、Fruit	Green	SP	Red	SU
Grossulariaceae	*Ribes*	*Ribes alpestre*	Deciduous shrub	Flower、Fruit	White	SP	Red	SU
Grossulariaceae	*Ribes*	*Ribes komarovii*	Deciduous shrub	Flower、Fruit	Green	SP	Red	SU
Grossulariaceae	*Ribes*	*Ribes mandshuricum*	Deciduous shrub	Flower、Fruit	Green	SP	Red	SU
Grossulariaceae	*Ribes*	*Ribes tenue*	Deciduous shrub	Flower、Fruit	Red	SP	Red	SU
Grossulariaceae	*Ribes*	*Ribes fasciculatum var. chinense*	Deciduous shrub	Flower、Fruit	Yellow	SP	Red	SU
Gross	*Ribes*	*Ribes*	Deciduo	Flowe	Gree	SU	Red	SU

ulariaceae		*maximowiczii*	us shrub	r 、Fruit	n			
Grossulariaceae	*Ribes*	*Ribes giraldii*	Deciduous shrub	Flower 、Fruit	Yellow	SP	Red	SU
Grossulariaceae	*Ribes*	*Ribes glabrifolium*	Deciduous shrub	Flower 、Fruit	Yellow	SP	Red	SU
Guttiferae	*Amygdalus*	*Hypericum beanii*	Deciduous shrub	Flower	Yellow	SP		
Guttiferae	*Amygdalus*	*Hypericum monogynum*	Deciduous shrub	Flower	Yellow	SP		
Guttiferae	*Amygdalus*	*Hypericum elatoides*	Deciduous shrub	Flower	Yellow	SP		
Guttiferae	*Amygdalus*	*Hypericum patulum*	Deciduous shrub	Flower	Yellow	SU		
Guttiferae	*Amygdalus*	*Hypericum longistylum*	Deciduous shrub	Flower	Yellow	SP		
Guttiferae	*Amygdalus*	*Hypericum elatoides*	Evergreen shrub	Flower	Yellow	SU		
Guttiferae	*Amygdalus*	*Hypericum przewalskii*	Perennial herbs	Flower	Yellow	SU	Multicolour(Fruit shape)	AU
Guttiferae	*Amygdalus*	*Hypericum perforatum*	Perennial herbs	Flower	Yellow	SU	Multicolour(Fruit shape)	AU
Guttiferae	*Amygdalus*	*Hypericum attenuatum*	Perennial herbs	Flower	Yellow	SU	Multicolour(Fruit shape)	AU
Guttiferae	*Amygdalus*	*Hypericum ascyron*	Perennial herbs	Flower	Yellow	SU		
Hamamelidaceae	*Distylium*	*Distylium chinense*	Evergreen shrub	Flower	Red	SP		
Hamamelidaceae	*Distylium*	*Distylium buxifolium*	Evergreen shrub	Flower	Red	SP		
Hamamelidaceae	*Pyrus Sycopsis*	*Sycopsis sinensis*	Evergreen tree	Flower	Red	SP		
Hamamelidaceae	*Sinowilsonia*	*Sinowilsonia henryi*	Deciduous shrub	Flower	Green	SP		
Hama	*Fortu*	*Fortunearia*	Deciduo	Flowe	Red	SP		

melidaceae	*nearia*	*sinensis*	us shrub	r		
Hamamelidaceae	*Corylopsis*	*Corylopsis microcarpa*	Deciduous shrub	Flower	Yellow	SP
Hamamelidaceae	*Liquidambar*	*Liquidambar formosana*	Deciduous tree	Flower、Fruit	Red	SP
Hippocastanaceae	*Aesculus*	*Aesculus chinensis var. wilsonii*	Deciduous tree	Flower	White	SP
Hippocastanaceae	*Aesculus*	*Aesculus chinensis*	Deciduous tree	Flower	White	SP
Hydrangeaceae	*Schizophragma*	*Schizophragma integrifolium*	Deciduous liana	Flower	Green	SU
Hydrangeaceae	*Hydrangea*	*Hydrangea paniculata*	Deciduous shrub	Flower	White	SU
Hydrangeaceae	*Hydrangea*	*Hydrangea longipes var. lanceolata*	Deciduous shrub	Flower	White	SU
Hydrangeaceae	*Hydrangea*	*Hydrangea longipes var. fulvescens*	Deciduous shrub	Flower	White	SU
Hydrangeaceae	*Hydrangea*	*Hydrangea bretschneideri*	Deciduous shrub	Flower	White	SU
Hydrangeaceae	*Hydrangea*	*Hydrangea hypoglauca*	Deciduous shrub	Flower	White	SU
Hydrangeaceae	*Hydrangea*	*Hydrangea xanthoneura*	Deciduous shrub	Flower	White	SU
Hydrangeaceae	*Hydrangea*	*Hydrangea robusta*	Deciduous shrub	Flower	White	SU
Hydrangeaceae	*Hydrangea*	*Hydrangea longipes*	Deciduous shrub	Flower	White	SU
Hydrangeaceae	*Hydrangea*	*Hydrangea strigosa*	Deciduous shrub	Flower	Purple	SU

Hydrangeaceae	*Hydrangea*	*Hydrangea aspera*	Deciduous shrub	Flower	Purple	SU
Hydrangeaceae	*Hydrangea*	*Hydrangea anomala*	Deciduous liana	Flower	White	SP
Hydrangeaceae	*Deutzia*	*Deutzia longifolia*	Deciduous shrub	Flower	Pink	SU
Hydrangeaceae	*Deutzia*	*Deutzia hypoglauca*	Deciduous shrub	Flower	White	SP
Hydrangeaceae	*Deutzia*	*Deutzia parviflora var. micrantha*	Deciduous shrub	Flower	White	SU
Hydrangeaceae	*Deutzia*	*Deutzia taibaiensis*	Deciduous shrub	Flower	White	SP
Hydrangeaceae	*Deutzia*	*Deutzia discolor*	Deciduous shrub	Flower	White	SU
Hydrangeaceae	*Deutzia*	*Deutzia parviflora*	Deciduous shrub	Flower	White	SP
Hydrangeaceae	*Deutzia*	*Deutzia baroniana*	Deciduous shrub	Flower	White	SP
Hydrangeaceae	*Deutzia*	*Deutzia albida*	Deciduous shrub	Flower	White	SU
Hydrangeaceae	*Deutzia*	*Deutzia ningpoensis*	Deciduous shrub	Flower	White	SP
Hydrangeaceae	*Deutzia*	*Deutzia grandiflora*	Deciduous shrub	Flower	White	SP
Hydrangeaceae	*Philadelphus*	*Philadelphus incanus var. baileyi*	Deciduous shrub	Flower	White	SP
Hydrangeaceae	*Philadelphus*	*Philadelphus kansuensis*	Deciduous shrub	Flower	White	SU
Hydrangeac	*Philadelphu*	*Philadelphus laxiflorus*	Deciduous shrub	Flower	White	SP

eae	*s*							
Hydrangeaceae	*Philadelphus*	*Philadelphus sericanthus*	Deciduous shrub	Flower	White	SP		
Hydrangeaceae	*Philadelphus*	*Philadelphus incanus*	Deciduous shrub	Flower	White	SP		
Hydrangeaceae	*Philadelphus*	*Philadelphus pekinensis*	Deciduous shrub	Flower	White	SP		
Hydrangeaceae	*Decumaria*	*Decumaria sinensis*	Evergreen liana	Flower	White	SP		
Hydrangeaceae	*Dichroa*	*Dichroa febrifuga*	Deciduous shrub	Flower、Fruit	Purple	SP	Purple	SP
Hydrocharitaceae	*Hydrilla*	*Hydrilla verticillata*	Perennial herbs	Flower、Fruit	Multicolour	SP、SU	Multicolour(Fruit shape)	SU、AU
Icacinaceae	*Nothapodytes*	*Nothapodytes pittosporoides*	Evergreen shrub	Flower	Yellow	SP	Red	SU
Iridaceae	*Iris*	*Iris lactea*	Perennial herbs	Flower	Multicolour	SU	Multicolour(Fruit shape)	SU、AU
Iridaceae	*Iris*	*Iris wilsonii*	Perennial herbs	Flower、Fruit	Yellow	SP、SU	Multicolour(Fruit shape)	SU
Iridaceae	*Iris*	*Iris tectorum*	Perennial herbs	Flower	Purple	SP	Multicolour(Fruit shape)	SU
Iridaceae	*Iris*	*Iris songarica*	Perennial herbs	Flower	Purple	SU	Multicolour(Fruit shape)	AU
Iridaceae	*Iris*	*Iris japonica*	Perennial herbs	Flower	Purple	SP	Multicolour(Fruit shape)	SP、SU
Iridaceae	*Iris*	*Iris dichotoma*	Perennial herbs	Flower	Purple	SU	Multicolour(Fruit shape)	AU
Iridaceae	*Iris*	*Iris ruthenica*	Perennial herbs	Flower	Purple	SP		
Iridaceae	*Belamcan*	*Belamcanda chinensis*	Perennial herbs	Flower	Multicolo	SU	Multicolour(Frui	SU、AU

	da				ur		t shape)	
Iridaceae	*Iris*	*Iris goniocarpa*	Perennial herbs	Flower	Purple	SP、SU	Multicolour(Fruit shape)	SU
Juglandaceae	*Cyclocarya*	*Cyclocarya paliurus*	Deciduous tree	Flower、Fruit	Green	SP	Multicolour(Fruit shape)	SU
Juglandaceae	*Platycarya*	*Platycarya strobilacea*	Deciduous tree	Flower	Yellow	SP	Multicolour(Fruit shape)	SU
Juglandaceae	*Amygdalus*	*Juglans mandshurica*	Deciduous tree	Flower	Yellow	SP	Multicolour(Fruit shape)	AU
Juglandaceae	*Amygdalus*	*Juglans regia*	Deciduous tree	Flower	Yellow	SP	Multicolour(Fruit shape)	AU
Juglandaceae	*Populus*	*Pterocarya macroptera*	Deciduous tree	Flower、Fruit	Yellow	SP	Multicolour(Fruit shape)	AU
Juglandaceae	*Populus*	*Pterocarya macroptera var. insignis*	Deciduous tree	Flower、Fruit	Yellow	SP	Multicolour(Fruit shape)	AU
Juglandaceae	*Populus*	*Pterocarya hupehensis*	Deciduous tree	Flower、Fruit	Yellow	SP	Multicolour(Fruit shape)	AU
Juglandaceae	*Populus*	*Pterocarya stenoptera*	Deciduous tree	Flower、Fruit	Yellow	SP	Multicolour(Fruit shape)	AU
Juncaceae	*Juncus*	*Juncus alatus*	Perennial herbs	Flower	Yellow	SP、SU	Brown	SU、AU
Juncaceae	*Juncus*	*Juncus allioides*	Perennial herbs	Flower	Multicolour	SU	Brown	SU、AU
Juncaceae	*Juncus*	*Juncus amplifolius*	Perennial herbs	Flower	Red	SU	Brown	SU
Juncaceae	*Juncus*	*Juncus articulatus*	Perennial herbs	Flower	Red	SU	Brown	SU、AU
Juncaceae	*Juncus*	*Juncus bufonius*	Annual herbs	Flower、Fruit		SP、SU	Brown	SU、AU
Juncaceae	*Juncus*	*Juncus compressus*	Perennial herbs	Flower、Fruit		SP、SU	Brown	SU
Juncac	*Juncu*	*Juncus*	Perennia	Flowe	Gree	SP、SU	Brown	SU

eae	*s*	*diastrophanthus*	l herbs	r	n			
Juncaceae	*Juncus*	*Juncus effusus*	Perennial herbs	Flower	Green	SP、SU	Brown	SU、AU
Juncaceae	*Juncus*	*Juncus inflexus*	Perennial herbs	Flower	Multicolour	SU	Brown	SU、AU
Juncaceae	*Juncus*	*Juncus luzuliformis*	Perennial herbs	Flower	Green	SU	Brown	SU、AU
Juncaceae	*Juncus*	*Juncus modicus*	Perennial herbs	Flower	Yellow	SU	Brown	AU
Juncaceae	*Juncus*	*Juncus potaninii*	Perennial herbs	Flower	Multicolour	SU	Brown	SU、AU
Juncaceae	*Juncus*	*Juncus przewalskii*	Perennial herbs	Flower	Yellow	SU	Brown	SU、AU
Juncaceae	*Juncus*	*Juncus setchuensis var. effusoides*	Perennial herbs	Flower	Green	SU	Brown	SU、AU
Juncaceae	*Luzula*	*Luzula effusa*	Perennial herbs	Flower	Multicolour	SP、SU	Brown	SU
Juncaceae	*Luzula*	*Luzula multiflora*	Perennial herbs	Flower	Red	SP、SU	Brown	SU
Juncaceae	*Luzula*	*Luzula multiflora subsp. frigida*	Perennial herbs	Flower、Fruit		SP、SU	Brown	SU
Juncaceae	*Luzula*	*Luzula plumosa*	Perennial herbs	Flower	Red	SP	Yellow	SP、SU
Lamiaceae	*Callicarpa*	*Callicarpa dichotoma*	Deciduous shrub	Flower、Fruit	Purple	SU	Purple	AU
Lamiaceae	*Callicarpa*	*Callicarpa giraldii*	Deciduous shrub	Flower、Fruit	Purple	SU	Purple	AU
Lamiaceae	*Callicarpa*	*Callicarpa membranacea*	Deciduous shrub	Flower、Fruit	Purple	SU	Purple	AU
Lamiaceae	*Callicarpa*	*Callicarpa bodinieri var. rosthornii*	Deciduous shrub	Flower、Fruit	Purple	SU	Purple	AU
Lamiaceae	*Callicarpa*	*Callicarpa membranacea*	Deciduous shrub	Flower、Fruit	Purple	SU	Purple	AU
Lamia	*Callic*	*Callicarpa*	Deciduo	Flowe	Purpl	SU	Purple	AU

ceae	*arpa*	*japonica*	us shrub	r 、Fruit	e			
Lamiaceae	*Galeopsis*	*Galeopsis bifida* *Boenn.*	Annual herbs	Fruit			Multicolour(Fruit shape)	AU
Lamiaceae	*Caryopteris*	*Caryopteris incana*	Deciduous shrub	Flower	Purple	SU		
Lamiaceae	*Caryopteris*	*Caryopteris terniflora*	Deciduous shrub	Flower	Purple	SU		
Lamiaceae	*Leonurus*	*Leonurus pseudomacranthus*	Perennial herbs	Flower	White	SU	Multicolour(Fruit shape)	AU
Lamiaceae	*Sesamum*	*Heterolamium debile*	Perennial herbs	Flower	White	SU	Multicolour(Fruit shape)	AU
Lamiaceae	*Sesamum*	*Lamium barbatum*	Perennial herbs	Flower	Multicolour	SP	Multicolour(Fruit shape)	SU
Lamiaceae	*Isodon*	*Isodon serra*	Perennial herbs	Flower 、Fruit	Purple	SU	Multicolour(Fruit shape)	AU
Lamiaceae	*Isodon*	*Isodon nervosus*	Perennial herbs	Flower 、Fruit	Purple	SU	Multicolour(Fruit shape)	AU
Lamiaceae	*Prunella*	*Prunella vulgaris*	Perennial herbs	Flower	Purple	SU		
Lamiaceae	*Stachys*	*Stachys sieboldii*	Perennial herbs	Flower	Pink	SU	Multicolour(Fruit shape)	AU
Lamiaceae	*Stachys*	*Stachys baicalensis*	Perennial herbs	Flower	Purple	SU	Multicolour(Fruit shape)	SU
Lamiaceae	*Amethystea*	*Amethystea coerulea*	Annual herbs	Fruit			Multicolour(Fruit shape)	AU
Lamiaceae	*Salvia*	*Salvia miltiorrhiza*	Perennial herbs	Flower	Purple	SP	Multicolour(Fruit shape)	AU
Lamiaceae	*Salvia*	*Salvia maximowicziana*	Perennial herbs	Flower	Purple	SU		
Lamiaceae	*Dracocephalum*	*Dracocephalum moldavica*	Annual herbs	Flower	Purple	SU		

Lamiaceae	*Panzeria*	*Panzerina lanata var. alashanica*	Perennial herbs	Flower	Multicolour	SU		
Lamiaceae	*Origanum*	*Origanum vulgare*	Perennial herbs	Flower、Fruit	Multicolour	SU	Multicolour(Fruit shape)	AU
Lamiaceae	*Vitex*	*Vitex negundo*	Deciduous shrub	Flower	Purple	SU		
Lamiaceae	*Vitex*	*Vitex negundo var. cannabifolia*	Deciduous shrub	Flower	Purple	SU	Black	AU
Lamiaceae	*Nepeta*	*Nepeta fordii*	Perennial herbs	Flower	White	SP、SU		
Lamiaceae	*Nepeta*	*Nepeta cataria*	Perennial herbs	Flower	White	SU	Multicolour(Fruit shape)	AU
Lamiaceae	*Ajuga*	*Ajuga ciliata*	Perennial herbs	Flower、Fruit	Purple	SP	Multicolour(Fruit shape)	SU
Lamiaceae	*Glechoma*	*Glechoma biondiana*	Perennial herbs	Flower、Fruit	Purple	SP	Multicolour(Fruit shape)	SU
Lamiaceae	*Glechoma*	*Glechoma longituba*	Perennial herbs	Flower、Fruit	Purple	SP	Brown	SP
Lamiaceae	*Scutellaria*	*Scutellaria indica*	Perennial herbs	Flower	Purple	SP	Multicolour(Fruit shape)	SU
Lamiaceae	*Scutellaria*	*Scutellaria franchetiana*	Perennial herbs	Flower	Purple	SU		
Lamiaceae	*Scutellaria*	*Scutellaria barbata*	Perennial herbs	Flower	Purple	SP	Multicolour(Fruit shape)	SU
Lamiaceae	*Scutellaria*	*Scutellaria baicalensis*	Perennial herbs	Flower	Purple	SU	Multicolour(Fruit shape)	SU
Lamiaceae	*Rostrinucula*	*Rostrinucula dependens*	Deciduous shrub	Flower	Purple	AU		
Lamiaceae	*Clinopodium*	*Clinopodium polycephalum*	Perennial herbs	Flower、Fruit	Purple	SU	Multicolour(Fruit shape)	AU
Lamiaceae	*Premna*	*Premna puberula*	Deciduous shrub	Flower、Fruit	Yellow	SU	Black	SU

Lamiaceae	*Clerodendrum*	*Clerodendrum trichotomum*	Deciduous shrub	Flower、Fruit	White	SU	Purple	AU
Lamiaceae	*Clerodendrum*	*Clerodendrum bungei*	Deciduous shrub	Flower、Fruit	Pink	SU	Black	AU
Lamiaceae	*Phlomis*	*Phlomoides megalantha*	Perennial herbs	Flower	Yellow	SU	Multicolour(Fruit shape)	AU
Lardizabalaceae	*Archakebia*	*Archakebia apetala*	Deciduous liana	Flower	Yellow	SP	Yellow	AU
Lardizabalaceae	*Akebia*	*Akebia trifoliata*	Deciduous liana	Flower	Purple	SP	Purple	SU
Lardizabalaceae	*Akebia*	*Akebia quinata*	Deciduous liana	Flower	Purple	SP	Purple	SU
Lardizabalaceae	*Akebia*	*Akebia trifoliata subsp. australis*	Deciduous liana	Flower	Purple	SP	Yellow	SU
Lardizabalaceae	*Decaisnea*	*Decaisnea insignis*	Deciduous shrub	Flower	Green	SP	Purple	SU
Lardizabalaceae	*Sargentodoxa*	*Sargentodoxa cuneata*	Deciduous liana	Flower、Fruit	White	SP	Purple	SU
Lardizabalaceae	*Sinofranchetia*	*Sinofranchetia chinensis*	Deciduous liana	Flower、Fruit	White	SP	Purple	AU
Lardizabalaceae	*Holboellia*	*Holboellia angustifolia*	Evergreen liana	Flower、Fruit	Purple	SP	Purple	SU
Lardizabalaceae	*Holboellia*	*Holboellia grandiflora*	Evergreen liana	Flower、Fruit	Purple	SP	Purple	SU
Lardizabalaceae	*Holboellia*	*Holboellia coriacea*	Evergreen liana	Flower、Fruit	White	SP	Purple	SU
Lauraceae	*Lindera*	*Lindera erythrocarpa*	Deciduous shrub	Flower	Yellow	SP	Red	AU
Lauraceae	*Lindera*	*Lindera neesiana*	Deciduous shrub	Flower	Yellow	SP		
Lauraceae	*Linder*	*Lindera*	Evergree	Flowe	Yello	SP		

eae	*a*	*limprichtii*	n tree	r	w			
Lauraceae	*Lindera*	*Lindera obtusiloba*	Deciduous shrub	Flower	Yellow	SP		
Lauraceae	*Lindera*	*Lindera floribunda*	Evergreen tree	Flower	Yellow	SP		
Lauraceae	*Lindera*	*Lindera pulcherrima var. hemsleyana*	Evergreen tree	Flower	Yellow	SP		
Lauraceae	*Lindera*	*Lindera megaphylla*	Evergreen tree	Flower	Yellow	SP	Purple	AU
Lauraceae	*Lindera*	*Lindera communis*	Evergreen tree	Flower	Yellow	SP	Red	AU
Lauraceae	*Lindera*	*Lindera glauca*	Deciduous shrub	Flower	Yellow	SP	Black	SU
Lauraceae	*Lindera*	*Lindera aggregata*	Evergreen shrub	Flower	Yellow	SP	Purple	SU
Lauraceae	*Lindera*	*Lindera angustifolia*	Deciduous shrub	Flower	Green	SP	Black	AU
Lauraceae	*Lindera*	*Lindera fragrans*	Evergreen tree	Flower	Yellow	SP	Purple	SU
Lauraceae	*Lindera*	*Lindera pulcherrima var. attenuata*	Evergreen tree	Flower	Yellow	SP		
Lauraceae	*Litsea*	*Litsea mollis*	Deciduous shrub	Flower	Yellow	SP	Black	AU
Lauraceae	*Litsea*	*Litsea ichangensis*	Deciduous shrub	Flower	Yellow	SP	Black	SU
Lauraceae	*Litsea*	*Litsea veitchiana*	Deciduous shrub	Flower	Yellow	SP	Black	AU
Lauraceae	*Litsea*	*Litsea tsinlingensis*	Deciduous shrub	Flower	Yellow	SP		
Lauraceae	*Litsea*	*Litsea pungens*	Deciduous tree	Flower	Yellow	SP	Black	AU
Lauraceae	*Litsea*	*Litsea moupinensis var. szechuanica*	Deciduous shrub	Flower	Yellow	SP	Black	AU
Lauraceae	*Litsea*	*Litsea cubeba*	Deciduous shrub	Flower	Yellow	SP	Black	SU
Lauraceae	*Sassafras*	*Sassafras tzumu*	Deciduous tree	Flower	Yellow	SP	Black	SU
Leguminosae	*Astragalus*	*Astragalus dahuricus*	Annual and biennial herbs	Flower	Purple	SU、AU	Multicolour(Fruit shape)	SU、AU

Leguminosae	*Astragalus*	*Astragalus kifonsanicus*	Perennial herbs	Flower	Multicolour	SP	Multicolour(Fruit shape)	SU、AU
Leguminosae	*Astragalus*	*Astragalus leansanicus*	Perennial herbs	Flower	Purple	SP、SU	Multicolour(Fruit shape)	SU、AU
Leguminosae	*Astragalus*	*Astragalus taipaishanensis*	Perennial herbs	Flower	Purple	SU	Multicolour(Fruit shape)	SU、AU
Leguminosae	*Lens*	*Lens culinaris*	Annual herbs	Flower	Multicolour	SP、SU	Yellow	SU、AU
Leguminosae	*Oxytropis*	*Oxytropis melanocalyx*	Perennial herbs	Flower	Purple	SU	Multicolour(Fruit shape)	SU、AU
Leguminosae	*Oxytropis*	*Oxytropis sitaipaiensis var. brevidentata*	Perennial herbs	Flower	Purple			
Leguminosae	*Oxytropis*	*Oxytropis myriophylla*	Perennial herbs	Flower	Purple	SP、SU	Multicolour(Fruit shape)	SU
Leguminosae	*Oxytropis*	*Oxytropis taochensis*	Perennial herbs	Flower	Purple	SU	Multicolour(Fruit shape)	SU
Leguminosae	*Pisum*	*Pisum sativum*	Annual herbs	Flower	Multicolour	SU	Multicolour(Fruit shape)	SU、AU
Leguminosae	*Podocarpium*	*Hylodesmum podocarpum subsp. oxyphyllum*	Perennial herbs	Flower	Purple	SU、AU	Multicolour(Fruit shape)	SU、AU
Lentibulariaceae	*Pinguicula*	*Pinguicula alpina*	Perennial herbs	Flower	White	SP、SU	Multicolour(Fruit shape)	SU、AU
Liliaceae	*Amana*	*Tulipa edulis*	Perennial herbs	Flower	White	SP		
Liliaceae	*Hosta*	*Hosta ventricosa*	Perennial herbs	Flower	White	AU		
Liliaceae	*Tricyrtis*	*Tricyrtis macropoda*	Perennial herbs	Flower、Fruit	White	SU	Multicolour(Fruit shape)	AU
Liliaceae	*Tricyrtis*	*Tricyrtis latifolia*	Perennial herbs	Flower	Yellow	SU	Multicolour(Fruit shape)	AU

Liliaceae	*Trillium*	*Trillium tachonoakii*	Perennial herbs	Flower、Fruit	White	SP	Multicolour(Fruit shape)	SU
Liliaceae	*Hemerocallis*	*Hemerocallis lilioasphodelus*	Perennial herbs	Flower	Yellow	SU	Multicolour(Fruit shape)	AU
Liliaceae	*Hemerocallis*	*Hemerocallis fulva*	Perennial herbs	Flower	Yellow	SP、SU	Multicolour(Fruit shape)	SU
Liliaceae	*Hemerocallis*	*Hemerocallis citrina*	Perennial herbs	Flower	Yellow	SP、SU	Multicolour(Fruit shape)	SU、AU
Liliaceae	*Hemerocallis*	*Hemerocallis dumortieri*	Perennial herbs	Flower	Yellow	SU		
Liliaceae	*Smilacina*	*Maianthemum japonicum*	Perennial herbs	Flower	White	SP、SU	Multicolour(Fruit shape)	AU
Liliaceae	*Smilacina*	*Maianthemum henryi*	Perennial herbs	Flower	Yellow	SP、SU	Multicolour(Fruit shape)	AU
Liliaceae	*Maianthemum*	*Maianthemum bifolium*	Perennial herbs	Flower	White	SP、SU	Multicolour(Fruit shape)	AU
Liliaceae	*Asparagus*	*Asparagus cochinchinensis*	Perennial herbs	Flower	White	SU		
Liliaceae	*Asparagus*	*Asparagus longiflorus*	Perennial herbs	Flower、Fruit	Purple	SU	Red	
Liliaceae	*Liriope*	*Liriope muscari*	Perennial herbs	Flower、Fruit	Purple	SU	Purple	SU
Liliaceae	*Clintonia*	*Clintonia udensis*	Perennial herbs	Flower、Fruit	White	SP	Multicolour(Fruit shape)	AU
Liliaceae	*Barnardia*	*Barnardia japonica*	Perennial herbs	Flower	Purple	SU		
Liliaceae	*Maianthemum*	*Maianthemum tubiferum*	Perennial herbs	Flower、Fruit	White	SU	Multicolour(Fruit shape)	AU
Liliaceae	*Veratrum*	*Veratrum nigrum*	Perennial herbs	Flower	Red	SU		
Liliaceae	*Notholirion*	*Notholirion bulbuliferum*	Perennial herbs	Flower	Purple	SU	Multicolour(Frui	AU

								t shape)	
Liliaceae	*Polygonatum*	*Polygonatum cirrhifolium*	Perennial herbs	Flower	Multicolour	SU			
Liliaceae	*Polygonatum*	*Polygonatum verticillatum*	Perennial herbs	Flower、Fruit	Purple	SU		Red	AU
Liliaceae	*Polygonatum*	*Polygonatum zanlanscianense*	Perennial herbs	Flower、Fruit	White	SU		Purple	AU
Liliaceae	*Aletris*	*Aletris alpestris*	Perennial herbs	Flower	White	SU			
Liliaceae	*Aletris*	*Aletris glabra*	Perennial herbs	Flower	White	SU			
Liliaceae	*Eremurus*	*Eremurus chinensis*	Perennial herbs	Flower	White	SU			
Liliaceae	*Cardiocrinum*	*Cardiocrinum giganteum var. yunnanense*	Perennial herbs	Flower	White	SU		Multicolour(Fruit shape)	AU
Liliaceae	*Cardiocrinum*	*Cardiocrinum giganteum*	Perennial herbs	Flower	White	SU		Multicolour(Fruit shape)	AU
Liliaceae	*Allium*	*Allium macranthum*	Perennial herbs	Flower	Purple	AU			
Liliaceae	*Fritillaria*	*Fritillaria taipaiensis*	Perennial herbs	Flower	Green	SP		Multicolour(Fruit shape)	SU
Liliaceae	*Fritillaria*	*Fritillaria cirrhosa*	Perennial herbs	Flower	Multicolour	SU		Multicolour(Fruit shape)	SU、AU
Liliaceae	*Lilium*	*Lilium lancifolium*	Perennial herbs	Flower	Red	SU		Multicolour(Fruit shape)	AU
Liliaceae	*Lilium*	*Lilium fargesii*	Perennial herbs	Flower	Green	SU		Multicolour(Fruit shape)	AU
Liliaceae	*Lilium*	*Lilium brownii var. viridulum*	Perennial herbs	Flower	White	SU		Multicolour(Fruit shape)	SU
Liliaceae	*Lilium*	*Lilium leichtlinii var. maximowiczii*	Perennial herbs	Flower	Yellow	AU			
Liliaceae	*Lilium*	*Lilium brownii*	Perennial herbs	Flower	Multicolo	SU			

					ur			
Liliaceae	*Lilium*	*Lilium davidii*	Perennial herbs	Flower	Yellow	SU		
Liliaceae	*Lilium*	*Lilium duchartrei*	Perennial herbs	Flower	Purple	SU		
Liliaceae	*Lilium*	*Lilium leucanthum*	Perennial herbs	Flower	White	SU		
Liliaceae	*Lilium*	*Lilium regale*	Perennial herbs	Flower	White	SU		
Liliaceae	*Lilium*	*Lilium pumilum*	Perennial herbs	Flower	Red	SU		
Liliaceae	*Smilax*	*Smilax china*	Evergreen shrub	Flower、Fruit	Green	SP	Red	AU
Liliaceae	*Smilax*	*Smilax menispermoidea*	Evergreen shrub	Flower、Fruit	Purple	SU	Black	AU
Liliaceae	*Smilax*	*Smilax discotis*	Evergreen shrub	Flower、Fruit	Green	SP	Black	AU
Liliaceae	*Smilax*	*Smilax polycolea*	Deciduous shrub	Flower、Fruit	Green	SP	Red	AU
Liliaceae	*Smilax*	*Smilax trachypoda*	Deciduous shrub	Flower、Fruit	Green	SP	Black	AU
Liliaceae	*Smilax*	*Smilax stans*	Deciduous shrub	Flower、Fruit	Green	SP	Black	AU
Liliaceae	*Smilax*	*Smilax ferox*	Evergreen shrub	Flower、Fruit	Green	SP	Red	AU
Liliaceae	*Smilax*	*Smilax scobinicaulis*	Evergreen shrub	Flower、Fruit	Green	SP	Black	AU
Liliaceae	*Smilax*	*Smilax lanceifolia*	Evergreen shrub	Flower、Fruit	Green	AU	Black	AU
Liliaceae	*Smilax*	*Smilax megalantha*	Evergreen shrub	Flower、Fruit	White	SP	Red	AU
Liliaceae	*Smilax*	*Smilax japonica*	Evergreen shrub	Flower、Fruit	Green	SU	Black	AU

Liliaceae	*Smilax*	*Smilax nigrescens*	Evergreen shrub	Flower、Fruit	Green	SP	Black	AU
Liliaceae	*Smilax*	*Smilax microphylla*	Evergreen shrub	Flower、Fruit	Green	SU	Black	AU
Liliaceae	*Smilax*	*Smilax glaucochina*	Evergreen shrub	Flower、Fruit	Green	SP	Black	AU
Liliaceae	*Aletris*	*Aletris spicata*	Perennial herbs	Flower	Multicolour	SP	Multicolour(Fruit shape)	SU
Liliaceae	*Aletris*	*Aletris stenoloba*	Perennial herbs	Flower	White	SP、SU	Multicolour(Fruit shape)	SP、SU
Liliaceae	*Hosta*	*Hosta ventricosa*	Perennial herbs	Flower	Purple	SU	Multicolour(Fruit shape)	SU、AU
Liliaceae	*Lloydia*	*Lloydia oxycarpa*	Perennial herbs	Flower	Yellow	SP、SU	Multicolour(Fruit shape)	SU
Liliaceae	*Lloydia*	*Lloydia tibetica*	Perennial herbs	Flower	Yellow	SP、SU		
Liliaceae	*Ophiopogon*	*Ophiopogon bodinieri*	Perennial herbs	Flower、Fruit	Multicolour	SU	Purple	SU、AU
Liliaceae	*Ophiopogon*	*Ophiopogon intermedius*	Perennial herbs	Flower、Fruit	Multicolour	SP、SU	Purple	SU、AU
Liliaceae	*Polygonatum*	*Polygonatum franchetii*	Perennial herbs	Flower	Green	SP、SU	Multicolour(Fruit shape)	AU
Liliaceae	*Polygonatum*	*Polygonatum gracile*	Perennial herbs	Flower	Yellow	SU	Multicolour(Fruit shape)	SU
Liliaceae	*Polygonatum*	*Polygonatum megaphyllum*	Perennial herbs	Flower	Green	SP、SU		
Linaceae	*Linum*	*Linum stelleroides*	Annual and biennial herbs	Flower	Multicolour	SU、AU	Multicolour(Fruit shape)	AU
Linaceae	*Linum*	*Linum usitatissimum*	Annual herbs	Flower	Multicolo	SU	Multicolour(Frui	SU、AU

					ur		t shape)	
Loranthaceae	*Viscum*	*Viscum coloratum*	Deciduous shrub	Flower	Yellow	SU	Red	AU
Lythraceae	*Lagerstroemia*	*Lagerstroemia villosa*	Deciduous tree	Flower	Purple	AU		
Lythraceae	*Lagerstroemia*	*Lagerstroemia excelsa*	Deciduous tree	Flower	Yellow	SP		
Lythraceae	*Lagerstroemia*	*Lagerstroemia subcostata*	Deciduous tree	Flower	White	SU		
Lythraceae	*Lythrum*	*Lythrum salicaria*	Perennial herbs	Flower	Purple	SU		
Magnoliaceae	*Magnolia*	*Yulania multiflora*	Deciduous tree	Flower	White	SP	Multicolour(Fruit shape)	SU
Magnoliaceae	*Magnolia*	*Yulania sprengeri*	Deciduous tree	Flower	White	SP	Multicolour(Fruit shape)	SU
Magnoliaceae	*Magnolia*	*Yulania viridula*	Deciduous tree	Flower	White	SP	Multicolour(Fruit shape)	SU
Magnoliaceae	*Magnolia*	*Magnolia biondii*	Deciduous tree	Flower	White	SP	Multicolour(Fruit shape)	SU
Magnoliaceae	*Magnolia*	*Yulania denudata*	Deciduous tree	Flower	White	SP	Multicolour(Fruit shape)	SU
Magnoliaceae	*Magnolia*	*Yulania liliiflora*	Deciduous shrub	Flower	Purple	SP	Multicolour(Fruit shape)	SU
Magnoliaceae	*Liriodendron*	*Liriodendron chinense*	Deciduous tree	Flower、Fruit	Yellow	SU	Multicolour(Fruit shape)	AU
Magnoliaceae	*Illicium*	*Illicium henryi*	Evergreen shrub	Flower	Red	SP		
Malvaceae	*Corchoropsis*	*Corchoropsis crenata*	Annual herbs	Flower	Yellow	SU	Multicolour(Fruit shape)	AU
Malvaceae	*Abelmosc*	*Abelmoschus manihot*	Annual herbs	Flower	Yellow	SU		

	hus							
Malvaceae	*Abutilon*	*Abutilon theophrasti*	Annual herbs	Flower、Fruit	Yellow	SU		
Malvaceae	*Hibiscus*	*Hibiscus leviseminus*	Deciduous shrub	Flower	Pink	SU		
Malvaceae	*Malva*	*Malva verticillata*	Annual and biennial herbs	Flower、Fruit	Purple	SU	Multicolour(Fruit shape)	AU
Malvaceae	*Malva*	*Malva pusilla*	Perennial herbs	Flower	Multicolour	SP、SU	Multicolour(Fruit shape)	AU
Malvaceae	*Malva*	*Malva cathayensis*	Perennial herbs	Flower	Multicolour	SP、SU		
Malvaceae	*Grewia*	*Grewia biloba var. parviflora*	Deciduous shrub	Flower、Fruit	Green	SP	Red	AU
Mazaceae	*Mazus*	*Mazus stachydifolius*	Perennial herbs	Flower	Purple	SP	Multicolour(Fruit shape)	SU
Melanthiaceae	*Paris*	*Paris polyphylla*	Perennial herbs	Flower、Fruit	Green	SP	Multicolour(Fruit shape)	AU
Melanthiaceae	*Paris*	*Paris fargesii var. petiolata*	Perennial herbs	Flower	Yellow	SU		
Melanthiaceae	*Paris*	*Paris verticillata*	Perennial herbs	Flower	Green	SU		
Meliaceae	*Toona*	*Toona sinensis*	Deciduous tree	Flower	White	SU	Multicolour(Fruit shape)	AU
Meliaceae	*Melia*	*Melia azedarach*	Deciduous tree	Flower	Purple	SP	Yellow	AU
Menispermaceae	*Cocculus*	*Cocculus orbiculatus var. mollis*	Deciduous liana	Flower	Green	SP	Red	AU
Menispermaceae	*Cocculus*	*Cocculus orbiculatus*	Deciduous liana	Flower	Green	SP	Red	AU
Menisperma	*Cyclea*	*Cyclea racemosa*	Deciduous liana	Flower	Green	SP	Red	SU

ceae								
Menispermaceae	*Sinomenium*	*Sinomenium acutum*	Deciduous liana	Flower、Fruit	Green	SU	Red	AU
Nyctaginaceae	*Mirabilis*	*Mirabilis jalapa*	Annual herbs	Flower	Multicolour	SU、AU	Black	SU、AU
Nyctaginaceae	*Oxybaphus*	*Oxybaphus himalaicus var. chinensis*	Annual herbs	Flower	Purple	SU、AU	Black	SU、AU
Nymphaeaceae	*Nelumbo*	*Nelumbo nucifera*	Perennial herbs	Flower	Multicolour	SU	Brown	SU、AU
Olacaceae	*Schoepfia*	*Schoepfia jasminodora*	Deciduous tree	Flower、Fruit	White	SP	Red	SU
Oleaceae	*Jasminum*	*Jasminum nudiflorum*	Deciduous shrub	Flower	Yellow	SP		
Oleaceae	*Jasminum*	*Jasminum lanceolarium*	Evergreen liana	Flower	White	SP	Yellow	SU
Oleaceae	*Jasminum*	*Chrysojasminum floridum*	Evergreen shrub	Flower	Yellow	WI、SP、SU		
Oleaceae	*Ligustrum*	*Ligustrum strongylophyllum*	Evergreen shrub	Flower	White	SU	Black	AU
Oleaceae	*Ligustrum*	*Ligustrum leucanthum*	Deciduous shrub	Flower	Green	SU	Black	AU
Oleaceae	*Ligustrum*	*Ligustrum henryi*	Evergreen shrub	Flower	White	SU	Black	SU
Oleaceae	*Ligustrum*	*Ligustrum quihoui*	Deciduous shrub	Flower	White	SU	Black	AU
Oleaceae	*Ligustrum*	*Ligustrum compactum*	Evergreen shrub	Flower	White	SP	Black	AU
Oleaceae	*Ligustrum*	*Ligustrum sinense*	Deciduous shrub	Flower	White	SP	Black	AU
Oleaceae	*Ligustrum*	*Ligustrum lucidum*	Evergreen tree	Flower	White	SU	Black	AU
Oleaceae	*Osmanthus*	*Osmanthus venosus*	Evergreen shrub	Flower	White	AU		
Oleaceae	*Chionanthus*	*Chionanthus retusus*	Deciduous shrub	Flower	White	SP	Black	SU
Oleaceae	*Forsythia*	*Forsythia giraldiana*	Deciduous shrub	Flower	Yellow	SP		

Oleaceae	*Forsythia*	*Forsythia suspensa*	Deciduous shrub	Flower	Yellow	SP		
Oleaceae	*Forsythia*	*Forsythia viridissima*	Deciduous shrub	Flower	Yellow	SP		
Oleaceae	*Forsythia*	*Forsythia mira*	Deciduous shrub	Flower	Yellow	SP		
Oleaceae	*Syringa*	*Syringa villosa*	Deciduous shrub	Flower	Purple	SU		
Oleaceae	*Syringa*	*Syringa sweginzowii*	Deciduous shrub	Flower	Purple	SU		
Oleaceae	*Syringa*	*Syringa pinnatifolia*	Deciduous shrub	Flower	White	SU		
Oleaceae	*Syringa*	*Syringa pubescens*	Deciduous shrub	Flower	Purple	SU		
Oleaceae	*Syringa*	*Syringa pubescens subsp. julianae*	Deciduous shrub	Flower	Purple	SU		
Oleaceae	*Syringa*	*Syringa reticulata subsp. pekinensis*	Deciduous shrub	Flower	White	SU		
Oleaceae	*Syringa*	*Syringa komarowii*	Deciduous shrub	Flower	Purple	SU		
Oleaceae	*Syringa*	*Syringa oblata*	Deciduous shrub	Flower	Purple	SP		
Oleaceae	*Fraxinus*	*Fraxinus paxiana*	Deciduous tree	Flower、Fruit	White	SU	Multicolour(Fruit shape)	AU
Oleaceae	*Fraxinus*	*Fraxinus mandshurica*	Deciduous tree	Flower、Fruit	Yellow	SP	Multicolour(Fruit shape)	AU
Oleaceae	*Fraxinus*	*Fraxinus chinensis*	Deciduous tree	Flower、Fruit	Yellow	SP	Multicolour(Fruit shape)	SU
Oleaceae	*Fraxinus*	*Fraxinus chinensis subsp. rhynchophylla*	Deciduous tree	Flower、Fruit	Yellow	SP	Multicolour(Fruit shape)	AU
Oleaceae	*Fraxinus*	*Fraxinus odontocalyx*	Deciduous tree	Flower、Fruit	Yellow	SP	Multicolour(Fruit shape)	AU
Oleaceae	*Fraxinus*	*Fraxinus insularis*	Deciduous tree	Flower、Fruit	White	SP	Multicolour(Fruit shape)	SU
Oleaceae	*Fraxinus*	*Fraxinus baroniana*	Deciduous tree	Flower、	Yellow	SP	Multicolour(Frui	SU

				Fruit			t shape)	
Oleaceae	*Fraxinus*	*Fraxinus depauperata*	Deciduous tree	Flower、Fruit	Yellow	SP	Multicolour(Fruit shape)	SU
Onagraceae	*Epilobium*	*Epilobium palustre*	Perennial herbs	Flower、Fruit	Multicolour	SU	Multicolour(Fruit shape)	AU
Onagraceae	*Epilobium*	*Epilobium hirsutum*	Perennial herbs	Flower	Purple	SU	Multicolour(Fruit shape)	AU
Onagraceae	*Epilobium*	*Chamerion angustifolium*	Perennial herbs	Flower	Multicolour	SU	Multicolour(Fruit shape)	AU
Onagraceae	*Circaea*	*Circaea alpina subsp. imaicola*	Perennial herbs	Flower	White	SU、AU	Multicolour(Fruit shape)	SU、AU
Onagraceae	*Circaea*	*Circaea cordata*	Perennial herbs	Flower	White	SU	Multicolour(Fruit shape)	SU、AU
Onagraceae	*Circaea*	*Circaea erubescens*	Perennial herbs	Flower	Pink	SU、AU	Multicolour(Fruit shape)	SU、AU
Onagraceae	*Circaea*	*Circaea mollis*	Perennial herbs	Flower	White	SU、AU	Multicolour(Fruit shape)	SU、AU
Onagraceae	*Epilobium*	*Epilobium amurense subsp. cephalostigma*	Perennial herbs	Flower	Purple	SU、AU	Multicolour(Fruit shape)	AU
Onagraceae	*Epilobium*	*Chamerion angustifolium subsp. circumvagum*	Perennial herbs	Flower	Purple	SU、AU	Multicolour(Fruit shape)	SU、AU
Onagraceae	*Epilobium*	*Chamerion conspersum*	Perennial herbs	Flower	Purple	SU、AU	Multicolour(Fruit shape)	AU
Onagraceae	*Epilobium*	*Epilobium fastigiatoramosum*	Perennial herbs	Flower	White	SU	Multicolour(Fruit shape)	AU
Onagraceae	*Epilobium*	*Epilobium minutiflorum*	Perennial herbs	Flower	Multicolour	SU	Multicolour(Fruit shape)	SU、AU
Onagraceae	*Epilobium*	*Epilobium parviflorum*	Perennial herbs	Flower	Multicolo	SU、AU	Multicolour(Frui	SU、AU

					ur		t shape)	
Onagraceae	*Epilobium*	*Epilobium pyrricholophum*	Perennial herbs	Flower	Pink	SU、AU	Multicolour(Fruit shape)	SU、AU
Onagraceae	*Ludwigia*	*Ludwigia epilobioides*	Annual and biennial herbs	Flower	Yellow	SU、AU	Brown	AU
Orchidaceae	*Chloranthus*	*Chloranthus japonicus*	Perennial herbs	Flower	White	SP	Multicolour(Fruit shape)	SU
Orchidaceae	*Calanthe*	*Calanthe alpina*	Perennial herbs	Flower	Purple	SU	Multicolour(Fruit shape)	AU
Orchidaceae	*Calanthe*	*Calanthe davidii*	Perennial herbs	Flower	Green	SU	Multicolour(Fruit shape)	AU
Orchidaceae	*Calanthe*	*Calanthe tricarinata*	Perennial herbs	Flower	Pink	SP、SU		
Orchidaceae	*Gastrodia*	*Gastrodia elata*	Annual herbs	Flower	Multicolour	SP、SU	Multicolour(Fruit shape)	SP、SU
Orchidaceae	*Spiranthes*	*Spiranthes sinensis*	Perennial herbs	Flower	Purple	SU		
Orchidaceae	*Gymnadenia*	*Gymnadenia conopsea*	Perennial herbs	Flower	Multicolour	SU		
Orchidaceae	*Platanthera*	*Platanthera souliei*	Perennial herbs	Flower	Green	SU	Multicolour(Fruit shape)	AU
Orchidaceae	*Platanthera*	*Platanthera japonica*	Perennial herbs	Flower	White	SP、SU		
Orchidaceae	*Corallorhiza*	*Corallorhiza trifida*	Annual herbs	Flower	Multicolour	SU	Multicolour(Fruit shape)	SU
Orchidaceae	*Cymbidium*	*Cymbidium faberi*	Perennial herbs	Flower	Yellow	SP		
Orchidaceae	*Cymbidium*	*Cymbidium goeringii*	Perennial herbs	Flower	Multicolour	WI、SP		
Orchidaceae	*Herminium*	*Herminium monorchis*	Perennial herbs	Flower	Green	SU		
Orchid	*Epipa*	*Epipactis maire*	Perennia	Flowe	Gree	SU	Multicol	AU

aceae	*ctis*		l herbs	r	n		our(Fruit shape)	
Orchidaceae	*Orchis*	*Ponerorchis chusua*	Perennial herbs	Flower	Purple	SU		
Orchidaceae	*Cremastra*	*Cremastra appendiculata*	Perennial herbs	Flower	Purple	SP、SU	Multicolour(Fruit shape)	AU、WI
Orchidaceae	*Pleione*	*Pleione bulbocodioides*	Perennial herbs	Flower	Purple	SP		
Orchidaceae	*Cypripedium*	*Cypripedium franchetii*	Perennial herbs	Flower	Purple	SP、SU		
Orchidaceae	*Cypripedium*	*Cypripedium japonicum*	Perennial herbs	Flower、Fruit	Green	SP	Multicolour(Fruit shape)	SU
Orchidaceae	*Goodyera*	*Goodyera schlechtendaliana*	Perennial herbs	Flower	White	SU		
Orchidaceae	*Bletilla*	*Bletilla striata*	Perennial herbs	Flower	Pink	SP		
Orchidaceae	*Amitostigma*	*Amitostigma monanthum*	Perennial herbs	Flower	Multicolour	SU		
Orchidaceae	*Calanthe*	*Calanthe arcuata*	Perennial herbs	Flower	Multicolour	SU、AU		
Orchidaceae	*Cephalanthera*	*Cephalanthera erecta*	Perennial herbs	Flower	White	SP、SU	Multicolour(Fruit shape)	SU、AU
Orchidaceae	*Coeloglossum*	*Dactylorhiza viridis*	Perennial herbs	Flower	Green	SU	Multicolour(Fruit shape)	AU
Orchidaceae	*Goodyera*	*Goodyera repens*	Perennial herbs	Flower	Multicolour	SU		
Orchidaceae	*Herminium*	*Herminium lanceum*	Perennial herbs	Flower	Green	SU		
Orchidaceae	*Holopogon*	*Holopogon smithianus*	Perennial herbs	Flower	Green	SU、AU		
Orchidaceae	*Neottia*	*Neottia acuminata*	Annual herbs	Flower	Yellow	SU	Multicolour(Fruit shape)	SU
Orchidaceae	*Neottianthe*	*Neottianthe cucullata*	Perennial herbs	Flower	Purple	SU、AU		

Orchidaceae	*Orchis*	*Galearis spathulata*	Perennial herbs	Flower	Purple	SU		
Orchidaceae	*Platanthera*	*Platanthera minutiflora*	Perennial herbs	Flower	Green	SU		
Orobanchaceae	*Siphonostegia*	*Siphonostegia chinensis*	Annual herbs	Flower	Yellow	SU		
Orobanchaceae	*Euphrasia*	*Euphrasia regelii*	Annual herbs	Flower	White	SP、SU		
Orobanchaceae	*Pedicularis*	*Pedicularis resupinata*	Perennial herbs	Flower、Fruit	Multicolour	SU	Multicolour(Fruit shape)	AU
Orobanchaceae	*Pedicularis*	*Pedicularis verticillata*	Perennial herbs	Flower	Purple	SU		
Orobanchaceae	*Pedicularis*	*Pedicularis spicata*	Annual herbs	Flower	Red	SU	Multicolour(Fruit shape)	AU
Orobanchaceae	*Pedicularis*	*Pedicularis muscicola*	Perennial herbs	Flower	Red	SP	Multicolour(Fruit shape)	SU
Orobanchaceae	*Pedicularis*	*Pedicularis decora*	Perennial herbs	Flower	Yellow	SP		
Orobanchaceae	*Pedicularis*	*Pedicularis rhinanthoides subsp. labellata*	Perennial herbs	Flower	Red	SU		
Orobanchaceae	*Phacellanthus*	*Phacellanthus tubiflorus*	Annual herbs	Flower	Yellow	SP	Multicolour(Fruit shape)	SU
Oxalidaceae	*Oxalis*	*Oxalis corniculata*	Perennial herbs	Flower	Yellow	SP、SU		
Oxalidaceae	*Oxalis*	*Oxalis acetosella*	Perennial herbs	Flower、Fruit	White	SU	Multicolour(Fruit shape)	AU
Oxalidaceae	*Oxalis*	*Oxalis griffithii*	Perennial herbs	Flower	White	AU		
Paeoniaceae	*Paeonia*	*Paeonia jishanensis*	Deciduous shrub	Flower	Multicolour	SP		
Paeoniaceae	*Paeonia*	*Paeonia rockii*	Deciduous shrub	Flower	White	SP		

Paeoniaceae	*Paeonia*	*Paeonia rockii subsp. atava*	Deciduous shrub	Flower	White	SP		
Paeoniaceae	*Paeonia*	*Paeonia lactiflora*	Perennial herbs	Flower	White	SP、SU	Multicolour(Fruit shape)	SU
Paeoniaceae	*Paeonia*	*Paeonia anomala subsp. veitchii*	Perennial herbs	Flower	Red	SU	Multicolour(Fruit shape)	AU
Papaveraceae	*Corydalis*	*Corydalis racemosa*	Perennial herbs	Flower	Yellow	SP		
Papaveraceae	*Corydalis*	*Corydalis ophiocarpa*	Perennial herbs	Flower、Fruit	White	SP	Multicolour(Fruit shape)	SU
Papaveraceae	*Corydalis*	*Corydalis edulis*	Annual herbs	Flower、Fruit	Purple	SP	Multicolour(Fruit shape)	SU
Papaveraceae	*Corydalis*	*Corydalis trisecta*	Perennial herbs	Flower、Fruit	Yellow	SU	Multicolour(Fruit shape)	SU
Papaveraceae	*Corydalis*	*Corydalis shensiana*	Perennial herbs	Flower、Fruit	Purple	SU	Multicolour(Fruit shape)	SU
Papaveraceae	*Papaver*	*Papaver nudicaule*	Perennial herbs	Flower	Multicolour	SU		
Papaveraceae	*Dicranostigma*	*Dicranostigma leptopodum*	Perennial herbs	Flower	Yellow	SP	Multicolour(Fruit shape)	SU
Papaveraceae	*Meconopsis*	*Meconopsis quintuplinervia*	Perennial herbs	Flower	Purple	SU、AU	Multicolour(Fruit shape)	SU、AU
Papaveraceae	*Meconopsis*	*Meconopsis oliveriana*	Perennial herbs	Flower	Yellow	SP、SU	Multicolour(Fruit shape)	SU、AU
Papaveraceae	*Papaver*	*Stylophorum lasiocarpum*	Perennial herbs	Flower	Yellow	SU		
Papaveraceae	*Hypecoum*	*Hypecoum leptocarpum*	Annual herbs	Flower	Purple	SU	Multicolour(Fruit shape)	SU、AU
Papaveraceae	*Hypecoum*	*Hypecoum erectum*	Annual herbs	Flower	Yellow	SP	Multicolour(Fruit shape)	SU

Papaveraceae	*Hylomecon*	*Hylomecon japonica*	Perennial herbs	Flower	Yellow	SP	Multicolour(Fruit shape)	SU
Papaveraceae	*Hylomecon*	*Hylomecon japonia*	Perennial herbs	Flower	Yellow	SP	Multicolour(Fruit shape)	SU
Papaveraceae	*Macleaya*	*Macleaya microcarpa*	Perennial herbs	Flower、Fruit	Yellow	SU	Multicolour(Fruit shape)	AU
Papaveraceae	*Macleaya*	*Macleaya cordata*	Perennial herbs	Flower、Fruit	Yellow	SU	Multicolour(Fruit shape)	AU
Papaveraceae	*Chelidonium*	*Chelidonium majus*	Perennial herbs	Flower	Yellow	SP、SU	Multicolour(Fruit shape)	SU
Papaveraceae	*Corydalis*	*Corydalis acuminata*	Perennial herbs	Flower	Purple	SP、SU	Multicolour(Fruit shape)	SP、SU
Papaveraceae	*Corydalis*	*Corydalis adunca*	Perennial herbs	Flower	Yellow			
Papaveraceae	*Corydalis*	*Corydalis fargesii*	Perennial herbs	Flower	Yellow	SU、AU	Multicolour(Fruit shape)	SU、AU
Papaveraceae	*Corydalis*	*Corydalis gamosepala*	Perennial herbs	Flower	Purple			
Papaveraceae	*Corydalis*	*Corydalis giraldii*	Perennial herbs	Flower	Yellow			
Papaveraceae	*Corydalis*	*Corydalis linarioides*	Perennial herbs	Flower	Yellow	SU、AU	Multicolour(Fruit shape)	SU、AU
Papaveraceae	*Hylomecon*	*Hylomecon japonica var. subincisa*	Perennial herbs	Flower	Yellow			
Paulowniaceae	*Paulownia*	*Paulownia tomentosa*	Deciduous tree	Flower	Purple	SP	Multicolour(Fruit shape)	AU
Pedaliaceae	*Sesamum*	*Sesamum indicum*	Annual herbs	Flower、Fruit	White	SU	Multicolour(Fruit shape)	AU
Pentaphylac	*Eurya*	*Eurya nitida*	Evergreen shrub	Flower	White	AU	Black	SU

aceae								
Pentaphylacaceae	*Eurya*	*Eurya japonica*	Evergreen shrub	Flower	White	SP	Black	AU
Pentaphylacaceae	*Eurya*	*Eurya brevistyla*	Evergreen shrub	Flower	White	AU	Black	SU
Phrymaceae	*Alkekengi*	*Mimulus szechuanensis*	Perennial herbs	Flower	Yellow	SU		
Phyllanthaceae	*Bischofia*	*Bischofia javanica*	Evergreen tree	Flower	Green	SP		
Phyllanthaceae	*Fluegea*	*Flueggea suffruticosa*	Deciduous shrub	Flower	Yellow	SP		
Phytolaccaceae	*Phytolacca*	*Phytolacca acinosa*	Perennial herbs	Fruit	Multicolour	SP、SU	Black	SU、AU
Pittosporaceae	*Pittosporum*	*Pittosporum podocarpum var. angustatum*	Evergreen shrub	Flower	Yellow	SP		
Pittosporaceae	*Pittosporum*	*Pittosporum heterophyllum*	Evergreen shrub	Flower	Yellow	SP		
Pittosporaceae	*Pittosporum*	*Pittosporum podocarpum*	Evergreen shrub	Flower	Yellow	SP		
Pittosporaceae	*Pittosporum*	*Pittosporum rehderianum*	Evergreen shrub	Flower	Yellow	SP		
Pittosporaceae	*Pittosporum*	*Pittosporum truncatum*	Evergreen shrub	Flower	Yellow	SP		
Plantaginaceae	*Veronica*	*Veronica serpyllifolia*	Perennial herbs	Flower	Multicolour	SP		
Plantaginaceae	*Veronica*	*Veronica polita Fries*	Annual and biennial herbs	Flower	Multicolour	SP、SU、AU		
Plantaginaceae	*Veronicastrum*	*Veronicastrum sibiricum*	Perennial herbs	Flower	Purple	SU	Multicolour(Fruit shape)	AU
Planta	*Hydra*	*Hemiphragma*	Perennia	Flowe	Red		Multicol	SU

ginaceae	*ngea*	*heterophyllum*	l herbs	r 、Fruit			our(Fruit shape)	
Plumbaginaceae	*Limonium*	*Limonium bicolor*	Perennial herbs	Flower	Yellow	SP	Multicolour(Fruit shape)	SU
Poaceae	*Poa*	*Poa sphondylodes*	Perennial herbs	观叶				
Poaceae	*Poa*	*Poa pratensis*	Perennial herbs	观叶				
Poaceae	*Phalaris*	*Phalaris arundinacea*	Perennial herbs	Flower		SU	Multicolour(Fruit shape)	SU
Poaceae	*Phaenosperma*	*Phaenosperma globosa*	Perennial herbs	Flower	White	SP、SU	Multicolour(Fruit shape)	SU
Poaceae	*Glyceria*	*Glyceria leptolepis*	Perennial herbs	Flower		SU	Brown	SU、AU
Poaceae	*Bromus*	*Bromus japonicus*	Annual herbs	Fruit			Multicolour(Fruit shape)	SU
Poaceae	*Miscanthus*	*Miscanthus sinensis*	Perennial herbs	Flower 、Fruit	Yellow	SU、AU	Multicolour(Fruit shape)	AU
Poaceae	*Phragmites*	*Phragmites australis*	Perennial herbs	观叶				
Poaceae	*Pennisetum*	*Pennisetum alopecuroides*	Perennial herbs	Flower 、Fruit		SU、AU	Multicolour(Fruit shape)	SU、AU
Poaceae	*Eragrostis*	*Eragrostis cilianensis*	Annual herbs	Flower 、Fruit	White	SU、AU	Multicolour(Fruit shape)	SU、AU
Poaceae	*Eragrostis*	*Eragrostis minor*	Annual herbs	Fruit			Multicolour(Fruit shape)	AU
Poaceae	*Setaria*	*Setaria viridis*	Annual herbs	Flower	Green	SP、SU	Multicolour(Fruit shape)	AU
Poaceae	*Saccharum*	*Saccharum arundinaceum*	Perennial herbs	Flower 、Fruit	Green	SU、AU	Multicolour(Fruit shape)	AU
Poaceae	*Deschampsia*	*Deschampsia cespitosa*	Perennial herbs	Flower 、Fruit		SU、AU	Multicolour(Fruit shape)	SU、AU
Poace	*Triarr*	*Miscanthus*	Perennia	Flowe		SU、AU	Multicol	SU、AU

ae	*hena*	*sacchariflorus*	l herbs	r、Fruit			our(Fruit shape)	
Poaceae	*Koeleria*	*Koeleria macrantha*	Perennial herbs	观叶				
Poaceae	*Imperata*	*Imperata cylindrica*	Perennial herbs	观叶				
Polemoniaceae	*Polemonium*	*Polemonium chinense*	Perennial herbs	Flower	Purple	SU	Multicolour(Fruit shape)	AU
Polygalaceae	*Polygala*	*Polygala arillata*	Evergreen shrub	Flower	Yellow	SU	Red	SU
Polygalaceae	*Polygala*	*Polygala tenuifolia*	Perennial herbs	Flower、Fruit	Purple	SU	Multicolour(Fruit shape)	AU
Polygalaceae	*Polygala*	*Polygala tatarinowii*	Annual herbs	Flower	Purple	SU	Multicolour(Fruit shape)	AU
Polygalaceae	*Polygala*	*Polygala sibirica*	Perennial herbs	Flower	Purple	SP	Multicolour(Fruit shape)	SU
Polygalaceae	*Polygala*	*Polygala japonica*	Perennial herbs	Flower	Purple	SP	Multicolour(Fruit shape)	SU
Polygonaceae	*Pteroxygonum*	*Pteroxygonum giraldii*	Perennial herbs	Flower	White	SU	Multicolour(Fruit shape)	AU
Polygonaceae	*Rumex*	*Rumex acetosa*	Perennial herbs	Flower	Green	SU		
Polygonaceae	*Oxyria*	*Oxyria digyna*	Perennial herbs	Flower、Fruit	Red	SU	Multicolour(Fruit shape)	AU
Polygonaceae	*Polygonum*	*Polygonum bistorta*	Perennial herbs	Flower	Multicolour	SU	Multicolour(Fruit shape)	AU
Polygonaceae	*Fagopyrum*	*Fagopyrum dibotrys*	Perennial herbs	Flower	White	SU	Multicolour(Fruit shape)	AU
Polygonaceae	*Polygonum*	*Persicaria perfoliata*	Annual herbs	Fruit			Black	SU
Polygonaceae	*Polygonum*	*Persicaria orientalis*	Annual herbs	Flower、Fruit	Multicolour	SU	Brown	AU

Polygonaceae	*Polygonum*	*Persicaria amphibia*	Perennial herbs	Flower	Multicolour	SU	Multicolour(Fruit shape)	SU
Polygonaceae	*Polygonum*	*Persicaria japonica*	Perennial herbs	Flower	Multicolour	SU	Multicolour(Fruit shape)	AU
Polygonaceae	*Polygonum*	*Persicaria runcinata var. sinensis*	Annual and biennial herbs	Fruit			Multicolour(Fruit shape)	SU
Polygonaceae	*Polygonum*	*Polygonum nepalense*	Perennial herbs	Flower	Purple	SU		
Polygonaceae	*Polygonum*	*Polygonum lapathifolium*	Annual herbs	Flower	Purple	SU		
Polygonaceae	*Polygonum*	*Polygonum posumbu*	Annual herbs	Flower	White	SU		
Polygonaceae	*Polygonum*	*Polygonum thunbergii*	Annual herbs	Flower	Red	SU		
Polygonaceae	*Polygonum*	*Polygonum amplexicaule var. sinense*	Perennial herbs	观叶				
Polygonaceae	*Antenoron*	*Antenoron filiforme*	Perennial herbs	Flower	Red	SU	Multicolour(Fruit shape)	AU
Polygonaceae	*Reynoutria*	*Reynoutria japonica*	Perennial herbs	Flower、Fruit	Green	SU、AU	Brown	AU
Polygonaceae	*Fallopia*	*Fallopia convolvulus*	Annual herbs	Flower、Fruit	Green	SP	Black	AU
Polygonaceae	*Fallopia*	*Fallopia multiflora*	Perennial herbs	Flower	White	AU		
Polygonaceae	*Rheum*	*Rheum tataricum*	Perennial herbs	Flower	Red	SU		
Polygonaceae	*Rheum*	*Rheum officinale*	Perennial herbs	Flower、Fruit	Multicolour	SP、SU	Multicolour(Fruit shape)	SU、AU
Polygo	*Polyg*	*Polygonum*	Perennia	Flowe	Multi	SU	Multicol	AU

naceae	*onum*	*suffultum*	l herbs	r	colour		our(Fruit shape)	
Polygonaceae	*Polygonum*	*Polygonum viviparum*	Perennial herbs	Flower	Multicolour	SP	Multicolour(Fruit shape)	SU
Polygonaceae	*Polygonum*	*Polygonum sphaerostachyum*	Perennial herbs	Flower	Multicolour	SU	Multicolour(Fruit shape)	AU
Polygonaceae	*Fagopyrum*	*Fagopyrum gracilipes*	Annual herbs	Flower	Pink	SU、AU	Multicolour(Fruit shape)	SU、AU
Polygonaceae	*Fallopia*	*Fallopia multiflora var. ciliinervis*	Perennial herbs	Flower、Fruit	Green	SU、AU	Brown	AU
Polygonaceae	*Polygonum*	*Polygonum dissitiflorum*	Annual herbs	Flower	Pink	SU	Brown	SU、AU
Polygonaceae	*Polygonum*	*Polygonum pinetorum*	Perennial herbs	Flower	Multicolour	SP、SU	Brown	SU、AU
Polygonaceae	*Polygonum*	*Polygonum sparsipilosum*	Annual herbs	Flower	White	SU	Brown	SU、AU
Polygonaceae	*Polygonum*	*Polygonum sparsipilosum var. hubertii*	Annual herbs	Flower	White	SU	Brown	SU、AU
Polygonaceae	*Rumex*	*Rumex dentatus*	Annual herbs	Flower	Green	SP、SU	Brown	SU
Polygonaceae	*Rumex*	*Rumex patientia*	Perennial herbs	Flower	Green	SP、SU	Brown	SU
Polypodiaceae	*Neocheiropteris*	*Neocheiropteris palmatopedata*	Perennial herbs	观叶				
Polypodiaceae	*Lepisorus*	*Lepisorus marginatus*	Annual herbs	观叶				
Pontederiaceae	*Monochoria*	*Monochoria korsakowii*	Perennial herbs	Flower	Purple	SU	Multicolour(Fruit shape)	AU
Pontederiaceae	*Monochoria*	*Monochoria vaginalis*	Annual herbs	Flower	Purple	SU、AU	Multicolour(Fruit shape)	AU

Portulacaceae	*Portulaca*	*Portulaca oleracea*	Annual herbs	Flower	Yellow	SP、SU	Multicolour(Fruit shape)	SU、AU
Potamogetonaceae	*Potamogeton*	*Potamogeton crispus*	Perennial herbs	Flower	Green	SP、SU	Multicolour(Fruit shape)	SP、SU
Potamogetonaceae	*Potamogeton*	*Potamogeton distinctus*	Perennial herbs	Flower	Green	SP、SU	Multicolour(Fruit shape)	SU、AU
Primulaceae	*Lysimachia*	*Lysimachia pentapetala*	Annual herbs	Flower	White	SU	Multicolour(Fruit shape)	SU
Primulaceae	*Lysimachia*	*Lysimachia hemsleyana*	Perennial herbs	Flower	Yellow	SP	Multicolour(Fruit shape)	SU
Primulaceae	*Lysimachia*	*Lysimachia grammica*	Perennial herbs	Flower	Yellow	SP	Multicolour(Fruit shape)	SU
Primulaceae	*Lysimachia*	*Lysimachia congestiflora*	Perennial herbs	Flower	Yellow	SP	Multicolour(Fruit shape)	AU
Primulaceae	*Lysimachia*	*Lysimachia christiniae*	Perennial herbs	Flower	Yellow	SP	Multicolour(Fruit shape)	AU
Primulaceae	*Lysimachia*	*Lysimachia candida*	Annual and biennial herbs	Flower	White	SP	Multicolour(Fruit shape)	SU
Primulaceae	*Lysimachia*	*Lysimachia barystachys*	Annual and biennial herbs	Flower	White	SP	Multicolour(Fruit shape)	AU
Primulaceae	*Lysimachia*	*Lysimachia clethroides*	Perennial herbs	Flower	White	SU		
Primulaceae	*Cortusa*	*Cortusa matthioli Linnaeus*	Perennial herbs	Flower	Purple	SP	Multicolour(Fruit shape)	SU
Primulaceae	*Cortusa*	*Cortusa matthioli subsp. pekinensis*	Perennial herbs	Flower	Purple	SP		
Primulaceae	*Androsace*	*Androsace umbellata*	Annual and	Flower	White	SP	Multicolour(Frui	SU

			biennial herbs				t shape)	
Primulaceae	*Primula*	*Primula maximowiczii*	Perennial herbs	Flower	Red	SP	Multicolour(Fruit shape)	SU
Primulaceae	*Primula*	*Primula purdomii*	Perennial herbs	Flower	Purple	SU		
Primulaceae	*Primula*	*Primula stenocalyx*	Perennial herbs	Flower	Purple	SP、SU	Multicolour(Fruit shape)	SU、AU
Primulaceae	*Primula*	*Primula odontocalyx*	Perennial herbs	Flower	Purple	SP	Multicolour(Fruit shape)	SU
Primulaceae	*Primula*	*Primula malacoides*	Annual and biennial herbs	Flower	Multicolour	SP		
Primulaceae	*Primula*	*Primula sinensis*	Perennial herbs	Flower	Multicolour	WI	Multicolour(Fruit shape)	WI、SP
Primulaceae	*Primula*	*Primula handeliana*	Perennial herbs	Flower	Yellow	SP、SU	Multicolour(Fruit shape)	SU
Primulaceae	*Primula*	*Primula knuthiana*	Perennial herbs	Flower	Purple	SP、SU	Multicolour(Fruit shape)	SU
Primulaceae	*Primula*	*Primula giraldiana*	Perennial herbs	Flower	Purple	SU		
Primulaceae	*Primula*	*Primula involucrata subsp. yargongensis*	Perennial herbs	Flower	Purple	SP		
Primulaceae	*Androsace*	*Androsace cuscutiformis*	Perennial herbs	Flower、Fruit	White	SP	Multicolour(Fruit shape)	SU
Primulaceae	*Androsace*	*Androsace laxa*	Perennial herbs	Flower	Pink	SU		
Primulaceae	*Primula*	*Primula conspersa*	Perennial herbs	Flower	Purple	SP、SU	Multicolour(Fruit shape)	SU、AU
Primulaceae	*Primula*	*Primula violaris*	Perennial herbs	Flower	Purple	SP、SU		
Primulaceae	*Primula*	*Primula woodwardii*	Perennial herbs	Flower	Multicolo	SU		

					ur			
Pteridiaceae	*Adiantum*	*Adiantum capillus-junonis*	Perennial herbs	观叶				
Pteridiaceae	*Pteridium*	*Drynaria baronii*	Perennial herbs	观叶				
Pteridiaceae	*Pteridium*	*Pteridium aquilinum var. latiusculum*	Perennial herbs	观叶				
Pteridiaceae	*Cyrtomium*	*Cyrtomium fortunei*	Perennial herbs	观叶				
Pteridiaceae	*Cyrtomium*	*Cyrtomium caryotideum*	Perennial herbs	观叶				
Pteridiaceae	*Cyrtomium*	*Cyrtomium yamamotoi*	Perennial herbs	观叶				
Pteridiaceae	*Cyrtomium*	*Cyrtomium tsinglingense*	Perennial herbs	观叶				
Ranunculaceae	*Semiaquilegia*	*Semiaquilegia adoxoides*	Perennial herbs	Flower、Fruit	White	SP	Multicolour(Fruit shape)	SP
Ranunculaceae	*Anemone*	*Anemone tomentosa*	Perennial herbs	Flower	Red	SU、AU		
Ranunculaceae	*Anemone*	*Anemone hupehensis*	Perennial herbs	Flower	Purple	SU、AU		
Ranunculaceae	*Anemone*	*Anemone rivularis*	Perennial herbs	Flower	White	SP、SU		
Ranunculaceae	*Anemone*	*Anemone altaica*	Perennial herbs	Flower	White	SP		
Ranunculaceae	*Anemone*	*Anemone vitifolia*	Annual herbs	Flower	White	SU		
Ranunculaceae	*Anemone*	*Anemone taipaiensis*	Perennial herbs	Flower	White	AU		
Ranunculaceae	*Anemone*	*Anemone begoniifolia*	Perennial herbs	Flower	White	WI		
Ranunculaceae	*Oxygraphis*	*Oxygraphis glacialis*	Perennial herbs	Flower	Yellow	SU	Multicolour(Fruit shape)	SU
Ranun	*Aconi*	*Aconitum*	Perennia	Flowe	Purpl	SU		

culaceae	*tum*	*tanguticum*	l herbs	r	e			
Ranunculaceae	*Aconitum*	*Aconitum pendulum*	Perennial herbs	Flower	Yellow	SU、AU		
Ranunculaceae	*Aconitum*	*Aconitum carmichaelii*	Perennial herbs	Flower	Purple	AU		
Ranunculaceae	*Aconitum*	*Aconitum sinomontanum*	Perennial herbs	Flower	Purple	SU、AU		
Ranunculaceae	*Aconitum*	*Aconitum taipaicum*	Perennial herbs	Flower	Purple	AU		
Ranunculaceae	*Aconitum*	*Aconitum lioui*	Perennial herbs	Flower	Purple	AU		
Ranunculaceae	*Aconitum*	*Aconitum shensiense*	Perennial herbs	Flower	Purple	AU		
Ranunculaceae	*Aconitum*	*Aconitum hemsleyanum*	Perennial herbs	Flower	Purple	AU		
Ranunculaceae	*Aconitum*	*Aconitum kirinense var. australe*	Perennial herbs	Flower、Fruit	White	SU	Multicolour(Fruit shape)	AU
Ranunculaceae	*Aconitum*	*Aconitum sungpanense*	Annual herbs	Flower	Purple	AU		
Ranunculaceae	*Clematis*	*Clematis dasyandra*	Deciduous liana	Flower	White	AU		
Ranunculaceae	*Clematis*	*Clematis macropetala*	Deciduous liana	Flower	Purple	SU		
Ranunculaceae	*Clematis*	*Clematis henryi var. ternata*	Deciduous liana	Flower	White	AU		
Ranunculaceae	*Clematis*	*Clematis potaninii*	Deciduous liana	Flower	White	SU		
Ranunculaceae	*Clematis*	*Clematis finetiana*	Deciduous liana	Flower	White	SP		

Ranunculaceae	*Clematis*	*Clematis montana var. longipes*	Deciduous liana	Flower	White	SU
Ranunculaceae	*Clematis*	*Clematis dingjunshanica*	Deciduous liana	Flower	Purple	AU
Ranunculaceae	*Clematis*	*Clematis apiifolia var. argentilucida*	Deciduous liana	Flower	White	SU
Ranunculaceae	*Clematis*	*Clematis puberula var. ganpiniana*	Deciduous liana	Flower	White	SU
Ranunculaceae	*Clematis*	*Clematis montana*	Deciduous liana	Flower	White	SU
Ranunculaceae	*Clematis*	*Clematis kirilowii*	Deciduous liana	Flower	White	SU
Ranunculaceae	*Clematis*	*Clematis peterae var. trichocarpa*	Deciduous liana	Flower	White	SU
Ranunculaceae	*Clematis*	*Clematis armandii var. farquhariana*	Deciduous liana	Flower	White	SP
Ranunculaceae	*Clematis*	*Clematis henryi*	Deciduous liana	Flower	White	AU
Ranunculaceae	*Clematis*	*Clematis linearifoliola*	Deciduous liana	Flower	Purple	AU
Ranunculaceae	*Clematis*	*Clematis uncinata var. coriacea*	Evergreen liana	Flower	White	SU
Ranunculaceae	*Clematis*	*Clematis gouriana*	Deciduous liana	Flower	White	AU
Ranunculaceae	*Clematis*	*Clematis brevicaudata*	Deciduous liana	Flower	White	SU
Ranunculaceae	*Clematis*	*Clematis armandii*	Evergreen liana	Flower	White	SP
Ranunculace	*Clematis*	*Clematis shensiensis*	Deciduous liana	Flower	White	SU

ae								
Ranunculaceae	*Clematis*	*Clematis uncinata*	Deciduous liana	Flower	White	SU		
Ranunculaceae	*Clematis*	*Clematis peterae*	Deciduous liana	Flower	White	SU		
Ranunculaceae	*Clematis*	*Clematis chinensis*	Deciduous liana	Flower	White	SU		
Ranunculaceae	*Clematis*	*Clematis pashanensis var. latisepala*	Deciduous liana	Flower	White	SU		
Ranunculaceae	*Clematis*	*Clematis grandidentata*	Deciduous liana	Flower	White	SU		
Ranunculaceae	*Clematis*	*Clematis gratopsis*	Deciduous liana	Flower	White	AU		
Ranunculaceae	*Clematis*	*Clematis pashanensis*	Deciduous liana	Flower	White	SU		
Ranunculaceae	*Clematis*	*Clematis puberula var. tenuisepala*	Deciduous liana	Flower	White	SU		
Ranunculaceae	*Clematis*	*Clematis chengguensis*	Deciduous liana	Flower	Pink	SU		
Ranunculaceae	*Clematis*	*Clematis chinensis var. vestita*	Deciduous liana	Flower	White	SU		
Ranunculaceae	*Clematis*	*Clematis shensiensis*	Deciduous liana	Flower	Purple	SU		
Ranunculaceae	*Clematis*	*Clematis hexapetala*	Perennial herbs	Flower	White	SU		
Ranunculaceae	*Clematis*	*Clematis heracleifolia*	Perennial herbs	Flower	Purple	SU、AU		
Ranunculaceae	*Clematis*	*Clematis patens*	Perennial herbs	Flower、Fruit	Multicolour	SP	Multicolour(Fruit shape)	SU
Ranun	*Helle*	*Helleborus*	Perennia	Flowe	Pink	SP	Multicol	SP

culaceae	*borus*	*thibetanus*	l herbs	r			our(Fruit shape)	
Ranunculaceae	*Thalictrum*	*Thalictrum petaloideum*	Perennial herbs	Flower	White	SU		
Ranunculaceae	*Cimicifuga*	*Cimicifuga foetida*	Perennial herbs	Flower、Fruit	White	SU、AU	Multicolour(Fruit shape)	AU
Ranunculaceae	*Cimicifuga*	*Cimicifuga dahurica*	Perennial herbs	Flower、Fruit	White	SU	Multicolour(Fruit shape)	AU
Ranunculaceae	*Cimicifuga*	*Cimicifuga simplex*	Perennial herbs	Flower、Fruit	White	SU	Yellow	AU
Ranunculaceae	*Paeonia*	*Paeonia obovata*	Perennial herbs	Flower	White	SU		
Ranunculaceae	*Callianthemum*	*Callianthemum taipaicum*	Perennial herbs	Flower	White	AU		
Ranunculaceae	*Ranunculus*	*Ranunculus sieboldii*	Perennial herbs	Flower	Yellow	SP、SU		
Ranunculaceae	*Ranunculus*	*Ranunculus japonicus*	Perennial herbs	Flower	Yellow	SP		
Ranunculaceae	*Aquilegia*	*Aquilegia yabeana*	Perennial herbs	Flower	Purple	SP、SU		
Ranunculaceae	*Aquilegia*	*Aquilegia ecalcarata*	Perennial herbs	Flower	Purple	SP、SU		
Ranunculaceae	*Aquilegia*	*Aquilegia incurvata*	Perennial herbs	Flower	Purple	SP、SU		
Ranunculaceae	*Aquilegia*	*Aquilegia viridiflora*	Perennial herbs	Flower	Multicolour	SU		
Ranunculaceae	*Trollius*	*Trollius buddae*	Perennial herbs	Flower	Yellow	SU	Multicolour(Fruit shape)	AU
Ranunculaceae	*Trollius*	*Trollius farreri*	Perennial herbs	Flower	Yellow	SU		

Ranunculaceae	*Souliea*	*Souliea vaginata*	Perennial herbs	Flower	White	SP	Multicolour(Fruit shape)	SU
Ranunculaceae	*Consolida*	*Consolida ajacis*	Perennial herbs	Flower	Purple	SU		
Ranunculaceae	*Kingdonia*	*Kingdonia uniflora*	Perennial herbs	Flower、Fruit	Red	SU		
Ranunculaceae	*Delphinium*	*Delphinium anthriscifolium*	Perennial herbs	Flower	Purple	SP		
Ranunculaceae	*Delphinium*	*Delphinium taipaicum*	Perennial herbs	Flower	Purple	AU		
Ranunculaceae	*Delphinium*	*Delphinium giraldii*	Perennial herbs	Flower	Purple	SU		
Ranunculaceae	*Delphinium*	*Delphinium campylocentrum*	Perennial herbs	Flower	Purple	AU		
Ranunculaceae	*Delphinium*	*Delphinium henryi*	Perennial herbs	Flower	Purple	AU		
Ranunculaceae	*Delphinium*	*Delphinium grandiflorum var. glandulosum*	Perennial herbs	Flower	Purple	AU		
Ranunculaceae	*Adonis*	*Adonis sutchuenensis*	Perennial herbs	Flower	Yellow	SP		
Ranunculaceae	*Pulsatilla*	*Pulsatilla chinensis*	Perennial herbs	Flower	Purple	SP		
Ranunculaceae	*Aconitum*	*Aconitum barbatum var. hispidum*	Perennial herbs	Flower	Green	SU		
Ranunculaceae	*Aconitum*	*Aconitum cannabifolium*	Perennial herbs	Flower	White	AU		
Ranunculaceae	*Aconitum*	*Aconitum scaposum*	Perennial herbs	Flower	Purple	SU、AU		
Ranun	*Aconi*	*Aconitum*	Perennia	Flowe	Multi	SU、AU		

culaceae	*tum*	*scaposum var. hupehanum*	l herbs	r	colour			
Ranunculaceae	*Aconitum*	*Aconitum scaposum var. vaginatum*	Perennial herbs	Flower	Multicolour	SU、AU		
Ranunculaceae	*Actaea*	*Actaea asiatica*	Perennial herbs	Flower、Fruit	White	SP、SU	Black	AU
Ranunculaceae	*Adonis*	*Adonis davidii*	Perennial herbs	Flower	Multicolour	SP、SU		
Ranunculaceae	*Anemone*	*Anemone baicalensis*	Perennial herbs	Flower	White	SP、SU		
Ranunculaceae	*Anemone*	*Anemone exigua*	Perennial herbs	Flower	White	SU		
Ranunculaceae	*Anemone*	*Anemone flaccida*	Perennial herbs	Flower	White	SP、SU		
Ranunculaceae	*Anemone*	*Anemone geum subsp. ovalifolia*	Perennial herbs	Flower	White	SP、SU		
Ranunculaceae	*Anemone*	*Anemone reflexa*	Perennial herbs	Flower	White	SP		
Ranunculaceae	*Anemone*	*Anemone rockii*	Perennial herbs	Flower	White	SU		
Ranunculaceae	*Aquilegia*	*Aquilegia oxysepala var. kansuensis*	Perennial herbs	Flower	Purple	SP、SU	Multicolour(Fruit shape)	SU
Ranunculaceae	*Beesia*	*Beesia calthifolia*	Perennial herbs	Flower、Fruit	Multicolour	SP、SU	Multicolour(Fruit shape)	SU
Ranunculaceae	*Caltha*	*Caltha palustris*	Perennial herbs	Flower、Fruit	Yellow	SP、SU	Multicolour(Fruit shape)	SU、AU
Ranunculaceae	*Cimicifuga*	*Cimicifuga japonica*	Perennial herbs	Flower	White	AU	Multicolour(Fruit shape)	AU
Ranunculaceae	*Circaeaster*	*Circaeaster agrestis*	Annual herbs	Flower		SP、SU		

Ranunculaceae	*Clematis*	*Clematis heracleifolia*	Perennial herbs	Flower	Purple	SU、AU	Multicolour(Fruit shape)	AU
Ranunculaceae	*Delphinium*	*Delphinium siwanense*	Perennial herbs	Flower	Purple	SU、AU		
Ranunculaceae	*Delphinium*	*Delphinium sparsiflorum*	Perennial herbs	Flower	White	SU		
Ranunculaceae	*Dichocarpum*	*Dichocarpum fargesii*	Perennial herbs	Flower	Yellow	SP、SU	Multicolour(Fruit shape)	SU
Ranunculaceae	*Paeonia*	*Paeonia mairei*	Perennial herbs	Flower	Pink	SP	Multicolour(Fruit shape)	SU
Ranunculaceae	*Paeonia*	*Paeonia obovata subsp. willmottiae*	Perennial herbs	Flower	White			
Ranunculaceae	*Ranunculus*	*Ranunculus petrogeiton*	Perennial herbs	Flower	Yellow	SU	Multicolour(Fruit shape)	SU
Rhamnaceae	*Hovenia*	*Hovenia acerba*	Deciduous tree	Flower、Fruit	Green	SU	Brown	AU
Rhamnaceae	*Hovenia*	*Hovenia dulcis*	Deciduous tree	Flower、Fruit	Green	SU	Black	AU
Rhamnaceae	*Rhamnella*	*Rhamnella franguloides*	Deciduous shrub	Flower	Yellow	SU	Red	AU
Rhamnaceae	*Paliurus*	*Paliurus hemsleyanus*	Deciduous tree	Flower	Yellow	SP	Red	SU
Rhamnaceae	*Paliurus*	*Paliurus ramosissimus*	Deciduous shrub	Flower	Green	SU	Multicolour(Fruit shape)	AU
Rosaceae	*Sorbaria*	*Sorbaria arborea var. glabrata*	Deciduous shrub	Flower	White	SU		
Rosaceae	*Sorbaria*	*Sorbaria kirilowii*	Deciduous shrub	Flower	White	SU		
Rosaceae	*Sorbaria*	*Sorbaria arborea*	Deciduous shrub	Flower	White	SU		
Rosac	*Ceras*	*Cerasus*	Deciduo	Flowe	Whit	SP	Red	SP

eae	*us*	*schneideriana*	us tree	r	e			
Rosaceae	*Cerasus*	*Cerasus discadenia*	Deciduous shrub	Flower	White	SP	Red	SU
Rosaceae	*Cerasus*	*Cerasus dolichadenia*	Deciduous tree	Flower	White	SU	Red	SU
Rosaceae	*Cerasus*	*Cerasus conadenia*	Deciduous tree	Flower	White	SP	Red	SU
Rosaceae	*Cerasus*	*Cerasus setulosa*	Deciduous tree	Flower	Pink	SP	Red	SU
Rosaceae	*Cerasus*	*Cerasus tatsienensis*	Deciduous tree	Flower	White	SP	Red	SU
Rosaceae	*Cerasus*	*Cerasus polytricha*	Deciduous tree	Flower	White	SP	Red	SU
Rosaceae	*Cerasus*	*Cerasus szechuanica*	Deciduous tree	Flower	White	SP	Red	SU
Rosaceae	*Cerasus*	*Cerasus tomentosa*	Deciduous shrub	Flower	White	SP	Red	SU
Rosaceae	*Cerasus*	*Cerasus dictyoneura*	Deciduous shrub	Flower	Pink	SP	Red	SU
Rosaceae	*Cerasus*	*Cerasus humilis*	Deciduous shrub	Flower	White	SP	Red	SU
Rosaceae	*Cerasus*	*Cerasus clarofolia*	Deciduous tree	Flower	Pink	SP	Red	SU
Rosaceae	*Cerasus*	*Cerasus conradinae*	Deciduous tree	Flower	White	SP	Red	SP
Rosaceae	*Cerasus*	*Cerasus serrulata var. pubescens*	Deciduous tree	Flower	White	SP	Red	SU
Rosaceae	*Cerasus*	*Cerasus stipulacea*	Deciduous tree	Flower	White	SP	Red	SU
Rosaceae	*Cerasus*	*Cerasus pseudocerasus*	Deciduous tree	Flower	White	SP	Red	SP
Rosaceae	*Cotoneaster*	*Cotoneaster ambiguus*	Deciduous shrub	Flower	White	SU	Black	AU
Rosaceae	*Cotoneaster*	*Cotoneaster multiflorus var. calocarpus*	Deciduous shrub	Flower	White	SU	Red	AU
Rosaceae	*Cotoneaster*	*Cotoneaster horizontalis var. perpusillus*	Deciduous shrub	Flower	Pink	SU	Red	AU
Rosaceae	*Cotoneaster*	*Cotoneaster moupinensis*	Deciduous shrub	Flower	Pink	SU	Black	AU
Rosaceae	*Cotoneaster*	*Cotoneaster apiculatus*	Deciduous shrub	Flower	Pink	SU	Red	AU

Rosaceae	*Cotoneaster*	*Cotoneaster tenuipes*	Deciduous shrub	Flower	White	SU	Black	AU
Rosaceae	*Cotoneaster*	*Cotoneaster divaricatus*	Deciduous shrub	Flower	Pink	SU	Red	AU
Rosaceae	*Cotoneaster*	*Cotoneaster salicifolius var. rugosus*	Deciduous shrub	Flower	White	SU	Red	AU
Rosaceae	*Cotoneaster*	*Cotoneaster adpressus*	Deciduous shrub	Flower	Pink	SU	Red	AU
Rosaceae	*Cotoneaster*	*Cotoneaster submultiflorus*	Deciduous shrub	Flower	White	SU	Red	AU
Rosaceae	*Cotoneaster*	*Cotoneaster acutifolius*	Deciduous shrub	Flower	Pink	SU	Black	AU
Rosaceae	*Cotoneaster*	*Cotoneaster acutifolius var. villosulus*	Deciduous shrub	Flower	Pink	SU	Black	AU
Rosaceae	*Cotoneaster*	*Cotoneaster foveolatus*	Deciduous shrub	Flower	Pink	SU	Black	AU
Rosaceae	*Cotoneaster*	*Cotoneaster horizontalis*	Deciduous shrub	Flower	Pink	SU	Red	AU
Rosaceae	*Cotoneaster*	*Cotoneaster gracilis*	Deciduous shrub	Flower	Pink	SU	Red	AU
Rosaceae	*Cotoneaster*	*Cotoneaster multiflorus*	Deciduous shrub	Flower	White	SU	Red	AU
Rosaceae	*Cotoneaster*	*Cotoneaster zabelii*	Deciduous shrub	Flower	Red	SU	Red	AU
Rosaceae	*Rubus*	*Rubus eucalyptus var. villosus*	Deciduous shrub	Flower	White	SP	Red	SU
Rosaceae	*Rubus*	*Rubus subtibetanus*	Deciduous shrub	Flower	White	SU	Black	SU
Rosaceae	*Rubus*	*Rubus pungens var. villosus*	Deciduous shrub	Flower	White	SP	Red	SU
Rosaceae	*Rubus*	*Rubus eucalyptus*	Deciduous shrub	Flower	White	SP	Red	SU
Rosaceae	*Rubus*	*Rubus idaeopsis*	Deciduous shrub	Flower	Purple	SU	Red	SU
Rosaceae	*Rubus*	*Rubus pungens var. oldhamii*	Deciduous shrub	Flower	White	SP	Red	SU
Rosaceae	*Rubus*	*Rubus amabilis*	Deciduous shrub	Flower	White	SP	Red	SU
Rosaceae	*Rubus*	*Rubus simplex*	Deciduous shrub	Flower	White	SU	Red	AU
Rosac	*Rubus*	*Rubus*	Deciduo	Flowe	Pink	SU	Red	AU

eae		*flosculosus*	us shrub	r				
Rosaceae	*Rubus*	*Rubus pileatus*	Deciduous shrub	Flower	White	SU	Red	AU
Rosaceae	*Rubus*	*Rubus piluliferus*	Deciduous shrub	Flower	Pink	SU	Red	SU
Rosaceae	*Rubus*	*Rubus bambusarum*	Evergreen shrub	Flower	Pink	SU	Red	SU
Rosaceae	*Rubus*	*Rubus pungens*	Deciduous shrub	Flower	White	SP	Red	SU
Rosaceae	*Rubus*	*Rubus biflorus*	Deciduous shrub	Flower	White	SU	Yellow	SU
Rosaceae	*Rubus*	*Rubus idaeus*	Deciduous shrub	Flower	White	SU	Red	AU
Rosaceae	*Rubus*	*Rubus phoenicolasius*	Deciduous shrub	Flower	Purple	SU	Red	SU
Rosaceae	*Rubus*	*Rubus swinhoei*	Deciduous shrub	Flower	White	SU	Black	SU
Rosaceae	*Rubus*	*Rubus xanthoneurus*	Deciduous shrub	Flower	White	SU	Red	AU
Rosaceae	*Rubus*	*Rubus innominatus*	Deciduous shrub	Flower	Purple	SU	Red	SU
Rosaceae	*Rubus*	*Rubus cockburnianus*	Deciduous shrub	Flower	Pink	SU	Purple	AU
Rosaceae	*Rubus*	*Rubus forrestianus*	Deciduous shrub	Flower	White	SU	Red	SU
Rosaceae	*Rubus*	*Rubus innominatus var. kuntzeanus*	Deciduous shrub	Flower	Purple	SU	Red	SU
Rosaceae	*Rubus*	*Rubus mesogaeus var. oxycomus*	Deciduous shrub	Flower	White	SP	Purple	SU
Rosaceae	*Rubus*	*Rubus mesogaeus*	Deciduous shrub	Flower	White	SP	Purple	SU
Rosaceae	*Rubus*	*Rubus subcoreanus*	Deciduous shrub	Flower	Purple	SU	Red	SU
Rosaceae	*Rubus*	*Rubus parvifolius var. adenochlamys*	Deciduous shrub	Flower	Pink	SU	Red	SU
Rosaceae	*Rubus*	*Rubus lasiostylus*	Deciduous shrub	Flower	Red	SU	Red	AU
Rosaceae	*Rubus*	*Rubus lambertianus*	Deciduous shrub	Flower	White	SU	Red	AU
Rosaceae	*Rubus*	*Rubus niveus*	Deciduous shrub	Flower	Red	SU	Purple	AU

Rosaceae	*Rubus*	*Rubus xanthocarpus*	Deciduous shrub	Flower	White	SP	Yellow	SU
Rosaceae	*Rubus*	*Rubus coreanus*	Deciduous shrub	Flower	Purple	SP	Red	SU
Rosaceae	*Rubus*	*Rubus wallichianus*	Deciduous shrub	Flower	White	SP	Yellow	SU
Rosaceae	*Rubus*	*Rubus corchorifolius*	Deciduous shrub	Flower	White	SP	Red	SP
Rosaceae	*Rubus*	*Rubus coreanus var. tomentosus*	Deciduous shrub	Flower	Purple	SP	Red	SU
Rosaceae	*Rubus*	*Rubus parkeri*	Deciduous shrub	Flower	White	SU	Purple	SU
Rosaceae	*Rubus*	*Rubus chroosepalus*	Evergreen shrub	Flower	Yellow	SU	Purple	SU
Rosaceae	*Rubus*	*Rubus lambertianus var. glaber*	Deciduous shrub	Flower	White	SU	Yellow	AU
Rosaceae	*Neillia*	*Neillia ribesioides*	Deciduous shrub	Flower	Pink	SP		
Rosaceae	*Neillia*	*Neillia sinensis*	Deciduous shrub	Flower	Pink	SU		
Rosaceae	*Spiraea*	*Spiraea alpina*	Deciduous shrub	Flower	White	SU		
Rosaceae	*Spiraea*	*Spiraea miyabei var. glabrata*	Deciduous shrub	Flower	White	SU		
Rosaceae	*Spiraea*	*Spiraea schneideriana*	Deciduous shrub	Flower	White	SU		
Rosaceae	*Spiraea*	*Spiraea lasiocarpa*	Deciduous shrub	Flower	White	SP		
Rosaceae	*Spiraea*	*Spiraea schneideriana var. amphidoxa*	Deciduous shrub	Flower	White	SU		
Rosaceae	*Spiraea*	*Spiraea henryi*	Deciduous shrub	Flower	White	SP		
Rosaceae	*Spiraea*	*Spiraea blumei var. pubicarpa*	Deciduous shrub	Flower	White	SP		
Rosaceae	*Spiraea*	*Spiraea miyabei var. pilosula*	Deciduous shrub	Flower	White	SU		
Rosaceae	*Spiraea*	*Spiraea longigemmis*	Deciduous shrub	Flower	White	SP		
Rosaceae	*Spiraea*	*Spiraea veitchii*	Deciduous shrub	Flower	White	SU		
Rosaceae	*Spiraea*	*Spiraea ovalis*	Deciduous shrub	Flower	White	SP		

Rosaceae	*Spiraea*	*Spiraea uratensis*	Deciduous shrub	Flower	White	SU
Rosaceae	*Spiraea*	*Spiraea myrtilloides*	Deciduous shrub	Flower	White	SU
Rosaceae	*Spiraea*	*Spiraea rosthornii*	Deciduous shrub	Flower	White	SU
Rosaceae	*Spiraea*	*Spiraea hirsuta var. rotundifolia*	Deciduous shrub	Flower	White	SP
Rosaceae	*Spiraea*	*Spiraea japonica var. fortunei*	Deciduous shrub	Flower	Pink	SU
Rosaceae	*Spiraea*	*Spiraea thunbergii*	Deciduous shrub	Flower	White	SP
Rosaceae	*Spiraea*	*Spiraea wilsonii*	Deciduous shrub	Flower	White	SU
Rosaceae	*Spiraea*	*Spiraea blumei var. microphylla*	Deciduous shrub	Flower	White	SP
Rosaceae	*Spiraea*	*Spiraea chinensis*	Deciduous shrub	Flower	White	SP
Rosaceae	*Spiraea*	*Spiraea hypericifolia*	Deciduous shrub	Flower	White	SU
Rosaceae	*Spiraea*	*Spiraea hirsuta*	Deciduous shrub	Flower	White	SP
Rosaceae	*Spiraea*	*Spiraea fritschiana*	Deciduous shrub	Flower	White	SU
Rosaceae	*Spiraea*	*Spiraea japonica var. acuminata*	Deciduous shrub	Flower	Pink	SU
Rosaceae	*Spiraea*	*Spiraea pubescens var. lasiocarpa*	Deciduous shrub	Flower	White	SU
Rosaceae	*Spiraea*	*Spiraea sericea*	Deciduous shrub	Flower	White	SU
Rosaceae	*Spiraea*	*Spiraea prunifolia*	Deciduous shrub	Flower	White	SP
Rosaceae	*Spiraea*	*Spiraea trilobata*	Deciduous shrub	Flower	White	SU
Rosaceae	*Spiraea*	*Spiraea flexuosa*	Deciduous shrub	Flower	White	SU
Rosaceae	*Spiraea*	*Spiraea pubescens*	Deciduous shrub	Flower	White	SU
Rosaceae	*Spiraea*	*Spiraea mollifolia*	Deciduous shrub	Flower	White	SU
Rosaceae	*Spiraea*	*Spiraea fritschiana var. angulata*	Deciduous shrub	Flower	White	SU

Rosaceae	*Spiraea*	*Spiraea pjassetzkii*	Deciduous shrub	Flower	White	SP		
Rosaceae	*Armeniaca*	*Armeniaca sibirica*	Deciduous tree	Flower	White	SP	Yellow	SU
Rosaceae	*Armeniaca*	*Armeniaca vulgaris var. meixianensis*	Deciduous tree	Flower	White	SP	Yellow	SU
Rosaceae	*Osteomeles*	*Osteomeles schwerinae var. microphylla*	Deciduous shrub	Flower	White	SP		
Rosaceae	*Potentilla*	*Potentilla freyniana*	Perennial herbs	Flower、Fruit	Yellow	SP	Multicolour(Fruit shape)	SU
Rosaceae	*Potentilla*	*Potentilla fragarioides*	Perennial herbs	Flower、Fruit	Yellow	SP	Multicolour(Fruit shape)	SU
Rosaceae	*Potentilla*	*Potentilla discolor*	Perennial herbs	Flower	Yellow	SP、SU	Multicolour(Fruit shape)	AU
Rosaceae	*Potentilla*	*Potentilla chinensis*	Perennial herbs	Flower	Yellow	SP、SU	Multicolour(Fruit shape)	AU
Rosaceae	*Sibbaldia*	*Sibbaldia procumbens var. aphanopetala*	Perennial herbs	Flower	Yellow	SU	Multicolour(Fruit shape)	SU
Rosaceae	*Amygdalus*	*Amygdalus davidiana*	Deciduous tree	Flower	Pink	SP	Yellow	SU
Rosaceae	*Amygdalus*	*Amygdalus kansuensis*	Deciduous tree	Flower	White	SP	Yellow	SU
Rosaceae	*Amelanchier*	*Amelanchier sinica*	Deciduous tree	Flower	White	SP	Black	AU
Rosaceae	*Photinia*	*Photinia beauverdiana*	Deciduous shrub	Flower	White	SP	Red	SU
Rosaceae	*Photinia*	*Photinia serratifolia*	Evergreen shrub	Flower	White	SP	Red	AU
Rosaceae	*Photinia*	*Photinia bodinieri*	Deciduous tree	Flower	White	SP		
Rosaceae	*Photinia*	*Photinia parvifolia*	Deciduous shrub	Flower	White	SP	Red	SU
Rosaceae	*Photinia*	*Photinia villosa var. sinica*	Deciduous shrub	Flower	White	SP	Red	AU
Rosaceae	*Photinia*	*Photinia beauverdiana*	Deciduous shrub	Flower	White	SP	Red	SU

		var. brevifolia						
Rosaceae	*Duchesnea*	*Duchesnea indica*	Perennial herbs	Flower、Fruit	Yellow	SU	Red	AU
Rosaceae	*Crataegus*	*Crataegus pinnatifida*	Deciduous tree	Flower	White	SU	Red	AU
Rosaceae	*Crataegus*	*Crataegus shensiensis*	Deciduous shrub	Flower	White	SU	Red	AU
Rosaceae	*Crataegus*	*Crataegus aurantia*	Deciduous tree	Flower	White	SU	Red	AU
Rosaceae	*Crataegus*	*Crataegus wilsonii*	Deciduous shrub	Flower	White	SP	Red	AU
Rosaceae	*Crataegus*	*Crataegus hupehensis*	Deciduous tree	Flower	White	SU	Red	AU
Rosaceae	*Crataegus*	*Crataegus cuneata*	Deciduous shrub	Flower	White	SU	Red	AU
Rosaceae	*Sibbaldia*	*Sibbaldia purpurea var. macropetala*	Perennial herbs	Flower	Red	SU		
Rosaceae	*Rosa*	*Rosa tsinglingensis*	Deciduous shrub	Flower、Fruit	White	SU	Red	AU
Rosaceae	*Rosa*	*Rosa prattii*	Deciduous shrub	Flower、Fruit	Pink	SU	Red	AU
Rosaceae	*Rosa*	*Rosa acicularis*	Deciduous shrub	Flower、Fruit	Pink	SU	Red	SU
Rosaceae	*Rosa*	*Rosa giraldii var. bidentata*	Deciduous shrub	Flower、Fruit	Pink	SU	Red	SU
Rosaceae	*Rosa*	*Rosa sweginzowii*	Deciduous shrub	Flower、Fruit	Pink	SU	Red	AU
Rosaceae	*Rosa*	*Rosa caudata*	Deciduous shrub	Flower、Fruit	Red	SU	Red	SU
Rosaceae	*Rosa*	*Rosa davidii var. elongata*	Deciduous shrub	Flower、Fruit	Pink	SU	Red	AU
Rosaceae	*Rosa*	*Rosa glomerata*	Deciduous shrub	Flower、Fruit	White	SU	Red	AU
Rosac	*Rosa*	*Rosa setipoda*	Deciduo	Flowe	Pink	SU	Red	AU

eae			us shrub	r 、Fruit				
Rosaceae	*Rosa*	*Rosa omeiensis f. pteracantha*	Deciduous shrub	Flower 、Fruit	White	SU	Red	SU
Rosaceae	*Rosa*	*Rosa filipes*	Deciduous shrub	Flower 、Fruit	White	SU	Red	SU
Rosaceae	*Rosa*	*Rosa caudata var. maxima*	Deciduous shrub	Flower 、Fruit	Red	SU	Red	SU
Rosaceae	*Rosa*	*Rosa rubus var. parviflorus*	Deciduous shrub	Flower 、Fruit	White	SP	Red	SU
Rosaceae	*Rosa*	*Rosa willmottiana*	Deciduous shrub	Flower 、Fruit	Pink	SU	Red	SU
Rosaceae	*Rosa*	*Rosa bella*	Deciduous shrub	Flower 、Fruit	Pink	SU	Red	AU
Rosaceae	*Rosa*	*Rosa cymosa*	Deciduous shrub	Flower 、Fruit	White	SU	Red	SU
Rosaceae	*Rosa*	*Rosa davidii*	Deciduous shrub	Flower 、Fruit	Pink	SU	Red	AU
Rosaceae	*Rosa*	*Rosa helenae*	Deciduous shrub	Flower 、Fruit	White	SU	Red	AU
Rosaceae	*Rosa*	*Rosa moyesii*	Deciduous shrub	Flower 、Fruit	Red	SU	Red	AU
Rosaceae	*Rosa*	*Rosa sweginzowii var. glandulosa*	Deciduous shrub	Flower 、Fruit	Pink	SU	Red	AU
Rosaceae	*Rosa*	*Rosa banksiopsis*	Deciduous shrub	Flower 、Fruit	Pink	SU	Red	SU
Rosaceae	*Rosa*	*Rosa corymbulosa*	Deciduous shrub	Flower 、Fruit	Red	SU	Red	AU
Rosaceae	*Rosa*	*Rosa sertata*	Deciduous shrub	Flower 、Fruit	Pink	SU	Red	AU

Rosaceae	*Rosa*	*Rosa giraldii var. venulosa*	Deciduous shrub	Flower、Fruit	Pink	SU	Red	SU
Rosaceae	*Rosa*	*Rosa brunonii*	Deciduous shrub	Flower、Fruit	White	SU	Purple	SU
Rosaceae	*Rosa*	*Rosa omeiensis*	Deciduous shrub	Flower、Fruit	White	SU	Red	SU
Rosaceae	*Rosa*	*Rosa multiflora*	Deciduous shrub	Flower、Fruit	White	SU	Red	AU
Rosaceae	*Rosa*	*Rosa hugonis*	Deciduous shrub	Flower、Fruit	Yellow	SU	Red	SU
Rosaceae	*Rosa*	*Rosa laevigata*	Evergreen shrub	Flower、Fruit	White	SP	Purple	SU
Rosaceae	*Rosa*	*Rosa cymosa var. puberula*	Deciduous shrub	Flower、Fruit	White	SU	Red	SU
Rosaceae	*Rosa*	*Rosa xanthina f. normalis*	Deciduous shrub	Flower、Fruit	Yellow	SP	Purple	SU
Rosaceae	*Rosa*	*Rosa giraldii*	Deciduous shrub	Flower、Fruit	Pink	SU	Red	SU
Rosaceae	*Rosa*	*Rosa rubus*	Deciduous shrub	Flower、Fruit	White	SP	Red	SU
Rosaceae	*Rosa*	*Rosa henryi*	Deciduous shrub	Flower、Fruit	White	SU	Red	AU
Rosaceae	*Rosa*	*Rosa multiflora var. cathayensis*	Deciduous shrub	Flower、Fruit	Pink	SU	Red	AU
Rosaceae	*Rosa*	*Rosa banksiae var. normalis*	Deciduous shrub	Flower	White	SU		
Rosaceae	*Rosa*	*Rosa roxburghii*	Deciduous shrub	Flower、Fruit	Pink	SU	Red	AU
Rosaceae	*Rosa*	*Rosa roxburghii f. normalis*	Deciduous shrub	Flower、Fruit	Pink	SU	Red	AU

Rosaceae	*Rosa*	*Rosa bella var. nuda*	Deciduous shrub	Flower、Fruit	Pink	SU	Red	AU
Rosaceae	*Malus*	*Malus toringo*	Deciduous shrub	Flower	Pink	SP	Red	AU
Rosaceae	*Malus*	*Malus kansuensis var. calva*	Deciduous tree	Flower	White	SU	Yellow	SU
Rosaceae	*Malus*	*Malus kansuensis*	Deciduous tree	Flower	White	SU	Yellow	SU
Rosaceae	*Malus*	*Malus halliana*	Deciduous tree	Flower	Pink	SP	Purple	AU
Rosaceae	*Malus*	*Malus honanensis*	Deciduous shrub	Flower	Pink	SP	Yellow	AU
Rosaceae	*Malus*	*Malus yunnanensis var. veitchii*	Deciduous tree	Flower	White	SP	Red	AU
Rosaceae	*Malus*	*Malus baccata var. gracilis*	Deciduous tree	Flower	White	SP	Red	AU
Rosaceae	*Malus*	*Malus mandshurica*	Deciduous tree	Flower	White	SU	Red	AU
Rosaceae	*Malus*	*Malus hupehensis*	Deciduous tree	Flower	White	SP	Yellow	AU
Rosaceae	*Chaenomeles*	*Chaenomeles cathayensis*	Deciduous tree	Flower	Pink	SP	Yellow	AU
Rosaceae	*Geum*	*Geum aleppicum*	Perennial herbs	Flower	Yellow	SU、AU	Multicolour(Fruit shape)	SU、AU
Rosaceae	*Geum*	*Geum japonicum*	Perennial herbs	Flower	Yellow	SP、SU	Multicolour(Fruit shape)	SU、AU
Rosaceae	*Prunus*	*Prunus salicina*	Deciduous tree	Flower	White	SP	Purple	SU
Rosaceae	*Pyrus*	*Pyrus serrulata*	Deciduous tree	Flower	White	SP	Brown	SU
Rosaceae	*Pyrus*	*Pyrus xerophila*	Deciduous tree	Flower	White	SP	Yellow	AU
Rosaceae	*Pyrus*	*Pyrus ussuriensis*	Deciduous tree	Flower	White	SP	Yellow	AU
Rosaceae	*Pyrus*	*Pyrus betulifolia*	Deciduous tree	Flower	White	SP	Brown	AU
Rosaceae	*Pyrus*	*Pyrus phaeocarpa*	Deciduous tree	Flower	White	SP	Brown	AU

Rosaceae	*Rhodotypos*	*Rhodotypos scandens*	Deciduous shrub	Flower	White	SP	Black	SU
Rosaceae	*Pyracantha*	*Pyracantha angustifolia*	Evergreen shrub	Fruit	White	SP	Red	AU
Rosaceae	*Pyracantha*	*Pyracantha crenulata*	Evergreen shrub	Fruit	White	SP	Red	AU
Rosaceae	*Pyracantha*	*Pyracantha atalantioides*	Evergreen shrub	Fruit	White	SP	Red	AU
Rosaceae	*Pyracantha*	*Pyracantha crenulata var. kansuensis*	Evergreen shrub	Fruit	White	SP	Red	AU
Rosaceae	*Pyracantha*	*Pyracantha fortuneana*	Evergreen shrub	Fruit	White	SP	Red	AU
Rosaceae	*Sorbus*	*Sorbus tapashana*	Deciduous tree	Flower	White	SU	Red	AU
Rosaceae	*Sorbus*	*Sorbus hemsleyi*	Deciduous tree	Flower	White	SP	Red	AU
Rosaceae	*Sorbus*	*Sorbus caloneura*	Deciduous tree	Flower	White	SP	Brown	AU
Rosaceae	*Sorbus*	*Sorbus alnifolia*	Deciduous tree	Flower	White	SP	Red	AU
Rosaceae	*Sorbus*	*Sorbus tsinlingensis*	Deciduous tree	Flower	White	SP	Red	AU
Rosaceae	*Sorbus*	*Sorbus folgneri var. parvifolius*	Deciduous tree	Flower	White	SP	Red	SU
Rosaceae	*Sorbus*	*Sorbus hupehensis*	Deciduous tree	Flower	White	SP	Purple	AU
Rosaceae	*Sorbus*	*Sorbus discolor*	Deciduous tree	Flower	White	SP	Multicolour(Fruit shape)	AU
Rosaceae	*Sorbus*	*Sorbus pohuashanensis*	Deciduous tree	Flower	White	SP	Red	AU
Rosaceae	*Sorbus*	*Sorbus folgneri*	Deciduous tree	Flower	White	SP	Red	SU
Rosaceae	*Sorbus*	*Sorbus yuana*	Deciduous tree	Flower	White	SU	Red	AU
Rosaceae	*Stranvaesia*	*Stranvaesia davidiana*	Evergreen shrub	Fruit	White	SU	Red	AU
Rosaceae	*Kerria*	*Kerria japonica*	Deciduous shrub	Flower	Yellow	SP		
Rosaceae	*Sanguisorba*	*Sanguisorba officinalis*	Perennial herbs	Flower、	Purple	SU	Multicolour(Frui	AU

				Fruit			t shape)	
Rosaceae	*Cerasus*	*Maddenia wilsonii*	Deciduous tree	Flower、Fruit	Yellow	SP	Black	SU
Rosaceae	*Cerasus*	*Maddenia incisoserrata*	Deciduous shrub	Flower、Fruit	Yellow	SP	Purple	SU
Rosaceae	*Cerasus*	*Laurocerasus zippeliana*	Evergreen tree	Flower、Fruit	White	SU	Black	WI
Rosaceae	*Prunus*	*Padus avium var. asiatica*	Deciduous tree	Flower、Fruit	White	SP	Black	SU
Rosaceae	*Prunus*	*Padus brachypoda*	Deciduous tree	Flower、Fruit	White	SP	Black	SU
Rosaceae	*Prunus*	*Padus napaulensis*	Deciduous tree	Flower、Fruit	White	SP	Black	SU
Rosaceae	*Prunus*	*Padus velutina*	Deciduous tree	Flower、Fruit	White	SP	Red	SU
Rosaceae	*Prunus*	*Padus obtusata*	Deciduous tree	Flower、Fruit	White	SP	Black	SU
Rosaceae	*Prunus*	*Padus stellipila*	Deciduous tree	Flower、Fruit	White	SP	Black	SU
Rosaceae	*Prunus*	*Padus wilsonii*	Deciduous tree	Flower、Fruit	White	SP	Black	SU
Rosaceae	*Prunus*	*Padus buergeriana*	Deciduous tree	Flower、Fruit	White	SP	Black	SU
Rosaceae	*Fragaria*	*Fragaria orientalis*	Perennial herbs	Flower	White	SU	Red	SU、AU
Rosaceae	*Prinsepia*	*Prinsepia uniflora var. serrata*	Deciduous shrub	Flower	White	SP	Red	AU
Rosaceae	*Exochorda*	*Exochorda giraldii*	Deciduous shrub	Flower	White	SP		
Rosaceae	*Acomastylis*	*Acomastylis elata*	Perennial herbs	Flower、Fruit	Yellow	SU	Multicolour(Fruit shape)	SU

Rosaceae	*Agrimonia*	*Agrimonia pilosa*	Perennial herbs	Flower	Yellow	SU、AU	Multicolour(Fruit shape)	SU、AU
Rosaceae	*Agrimonia*	*Agrimonia pilosa var. nepalensis*	Perennial herbs	Flower	Yellow			
Rosaceae	*Alchemilla*	*Alchemilla japonica*	Perennial herbs	Flower、Fruit	Green	SU		
Rosaceae	*Fragaria*	*Fragaria gracilis*	Perennial herbs	Flower、Fruit	White	SP、SU	Red	SU
Rosaceae	*Fragaria*	*Fragaria nilgerrensis*	Perennial herbs	Flower、Fruit	White	SP、SU	Multicolour(Fruit shape)	SP、SU
Rosaceae	*Fragaria*	*Fragaria pentaphylla*	Perennial herbs	Flower、Fruit	White	SP	Red	SP、SU
Rosaceae	*Potentilla*	*Potentilla ancistrifolia*	Perennial herbs	Flower	Yellow	SP、SU	Multicolour(Fruit shape)	SU、AU
Rosaceae	*Potentilla*	*Potentilla anserina*	Perennial herbs	Flower	Yellow	SP、SU	Multicolour(Fruit shape)	SU、AU
Rosaceae	*Potentilla*	*Potentilla centigrana*	Annual and biennial herbs	Flower	Yellow	SP、SU	Multicolour(Fruit shape)	SP、SU
Rosaceae	*Potentilla*	*Potentilla cryptotaeniae*	Annual and biennial herbs	Flower	Yellow	SU、AU	Multicolour(Fruit shape)	SU、AU
Rosaceae	*Potentilla*	*Potentilla kleiniana*	Annual and biennial herbs	Flower、Fruit	Yellow	SP、SU	Multicolour(Fruit shape)	SU、AU
Rosaceae	*Potentilla*	*Potentilla reptans var. sericophylla*	Perennial herbs	Flower	Yellow	SP、SU	Multicolour(Fruit shape)	SU、AU
Rosaceae	*Potentilla*	*Potentilla sischanensis*	Perennial herbs	Flower	Yellow	SP、SU	Multicolour(Fruit shape)	SP、SU
Rosaceae	*Potentilla*	*Potentilla supina*	Annual and	Flower	Yellow	SP、SU	Multicolour(Frui	SU、AU

			biennial herbs				t shape)	
Rubiaceae	*Leptodermis*	*Leptodermis pilosa*	Evergreen shrub	Flower	Pink	SU		
Rubiaceae	*Leptodermis*	*Leptodermis pilosa var. spicatiformis*	Evergreen shrub	Flower	Pink	SU		
Rubiaceae	*Leptodermis*	*Leptodermis oblonga*	Deciduous shrub	Flower	Red	SU		
Rubiaceae	*Emmenopterys*	*Emmenopterys henryi*	Deciduous tree	Flower	White	SU		
Rubiaceae	*Adina*	*Adina rubella*	Deciduous shrub	Flower	Red	SU		
Rubiaceae	*Ophiorrhiza*	*Ophiorrhiza japonica*	Annual herbs	Flower	White	SU		
Rubiaceae	*Galium*	*Galium verum*	Perennial herbs	Flower	Yellow	SP	Multicolour(Fruit shape)	SU、AU
Rubiaceae	*Paederia*	*Paederia foetida*	Deciduous liana	Flower	Purple	SU	Black	AU
Rubiaceae	*Uncaria*	*Uncaria sinensis*	Evergreen liana	Flower	Green	SU		
Rubiaceae	*Serissa*	*Serissa serissoides*	Deciduous shrub	Flower	White	SP		
Rubiaceae	*Galium*	*Galium boreale*	Perennial herbs	Flower	Multicolour	SP、SU	Multicolour(Fruit shape)	SU、AU
Rubiaceae	*Galium*	*Galium bungei*	Perennial herbs	Flower	Green	SP、SU、AU	Multicolour(Fruit shape)	SP、SU、AU、WI
Rubiaceae	*Galium*	*Galium bungei var. angustifolium*	Perennial herbs	Flower	Green	SU	Multicolour(Fruit shape)	AU
Rubiaceae	*Galium*	*Galium exile*	Annual herbs	Flower	White	SU	Brown	SU、AU
Rubiaceae	*Galium*	*Galium kinuta*	Perennial herbs	Flower	Multicolour	SU	Multicolour(Fruit shape)	SU、AU
Rubiaceae	*Galium*	*Galium odoratum*	Perennial herbs	Flower、Fruit	Multicolour	SU、AU	Multicolour(Fruit shape)	SU、AU

Rubiaceae	*Galium*	*Galium paradoxum*	Perennial herbs	Flower	White	SU、AU	Multicolour(Fruit shape)	SU、AU
Rubiaceae	*Rubia*	*Rubia ovatifolia*	Perennial herbs	Flower	Yellow	SU	Black	AU
Rutaceae	*Skimmia*	*Skimmia melanocarpa*	Evergreen shrub	Flower	Yellow	SP	Black	AU
Rutaceae	*Tetradium*	*Tetradium trichotomum*	Deciduous tree	Flower	White	SU	Red	AU
Rutaceae	*Tetradium*	*Tetradium ruticarpum*	Deciduous tree	Flower	White	SU	Red	AU
Rutaceae	*Tetradium*	*Tetradium daniellii*	Deciduous tree	Flower	White	SU	Black	AU
Rutaceae	*Phellodendron*	*Phellodendron chinense var. glabriusculum*	Deciduous tree	Flower	Green	SU	Black	AU
Rutaceae	*Citrus*	*Citrus cavaleriei*	Evergreen tree	Flower、Fruit	Purple	SU	Yellow	AU
Rutaceae	*Citrus*	*Citrus trifoliata*	Evergreen tree	Flower	White	SU	Yellow	AU
Rutaceae	*Toddalia*	*Toddalia asiatica*	Evergreen liana	Flower	Yellow	SU		
Rutaceae	*Dichroa*	*Orixa japonica*	Deciduous shrub	Flower	Green	SP		
Rutaceae	*Dictamnus*	*Dictamnus dasycarpus*	Perennial herbs	Flower	Purple	SP	Multicolour(Fruit shape)	SU
Sabiaceae	*Meliosma*	*Meliosma cuneifolia var. glabriuscula*	Deciduous shrub	Flower	Yellow	SU		
Sabiaceae	*Meliosma*	*Meliosma veitchiorum*	Deciduous tree	Flower	White	SP		
Sabiaceae	*Meliosma*	*Meliosma myriantha var. pilosa*	Deciduous tree	Flower	Yellow	SU		
Sabiaceae	*Meliosma*	*Meliosma cuneifolia*	Deciduous shrub	Flower	Yellow	SU		
Sabiaceae	*Meliosma*	*Meliosma oldhamii*	Deciduous tree	Flower	White	SU		
Sabiaceae	*Meliosma*	*Meliosma flexuosa*	Deciduous tree	Flower	White	SU		
Sabiaceae	*Meliosma*	*Meliosma alba*	Deciduous tree	Flower	Yellow	SU		

Salicaceae	*Xylosma*	*Xylosma congesta*	Evergreen tree	Flower	Yellow	SP	Black	WI
Salicaceae	*Populus*	*Populus davidiana*	Deciduous tree	Flower	Red	SP	Multicolour(Fruit shape)	SU
Salicaceae	*Idesia*	*Idesia polycarpa var. vestita*	Deciduous tree	Flower	Green	SP	Red	AU
Sapindaceae	*Sapindus*	*Sapindus delavayi*	Deciduous tree	Flower	Yellow	SU	Yellow	AU
Sapindaceae	*Sapindus*	*Sapindus saponaria*	Deciduous tree	Flower	Yellow	SP	Yellow	AU
Sapindaceae	*Xanthoceras*	*Xanthoceras sorbifolium*	Deciduous shrub	Flower	White	SP	Multicolour(Fruit shape)	AU
Sapindaceae	*Koelreuteria*	*Koelreuteria paniculata*	Deciduous tree	Flower	Yellow	SU	Multicolour(Fruit shape)	AU
Saururaceae	*Saururus*	*Saururus chinensis*	Perennial herbs	Flower	White	SP		
Saururaceae	*Houttuynia*	*Houttuynia cordata*	Perennial herbs	Flower、Fruit	White	SP	Multicolour(Fruit shape)	SU
Saxifragaceae	*Bergenia*	*Bergenia scopulosa*	Perennial herbs	Flower	Purple	SP、SU	Multicolour(Fruit shape)	SU、AU
Saxifragaceae	*Itea*	*Itea ilicifolia*	Evergreen shrub	Flower	Green	SP		
Saxifragaceae	*Astilbe*	*Astilbe chinensis*	Perennial herbs	Flower	Purple	SU	Multicolour(Fruit shape)	SU、AU
Saxifragaceae	*Astilbe*	*Astilbe rivularis var. myriantha*	Perennial herbs	Flower	White	SU	Multicolour(Fruit shape)	SU、AU
Saxifragaceae	*Chrysosplenium*	*Chrysosplenium macrophyllum*	Perennial herbs	Flower	White	SP		
Saxifragaceae	*Tiarella*	*Tiarella polyphylla*	Perennial herbs	Flower	White	SP、SU		
Saxifragacea	*Saxifraga*	*Saxifraga stolonifera*	Perennial herbs	Flower	White	SP、SU		

e								
Saxifragaceae	*Saxifraga*	*Saxifraga giraldiana*	Perennial herbs	Flower	Yellow	SU		
Saxifragaceae	*Saxifraga*	*Saxifraga sinomontana*	Perennial herbs	Flower	Yellow	SU		
Saxifragaceae	*Rodgersia*	*Rodgersia aesculifolia*	Perennial herbs	Flower、Fruit	White	SP、SU	Multicolour(Fruit shape)	SU、AU
Saxifragaceae	*Penthorum*	*Penthorum chinense*	Perennial herbs	Flower、Fruit	Yellow	SU、AU	Purple	AU
Saxifragaceae	*Chrysosplenium*	*Chrysosplenium biondianum*	Perennial herbs	Flower、Fruit	Green	SU	Multicolour(Fruit shape)	SU
Saxifragaceae	*Chrysosplenium*	*Chrysosplenium axillare*	Perennial herbs	Flower	Green	SU、AU	Multicolour(Fruit shape)	SU、AU
Saxifragaceae	*Chrysosplenium*	*Chrysosplenium giraldianum*	Perennial herbs	Flower	Green	SU、AU	Multicolour(Fruit shape)	SU、AU
Saxifragaceae	*Chrysosplenium*	*Chrysosplenium griffithii*	Perennial herbs	Flower	Yellow	SU、AU	Multicolour(Fruit shape)	SU、AU
Saxifragaceae	*Chrysosplenium*	*Chrysosplenium pilosum var. valdepilosum*	Perennial herbs	Flower	Yellow	SP、SU	Multicolour(Fruit shape)	SP、SU
Saxifragaceae	*Chrysosplenium*	*Chrysosplenium sinicum*	Perennial herbs	Flower	Green	SP、SU	Multicolour(Fruit shape)	SP、SU
Saxifragaceae	*Parnassia*	*Parnassia oreophila*	Perennial herbs	Flower	White	SU	Multicolour(Fruit shape)	AU
Saxifragaceae	*Parnassia*	*Parnassia viridiflora*	Perennial herbs	Flower	Green	SU	Multicolour(Fruit shape)	AU
Saxifragaceae	*Saxifraga*	*Saxifraga aurantiaca*	Perennial herbs	Flower	Yellow	SU		
Saxifragaceae	*Saxifraga*	*Saxifraga gemmigera*	Perennial herbs	Flower	Yellow	SU、AU	Multicolour(Fruit shape)	SU、AU
Saxifra	*Saxifr*	*Saxifraga*	Perennia	Flowe	Multi	SU、AU	Multicol	SU、AU

gaceae	*aga*	*melanocentra*	l herbs	r	colour		our(Fruit shape)	
Saxifragaceae	*Saxifraga*	*Saxifraga pseudohirculus*	Perennial herbs	Flower	Yellow	SU、AU	Multicolour(Fruit shape)	SU、AU
Saxifragaceae	*Saxifraga*	*Saxifraga sibirica*	Perennial herbs	Flower	White	SP、SU	Multicolour(Fruit shape)	SU、AU
Schisandraceae	*Schisandra*	*Schisandra glaucescens*	Deciduous liana	Flower	Yellow	SU	Red	SU
Schisandraceae	*Schisandra*	*Schisandra grandiflora*	Deciduous liana	Flower	White	SP	Red	SU
Schisandraceae	*Schisandra*	*Schisandra lancifolia*	Deciduous liana	Flower	Yellow	SU	Red	SU
Schisandraceae	*Schisandra*	*Schisandra propinqua subsp. sinensis*	Deciduous liana	Flower	Yellow	SU		
Schisandraceae	*Schisandra*	*Schisandra sphenanthera*	Deciduous liana	Flower	Yellow	SP		
Schisandraceae	*Schisandra*	*Schisandra propinqua subsp. intermedia*	Deciduous liana	Flower	Green	SU		
Schisandraceae	*Schisandra*	*Kadsura longipedunculata*	Evergreen liana	Flower	Yellow	SU	Red	AU
Scrophulariaceae	*Buddleja*	*Buddleja jinsixiaensis*	Deciduous shrub	Flower	Purple	SU		
Scrophulariaceae	*Buddleja*	*Buddleja alternifolia*	Deciduous shrub	Flower	Purple	SU		
Scrophulariaceae	*Buddleja*	*Buddleja nivea*	Deciduous shrub	Flower	Purple	SU		
Scrophulariaceae	*Buddleja*	*Buddleja officinalis*	Deciduous shrub	Flower	Purple	SP		
Scrophulari	*Buddleja*	*Buddleja albiflora*	Deciduous shrub	Flower	Purple	SP		

aceae								
Scrophulariaceae	*Buddleja*	*Buddleja davidii*	Deciduous shrub	Flower	Purple	SU		
Scrophulariaceae	*Digitalis*	*Digitalis purpurea*	Annual herbs	Flower	Red	SU		
Scrophulariaceae	*Pedicularis*	*Pedicularis torta*	Perennial herbs	Flower	White	SU		
Scrophulariaceae	*Pedicularis*	*Pedicularis plicata*	Perennial herbs	Flower	Yellow	SU		
Scrophulariaceae	*Pedicularis*	*Pedicularis giraldiana*	Perennial herbs	Flower	Multicolour	SU		
Scrophulariaceae	*Pedicularis*	*Pedicularis striata*	Perennial herbs	Flower	Yellow	SU		
Scrophulariaceae	*Linaria*	*Linaria vulgaris subsp. sinensis*	Perennial herbs	Flower	Multicolour	SU		
Scrophulariaceae	*Linaria*	*Linaria vulgaris*	Perennial herbs	Flower	Yellow			
Scrophulariaceae	*Rehmannia*	*Rehmannia glutinosa*	Perennial herbs	Flower	Red	SP	Multicolour(Fruit shape)	AU
Scrophulariaceae	*Mimulus*	*Mimulus tenellus var. nepalensis*	Perennial herbs	Flower	Yellow	SU、AU	Multicolour(Fruit shape)	SU、AU
Scrophulariaceae	*Mimulus*	*Mimulus tenellus var. tenellus*	Perennial herbs	Flower	Yellow	SU、AU	Multicolour(Fruit shape)	SU、AU
Scrophulariaceae	*Pedicularis*	*Pedicularis dissecta*	Perennial herbs	Flower、Fruit		SU	Multicolour(Fruit shape)	SU
Scrophulariaceae	*Pedicularis*	*Pedicularis lineata*	Perennial herbs	Flower	Purple			
Scrophulariaceae	*Scrophularia*	*Scrophularia modesta*	Perennial herbs	Flower	Green	SU、AU	Multicolour(Fruit shape)	SU、AU
Selagi	*Selagi*	*Selaginella*	Perennia	观叶				

nellaceae	*nella*	*sinensis*	l herbs					
Solanaceae	*Nicotiana*	*Nicotiana tabacum*	Annual herbs	Flower、Fruit	Red	SU	Multicolour(Fruit shape)	AU
Solanaceae	*Hyoscyamus*	*Hyoscyamus niger*	Annual and biennial herbs	Flower、Fruit	Yellow	SU	Multicolour(Fruit shape)	SU
Solanaceae	*Alkekengi*	*Physalis alkekengi var. franchetii*	Perennial herbs	Fruit			Yellow	AU
Solanaceae	*Alkekengi*	*Physalis alkekengi*	Perennial herbs	Fruit			Red	AU
Solanaceae	*Solanum*	*Solanum septemlobum*	Perennial herbs	Flower、Fruit	Purple	SU	Red	AU
Solanaceae	*Solanum*	*Solanum nigrum*	Annual herbs	Fruit			Black	AU
Solanaceae	*Physochlaina*	*Physochlaina infundibularis*	Perennial herbs	Flower	Yellow	SP		
Solanaceae	*Datura*	*Datura stramonium*	Annual herbs	Flower、Fruit	White	SU	Multicolour(Fruit shape)	AU
Solanaceae	*Physalis*	*Physalis angulata*	Annual herbs	Fruit	Multicolour	SP、SU、AU	Multicolour(Fruit shape)	SU、AU、WI
Stachyuraceae	*Stachyurus*	*Stachyurus chinensis*	Deciduous shrub	Flower	Yellow	SP		
Staphyleaceae	*Euscaphis*	*Euscaphis japonica*	Deciduous tree	Flower	Yellow	SU	Black	AU
Staphyleaceae	*Staphylea*	*Staphylea bumalda*	Deciduous shrub	Flower	White	SP	Multicolour(Fruit shape)	AU
Staphyleaceae	*Staphylea*	*Staphylea holocarpa var. rosea*	Deciduous shrub	Flower	Pink	SP	Multicolour(Fruit shape)	AU
Staphyleaceae	*Staphylea*	*Staphylea holocarpa*	Deciduous shrub	Flower	White	SP	Multicolour(Fruit shape)	AU
Styrac	*Ptero*	*Pterostyrax*	Deciduo	Flowe	Whit	SP		

aceae	*styrax*	*psilophyllus*	us tree	r	e			
Styracaceae	*Styrax*	*Styrax hemsleyanus*	Deciduous tree	Flower	White	SP		
Styracaceae	*Styrax*	*Styrax japonicus var. nervillosus*	Deciduous tree	Flower	White	SP		
Styracaceae	*Styrax*	*Styrax roseus*	Deciduous tree	Flower	White	SU		
Styracaceae	*Styrax*	*Styrax japonicus*	Deciduous tree	Flower	White	SP		
Symplocaceae	*Symplocos*	*Symplocos lucida*	Deciduous tree	Flower	White	SP	Black	SP
Symplocaceae	*Symplocos*	*Symplocos sumuntia*	Deciduous tree	Flower	White	SP	Purple	SU
Symplocaceae	*Symplocos*	*Symplocos anomala*	Deciduous tree	Flower	White	SP	Black	SP
Symplocaceae	*Symplocos*	*Symplocos paniculata*	Deciduous tree	Flower	White	SP	Purple	SU
Tamaricaceae	*Myricaria*	*Myricaria paniculata*	Deciduous shrub	Flower	Purple	SP		
Tamaricaceae	*Myricaria*	*Myricaria bracteata*	Deciduous shrub	Flower	Purple	SU		
Tapisciaceae	*Tapiscia*	*Tapiscia sinensis*	Deciduous tree	Flower	Yellow	SP	Red	SU
Tetracentraceae	*Tetracentron*	*Tetracentron sinense*	Deciduous tree	Flower	Yellow	SU		
Theaceae	*Stewartia*	*Stewartia sinensis var. shensiensis*	Deciduous shrub	Flower	White	SU		
Theaceae	*Camellia*	*Camellia grijsii var. shensiensis*	Evergreen shrub	Flower	White	SP		
Theaceae	*Camellia*	*Camellia cuspidata*	Evergreen shrub	Flower	White	SP		
Theaceae	*Camellia*	*Camellia oleifera*	Evergreen shrub	Flower	White	SP		
Theaceae	*Camellia*	*Camellia sinensis*	Evergreen shrub	Flower	White	AU		
Thym	*Daph*	*Daphne*	Evergree	Flowe	Purpl	SP	Red	SU

elaeaceae	*ne*	*tangutica var. wilsonii*	n shrub	r、Fruit	e			
Thymelaeaceae	*Daphne*	*Daphne giraldii*	Deciduous shrub	Flower、Fruit	Yellow	SP	Red	SU
Thymelaeaceae	*Daphne*	*Daphne myrtilloides*	Deciduous shrub	Flower、Fruit	Yellow	SP		
Thymelaeaceae	*Daphne*	*Daphne retusa*	Evergreen shrub	Flower、Fruit	Pink	SP	Red	SU
Thymelaeaceae	*Daphne*	*Daphne tangutica*	Evergreen shrub	Flower、Fruit	Purple	SP	Red	SU
Thymelaeaceae	*Daphne*	*Daphne genkwa*	Deciduous shrub	Flower、Fruit	Purple	SP	Multicolour(Fruit shape)	SU
Thymelaeaceae	*Wikstroemia*	*Wikstroemia ligustrina*	Deciduous shrub	Flower	Yellow	AU		
Thymelaeaceae	*Wikstroemia*	*Wikstroemia capitata*	Deciduous shrub	Flower	Yellow	SU		
Thymelaeaceae	*Wikstroemia*	*Wikstroemia stenophylla*	Evergreen shrub	Flower	Yellow	SU		
Thymelaeaceae	*Wikstroemia*	*Wikstroemia chamaedaphne*	Deciduous shrub	Flower	Yellow	SU		
Thymelaeaceae	*Wikstroemia*	*Wikstroemia pampaninii*	Deciduous shrub	Flower	Yellow	SU		
Thymelaeaceae	*Wikstroemia*	*Wikstroemia micrantha*	Deciduous shrub	Flower	Yellow	AU		
Thymelaeaceae	*Wikstroemia*	*Wikstroemia angustifolia*	Deciduous shrub	Flower	Yellow	SU		
Thymelaeaceae	*Stellera*	*Stellera chamaejasme*	Perennial herbs	Flower	Yellow	SP	Multicolour(Fruit shape)	SU
Thymelaeaceae	*Edgeworthia*	*Edgeworthia chrysantha*	Deciduous shrub	Flower	Yellow	SP		

Typhaceae	*Typha*	*Typha orientalis*	Perennial herbs	Flower、Fruit	Red	SU	Multicolour(Fruit shape)	AU
Typhaceae	*Typha*	*Typha latifolia*	Perennial herbs	Flower、Fruit	Red	SU	Multicolour(Fruit shape)	AU
Typhaceae	*Typha*	*Typha angustifolia*	Perennial herbs	Flower、Fruit	Red	SU	Multicolour(Fruit shape)	AU
Umbelliferae	*Libanotis*	*Libanotis sibirica*	Perennial herbs	Flower、Fruit	White	SU	Multicolour(Fruit shape)	SU
Umbelliferae	*Libanotis*	*Libanotis spodotrichoma*	Perennial herbs	Flower、Fruit	White	SU、AU	Multicolour(Fruit shape)	AU
Umbelliferae	*Libanotis*	*Libanotis lancifolia*	Perennial herbs	Flower、Fruit	Multicolour	AU	Multicolour(Fruit shape)	AU
Umbelliferae	*Libanotis*	*Libanotis buchtormensis*	Perennial herbs	Flower、Fruit	White	SU	Multicolour(Fruit shape)	SU
Umbelliferae	*Cryptotaenia*	*Cryptotaenia japonica*	Perennial herbs	Flower	White	SP	Multicolour(Fruit shape)	SU、AU
Umbelliferae	*Cnidium*	*Cnidium sinchianum*	Perennial herbs	Flower、Fruit	White	SU	Multicolour(Fruit shape)	AU
Umbelliferae	*Torilis*	*Torilis scabra*	Perennial herbs	Flower、Fruit	Multicolour	SP、SU	Brown	AU
Umbelliferae	*Notopterygium*	*Notopterygium incisum*	Perennial herbs	Flower	White	SU	Multicolour(Fruit shape)	SU
Umbelliferae	*Notopterygium*	*Notopterygium franchetii*	Perennial herbs	Flower	Yellow	SU	Multicolour(Fruit shape)	SU
Umbelliferae	*Peucedanum*	*Peucedanum ledebourielloides*	Perennial herbs	Flower	White	SU		
Umbelliferae	*Centella*	*Centella asiatica*	Perennial herbs	Flower、Fruit	Multicolour	SP、SU	Multicolour(Fruit shape)	AU
Umbelliferae	*Pimpinella*	*Pimpinella rhomboidea*	Perennial herbs	Flower	White	SP、SU	Multicolour(Frui	SU

							t shape)	
Umbelliferae	*Pimpinella*	*Pimpinella diversifolia*	Perennial herbs	Flower	White	SP、SU	Multicolour(Fruit shape)	AU
Umbelliferae	*Ligusticum*	*Ligusticum sinense 'Chuanxiong'*	Perennial herbs	Flower	White	SU	Multicolour(Fruit shape)	AU
Umbelliferae	*Ligusticum*	*Ligusticum sinense Oliv.*	Perennial herbs	Flower	White	SU	Multicolour(Fruit shape)	AU
Umbelliferae	*Saposhnikovia*	*Saposhnikovia divaricata*	Perennial herbs	Flower	White	SU	Multicolour(Fruit shape)	AU
Umbelliferae	*Anthriscus*	*Anthriscus sylvestris*	Perennial herbs	Flower	White	SP	Multicolour(Fruit shape)	SU
Umbelliferae	*Tongoloa*	*Tongoloa silaifolia*	Perennial herbs	Flower	Purple	AU	Multicolour(Fruit shape)	AU
Umbelliferae	*Tongoloa*	*Tongoloa elata*	Annual herbs	Flower、Fruit	White	SU、AU	Multicolour(Fruit shape)	SU、AU
Umbelliferae	*Angelica*	*Angelica tsinlingensis*	Perennial herbs	Flower	White	SU、AU	Multicolour(Fruit shape)	AU
Umbelliferae	*Angelica*	*Angelica sinensis*	Perennial herbs	Flower	White	SU	Multicolour(Fruit shape)	SU
Umbelliferae	*Angelica*	*Angelica polymorpha*	Perennial herbs	Flower	White	SU	Multicolour(Fruit shape)	AU
Umbelliferae	*Angelica*	*Angelica laxifoliata*	Perennial herbs	Flower	White	SU、AU	Multicolour(Fruit shape)	AU
Umbelliferae	*Angelica*	*Angelica dahurica*	Perennial herbs	Flower	White	SU	Multicolour(Fruit shape)	SU
Umbelliferae	*Bupleurum*	*Bupleurum scorzonerifolium*	Perennial herbs	Flower	Yellow	SU	Multicolour(Fruit shape)	AU
Umbelliferae	*Bupleurum*	*Bupleurum longicaule var. giraldii*	Perennial herbs	Flower、Fruit	Yellow	SU	Multicolour(Fruit shape)	AU
Umbel	*Buple*	*Bupleurum*	Perennia	Flowe	Yello	AU	Multicol	AU

liferae	*urum*	*Chinense*	l herbs	r	w		our(Fruit shape)	
Umbelliferae	*Bupleurum*	*Bupleurum dielsianum*	Perennial herbs	Flower	Yellow	AU		
Umbelliferae	*Sanicula*	*Sanicula elongata*	Perennial herbs	Flower、Fruit	White	SP	Yellow	SU
Umbelliferae	*Aegopodium*	*Aegopodium alpestre*	Perennial herbs	Flower、Fruit	White	SU	Multicolour(Fruit shape)	SU
Umbelliferae	*Carum*	*Carum buriaticum*	Perennial herbs	Flower	White	SP、SU	Multicolour(Fruit shape)	SU、AU
Umbelliferae	*Carum*	*Carum carvi*	Perennial herbs	Flower	Multicolour	SP、SU	Brown	SP、SU
Umbelliferae	*Cnidium*	*Cnidium monnieri*	Annual herbs	Flower	White	SP、SU	Multicolour(Fruit shape)	SU、AU
Umbelliferae	*Ferula*	*Ferula licentiana*	Perennial herbs	Flower	Yellow	SU	Multicolour(Fruit shape)	SU
Umbelliferae	*Foeniculum*	*Foeniculum vulgare*	Perennial herbs	Flower	Yellow	SU	Multicolour(Fruit shape)	SU、AU
Umbelliferae	*Heracleum*	*Heracleum moellendorffii*	Perennial herbs	Flower	White	SU	Multicolour(Fruit shape)	SU、AU
Umbelliferae	*Ligusticum*	*Ligusticum angelicifolium*	Perennial herbs	Flower	Purple	SU	Multicolour(Fruit shape)	AU
Umbelliferae	*Peucedanum*	*Peucedanum harry-smithii var. grande*	Perennial herbs	Flower	White	SU、AU	Multicolour(Fruit shape)	AU
Umbelliferae	*Pimpinella*	*Pimpinella arguta*	Perennial herbs	Flower	White	SU、AU	Multicolour(Fruit shape)	SU、AU
Umbelliferae	*Pimpinella*	*Pimpinella smithii*	Perennial herbs	Flower	White	SU、AU	Multicolour(Fruit shape)	SU、AU
Umbelliferae	*Pimpinella*	*Pimpinella valleculosa*	Perennial herbs	Flower	White	SU、AU	Multicolour(Fruit shape)	SU、AU
Umbel	*Ptern*	*Pternopetalum*	Annual	Flowe	Whit			

liferae	*opetalum*	*caespitosum*	herbs	r	e			
Umbelliferae	*Pternopetalum*	*Pternopetalum filicinum*	Perennial herbs	观叶				
Umbelliferae	*Pternopetalum*	*Pternopetalum vulgare*	Perennial herbs	Flower	Multicolour	SP、SU	Multicolour(Fruit shape)	SP、SU
Urticaceae	*Boehmeria*	*Boehmeria spicata*	Perennial herbs	Flower	Yellow	SU		
Urticaceae	*Gonostegia*	*Gonostegia hirta*	Perennial herbs	Flower、Fruit		SU、AU	Multicolour(Fruit shape)	
Urticaceae	*Elatostema*	*Elatostema stewardii*	Perennial herbs	Flower	Yellow	SU、AU		
Urticaceae	*Pilea*	*Pilea pumila*	Perennial herbs	Flower、Fruit		SU	Multicolour(Fruit shape)	AU
Urticaceae	*Pilea*	*Pilea notata*	Perennial herbs	Flower、Fruit	Yellow	SU、AU	Multicolour(Fruit shape)	AU
Urticaceae	*Pilea*	*Pilea angulata*	Perennial herbs	Flower、Fruit	Yellow	SU	Red	AU
Urticaceae	*Nanocnide*	*Nanocnide japonica*	Perennial herbs	Flower、Fruit	Purple	SP	Multicolour(Fruit shape)	SU
Urticaceae	*Boehmeria*	*Boehmeria tricuspis*	Perennial herbs	Flower		SU		
Urticaceae	*Elatostema*	*Elatostema involucratum*	Perennial herbs	Flower、Fruit		SU、AU	Multicolour(Fruit shape)	SU、AU
Urticaceae	*Elatostema*	*Elatostema obtusum*	Perennial herbs	Flower、Fruit		SU、AU	Multicolour(Fruit shape)	SU、AU
Urticaceae	*Laportea*	*Laportea bulbifera*	Perennial herbs	Flower、Fruit		SU	Multicolour(Fruit shape)	SU、AU、WI
Urticaceae	*Laportea*	*Laportea cuspidata*	Perennial herbs	Flower、Fruit		SU	Brown	SU、AU
Urticaceae	*Parietaria*	*Parietaria micrantha*	Annual herbs	Flower	Green	SU	Black	SU、AU
Urtica	*Pilea*	*Pilea japonica*	Perennia	Flowe		SU、AU	Brown	SU、AU

ceae			l herbs	r 、Fruit				
Urticaceae	*Pilea*	*Pilea sinofasciata*	Perennial herbs	Flower 、Fruit		SU	Multicolour(Fruit shape)	SU、AU
Verbenaceae	*Verbena*	*Verbena officinalis*	Perennial herbs	Flower 、Fruit	Purple	SP	Multicolour(Fruit shape)	AU
Violaceae	*Viola*	*Viola prionantha*	Perennial herbs	Flower	Purple	SP、SU		
Violaceae	*Viola*	*Viola philippica*	Perennial herbs	Flower	Purple	SP、SU		
Violaceae	*Viola*	*Viola grypoceras*	Perennial herbs	Flower	Purple	SP	Multicolour(Fruit shape)	SU
Violaceae	*Viola*	*Viola dissecta*	Perennial herbs	Flower 、Fruit	Purple	SP	Multicolour(Fruit shape)	SU
Violaceae	*Viola*	*Viola diffusa*	Annual herbs	Flower	Purple	SP	Multicolour(Fruit shape)	SU
Violaceae	*Viola*	*Viola biflora*	Perennial herbs	Flower	Yellow	SP、SU		
Violaceae	*Viola*	*Viola acuminata*	Perennial herbs	Flower	Multicolour	SP、SU		
Vitaceae	*Ampelopsis*	*Ampelopsis glandulosa var. hancei*	Deciduous liana	Flower	Yellow	SP	Purple	SU
Vitaceae	*Ampelopsis*	*Ampelopsis megalophylla*	Deciduous liana	Flower	Yellow	SU	Purple	SU
Vitaceae	*Ampelopsis*	*Ampelopsis delavayana*	Deciduous liana	Flower	Yellow	SU	Purple	AU
Vitaceae	*Ampelopsis*	*Ampelopsis delavayana var. glabra*	Deciduous liana	Flower	Yellow	SU	Purple	AU
Vitaceae	*Ampelopsis*	*Ampelopsis bodinieri var. cinerea*	Deciduous liana	Flower	Yellow	SP	Purple	SU
Vitaceae	*Ampelopsis*	*Ampelopsis chaffanjonii*	Deciduous liana	Flower	Yellow	SU	Red	SU
Vitaceae	*Ampelopsis*	*Ampelopsis aconitifolia*	Deciduous liana	Flower	Yellow	SU	Red	SU
Vitace	*Ampe*	*Ampelopsis*	Deciduo	Flowe	Yello	SU	Purple	SU

ae	*lopsis*	*humulifolia*	us liana	r	w			
Vitaceae	*Ampelopsis*	*Ampelopsis bodinieri*	Deciduous liana	Flower	Yellow	SP	Purple	SU
Vitaceae	*Ampelopsis*	*Ampelopsis japonica*	Deciduous liana	Flower	Yellow	SU	Multicolour(Fruit shape)	SU
Vitaceae	*Ampelopsis*	*Ampelopsis aconitifolia var. palmiloba*	Deciduous liana	Flower	Yellow	SU	Red	SU
Vitaceae	*Ampelopsis*	*Ampelopsis delavayana var. setulosa*	Deciduous liana	Flower	Yellow	SU	Purple	AU
Vitaceae	*Vitis*	*Vitis pseudoreticulata*	Deciduous liana	Flower	Green	SP	Purple	SU
Vitaceae	*Vitis*	*Vitis shenxiensis*	Deciduous liana	Flower	Green	SU	Purple	AU
Vitaceae	*Vitis*	*Vitis davidii*	Deciduous liana	Flower	Green	SP	Purple	SU
Vitaceae	*Vitis*	*Vitis wilsonae*	Deciduous liana	Flower	Green	SU	Purple	SU
Vitaceae	*Vitis*	*Vitis flexuosa*	Deciduous liana	Flower	Green	SP	Purple	SU
Vitaceae	*Vitis*	*Vitis romaneti*	Deciduous liana	Flower	Green	SP	Purple	SU
Vitaceae	*Vitis*	*Vitis betulifolia*	Deciduous liana	Flower	Green	SP	Purple	SU
Vitaceae	*Vitis*	*Vitis heyneana subsp. ficifolia*	Deciduous liana	Flower	Green	SU	Purple	SU
Vitaceae	*Vitis*	*Vitis piasezkii*	Deciduous liana	Flower	Green	SU	Purple	SU
Vitaceae	*Vitis*	*Vitis lanceolatifoliosa*	Deciduous liana	Flower	Green	SP	Purple	AU
Vitaceae	*Vitis*	*Vitis heyneana*	Deciduous liana	Flower	Green	SU	Purple	SU
Vitaceae	*Vitis*	*Vitis silvestrii*	Deciduous liana	Flower	Green	SP		
Vitaceae	*Vitis*	*Vitis bryoniifolia*	Deciduous liana	Flower	Green	SP	Red	SU
