# Supplementary material for: Enhancing Conservation Efforts in the Qinling Mountains Through Phenotypic Trait Diversity Optimization
Source: Plants (Basel). 2025 Jul 10;14(14):2130. doi: 10.3390/plants14142130 (PMC12300060; doi:10.3390/plants14142130)
Supplement: Supplementary file 1 [file plants-14-02130-s001.zip › S2 (ES, TS and PS).pdf]

Family	Genus	Species	Key protected wild plants in China(2021)
Actinidiaceae	*Actinidia*	*Actinidia arguta*	II
Apiaceae	*Glehnia*	*Glehnia littoralis*	II
Araliaceae	*Panax*	*Panax ginseng*	II
Araliaceae	*Panax*	*Panax bipinnatifidus*	II
Aristolochiaceae	*Saruma*	*Saruma henryi*	II
Berberidaceae	*Dysosma*	*Dysosma versipellis*	II
Berberidaceae	*Dysosma*	*Dysosma pleiantha*	II
Berberidaceae	*Sinopodophyllum*	*Sinopodophyllum hexandrum*	II
Cabombaceae	*Brasenia*	*Brasenia schreberi*	II
Calycanthaceae	*Calycanthus*	*Calycanthus chinensis*	II
Cercidiphyllaceae	*Cercidiphyllum*	*Cercidiphyllum japonicum*	II
Circaeasteraceae	*Kingdonia*	*Kingdonia uniflora*	II
Crassulaceae	*Rhodiola*	*Rhodiola yunnanensis*	II
Cupressaceae	*Metasequoia*	*Metasequoia glyptostroboides*	I
Cupressaceae	*Thuja*	*Thuja sutchuenensis*	I
Cycadaceae	*Cycas*	*Cycas revoluta*	I
Cynomoriaceae	*Cynomorium*	*Cynomorium songaricum*	II
Elaeagnaceae	*Elaeagnus*	*Elaeagnus mollis*	II
Fabaceae	*Ammopiptanthus*	*Ammopiptanthus mongolicus*	II
Fabaceae	*Glycine*	*Glycine soja*	II
Fabaceae	*Glycyrrhiza*	*Glycyrrhiza uralensis*	II
Fabaceae	*Ormosia*	*Ormosia hosiei*	II
Fagaceae	*Fagus*	*Fagus hayatae*	II
Fagaceae	*Quercus*	*Quercus oxyphylla*	II
Gesneriaceae	*Petrocosmea*	*Petrocosmea qinlingensis*	II
Ginkgoaceae	*Ginkgo*	*Ginkgo biloba*	I
Hamamelidaceae	*Parrotia*	*Parrotia subaequalis*	I
Hydrocharitaceae	*Ottelia*	*Ottelia alismoides*	II
Lauraceae	*Cinnamomum*	*Cinnamomum longepaniculatum*	II
Lauraceae	*Machilus*	*Machilus nanmu*	II
Lauraceae	*Phoebe*	*Phoebe bournei*	II
Lauraceae	*Phoebe*	*Phoebe zhennan*	II
Lauraceae	*Phoebe*	*Phoebe hui*	II
Liliaceae	*Cardiocrinum*	*Cardiocrinum cathayanum*	II

Liliaceae	*Fritillaria*	*Fritillaria cirrhosa*	II
Liliaceae	*Fritillaria*	*Fritillaria pallidiflora*	II
Liliaceae	*Fritillaria*	*Fritillaria taipaiensis*	II
Liliaceae	*Fritillaria*	*Fritillaria thunbergii*	II
Liliaceae	*Lilium*	*Lilium fargesii*	II
Liliaceae	*Lilium*	*Lilium papilliferum*	II
Lycopodiaceae	*Huperzia*	*Huperzia appressa*	II
Lycopodiaceae	*Huperzia*	*Huperzia chinensis*	II
Lycopodiaceae	*Huperzia*	*Huperzia serrata*	II
Lythraceae	*Lagerstroemia*	*Lagerstroemia villosa*	II
Magnoliaceae	*Houpoea*	*Houpoea officinalis*	II
Magnoliaceae	*Liriodendron*	*Liriodendron chinense*	II
Magnoliaceae	*Michelia*	*Michelia wilsonii*	II
Magnoliaceae	*Yulania*	*Yulania zenii*	II
Melanthiaceae	*Paris*	*Paris bashanensis*	II
Melanthiaceae	*Paris*	*Paris fargesii*	II
Melanthiaceae	*Paris*	*Paris fargesii var. petiolata*	II
Melanthiaceae	*Paris*	*Paris polyphylla*	II
Melanthiaceae	*Paris*	*Paris polyphylla var. chinensis*	II
Melanthiaceae	*Paris*	*Paris polyphylla var. latifolia*	II
Melanthiaceae	*Paris*	*Paris polyphylla var. stenophylla*	II
Melanthiaceae	*Paris*	*Paris thibetica*	II
Melanthiaceae	*Paris*	*Paris thibetica var. apetala*	II
Melanthiaceae	*Paris*	*Paris polyphylla var. yunnanensis*	II
Nelumbonaceae	*Nelumbo*	*Nelumbo nucifera*	II
Nyssaceae	*Davidia*	*Davidia involucrata*	II
Oleaceae	*Fraxinus*	*Fraxinus mandshurica*	II
Oleaceae	*Osmanthus*	*Osmanthus venosus*	II
Orchidaceae	*Bletilla*	*Bletilla striata*	II
Orchidaceae	*Changnienia*	*Changnienia amoena*	II
Orchidaceae	*Cymbidium*	*Cymbidium goeringii*	II
Orchidaceae	*Cymbidium*	*Cymbidium faberi*	II
Orchidaceae	*Cymbidium*	*Cymbidium macrorhizon*	II
Orchidaceae	*Cymbidium*	*Cymbidium serratum*	II
Orchidaceae	*Cypripedium*	*Cypripedium fasciolatum*	II
Orchidaceae	*Cypripedium*	*Cypripedium*	II

		franchetii	
Orchidaceae	*Cypripedium*	*Cypripedium guttatum*	II
Orchidaceae	*Cypripedium*	*Cypripedium macranthos*	II
Orchidaceae	*Cypripedium*	*Cypripedium taibaiense*	II
Orchidaceae	*Cypripedium*	*Cypripedium henryi*	II
Orchidaceae	*Cypripedium*	*Cypripedium japonicum*	II
Orchidaceae	*Dendrobium*	*Dendrobium flexicaule*	I
Orchidaceae	*Dendrobium*	*Dendrobium hancockii*	II
Orchidaceae	*Dendrobium*	*Dendrobium moniliforme*	II
Orchidaceae	*Gastrodia*	*Gastrodia elata*	II
Orchidaceae	*Gymnadenia*	*Gymnadenia conopsea*	II
Orchidaceae	*Gymnadenia*	*Gymnadenia orchidis*	II
Orchidaceae	*Phalaenopsis*	*Phalaenopsis wilsonii*	II
Orchidaceae	*Phalaenopsis*	*Phalaenopsis zhejiangensis*	I
Orchidaceae	*Pleione*	*Pleione bulbocodioides*	II
Paeoniaceae	*Paeonia*	*Paeonia jishanensis*	II
Paeoniaceae	*Paeonia*	*Paeonia ostii*	II
Paeoniaceae	*Paeonia*	*Paeonia rockii*	I
Paeoniaceae	*Paeonia*	*Paeonia rockii subsp. taibaishanica*	II
Papaveraceae	*Corydalis*	*Corydalis saxicola*	II
Pinaceae	*Abies*	*Abies chensiensis*	II
Pinaceae	*Picea*	*Picea neoveitchii*	II
Pinaceae	*Pseudolarix*	*Pseudolarix amabilis*	II
Pinaceae	*Pseudotsuga*	*Pseudotsuga sinensis*	II
Poaceae	*Agropyron*	*Agropyron mongolicum*	II
Poaceae	*Aristida*	*Aristida triseta*	II
Poaceae	*Psathyrostachys*	*Psathyrostachys huashanica*	I
Podocarpaceae	*Podocarpus*	*Podocarpus macrophyllus*	II
Podocarpaceae	*Podocarpus*	*Podocarpus chinensis*	II
Polygonaceae	*Fagopyrum*	*Fagopyrum dibotrys*	II
Ranunculaceae	*Coptis*	*Coptis chinensis*	II
Rhamnaceae	*Berchemia*	*Berchemiella wilsonii*	II

Rosaceae	*Prunus*	*Prunus mongolica*	II
Rosaceae	*Rosa*	*Rosa rugosa*	II
Rubiaceae	*Emmenopterys*	*Emmenopterys henryi*	II
Rutaceae	*Citrus*	*Citrus cavaleriei*	II
Sapindaceae	*Acer*	*Acer miaotaiense*	II
Scheuchzeriaceae	*Scheuchzeria*	*Scheuchzeria palustris*	II
Scrophulariaceae	*Scrophularia*	*Scrophularia stylosa*	II
Solanaceae	*Lycium*	*Lycium ruthenicum*	II
Styracaceae	*Sinojackia*	*Sinojackia xylocarpa*	II
Taxaceae	*Amentotaxus*	*Amentotaxus argotaenia*	II
Taxaceae	*Taxus*	*Taxus cuspidata*	I
Taxaceae	*Taxus*	*Taxus wallichiana var. chinensis*	I
Taxaceae	*Taxus*	*Taxus wallichiana var. mairei*	I
Taxaceae	*Torreya*	*Torreya fargesii*	II
Theaceae	*Camellia*	*Camellia sinensis*	II
Trochodendraceae	*Tetracentron*	*Tetracentron sinense*	II
Ulmaceae	*Ulmus*	*Ulmus elongata*	II
Ulmaceae	*Zelkova*	*Zelkova schneideriana*	II

Family	Genus	Species	Threatened level
Adoxaceae	*Viburnum*	*Viburnum kansuense*	VU
Alismataceae	*Sagittaria*	*Sagittaria guayanensis subsp. Lappula*	EN
Amaryllidaceae	*Allium*	*Allium funckiifolium*	VU
Apiaceae	*Glehnia*	*Glehnia littoralis*	CR
Apiaceae	*Peucedanum*	*Peucedanum ampliatum*	CR
Apocynaceae	*Ceropegia*	*Ceropegia paohsingensis*	VU
Araliaceae	*Aralia*	*Aralia continentalis*	VU
Araliaceae	*Gamblea*	*Gamblea ciliata var. evodiifolia*	VU
Araliaceae	*Panax*	*Panax ginseng*	CR
Araliaceae	*Panax*	*Panax bipinnatifidus*	EN
Aristolochiaceae	*Asarum*	*Asarum himalaicum*	VU
Aristolochiaceae	*Asarum*	*Asarum sieboldii*	VU
Aristolochiaceae	*Saruma*	*Saruma henryi*	EN
Balsaminaceae	*Impatiens*	*Impatiens nasuta*	VU
Berberidaceae	*Dysosma*	*Dysosma versipellis*	VU
Berberidaceae	*Epimedium*	*Epimedium fargesii*	EN
Berberidaceae	*Epimedium*	*Epimedium ilicifolium*	EN
Berberidaceae	*Mahonia*	*Mahonia conferta*	VU

Buxaceae	*Buxus*	*Buxus ichangensis*	CR
Cabombaceae	*Brasenia*	*Brasenia schreberi*	CR
Calycanthaceae	*Calycanthus*	*Calycanthus chinensis*	EN
Campanulaceae	*Codonopsis*	*Codonopsis cardiophylla*	VU
Campanulaceae	*Codonopsis*	*Codonopsis tsinlingensis*	VU
Caprifoliaceae	*Dipelta*	*Dipelta yunnanensis*	VU
Caprifoliaceae	*Kolkwitzia*	*Kolkwitzia amabilis*	VU
Caryophyllaceae	*Cerastium*	*Cerastium limprichtii*	VU
Celastraceae	*Euonymus*	*Euonymus szechuanensis*	VU
Circaeasteraceae	*Kingdonia*	*Kingdonia uniflora*	VU
Crassulaceae	*Hylotelephium*	*Hylotelephium angustum var. longipedunculum*	VU
Crassulaceae	*Sinocrassula*	*Sinocrassula indica var. viridiflora*	VU
Cucurbitaceae	*Gynostemma*	*Gynostemma cardiospermum*	EN
Cucurbitaceae	*Hemsleya*	*Hemsleya graciliflora*	VU
Cupressaceae	*Metasequoia*	*Metasequoia glyptostroboides*	EN
Cupressaceae	*Thuja*	*Thuja sutchuenensis*	EN
Cycadaceae	*Cycas*	*Cycas revoluta*	CR
Cynomoriaceae	*Cynomorium*	*Cynomorium songaricum*	VU
Cyperaceae	*Carex*	*Carex shaanxiensis*	VU
Dioscoreaceae	*Dioscorea*	*Dioscorea althaeoides*	VU
Elaeagnaceae	*Elaeagnus*	*Elaeagnus mollis*	EN
Ericaceae	*Pyrola*	*Pyrola rugosa*	EN
Ericaceae	*Rhododendron*	*Rhododendron detersile*	VU
Ericaceae	*Rhododendron*	*Rhododendron purdomii*	VU
Eucommiaceae	*Eucommia*	*Eucommia ulmoides*	VU
Fabaceae	*Ammopiptanthus*	*Ammopiptanthus mongolicus*	VU
Fabaceae	*Astragalus*	*Astragalus henryi*	VU
Fabaceae	*Astragalus*	*Astragalus mongholicus*	VU
Fabaceae	*Astragalus*	*Astragalus taiyuanensis*	EN
Fabaceae	*Caragana*	*Caragana purdomii*	VU
Fabaceae	*Caragana*	*Caragana stipitata*	EN
Fabaceae	*Hedysarum*	*Hedysarum taipeicum*	EN
Fabaceae	*Ormosia*	*Ormosia hosiei*	EN
Gentianaceae	*Gentiana*	*Gentiana manshurica*	EN
Gesneriaceae	*Petrocosmea*	*Petrocosmea qinlingensis*	CR
Ginkgoaceae	*Ginkgo*	*Ginkgo biloba*	CR
Hamamelidaceae	*Corylopsis*	*Corylopsis microcarpa*	EN
Hamamelidaceae	*Distylium*	*Distylium chinense*	EN
Hamamelidaceae	*Fortunearia*	*Fortunearia sinensis*	VU
Hamamelidaceae	*Parrotia*	*Parrotia subaequalis*	CR
Hamamelidaceae	*Sinowilsonia*	*Sinowilsonia henryi*	VU
Hydrangeaceae	*Deinanthe*	*Deinanthe caerulea*	VU

Hydrocharitaceae	*Ottelia*	*Ottelia alismoides*	VU
Juglandaceae	*Juglans*	*Juglans regia*	VU
Lauraceae	*Actinodaphne*	*Actinodaphne obscurinervia*	EN
Lauraceae	*Machilus*	*Machilus nanmu*	EN
Lauraceae	*Phoebe*	*Phoebe bournei*	VU
Lauraceae	*Phoebe*	*Phoebe zhennan*	VU
Liliaceae	*Fritillaria*	*Fritillaria pallidiflora*	VU
Liliaceae	*Fritillaria*	*Fritillaria taipaiensis*	EN
Liliaceae	*Lilium*	*Lilium leichtlinii var. maximowiczii*	VU
Lycopodiaceae	*Huperzia*	*Huperzia serrata*	EN
Lythraceae	*Lagerstroemia*	*Lagerstroemia villosa*	VU
Magnoliaceae	*Manglietia*	*Manglietia insignis*	VU
Magnoliaceae	*Michelia*	*Michelia wilsonii*	VU
Magnoliaceae	*Yulania*	*Yulania amoena*	VU
Magnoliaceae	*Yulania*	*Yulania campbellii*	VU
Magnoliaceae	*Yulania*	*Yulania dawsoniana*	EN
Magnoliaceae	*Yulania*	*Yulania liliiflora*	VU
Magnoliaceae	*Yulania*	*Yulania sargentiana*	VU
Magnoliaceae	*Yulania*	*Yulania viridula*	EN
Magnoliaceae	*Yulania*	*Yulania zenii*	CR
Malvaceae	*Hibiscus*	*Hibiscus leviseminus*	VU
Melanthiaceae	*Paris*	*Paris fargesii var. petiolata*	EN
Melanthiaceae	*Paris*	*Paris polyphylla var. chinensis*	VU
Menispermaceae	*Tinospora*	*Tinospora sagittata*	EN
Oleaceae	*Fraxinus*	*Fraxinus baroniana*	EN
Oleaceae	*Fraxinus*	*Fraxinus mandshurica*	VU
Oleaceae	*Osmanthus*	*Osmanthus venosus*	EN
Orchidaceae	*Bletilla*	*Bletilla formosana*	EN
Orchidaceae	*Bletilla*	*Bletilla ochracea*	EN
Orchidaceae	*Bletilla*	*Bletilla striata*	EN
Orchidaceae	*Bulbophyllum*	*Bulbophyllum chondriophorum*	VU
Orchidaceae	*Bulbophyllum*	*Bulbophyllum henanense*	VU
Orchidaceae	*Calanthe*	*Calanthe arcuata*	VU
Orchidaceae	*Calanthe*	*Calanthe emeishanica*	CR
Orchidaceae	*Calanthe*	*Calanthe yaoshanensis*	EN
Orchidaceae	*Calypso*	*Calypso bulbosa var. speciosa*	VU
Orchidaceae	*Changnienia*	*Changnienia amoena*	EN
Orchidaceae	*Cymbidium*	*Cymbidium goeringii*	VU
Orchidaceae	*Cypripedium*	*Cypripedium fasciolatum*	EN
Orchidaceae	*Cypripedium*	*Cypripedium franchetii*	VU
Orchidaceae	*Cypripedium*	*Cypripedium guttatum*	EN
Orchidaceae	*Cypripedium*	*Cypripedium macranthos*	EN
Orchidaceae	*Cypripedium*	*Cypripedium taibaiense*	EN

Orchidaceae	*Dendrobium*	*Dendrobium catenatum*	CR
Orchidaceae	*Dendrobium*	*Dendrobium flexicaule*	CR
Orchidaceae	*Dendrobium*	*Dendrobium hancockii*	EN
Orchidaceae	*Epipogium*	*Epipogium aphyllum*	EN
Orchidaceae	*Eulophia*	*Eulophia dabia*	VU
Orchidaceae	*Galearis*	*Galearis tschiliensis*	VU
Orchidaceae	*Goodyera*	*Goodyera wolongensis*	VU
Orchidaceae	*Gymnadenia*	*Gymnadenia conopsea*	EN
Orchidaceae	*Gymnadenia*	*Gymnadenia orchidis*	VU
Orchidaceae	*Habenaria*	*Habenaria fargesii*	VU
Orchidaceae	*Kuhlhasseltia*	*Kuhlhasseltia yakushimensis*	VU
Orchidaceae	*Liparis*	*Liparis campylostalix*	VU
Orchidaceae	*Myrmechis*	*Myrmechis chinensis*	VU
Orchidaceae	*Neofinetia*	*Neofinetia richardsiana*	CR
Orchidaceae	*Neottia*	*Neottia oblata*	EN
Orchidaceae	*Neottia*	*Neottia papilligera*	EN
Orchidaceae	*Neottia*	*Neottia puberula var. maculata*	VU
Orchidaceae	*Neottianthe*	*Neottianthe cucullata var. cucullata/Neottianthe cucullata*	VU
Orchidaceae	*Nothodoritis*	*Nothodoritis zhejiangensis*	EN
Orchidaceae	*Oberonia*	*Oberonia sinica*	EN
Orchidaceae	*Phalaenopsis*	*Phalaenopsis wilsonii*	VU
Orchidaceae	*Tipularia*	*Tipularia szechuanica*	VU
Orobanchaceae	*Gleadovia*	*Gleadovia ruborum*	VU
Orobanchaceae	*Pedicularis*	*Pedicularis bicolor*	VU
Orobanchaceae	*Pedicularis*	*Pedicularis odontochila*	EN
Paeoniaceae	*Paeonia*	*Paeonia jishanensis*	VU
Paeoniaceae	*Paeonia*	*Paeonia ostii*	VU
Paeoniaceae	*Paeonia*	*Paeonia rockii*	VU
Paeoniaceae	*Paeonia*	*Paeonia rockii subsp. taibaishanica*	VU
Paeoniaceae	*Paeonia*	*Paeonia suffruticosa*	VU
Papaveraceae	*Corydalis*	*Corydalis anethifolia*	EN
Papaveraceae	*Corydalis*	*Corydalis yanhusuo*	VU
Papaveraceae	*Meconopsis*	*Meconopsis quintuplinervia var. glabra*	VU
Pinaceae	*Abies*	*Abies chensiensis*	VU
Pinaceae	*Larix*	*Larix gmelinii var. principis-rupprechtii*	VU
Pinaceae	*Pinus*	*Pinus sylvestris var. mongolica*	VU
Pinaceae	*Pinus*	*Pinus tabuliformis var. henryi*	VU
Pinaceae	*Pseudolarix*	*Pseudolarix amabilis*	VU

Poaceae	*Chimonobambusa*	*Chimonobambusa marmorea*	VU
Poaceae	*Psathyrostachys*	*Psathyrostachys huashanica*	CR
Podocarpaceae	*Podocarpus*	*Podocarpus macrophyllus var. macrophyllus/Podocarpus macrophyllus*	VU
Polygonaceae	*Rheum*	*Rheum tanguticum var. tanguticum/Rheum tanguticum*	VU
Primulaceae	*Primula*	*Primula filchnerae*	EN
Primulaceae	*Primula*	*Primula rupestris*	CR
Primulaceae	*Primula*	*Primula sinensis*	EN
Psilotaceae	*Psilotum*	*Psilotum nudum*	VU
Ranunculaceae	*Aconitum*	*Aconitum taipeicum*	EN
Ranunculaceae	*Callianthemum*	*Callianthemum taipaicum*	EN
Ranunculaceae	*Coptis*	*Coptis chinensis*	VU
Ranunculaceae	*Helleborus*	*Helleborus thibetanus*	VU
Ranunculaceae	*Hepatica*	*Hepatica henryi*	VU
Rosaceae	*Amygdalus*	*Amygdalus mongolica*	VU
Rosaceae	*Physocarpus*	*Physocarpus amurensis*	VU
Rosaceae	*Rosa*	*Rosa rugosa*	EN
Rosaceae	*Sorbus*	*Sorbus tsinlingensis*	VU
Rosaceae	*Sorbus*	*Sorbus yuana*	VU
Rutaceae	*Citrus*	*Citrus japonica*	EN
Rutaceae	*Zanthoxylum*	*Zanthoxylum molle*	VU
Salicaceae	*Populus*	*Populus pseudomaximowiczii*	EN
Salicaceae	*Salix*	*Salix permollis*	CR
Salicaceae	*Salix*	*Salix pingliensis*	VU
Salicaceae	*Salix*	*Salix pseudopermollis*	VU
Salicaceae	*Salix*	*Salix pseudotangii*	VU
Salicaceae	*Salix*	*Salix yuhuangshanensis*	EN
Sapindaceae	*Acer*	*Acer cappadocicum subsp. sinicum*	VU
Sapindaceae	*Acer*	*Acer fulvescens*	VU
Sapindaceae	*Acer*	*Acer griseum*	VU
Sapindaceae	*Acer*	*Acer mandshuricum*	VU
Sapindaceae	*Acer*	*Acer miaotaiense*	VU
Sapindaceae	*Acer*	*Acer palmatum*	VU
Sapindaceae	*Acer*	*Acer pilosum*	VU
Sapindaceae	*Acer*	*Acer tsinglingense*	VU
Saxifragaceae	*Bergenia*	*Bergenia scopulosa*	VU
Saxifragaceae	*Chrysosplenium*	*Chrysosplenium taibaishanense*	EN
Scheuchzeriaceae	*Scheuchzeria*	*Scheuchzeria palustris*	VU
Schisandraceae	*Kadsura*	*Kadsura coccinea*	VU

Scrophulariaceae	*Scrophularia*	*Scrophularia stylosa*	VU
Solanaceae	*Physochlaina*	*Physochlaina infundibularis*	VU
Styracaceae	*Sinojackia*	*Sinojackia xylocarpa*	EN
Taxaceae	*Taxus*	*Taxus cuspidata*	EN
Taxaceae	*Taxus*	*Taxus wallichiana var. chinensis*	VU
Taxaceae	*Taxus*	*Taxus wallichiana var. mairei*	VU
Taxaceae	*Torreya*	*Torreya fargesii*	VU
Thymelaeaceae	*Daphne*	*Daphne gemmata*	VU
Ulmaceae	*Ulmus*	*Ulmus elongata*	EN
Violaceae	*Viola*	*Viola diamantiaca*	VU
Vitaceae	*Vitis*	*Vitis bashanica*	CR

Family	Genus	Species
Acanthaceae	*Gymnostachyum*	*Gymnostachyum subrosulatum*
Actinidiaceae	*Actinidia*	*Actinidia chinensis*
Actinidiaceae	*Actinidia*	*Actinidia tetramera*
Adoxaceae	*Viburnum*	*Viburnum kansuense*
Adoxaceae	*Sambucus*	*Sambucus williamsii*
Adoxaceae	*Viburnum*	*Viburnum betulifolium*
Adoxaceae	*Viburnum*	*Viburnum chinshanense*
Adoxaceae	*Viburnum*	*Viburnum davidii*
Adoxaceae	*Viburnum*	*Viburnum farreri*
Adoxaceae	*Viburnum*	*Viburnum foetidum var. rectangulatum*
Adoxaceae	*Viburnum*	*Viburnum henryi*
Adoxaceae	*Viburnum*	*Viburnum oliganthum*
Adoxaceae	*Viburnum*	*Viburnum propinquum*
Adoxaceae	*Viburnum*	*Viburnum schensianum*
Adoxaceae	*Viburnum*	*Viburnum setigerum*
Adoxaceae	*Viburnum*	*Viburnum sympodiale*
Adoxaceae	*Viburnum*	*Viburnum utile*
Amaranthaceae	*Chenopodium*	*Chenopodium urbicum subsp. Sinicum*
Amaranthaceae	*Corispermum*	*Corispermum stauntonii*
Amaryllidaceae	*Allium*	*Allium funckiifolium*
Amaryllidaceae	*Lycoris*	*Lycoris shaanxiensis*
Amaryllidaceae	*Lycoris*	*Lycoris sprengeri*
Amaryllidaceae	*Allium*	*Allium anisopodium var. zimmermannianum*
Amaryllidaceae	*Allium*	*Allium chrysanthum*

Amaryllidaceae	*Allium*	*Allium dentigerum*
Amaryllidaceae	*Allium*	*Allium funckiifolium*
Amaryllidaceae	*Allium*	*Allium listera*
Amaryllidaceae	*Allium*	*Allium ovalifolium*
Amaryllidaceae	*Allium*	*Allium paepalanthoides*
Amaryllidaceae	*Allium*	*Allium plurifoliatum*
Amaryllidaceae	*Allium*	*Allium tubiflorum*
Amaryllidaceae	*Allium*	*Allium yanchiense*
Anacardiaceae	*Cotinus*	*Cotinus coggygria var. glaucophylla*
Anacardiaceae	*Pistacia*	*Pistacia chinensis*
Anacardiaceae	*Rhus*	*Rhus potaninii*
Anacardiaceae	*Rhus*	*Rhus punjabensis var. sinica*
Anacardiaceae	*Toxicodendron*	*Toxicodendron radicans subsp. Hispidum*
Apiaceae	*Peucedanum*	*Peucedanum ampliatum*
Apiaceae	*Aegopodium*	*Aegopodium henryi*
Apiaceae	*Angelica*	*Angelica biserrata*
Apiaceae	*Angelica*	*Angelica dielsii*
Apiaceae	*Angelica*	*Angelica fargesii*
Apiaceae	*Angelica*	*Angelica laxifoliata*
Apiaceae	*Angelica*	*Angelica pseudoselinum*
Apiaceae	*Angelica*	*Angelica tsinlingensis*
Apiaceae	*Bupleurum*	*Bupleurum chinense*
Apiaceae	*Bupleurum*	*Bupleurum dielsianum*
Apiaceae	*Bupleurum*	*Bupleurum longicaule var. franchetii*
Apiaceae	*Bupleurum*	*Bupleurum longicaule var. giraldii*
Apiaceae	*Bupleurum*	*Bupleurum microcephalum*
Apiaceae	*Bupleurum*	*Bupleurum smithii*
Apiaceae	*Bupleurum*	*Bupleurum yinchowense*
Apiaceae	*Cnidium*	*Cnidium sinchianum*
Apiaceae	*Ferula*	*Ferula licentiana*
Apiaceae	*Heracleum*	*Heracleum fargesii*
Apiaceae	*Libanotis*	*Libanotis lancifolia*
Apiaceae	*Libanotis*	*Libanotis spodotrichoma*
Apiaceae	*Ligusticum*	*Ligusticum acuminatum*
Apiaceae	*Ligusticum*	*Ligusticum angelicifolium*
Apiaceae	*Ligusticum*	*Ligusticum brachylobum*
Apiaceae	*Ligusticum*	*Ligusticum nullivittatum*
Apiaceae	*Ligusticum*	*Ligusticum oliverianum*
Apiaceae	*Ligusticum*	*Ligusticum sinense*
Apiaceae	*Ligusticum*	*Ligusticum tenuisectum*

Apiaceae	*Melanosciadium*	*Melanosciadium pimpinelloideum*
Apiaceae	*Nothosmyrnium*	*Nothosmyrnium japonicum var. sutchuensis*
Apiaceae	*Notopterygium*	*Notopterygium franchetii*
Apiaceae	*Notopterygium*	*Notopterygium incisum*
Apiaceae	*Notopterygium*	*Notopterygium oviforme*
Apiaceae	*Peucedanum*	*Peucedanum harry-smithii var. grande*
Apiaceae	*Peucedanum*	*Peucedanum harry-smithii*
Apiaceae	*Peucedanum*	*Peucedanum ledebourielloides*
Apiaceae	*Peucedanum*	*Peucedanum praeruptorum*
Apiaceae	*Pimpinella*	*Pimpinella arguta*
Apiaceae	*Pimpinella*	*Pimpinella diversifolia var. angustipetala*
Apiaceae	*Pimpinella*	*Pimpinella fargesii*
Apiaceae	*Pimpinella*	*Pimpinella henryi*
Apiaceae	*Pimpinella*	*Pimpinella rhomboidea*
Apiaceae	*Pimpinella*	*Pimpinella smithii*
Apiaceae	*Pimpinella*	*Pimpinella valleculosa*
Apiaceae	*Pleurospermum*	*Pleurospermum cristatum*
Apiaceae	*Pleurospermum*	*Pleurospermum franchetianum*
Apiaceae	*Pleurospermum*	*Pleurospermum giraldii*
Apiaceae	*Pternopetalum*	*Pternopetalum caespitosum*
Apiaceae	*Pternopetalum*	*Pternopetalum davidii*
Apiaceae	*Pternopetalum*	*Pternopetalum molle*
Apiaceae	*Sanicula*	*Sanicula elongata*
Apiaceae	*Sanicula*	*Sanicula giraldii*
Apiaceae	*Sanicula*	*Sanicula serrata*
Apiaceae	*Tongoloa*	*Tongoloa elata*
Apiaceae	*Tongoloa*	*Tongoloa silaifolia*
Apocynaceae	*Ceropegia*	*Ceropegia paohsingensis*
Apocynaceae	*Sindechites*	*Sindechites henryi*
Apocynaceae	*Trachelospermum*	*Trachelospermum axillare*
Apocynaceae	*Biondia*	*Biondia chinensis*
Apocynaceae	*Biondia*	*Biondia hemsleyana*
Apocynaceae	*Biondia*	*Biondia henryi*
Apocynaceae	*Biondia*	*Biondia insignis*
Apocynaceae	*Cynanchum*	*Cynanchum forrestii*
Apocynaceae	*Cynanchum*	*Cynanchum giraldii*
Apocynaceae	*Cynanchum*	*Cynanchum glaucescens*
Apocynaceae	*Cynanchum*	*Cynanchum mongolicum*
Apocynaceae	*Cynanchum*	*Cynanchum officinale*

Apocynaceae	*Cynanchum*	*Cynanchum taihangense*
Apocynaceae	*Dregea*	*Dregea sinensis var. corrugata*
Apocynaceae	*Dregea*	*Dregea sinensis*
Apocynaceae	*Dregea*	*Dregea yunnanensis*
Apocynaceae	*Marsdenia*	*Marsdenia oreophila*
Apocynaceae	*Metaplexis*	*Metaplexis hemsleyana*
Apocynaceae	*Periploca*	*Periploca sepium*
Apocynaceae	*Tylophora*	*Tylophora henryi*
Aquifoliaceae	*Ilex*	*Ilex corallina*
Aquifoliaceae	*Ilex*	*Ilex dunniana*
Aquifoliaceae	*Ilex*	*Ilex fargesii var. angustifolia*
Aquifoliaceae	*Ilex*	*Ilex fargesii*
Aquifoliaceae	*Ilex*	*Ilex franchetiana var. parvifolia*
Aquifoliaceae	*Ilex*	*Ilex hylonoma*
Aquifoliaceae	*Ilex*	*Ilex intermedia*
Aquifoliaceae	*Ilex*	*Ilex macrocarpa var. longipedunculata*
Aquifoliaceae	*Ilex*	*Ilex macrocarpa*
Aquifoliaceae	*Ilex*	*Ilex macrocarpa var. reevesae*
Aquifoliaceae	*Ilex*	*Ilex pernyi*
Aquifoliaceae	*Ilex*	*Ilex szechwanensis*
Aquifoliaceae	*Ilex*	*Ilex wilsonii*
Aquifoliaceae	*Ilex*	*Ilex yunnanensis var. gentilis*
Araceae	*Amorphophallus*	*Amorphophallus konjac*
Araceae	*Arisaema*	*Arisaema asperatum*
Araceae	*Arisaema*	*Arisaema lobatum*
Araceae	*Arisaema*	*Arisaema silvestrii*
Araceae	*Arisaema*	*Arisaema wardii*
Araceae	*Pinellia*	*Pinellia cordata*
Araliaceae	*Aralia*	*Aralia fargesii*
Araliaceae	*Aralia*	*Aralia henryi*
Araliaceae	*Aralia*	*Aralia officinalis*
Araliaceae	*Eleutherococcus*	*Eleutherococcus brachypus*
Araliaceae	*Eleutherococcus*	*Eleutherococcus eleutheristylus*
Araliaceae	*Eleutherococcus*	*Eleutherococcus henryi var. faberi*
Araliaceae	*Eleutherococcus*	*Eleutherococcus henryi*
Araliaceae	*Eleutherococcus*	*Eleutherococcus leucorrhizus var. fulvescens*
Araliaceae	*Eleutherococcus*	*Eleutherococcus leucorrhizus var. scaberulus*
Araliaceae	*Eleutherococcus*	*Eleutherococcus nodiflorus*

Araliaceae	*Eleutherococcus*	*Eleutherococcus rehderianus*
Araliaceae	*Eleutherococcus*	*Eleutherococcus wilsonii*
Araliaceae	*Macropanax*	*Macropanax rosthornii*
Araliaceae	*Tetrapanax*	*Tetrapanax papyrifer*
Aristolochiaceae	*Saruma*	*Saruma henryi*
Aristolochiaceae	*Asarum*	*Asarum debile*
Aristolochiaceae	*Asarum*	*Asarum forbesii*
Asparagaceae	*Asparagus*	*Asparagus longiflorus*
Asparagaceae	*Campylandra*	*Campylandra chinensis*
Asparagaceae	*Diuranthera*	*Diuranthera chinglingensis*
Asparagaceae	*Liriope*	*Liriope graminifolia*
Asparagaceae	*Liriope*	*Liriope kansuensis*
Asparagaceae	*Liriope*	*Liriope longipedicellata*
Asparagaceae	*Maianthemum*	*Maianthemum lichiangense*
Asparagaceae	*Maianthemum*	*Maianthemum stenolobum*
Asparagaceae	*Maianthemum*	*Maianthemum tubiferum*
Asparagaceae	*Polygonatum*	*Polygonatum curvistylum*
Asparagaceae	*Polygonatum*	*Polygonatum cyrtonema*
Asparagaceae	*Polygonatum*	*Polygonatum franchetii*
Asparagaceae	*Polygonatum*	*Polygonatum gracile*
Asparagaceae	*Polygonatum*	*Polygonatum megaphyllum*
Asparagaceae	*Polygonatum*	*Polygonatum nodosum*
Asparagaceae	*Polygonatum*	*Polygonatum zanlanscianense*
Asphodelaceae	*Eremurus*	*Eremurus chinensis*
Aspleniaceae	*Asplenium*	*Asplenium humistratum*
Asteraceae	*Achillea*	*Achillea wilsoniana*
Asteraceae	*Ainsliaea*	*Ainsliaea angustata*
Asteraceae	*Ainsliaea*	*Ainsliaea gracilis*
Asteraceae	*Ainsliaea*	*Ainsliaea henryi*
Asteraceae	*Ainsliaea*	*Ainsliaea ramosa*
Asteraceae	*Ajania*	*Ajania ramosa*
Asteraceae	*Ajania*	*Ajania remotipinna*
Asteraceae	*Ajania*	*Ajania salicifolia*
Asteraceae	*Anaphalis*	*Anaphalis aureopunctata*
Asteraceae	*Anaphalis*	*Anaphalis aureopunctata var. plantaginifolia*
Asteraceae	*Anaphalis*	*Anaphalis aureopunctata var. tomentosa*
Asteraceae	*Anaphalis*	*Anaphalis flavescens*
Asteraceae	*Anaphalis*	*Anaphalis flavescens var. taipeiensis*
Asteraceae	*Anaphalis*	*Anaphalis hancockii*
Asteraceae	*Anaphalis*	*Anaphalis sinica var. densata*
Asteraceae	*Artemisia*	*Artemisia deversa*

Asteraceae	*Artemisia*	*Artemisia frigida var. atropurpurea*
Asteraceae	*Artemisia*	*Artemisia gansuensis*
Asteraceae	*Artemisia*	*Artemisia giraldii*
Asteraceae	*Artemisia*	*Artemisia igniaria*
Asteraceae	*Artemisia*	*Artemisia kanashiroi*
Asteraceae	*Artemisia*	*Artemisia lactiflora var. incisa*
Asteraceae	*Artemisia*	*Artemisia lactiflora var. taibaishanensis*
Asteraceae	*Artemisia*	*Artemisia qinlingensis*
Asteraceae	*Artemisia*	*Artemisia shangnanensis*
Asteraceae	*Artemisia*	*Artemisia taibaishanensis*
Asteraceae	*Artemisia*	*Artemisia velutina*
Asteraceae	*Artemisia*	*Artemisia verbenacea*
Asteraceae	*Aster*	*Aster ageratoides var. heterophyllus*
Asteraceae	*Aster*	*Aster ageratoides var. laticorymbus*
Asteraceae	*Aster*	*Aster ageratoides var. pilosus*
Asteraceae	*Aster*	*Aster falcifolius*
Asteraceae	*Aster*	*Aster flaccidus subsp. flaccidus*
Asteraceae	*Aster*	*Aster giraldii*
Asteraceae	*Aster*	*Aster poliothamnus*
Asteraceae	*Aster*	*Aster sikuensis*
Asteraceae	*Aster*	*Aster smithianus*
Asteraceae	*Carpesium*	*Carpesium lipskyi*
Asteraceae	*Carpesium*	*Carpesium longifolium*
Asteraceae	*Carpesium*	*Carpesium szechuanense*
Asteraceae	*Chrysanthemum*	*Chrysanthemum argyrophyllum*
Asteraceae	*Chrysanthemum*	*Chrysanthemum glabriusculum*
Asteraceae	*Chrysanthemum*	*Chrysanthemum potentilloides*
Asteraceae	*Chrysanthemum*	*Chrysanthemum vestitum*
Asteraceae	*Cirsium*	*Cirsium fargesii*
Asteraceae	*Cirsium*	*Cirsium monocephalum*
Asteraceae	*Doronicum*	*Doronicum gansuense*
Asteraceae	*Erigeron*	*Erigeron taipeiensis*
Asteraceae	*Leontopodium*	*Leontopodium calocephalum*
Asteraceae	*Leontopodium*	*Leontopodium giraldii*
Asteraceae	*Leontopodium*	*Leontopodium japonicum var. microcephalum*
Asteraceae	*Leontopodium*	*Leontopodium smithianum*
Asteraceae	*Ligularia*	*Ligularia achyrotricha*

Asteraceae	*Ligularia*	*Ligularia dolichobotrys*
Asteraceae	*Ligularia*	*Ligularia fargesii*
Asteraceae	*Ligularia*	*Ligularia przewalskii*
Asteraceae	*Ligularia*	*Ligularia tenuipes*
Asteraceae	*Ligularia*	*Ligularia veitchiana*
Asteraceae	*Myripnois*	*Myripnois dioica*
Asteraceae	*Nannoglottis*	*Nannoglottis carpesioides*
Asteraceae	*Notoseris*	*Notoseris melanantha*
Asteraceae	*Olgaea*	*Olgaea tangutica*
Asteraceae	*Parasenecio*	*Parasenecio ambiguus*
Asteraceae	*Parasenecio*	*Parasenecio bulbiferoides*
Asteraceae	*Parasenecio*	*Parasenecio dasythyrsus*
Asteraceae	*Parasenecio*	*Parasenecio gansuensis*
Asteraceae	*Parasenecio*	*Parasenecio hastatus var. glaber*
Asteraceae	*Parasenecio*	*Parasenecio otopteryx*
Asteraceae	*Parasenecio*	*Parasenecio petasitoides*
Asteraceae	*Parasenecio*	*Parasenecio pilgerianus*
Asteraceae	*Parasenecio*	*Parasenecio roborowskii*
Asteraceae	*Parasenecio*	*Parasenecio rufipilis*
Asteraceae	*Parasenecio*	*Parasenecio sinicus*
Asteraceae	*Parasenecio*	*Parasenecio tsinlingensis*
Asteraceae	*Parasenecio*	*Parasenecio xinjiashanensis*
Asteraceae	*Pertya*	*Pertya sinensis*
Asteraceae	*Saussurea*	*Saussurea acromelaena*
Asteraceae	*Saussurea*	*Saussurea acrophila*
Asteraceae	*Saussurea*	*Saussurea alatipes*
Asteraceae	*Saussurea*	*Saussurea baroniana*
Asteraceae	*Saussurea*	*Saussurea bullockii*
Asteraceae	*Saussurea*	*Saussurea cauloptera*
Asteraceae	*Saussurea*	*Saussurea conyzoides*
Asteraceae	*Saussurea*	*Saussurea cordifolia*
Asteraceae	*Saussurea*	*Saussurea dielsiana*
Asteraceae	*Saussurea*	*Saussurea dolichopoda*
Asteraceae	*Saussurea*	*Saussurea flaccida*
Asteraceae	*Saussurea*	*Saussurea flexuosa*
Asteraceae	*Saussurea*	*Saussurea globosa*
Asteraceae	*Saussurea*	*Saussurea henryi*
Asteraceae	*Saussurea*	*Saussurea huashanensis*
Asteraceae	*Saussurea*	*Saussurea iodostegia*
Asteraceae	*Saussurea*	*Saussurea kungii*
Asteraceae	*Saussurea*	*Saussurea licentiana*
Asteraceae	*Saussurea*	*Saussurea macrota*
Asteraceae	*Saussurea*	*Saussurea morifolia*

Asteraceae	*Saussurea*	*Saussurea mutabilis*
Asteraceae	*Saussurea*	*Saussurea nigrescens*
Asteraceae	*Saussurea*	*Saussurea oligantha*
Asteraceae	*Saussurea*	*Saussurea oligocephala*
Asteraceae	*Saussurea*	*Saussurea paucijiuga*
Asteraceae	*Saussurea*	*Saussurea pectinata*
Asteraceae	*Saussurea*	*Saussurea pinetorum*
Asteraceae	*Saussurea*	*Saussurea ploycephala*
Asteraceae	*Saussurea*	*Saussurea populifolia*
Asteraceae	*Saussurea*	*Saussurea rotundifolia*
Asteraceae	*Saussurea*	*Saussurea saligna*
Asteraceae	*Saussurea*	*Saussurea sobarocephala*
Asteraceae	*Saussurea*	*Saussurea sutchuenensis*
Asteraceae	*Saussurea*	*Saussurea sylvatica*
Asteraceae	*Saussurea*	*Saussurea veitchiana*
Asteraceae	*Sinosenecio*	*Sinosenecio globigerus*
Asteraceae	*Sinosenecio*	*Sinosenecio hederifolius*
Asteraceae	*Sinosenecio*	*Sinosenecio villiferus*
Asteraceae	*Taraxacum*	*Taraxacum nutans*
Asteraceae	*Tephroseris*	*Tephroseris pseudosonchus*
Asteraceae	*Tephroseris*	*Tephroseris rufa*
Asteraceae	*Youngia*	*Youngia henryi*
Athyriaceae	*Athyrium*	*Athyrium omeiense*
Athyriaceae	*Athyrium*	*Athyrium sinense*
Athyriaceae	*Deparia*	*Deparia confusa*
Balsaminaceae	*Impatiens*	*Impatiens nasuta*
Balsaminaceae	*Impatiens*	*Impatiens dicentra*
Balsaminaceae	*Impatiens*	*Impatiens fissicornis*
Balsaminaceae	*Impatiens*	*Impatiens lacinulifera*
Balsaminaceae	*Impatiens*	*Impatiens latebracteata*
Balsaminaceae	*Impatiens*	*Impatiens linocentra*
Balsaminaceae	*Impatiens*	*Impatiens notolopha*
Balsaminaceae	*Impatiens*	*Impatiens potaninii*
Balsaminaceae	*Impatiens*	*Impatiens pterosepala*
Balsaminaceae	*Impatiens*	*Impatiens rostellata*
Balsaminaceae	*Impatiens*	*Impatiens stenosepala*
Balsaminaceae	*Impatiens*	*Impatiens sutchuanensis*
Begoniaceae	*Begonia*	*Begonia grandis subsp. Sinensis*
Berberidaceae	*Dysosma*	*Dysosma versipellis*
Berberidaceae	*Epimedium*	*Epimedium fargesii*
Berberidaceae	*Epimedium*	*Epimedium ilicifolium*
Berberidaceae	*Mahonia*	*Mahonia conferta*
Berberidaceae	*Berberis*	*Berberis brachypoda*

Berberidaceae	*Berberis*	*Berberis candidula*
Berberidaceae	*Berberis*	*Berberis circumserrata*
Berberidaceae	*Berberis*	*Berberis dasystachya*
Berberidaceae	*Berberis*	*Berberis diaphana*
Berberidaceae	*Berberis*	*Berberis dictyoneura*
Berberidaceae	*Berberis*	*Berberis dielsiana*
Berberidaceae	*Berberis*	*Berberis feddeana*
Berberidaceae	*Berberis*	*Berberis francisci-ferdinandi*
Berberidaceae	*Berberis*	*Berberis gilgiana*
Berberidaceae	*Berberis*	*Berberis henryana*
Berberidaceae	*Berberis*	*Berberis julianae*
Berberidaceae	*Berberis*	*Berberis kansuensis*
Berberidaceae	*Berberis*	*Berberis potaninii*
Berberidaceae	*Berberis*	*Berberis pubescens*
Berberidaceae	*Berberis*	*Berberis purdomii*
Berberidaceae	*Berberis*	*Berberis reticulata*
Berberidaceae	*Berberis*	*Berberis salicaria*
Berberidaceae	*Berberis*	*Berberis shensiana*
Berberidaceae	*Berberis*	*Berberis soulieana*
Berberidaceae	*Berberis*	*Berberis vernae*
Berberidaceae	*Berberis*	*Berberis virgetorum*
Berberidaceae	*Diphylleia*	*Diphylleia sinensis*
Berberidaceae	*Dysosma*	*Dysosma pleiantha*
Berberidaceae	*Epimedium*	*Epimedium brevicornu*
Berberidaceae	*Epimedium*	*Epimedium pubescens*
Berberidaceae	*Epimedium*	*Epimedium sagittatum*
Berberidaceae	*Epimedium*	*Epimedium sutchuenense*
Berberidaceae	*Epimedium*	*Epimedium wushanense*
Betulaceae	*Alnus*	*Alnus cremastogyne*
Betulaceae	*Betula*	*Betula albosinensis*
Betulaceae	*Betula*	*Betula insignis*
Betulaceae	*Betula*	*Betula luminifera*
Betulaceae	*Carpinus*	*Carpinus cordata var. chinensis*
Betulaceae	*Carpinus*	*Carpinus cordata var. mollis*
Betulaceae	*Carpinus*	*Carpinus fargesiana*
Betulaceae	*Carpinus*	*Carpinus henryana*
Betulaceae	*Carpinus*	*Carpinus hupeana*
Betulaceae	*Carpinus*	*Carpinus polyneura*
Betulaceae	*Carpinus*	*Carpinus shensiensis*
Betulaceae	*Corylus*	*Corylus chinensis*
Betulaceae	*Corylus*	*Corylus fargesii*
Betulaceae	*Corylus*	*Corylus ferox var. thibetica*
Betulaceae	*Corylus*	*Corylus heterophylla var. sutchuenensis*

Betulaceae	*Ostryopsis*	*Ostryopsis davidiana*
Bignoniaceae	*Catalpa*	*Catalpa bungei*
Bignoniaceae	*Catalpa*	*Catalpa fargesii*
Bignoniaceae	*Incarvillea*	*ncarvillea sinensis var. przewalskii*
Boraginaceae	*Microula*	*Microula turbinata*
Boraginaceae	*Sinojohnstonia*	*Sinojohnstonia chekiangensis*
Boraginaceae	*Sinojohnstonia*	*Sinojohnstonia moupinensis*
Boraginaceae	*Trigonotis*	*Trigonotis giraldii*
Boraginaceae	*Trigonotis*	*Trigonotis mollis*
Boraginaceae	*Trigonotis*	*Trigonotis peduncularis var. macrantha*
Boraginaceae	*Trigonotis*	*Trigonotis vestita*
Brassicaceae	*Cardamine*	*Cardamine engleriana*
Brassicaceae	*Cardamine*	*Cardamine scaposa*
Brassicaceae	*Cardamine*	*Cardamine stenoloba*
Brassicaceae	*Cardamine*	*Cardamine tangutorum*
Brassicaceae	*Dontostemon*	*Dontostemon integrifolius*
Brassicaceae	*Draba*	*Draba ladyginii*
Brassicaceae	*Erysimum*	*Erysimum macilentum*
Brassicaceae	*Eutrema*	*Eutrema yunnanense*
Brassicaceae	*Lepidium*	*Lepidium cuneiforme*
Brassicaceae	*Pugionium*	*Pugionium cornutum*
Brassicaceae	*Yinshania*	*Yinshania acutangula*
Buxaceae	*Buxus*	*Buxus ichangensis*
Buxaceae	*Buxus*	*Buxus henryi*
Buxaceae	*Pachysandra*	*Pachysandra axillaris*
Buxaceae	*Sarcococca*	*Sarcococca ruscifolia*
Calycanthaceae	*Calycanthus*	*Calycanthus chinensis*
Calycanthaceae	*Chimonanthus*	*Chimonanthus nitens*
Calycanthaceae	*Chimonanthus*	*Chimonanthus praecox*
Calycanthaceae	*Chimonanthus*	*Chimonanthus salicifolius*
Campanulaceae	*Codonopsis*	*Codonopsis cardiophylla*
Campanulaceae	*Codonopsis*	*Codonopsis tsinlingensis*
Campanulaceae	*Adenophora*	*Adenophora capillaris subsp. Paniculata*
Campanulaceae	*Adenophora*	*Adenophora liliifolioides*
Campanulaceae	*Adenophora*	*Adenophora petiolata subsp. hunanensis*
Campanulaceae	*Adenophora*	*Adenophora petiolata*
Campanulaceae	*Adenophora*	*Adenophora polyantha subsp. scabricalyx*
Campanulaceae	*Adenophora*	*Adenophora potaninii*
Campanulaceae	*Adenophora*	*Adenophora potaninii subsp.*

		wawreana
Campanulaceae	*Adenophora*	*Adenophora stricta subsp. sessilifolia*
Campanulaceae	*Codonopsis*	*Codonopsis pilosula subsp. Tangshen*
Campanulaceae	*Codonopsis*	*Codonopsis viridiflora*
Campanulaceae	*Echinocodon*	*Echinocodon lobophyllus*
Cannabaceae	*Celtis*	*Celtis cerasifera*
Cannabaceae	*Celtis*	*Celtis julianae*
Cannabaceae	*Pteroceltis*	*Pteroceltis tatarinowii*
Caprifoliaceae	*Dipelta*	*Dipelta yunnanensis*
Caprifoliaceae	*Kolkwitzia*	*Kolkwitzia amabilis*
Caprifoliaceae	*Lonicera*	*Lonicera chrysantha var. koehneana*
Caprifoliaceae	*Lonicera*	*Lonicera elisae*
Caprifoliaceae	*Lonicera*	*Lonicera fargesii*
Caprifoliaceae	*Lonicera*	*Lonicera fragrantissima*
Caprifoliaceae	*Lonicera*	*Lonicera gynochlamydea*
Caprifoliaceae	*Lonicera*	*Lonicera japonica var. chinensis*
Caprifoliaceae	*Lonicera*	*Lonicera kansuensis*
Caprifoliaceae	*Lonicera*	*Lonicera ligustrina var. pileata*
Caprifoliaceae	*Lonicera*	*Lonicera ligustrina var. yunnanensis*
Caprifoliaceae	*Lonicera*	*Lonicera modesta*
Caprifoliaceae	*Lonicera*	*Lonicera nervosa*
Caprifoliaceae	*Lonicera*	*Lonicera retusa*
Caprifoliaceae	*Lonicera*	*Lonicera stephanocarpa*
Caprifoliaceae	*Lonicera*	*Lonicera tragophylla*
Caprifoliaceae	*Lonicera*	*Lonicera trichosantha*
Caprifoliaceae	*Symphoricarpos*	*Symphoricarpos sinensis*
Caprifoliaceae	*Abelia*	*Abelia macrotera*
Caprifoliaceae	*Abelia*	*Abelia uniflora*
Caprifoliaceae	*Dipelta*	*Dipelta floribunda*
Caprifoliaceae	*Patrinia*	*Patrinia scabra*
Caprifoliaceae	*Valeriana*	*Valeriana daphniflora*
Caprifoliaceae	*Valeriana*	*Valeriana minutiflora*
Caryophyllaceae	*Cerastium*	*Cerastium limprichtii*
Caryophyllaceae	*Arenaria*	*Arenaria giraldii*
Caryophyllaceae	*Arenaria*	*Arenaria taibaishanensis*
Caryophyllaceae	*Cerastium*	*Cerastium wilsonii*
Caryophyllaceae	*Gypsophila*	*Gypsophila huashanensis*
Caryophyllaceae	*Silene*	*Silene fortunei*
Caryophyllaceae	*Silene*	*Silene hupehensis*

Caryophyllaceae	*Silene*	*Silene hupehensis var. pubescens*
Caryophyllaceae	*Silene*	*Silene tatarinowii*
Caryophyllaceae	*Stellaria*	*Stellaria chinensis*
Caryophyllaceae	*Stellaria*	*Stellaria salicifolia*
Celastraceae	*Euonymus*	*Euonymus szechuanensis*
Celastraceae	*Celastrus*	*Celastrus angulatus*
Celastraceae	*Celastrus*	*Celastrus gemmatus*
Celastraceae	*Celastrus*	*Celastrus hypoleucus*
Celastraceae	*Celastrus*	*Celastrus rosthornianus var. loeseneri*
Celastraceae	*Celastrus*	*Celastrus rosthornianus*
Celastraceae	*Euonymus*	*Euonymus actinocarpus*
Celastraceae	*Euonymus*	*Euonymus giraldii*
Celastraceae	*Euonymus*	*Euonymus microcarpus*
Celastraceae	*Euonymus*	*Euonymus myrianthus*
Celastraceae	*Euonymus*	*Euonymus nanoides*
Celastraceae	*Euonymus*	*Euonymus phellomanus*
Celastraceae	*Euonymus*	*Euonymus sanguineus*
Celastraceae	*Euonymus*	*Euonymus schensianus*
Celastraceae	*Euonymus*	*Euonymus venosus*
Celastraceae	*Euonymus*	*Euonymus verrucosoides*
Celastraceae	*Euonymus*	*Euonymus wilsonii*
Celastraceae	*Parnassia*	*Parnassia brevistyla*
Celastraceae	*Parnassia*	*Parnassia oreophila*
Celastraceae	*Parnassia*	*Parnassia viridiflora*
Chloranthaceae	*Chloranthus*	*Chloranthus henryi*
Circaeasteraceae	*Kingdonia*	*Kingdonia uniflora*
Colchicaceae	*Disporum*	*Disporum longistylum*
Colchicaceae	*Disporum*	*Disporum megalanthum*
Convolvulaceae	*Tridynamia*	*Tridynamia sinensis var. delavayi*
Cornaceae	*Alangium*	*Alangium chinense subsp. Pauciflorum*
Cornaceae	*Cornus*	*Cornus bretschneideri*
Cornaceae	*Cornus*	*Cornus bretschneideri var. crispa*
Cornaceae	*Cornus*	*Cornus elliptica*
Cornaceae	*Cornus*	*Cornus hemsleyi*
Cornaceae	*Cornus*	*Cornus koehneana*
Cornaceae	*Cornus*	*Cornus kousa subsp. chinensis*
Cornaceae	*Cornus*	*Cornus multinervosa*
Cornaceae	*Cornus*	*Cornus quinquenervis*
Cornaceae	*Cornus*	*Cornus schindleri subsp.*

		poliophylla
Cornaceae	*Cornus*	*Cornus ulotricha*
Cornaceae	*Cornus*	*Cornus walteri*
Cornaceae	*Cornus*	*Cornus wilsoniana*
Crassulaceae	*Hylotelephium*	*Hylotelephium angustum var. longipedunculum*
Crassulaceae	*Sinocrassula*	*Sinocrassula indica var. viridiflora*
Crassulaceae	*Hylotelephium*	*Hylotelephium angustum*
Crassulaceae	*Kungia*	*Kungia schoenlandii var. stenostachya*
Crassulaceae	*Orostachys*	*Orostachys chanetii*
Crassulaceae	*Phedimus*	*Phedimus aizoon var. scabrus*
Crassulaceae	*Rhodiola*	*Rhodiola yunnanensis*
Crassulaceae	*Sedum*	*Sedum barbeyi*
Crassulaceae	*Sedum*	*Sedum pampaninii*
Crassulaceae	*Sedum*	*Sedum phyllanthum*
Crassulaceae	*Sedum*	*Sedum planifolium*
Crassulaceae	*Sedum*	*Sedum stellariifolium*
Cucurbitaceae	*Gynostemma*	*Gynostemma cardiospermum*
Cucurbitaceae	*Bolbostemma*	*Bolbostemma paniculatum*
Cucurbitaceae	*Gynostemma*	*Gynostemma longipes*
Cucurbitaceae	*Schizopepon*	*Schizopepon dioicus*
Cucurbitaceae	*Schizopepon*	*Schizopepon dioicus var. wilsonii*
Cucurbitaceae	*Thladiantha*	*Thladiantha henryi*
Cucurbitaceae	*Thladiantha*	*Thladiantha villosula*
Cucurbitaceae	*Trichosanthes*	*Trichosanthes rosthornii*
Cupressaceae	*Metasequoia*	*Metasequoia glyptostroboides*
Cupressaceae	*Thuja*	*Thuja sutchuenensis*
Cupressaceae	*Cupressus*	*Cupressus duclouxiana*
Cupressaceae	*Cupressus*	*Cupressus funebris*
Cupressaceae	*Juniperus*	*Juniperus formosana*
Cupressaceae	*Juniperus*	*Juniperus pingii var. wilsonii*
Cyperaceae	*Carex*	*Carex shaanxiensis*
Cyperaceae	*Blysmus*	*Blysmus sinocompressus var. nodosus*
Cyperaceae	*Carex*	*Carex agglomerata*
Cyperaceae	*Carex*	*Carex atrofuscoides*
Cyperaceae	*Carex*	*Carex breviaristata*
Cyperaceae	*Carex*	*Carex brevicuspis var. basiflora*
Cyperaceae	*Carex*	*Carex capilliculmis*
Cyperaceae	*Carex*	*Carex capilliformis*
Cyperaceae	*Carex*	*Carex chinensis*

Cyperaceae	*Carex*	*Carex chungii*
Cyperaceae	*Carex*	*Carex cranaocarpa*
Cyperaceae	*Carex*	*Carex davidii*
Cyperaceae	*Carex*	*Carex diplodon*
Cyperaceae	*Carex*	*Carex fargesii*
Cyperaceae	*Carex*	*Carex gentilis*
Cyperaceae	*Carex*	*Carex gentilis var. intermedia*
Cyperaceae	*Carex*	*Carex giraldiana*
Cyperaceae	*Carex*	*Carex grandiligulata*
Cyperaceae	*Carex*	*Carex henryi*
Cyperaceae	*Carex*	*Carex heudesii*
Cyperaceae	*Carex*	*Carex huashanica*
Cyperaceae	*Carex*	*Carex kansuensis*
Cyperaceae	*Carex*	*Carex kwangtoushanica*
Cyperaceae	*Carex*	*Carex lancifolia*
Cyperaceae	*Carex*	*Carex luctuosa*
Cyperaceae	*Carex*	*Carex meihsienica*
Cyperaceae	*Carex*	*Carex omeiensis*
Cyperaceae	*Carex*	*Carex ovatispiculata*
Cyperaceae	*Carex*	*Carex paracuraica*
Cyperaceae	*Carex*	*Carex polyschoenoides*
Cyperaceae	*Carex*	*Carex pseudodispalata*
Cyperaceae	*Carex*	*Carex rochebrunii subsp. remotispicula*
Cyperaceae	*Carex*	*Carex rochebrunii subsp. reptans*
Cyperaceae	*Carex*	*Carex rubrobrunnea var. taliensis*
Cyperaceae	*Carex*	*Carex scabrirostris*
Cyperaceae	*Carex*	*Carex schneideri*
Cyperaceae	*Carex*	*Carex setosa var. mianxianica*
Cyperaceae	*Carex*	*Carex shaanxiensis*
Cyperaceae	*Carex*	*Carex stipitinux*
Cyperaceae	*Carex*	*Carex thibetica*
Cyperaceae	*Fimbristylis*	*Fimbristylis henryi*
Cystopteridaceae	*Cystopteris*	*Cystopteris fragilis*
Cystopteridaceae	*Cystopteris*	*Cystopteris pellucida*
Daphniphyllaceae	*Daphniphyllum*	*Daphniphyllum angustifolium*
Dennstaedtiaceae	*Dennstaedtia*	*Dennstaedtia wilfordii*
Dennstaedtiaceae	*Microlepia*	*Microlepia marginata*
Dennstaedtiaceae	*Microlepia*	*Microlepia pseudostrigosa*
Dioscoreaceae	*Dioscorea*	*Dioscorea nipponica subsp. rosthornii*
Dioscoreaceae	*Dioscorea*	*Dioscorea zingiberensis*

Dryopteridaceae	*Cyrtomium*	*Cyrtomium caryotideum*
Dryopteridaceae	*Cyrtomium*	*Cyrtomium fortunei*
Dryopteridaceae	*Cyrtomium*	*Cyrtomium macrophyllum*
Dryopteridaceae	*Cyrtomium*	*Cyrtomium tsinglingense*
Dryopteridaceae	*Cyrtomium*	*Cyrtomium yamamotoi*
Dryopteridaceae	*Dryopteris*	*Dryopteris atrata*
Dryopteridaceae	*Dryopteris*	*Dryopteris immixta*
Dryopteridaceae	*Dryopteris*	*Dryopteris peninsulae*
Dryopteridaceae	*Dryopteris*	*Dryopteris pulcherrima*
Dryopteridaceae	*Dryopteris*	*Dryopteris rosthornii*
Dryopteridaceae	*Dryopteris*	*Dryopteris sericea*
Dryopteridaceae	*Polystichum*	*Polystichum baoxingense*
Dryopteridaceae	*Polystichum*	*Polystichum erosum*
Dryopteridaceae	*Polystichum*	*Polystichum kangdingense*
Dryopteridaceae	*Polystichum*	*Polystichum longiaristatum*
Dryopteridaceae	*Polystichum*	*Polystichum melanostipes*
Dryopteridaceae	*Polystichum*	*Polystichum mollissimum*
Dryopteridaceae	*Polystichum*	*Polystichum muscicola*
Dryopteridaceae	*Polystichum*	*Polystichum shensiense*
Dryopteridaceae	*Polystichum*	*Polystichum sinense*
Dryopteridaceae	*Polystichum*	*Polystichum submite*
Dryopteridaceae	*Polystichum*	*Polystichum tsus-simense*
Ebenaceae	*Diospyros*	*Diospyros armata*
Ebenaceae	*Diospyros*	*Diospyros cathayensis*
Ebenaceae	*Diospyros*	*Diospyros kaki var. silvestris*
Ebenaceae	*Diospyros*	*Diospyros lotus var. mollissima*
Ebenaceae	*Diospyros*	*Diospyros rhombifolia*
Elaeagnaceae	*Elaeagnus*	*Elaeagnus mollis*
Elaeagnaceae	*Elaeagnus*	*Elaeagnus angustata*
Elaeagnaceae	*Elaeagnus*	*Elaeagnus bockii*
Elaeagnaceae	*Elaeagnus*	*Elaeagnus difficilis*
Elaeagnaceae	*Elaeagnus*	*Elaeagnus henryi*
Elaeagnaceae	*Elaeagnus*	*Elaeagnus lanceolata*
Elaeagnaceae	*Elaeagnus*	*Elaeagnus magna*
Elaeagnaceae	*Elaeagnus*	*Elaeagnus viridis*
Elaeagnaceae	*Hippophae*	*Hippophae rhamnoides subsp. Sinensis*
Elaeocarpaceae	*Sloanea*	*Sloanea hemsleyana*
Ericaceae	*Pyrola*	*Pyrola rugosa*
Ericaceae	*Rhododendron*	*Rhododendron detersile*
Ericaceae	*Rhododendron*	*Rhododendron purdomii*
Ericaceae	*Enkianthus*	*Enkianthus chinensis*
Ericaceae	*Lyonia*	*Lyonia ovalifolia var. hebecarpa*

Ericaceae	*Pyrola*	*Pyrola atropurpurea*
Ericaceae	*Pyrola*	*Pyrola calliantha*
Ericaceae	*Rhododendron*	*Rhododendron augustinii*
Ericaceae	*Rhododendron*	*Rhododendron auriculatum*
Ericaceae	*Rhododendron*	*Rhododendron calophytum*
Ericaceae	*Rhododendron*	*Rhododendron capitatum*
Ericaceae	*Rhododendron*	*Rhododendron clementinae subsp. aureodorsale*
Ericaceae	*Rhododendron*	*Rhododendron concinnum*
Ericaceae	*Rhododendron*	*Rhododendron declivatum*
Ericaceae	*Rhododendron*	*Rhododendron discolor*
Ericaceae	*Rhododendron*	*Rhododendron fortunei*
Ericaceae	*Rhododendron*	*Rhododendron hunnewellianum*
Ericaceae	*Rhododendron*	*Rhododendron hypoglaucum*
Ericaceae	*Rhododendron*	*Rhododendron maculiferum*
Ericaceae	*Rhododendron*	*Rhododendron mariesii*
Ericaceae	*Rhododendron*	*Rhododendron oreodoxa var. fargesii*
Ericaceae	*Rhododendron*	*Rhododendron oreodoxa*
Ericaceae	*Rhododendron*	*Rhododendron oreodoxa var. shensiense*
Ericaceae	*Rhododendron*	*Rhododendron pachytrichum*
Ericaceae	*Rhododendron*	*Rhododendron polylepis*
Ericaceae	*Rhododendron*	*Rhododendron praevernum*
Ericaceae	*Rhododendron*	*Rhododendron przewalskii*
Ericaceae	*Rhododendron*	*Rhododendron roxieanum*
Ericaceae	*Rhododendron*	*Rhododendron rufum*
Ericaceae	*Rhododendron*	*Rhododendron stamineum*
Ericaceae	*Rhododendron*	*Rhododendron sutchuenense*
Ericaceae	*Rhododendron*	*Rhododendron taibaiense*
Ericaceae	*Vaccinium*	*Vaccinium henryi*
Ericaceae	*Vaccinium*	*Vaccinium japonicum var. sinicum*
Ericaceae	*Vaccinium*	*Vaccinium mandarinorum*
Eucommiaceae	*Eucommia*	*Eucommia ulmoides*
Euphorbiaceae	*Alchornea*	*Alchornea davidii*
Euphorbiaceae	*Discocleidion*	*Discocleidion rufescens*
Euphorbiaceae	*Euphorbia*	*Euphorbia kansuensis*
Euphorbiaceae	*Euphorbia*	*Euphorbia kansui*
Euphorbiaceae	*Mallotus*	*Mallotus tenuifolius var. paxii*
Euphorbiaceae	*Speranskia*	*Speranskia cantonensis*
Euphorbiaceae	*Speranskia*	*Speranskia tuberculata*
Fabaceae	*Astragalus*	*Astragalus henryi*

Fabaceae	*Astragalus*	*Astragalus taiyuanensis*
Fabaceae	*Caragana*	*Caragana purdomii*
Fabaceae	*Caragana*	*Caragana stipitata*
Fabaceae	*Hedysarum*	*Hedysarum taipeicum*
Fabaceae	*Ormosia*	*Ormosia hosiei*
Fabaceae	*Astragalus*	*Astragalus chrysopterus*
Fabaceae	*Astragalus*	*Astragalus havianus*
Fabaceae	*Astragalus*	*Astragalus huiningensis*
Fabaceae	*Astragalus*	*Astragalus kifonsanicus*
Fabaceae	*Astragalus*	*Astragalus limprichtii*
Fabaceae	*Astragalus*	*Astragalus monadelphus*
Fabaceae	*Astragalus*	*Astragalus satoi*
Fabaceae	*Astragalus*	*Astragalus taipaishanensis*
Fabaceae	*Astragalus*	*Astragalus xitaibaicus*
Fabaceae	*Campylotropis*	*Campylotropis macrocarpa var. hupehensis*
Fabaceae	*Caragana*	*Caragana boisii*
Fabaceae	*Caragana*	*Caragana leveillei*
Fabaceae	*Caragana*	*Caragana opulens*
Fabaceae	*Caragana*	*Caragana roborovskyi*
Fabaceae	*Caragana*	*Caragana rosea*
Fabaceae	*Caragana*	*Caragana shensiensis*
Fabaceae	*Cladrastis*	*Cladrastis delavayi*
Fabaceae	*Cladrastis*	*Cladrastis wilsonii*
Fabaceae	*Corethrodendron*	*Corethrodendron multijugum*
Fabaceae	*Gleditsia*	*Gleditsia microphylla*
Fabaceae	*Gleditsia*	*Gleditsia sinensis*
Fabaceae	*Hedysarum*	*Hedysarum dentatoalatum*
Fabaceae	*Hedysarum*	*Hedysarum petrovii*
Fabaceae	*Indigofera*	*Indigofera amblyantha*
Fabaceae	*Indigofera*	*Indigofera carlesii*
Fabaceae	*Indigofera*	*Indigofera fortunei*
Fabaceae	*Indigofera*	*Indigofera silvestrii*
Fabaceae	*Lathyrus*	*Lathyrus dielsianus*
Fabaceae	*Lespedeza*	*Lespedeza potaninii*
Fabaceae	*Maackia*	*Maackia hupehensis*
Fabaceae	*Maackia*	*Maackia hwashanensis*
Fabaceae	*Maackia*	*Maackia tenuifolia*
Fabaceae	*Medicago*	*Medicago archiducis-nicolaii*
Fabaceae	*Oxytropis*	*Oxytropis chinglingensis*
Fabaceae	*Oxytropis*	*Oxytropis giraldii*
Fabaceae	*Oxytropis*	*Oxytropis gueldenstaedtioides*
Fabaceae	*Oxytropis*	*Oxytropis latibracteata*
Fabaceae	*Oxytropis*	*Oxytropis melanocalyx*

Fabaceae	*Oxytropis*	*Oxytropis ramosissima*
Fabaceae	*Oxytropis*	*Oxytropis sitaipaiensis*
Fabaceae	*Oxytropis*	*Oxytropis taochensis*
Fabaceae	*Oxytropis*	*Oxytropis trichophora*
Fabaceae	*Phyllolobium*	*Phyllolobium chinense*
Fabaceae	*Piptanthus*	*Piptanthus nepalensis*
Fabaceae	*Rhynchosia*	*Rhynchosia dielsii*
Fabaceae	*Sophora*	*Sophora davidii*
Fabaceae	*Thermopsis*	*Thermopsis przewalskii*
Fabaceae	*Vicia*	*Vicia chinensis*
Fabaceae	*Vicia*	*Vicia kioshanica*
Fabaceae	*Vicia*	*Vicia kioshanica*
Fabaceae	*Vicia*	*Vicia perelegans*
Fabaceae	*Vicia*	*Vicia taipaica*
Fabaceae	*Wisteria*	*Wisteria villosa*
Fagaceae	*Cyclobalanopsis*	*Cyclobalanopsis gracilis*
Fagaceae	*Cyclobalanopsis*	*Cyclobalanopsis multinervis*
Fagaceae	*Fagus*	*Fagus engleriana*
Fagaceae	*Fagus*	*Fagus hayatae*
Fagaceae	*Lithocarpus*	*Lithocarpus cleistocarpus*
Fagaceae	*Lithocarpus*	*Lithocarpus henryi*
Fagaceae	*Quercus*	*Quercus acrodonta*
Fagaceae	*Quercus*	*Quercus baronii*
Fagaceae	*Quercus*	*Quercus cocciferoides*
Fagaceae	*Quercus*	*Quercus dolicholepis*
Fagaceae	*Quercus*	*Quercus engleriana*
Fagaceae	*Quercus*	*Quercus fabri*
Fagaceae	*Quercus*	*Quercus oxyphylla*
Garryaceae	*Aucuba*	*Aucuba albopunctifolia var. angustula*
Garryaceae	*Aucuba*	*Aucuba himalaica var. dolichophylla*
Garryaceae	*Aucuba*	*Aucuba himalaica var. pilosissima*
Garryaceae	*Aucuba*	*Aucuba obcordata*
Gentianaceae	*Gentiana*	*Gentiana apiata*
Gentianaceae	*Gentiana*	*Gentiana arethusae var. delicatula*
Gentianaceae	*Gentiana*	*Gentiana hexaphylla*
Gentianaceae	*Gentiana*	*Gentiana licentii*
Gentianaceae	*Gentiana*	*Gentiana macrauchena*
Gentianaceae	*Gentiana*	*Gentiana officinalis*
Gentianaceae	*Gentiana*	*Gentiana oligophylla*
Gentianaceae	*Gentiana*	*Gentiana pudica*

Gentianaceae	*Gentiana*	*Gentiana rhodantha*
Gentianaceae	*Gentiana*	*Gentiana shaanxiensis*
Gentianaceae	*Gentiana*	*Gentiana spathulifolia*
Gentianaceae	*Gentiana*	*Gentiana sutchuenensis*
Gentianaceae	*Gentiana*	*Gentiana vandellioides*
Gentianaceae	*Gentianopsis*	*Gentianopsis paludosa var. ovatodeltoidea*
Gentianaceae	*Halenia*	*Halenia elliptica var. grandiflora*
Gentianaceae	*Lomatogonium*	*Lomatogonium bellum*
Gentianaceae	*Lomatogonium*	*Lomatogonium perenne*
Gentianaceae	*Swertia*	*Swertia bifolia*
Gentianaceae	*Swertia*	*Swertia dichotoma var. punctata*
Gentianaceae	*Swertia*	*Swertia kouitchensis*
Gentianaceae	*Tripterospermum*	*Tripterospermum coeruleum*
Gentianaceae	*Tripterospermum*	*Tripterospermum cordatum*
Gentianaceae	*Tripterospermum*	*Tripterospermum discoideum*
Gentianaceae	*Tripterospermum*	*Tripterospermum filicaule*
Geraniaceae	*Geranium*	*Geranium napuligerum*
Geraniaceae	*Geranium*	*Geranium pylzowianum*
Geraniaceae	*Geranium*	*Geranium rosthornii*
Geraniaceae	*Geranium*	*Geranium shensianum*
Gesneriaceae	*Petrocosmea*	*Petrocosmea qinlingensis*
Gesneriaceae	*Ancylostemon*	*Ancylostemon saxatilis*
Gesneriaceae	*Boea*	*Boea clarkeana*
Gesneriaceae	*Boea*	*Boea hygrometrica*
Gesneriaceae	*Isometrum*	*Isometrum farreri*
Gesneriaceae	*Isometrum*	*Isometrum giraldii*
Gesneriaceae	*Isometrum*	*Isometrum primuliflorum*
Ginkgoaceae	*Ginkgo*	*Ginkgo biloba*
Grossulariaceae	*Ribes*	*Ribes franchetii*
Grossulariaceae	*Ribes*	*Ribes giraldii*
Grossulariaceae	*Ribes*	*Ribes glabrifolium*
Grossulariaceae	*Ribes*	*Ribes himalense var. glandulosum*
Grossulariaceae	*Ribes*	*Ribes himalense var. trichophyllum*
Grossulariaceae	*Ribes*	*Ribes himalense var. verruculosum*
Grossulariaceae	*Ribes*	*Ribes longiracemosum var. gracillimum*
Grossulariaceae	*Ribes*	*Ribes mandshuricum var. subglabrum*

Grossulariaceae	*Ribes*	*Ribes moupinense*
Grossulariaceae	*Ribes*	*Ribes moupinense var. tripartitum*
Grossulariaceae	*Ribes*	*Ribes rubrisepalum*
Grossulariaceae	*Ribes*	*Ribes stenocarpum*
Grossulariaceae	*Ribes*	*Ribes vilmorinii*
Hamamelidaceae	*Sinowilsonia*	*Sinowilsonia henryi*
Hamamelidaceae	*Corylopsis*	*Corylopsis microcarpa*
Hamamelidaceae	*Distylium*	*Distylium chinense*
Hamamelidaceae	*Fortunearia*	*Fortunearia sinensis*
Hamamelidaceae	*Parrotia*	*Parrotia subaequalis*
Hamamelidaceae	*Corylopsis*	*Corylopsis sinensis*
Hamamelidaceae	*Distylium*	*Distylium buxifolium*
Hamamelidaceae	*Loropetalum*	*Loropetalum chinense var. rubrum*
Hamamelidaceae	*Sycopsis*	*Sycopsis sinensis*
Helwingiaceae	*Helwingia*	*Helwingia chinensis var. crenata*
Helwingiaceae	*Helwingia*	*Helwingia japonica var. hypoleuca*
Helwingiaceae	*Helwingia*	*Helwingia japonica var. papillosa*
Helwingiaceae	*Helwingia*	*Helwingia omeiensis*
Hydrangeaceae	*Deinanthe*	*Deinanthe caerulea*
Hydrangeaceae	*Decumaria*	*Decumaria sinensis*
Hydrangeaceae	*Deinanthe*	*Deinanthe caerulea*
Hydrangeaceae	*Deutzia*	*Deutzia albida*
Hydrangeaceae	*Deutzia*	*Deutzia baroniana*
Hydrangeaceae	*Deutzia*	*Deutzia discolor*
Hydrangeaceae	*Deutzia*	*Deutzia glabrata var. sessilifolia*
Hydrangeaceae	*Deutzia*	*Deutzia grandiflora*
Hydrangeaceae	*Deutzia*	*Deutzia hypoglauca*
Hydrangeaceae	*Deutzia*	*Deutzia longifolia*
Hydrangeaceae	*Deutzia*	*Deutzia ningpoensis*
Hydrangeaceae	*Deutzia*	*Deutzia parviflora var. micrantha*
Hydrangeaceae	*Deutzia*	*Deutzia rubens*
Hydrangeaceae	*Deutzia*	*Deutzia taibaiensis*
Hydrangeaceae	*Hydrangea*	*Hydrangea bretschneideri*
Hydrangeaceae	*Hydrangea*	*Hydrangea coacta*
Hydrangeaceae	*Hydrangea*	*Hydrangea hypoglauca*
Hydrangeaceae	*Hydrangea*	*Hydrangea longipes var. fulvescens*

Hydrangeaceae	*Hydrangea*	*Hydrangea longipes var. lanceolata*
Hydrangeaceae	*Hydrangea*	*Hydrangea longipes*
Hydrangeaceae	*Hydrangea*	*Hydrangea strigosa*
Hydrangeaceae	*Hydrangea*	*Hydrangea xanthoneura*
Hydrangeaceae	*Philadelphus*	*Philadelphus dasycalyx*
Hydrangeaceae	*Philadelphus*	*Philadelphus incanus var. baileyi*
Hydrangeaceae	*Philadelphus*	*Philadelphus incanus var. baileyi*
Hydrangeaceae	*Philadelphus*	*Philadelphus kansuensis*
Hydrangeaceae	*Philadelphus*	*Philadelphus laxiflorus*
Hydrangeaceae	*Philadelphus*	*Philadelphus sericanthus*
Hydrangeaceae	*Schizophragma*	*Schizophragma hypoglaucum*
Hydrangeaceae	*Schizophragma*	*Schizophragma integrifolium*
Hymenophyllaceae	*Hymenophyllum*	*Hymenophyllum corrugatum*
Hypericaceae	*Hypericum*	*Hypericum beanii*
Hypericaceae	*Hypericum*	*Hypericum elatoides*
Hypericaceae	*Hypericum*	*Hypericum faberi*
Hypericaceae	*Hypericum*	*Hypericum longistylum*
Hypericaceae	*Hypericum*	*Hypericum przewalskii*
Hypericaceae	*Hypericum*	*Hypericum subcordatum*
Hypodematiaceae	*Hypodematium*	*Hypodematium gracile*
Icacinaceae	*Hosiea*	*Hosiea sinensis*
Icacinaceae	*Nothapodytes*	*Nothapodytes pittosporoides*
Iridaceae	*Iris*	*Iris henryi*
Iridaceae	*Iris*	*Iris speculatrix*
Iteaceae	*Itea*	*Itea ilicifolia*
Juglandaceae	*Cyclocarya*	*Cyclocarya paliurus*
Juglandaceae	*Pterocarya*	*Pterocarya hupehensis*
Juglandaceae	*Pterocarya*	*Pterocarya macroptera var. insignis*
Juglandaceae	*Pterocarya*	*Pterocarya macroptera*
Juncaceae	*Juncus*	*Juncus modicus*
Juncaceae	*Juncus*	*Juncus potaninii*
Juncaceae	*Juncus*	*Juncus tanguticus*
Lamiaceae	*Ajuga*	*Ajuga ciliata var. chanetii*
Lamiaceae	*Ajuga*	*Ajuga ciliata var. glabrescens*
Lamiaceae	*Ajuga*	*Ajuga linearifolia*
Lamiaceae	*Chelonopsis*	*Chelonopsis giraldii*
Lamiaceae	*Clinopodium*	*Clinopodium polycephalum*
Lamiaceae	*Elsholtzia*	*Elsholtzia cyprianii*
Lamiaceae	*Elsholtzia*	*Elsholtzia feddei*
Lamiaceae	*Elsholtzia*	*Elsholtzia stauntonii*

Lamiaceae	*Glechoma*	*Glechoma biondiana*
Lamiaceae	*Glechoma*	*Glechoma biondiana var. glabrescens*
Lamiaceae	*Heterolamium*	*Heterolamium debile*
Lamiaceae	*Isodon*	*Isodon excisoides*
Lamiaceae	*Isodon*	*Isodon henryi*
Lamiaceae	*Isodon*	*Isodon nervosus*
Lamiaceae	*Isodon*	*Isodon parvifolius*
Lamiaceae	*Isodon*	*Isodon racemosus*
Lamiaceae	*Isodon*	*Isodon rubescens*
Lamiaceae	*Kinostemon*	*Kinostemon ornatum*
Lamiaceae	*Leonurus*	*Leonurus chaituroides*
Lamiaceae	*Leonurus*	*Leonurus pseudomacranthus*
Lamiaceae	*Loxocalyx*	*Loxocalyx urticifolius*
Lamiaceae	*Loxocalyx*	*Loxocalyx urticifolius var. decemnervius*
Lamiaceae	*Meehania*	*Meehania faberi*
Lamiaceae	*Meehania*	*Meehania henryi*
Lamiaceae	*Nepeta*	*Nepeta fordii*
Lamiaceae	*Nepeta*	*Nepeta prattii*
Lamiaceae	*Phlomis*	*Phlomis megalantha*
Lamiaceae	*Phlomis*	*Phlomis mongolica*
Lamiaceae	*Phlomis*	*Phlomis szechuanensis*
Lamiaceae	*Phlomis*	*Phlomis umbrosa var. australis*
Lamiaceae	*Phlomis*	*Phlomis umbrosa var. latibracteata*
Lamiaceae	*Phlomis*	*Phlomis umbrosa var. stenocalyx*
Lamiaceae	*Phlomis*	*Phlomis umbrosa*
Lamiaceae	*Rostrinucula*	*Rostrinucula dependens*
Lamiaceae	*Salvia*	*Salvia cavaleriei var. erythrophylla*
Lamiaceae	*Salvia*	*Salvia cavaleriei var. simplicifolia*
Lamiaceae	*Salvia*	*Salvia chinensis*
Lamiaceae	*Salvia*	*Salvia hupehensis*
Lamiaceae	*Salvia*	*Salvia maximowicziana*
Lamiaceae	*Salvia*	*Salvia miltiorrhiza var. charbonnelii*
Lamiaceae	*Salvia*	*Salvia piasezkii*
Lamiaceae	*Salvia*	*Salvia tricuspis*
Lamiaceae	*Salvia*	*Salvia umbratica*
Lamiaceae	*Scutellaria*	*Scutellaria amoena*
Lamiaceae	*Scutellaria*	*Scutellaria caryopteroides*

Lamiaceae	*Scutellaria*	*Scutellaria franchetiana*
Lamiaceae	*Scutellaria*	*Scutellaria honanensis*
Lamiaceae	*Scutellaria*	*Scutellaria rehderiana*
Lamiaceae	*Scutellaria*	*Scutellaria scordifolia var. ammophila*
Lamiaceae	*Scutellaria*	*Scutellaria scordifolia var. puberula*
Lamiaceae	*Scutellaria*	*Scutellaria scordifolia var. villosissima*
Lamiaceae	*Stachys*	*Stachys arrecta*
Lamiaceae	*Stachys*	*Stachys pseudophlomis*
Lamiaceae	*Stachys*	*Stachys sieboldii var. malacotricha*
Lamiaceae	*Teucrium*	*Teucrium viscidum var. leiocalyx*
Lamiaceae	*Teucrium*	*Teucrium viscidum var. nepetoides*
Lamiaceae	*Thymus*	*Thymus mongolicus*
Lamiaceae	*Callicarpa*	*Callicarpa giraldii*
Lamiaceae	*Callicarpa*	*Callicarpa gracilipes*
Lamiaceae	*Caryopteris*	*Caryopteris tangutica*
Lamiaceae	*Caryopteris*	*Caryopteris terniflora*
Lamiaceae	*Premna*	*Premna puberula*
Lardizabalaceae	*Akebia*	*Akebia trifoliata subsp. australis*
Lardizabalaceae	*Akebia*	*Archakebia apetala*
Lardizabalaceae	*Holboellia*	*Holboellia coriacea*
Lardizabalaceae	*Sinofranchetia*	*Sinofranchetia chinensis*
Lardizabalaceae	*Stauntonia*	*Stauntonia duclouxii*
Lauraceae	*Actinodaphne*	*Actinodaphne obscurinervia*
Lauraceae	*Machilus*	*Machilus nanmu*
Lauraceae	*Phoebe*	*Phoebe bournei*
Lauraceae	*Phoebe*	*Phoebe zhennan*
Lauraceae	*Actinodaphne*	*Actinodaphne cupularis*
Lauraceae	*Cinnamomum*	*Cinnamomum bodinieri*
Lauraceae	*Cinnamomum*	*Cinnamomum longepaniculatum*
Lauraceae	*Cinnamomum*	*Cinnamomum septentrionale*
Lauraceae	*Cinnamomum*	*Cinnamomum wilsonii*
Lauraceae	*Lindera*	*Lindera floribunda*
Lauraceae	*Lindera*	*Lindera fragrans*
Lauraceae	*Lindera*	*Lindera limprichtii*
Lauraceae	*Lindera*	*Lindera megaphylla*
Lauraceae	*Lindera*	*Lindera pulcherrima var.*

		attenuata
Lauraceae	*Lindera*	*Lindera pulcherrima var. hemsleyana*
Lauraceae	*Lindera*	*Lindera tienchuanensis*
Lauraceae	*Litsea*	*Litsea hupehana*
Lauraceae	*Litsea*	*Litsea moupinensis var. szechuanica*
Lauraceae	*Litsea*	*Litsea pungens*
Lauraceae	*Litsea*	*Litsea rubescens*
Lauraceae	*Litsea*	*Litsea tsinlingensis*
Lauraceae	*Litsea*	*Litsea veitchiana*
Lauraceae	*Machilus*	*Machilus faberi*
Lauraceae	*Machilus*	*Machilus montana*
Lauraceae	*Neolitsea*	*Neolitsea confertifolia*
Lauraceae	*Neolitsea*	*Neolitsea wushanica*
Lauraceae	*Phoebe*	*Phoebe hui*
Lauraceae	*Phoebe*	*Phoebe hunanensis*
Lauraceae	*Phoebe*	*Phoebe neurantha*
Lauraceae	*Phoebe*	*Phoebe neuranthoides*
Liliaceae	*Fritillaria*	*Fritillaria taipaiensis*
Liliaceae	*Cardiocrinum*	*Cardiocrinum cathayanum*
Liliaceae	*Fritillaria*	*Fritillaria taipaiensis*
Liliaceae	*Lilium*	*Lilium brownii*
Liliaceae	*Lilium*	*Lilium brownii var. viridulum*
Liliaceae	*Lilium*	*Lilium fargesii*
Liliaceae	*Lilium*	*Lilium leucanthum*
Liliaceae	*Lilium*	*Lilium papilliferum*
Liliaceae	*Streptopus*	*Streptopus obtusatus*
Loganiaceae	*Gardneria*	*Gardneria lanceolata*
Loranthaceae	*Taxillus*	*Taxillus sutchuenensis*
Lycopodiaceae	*Huperzia*	*Huperzia chinensis*
Lycopodiaceae	*Lycopodium*	*Lycopodium obscurum f. obscurum/Lycopodium obscurum*
Lycopodiaceae	*Lycopodium*	*Lycopodium zonatum*
Lythraceae	*Lagerstroemia*	*Lagerstroemia excelsa*
Magnoliaceae	*Michelia*	*Michelia wilsonii*
Magnoliaceae	*Yulania*	*Yulania amoena*
Magnoliaceae	*Yulania*	*Yulania dawsoniana*
Magnoliaceae	*Yulania*	*Yulania liliiflora*
Magnoliaceae	*Yulania*	*Yulania sargentiana*
Magnoliaceae	*Yulania*	*Yulania viridula*
Magnoliaceae	*Yulania*	*Yulania zenii*
Magnoliaceae	*Houpoea*	*Houpoea officinalis*

Magnoliaceae	*Michelia*	*Michelia cavaleriei var. platypetala*
Magnoliaceae	*Michelia*	*Michelia maudiae*
Magnoliaceae	*Yulania*	*Yulania cylindrica*
Magnoliaceae	*Yulania*	*Yulania denudata*
Magnoliaceae	*Yulania*	*Yulania sprengerii*
Malvaceae	*Hibiscus*	*Hibiscus leviseminus*
Malvaceae	*Grewia*	*Grewia biloba var. parviflora*
Malvaceae	*Tilia*	*Tilia chinensis*
Malvaceae	*Tilia*	*Tilia chinensis var. intonsa*
Malvaceae	*Tilia*	*Tilia chinensis var. investita*
Malvaceae	*Tilia*	*Tilia henryana*
Malvaceae	*Tilia*	*Tilia henryana var. subglabra*
Malvaceae	*Tilia*	*Tilia oliveri*
Malvaceae	*Tilia*	*Tilia paucicostata var. dictyoneura*
Malvaceae	*Tilia*	*Tilia paucicostata var. dictyoneura*
Malvaceae	*Tilia*	*Tilia paucicostata var. yunnanensis*
Malvaceae	*Tilia*	*Tilia tuan*
Mazaceae	*Mazus*	*Mazus spicatus*
Melanthiaceae	*Paris*	*Paris fargesii var. petiolata*
Melanthiaceae	*Paris*	*Paris bashanensis*
Melanthiaceae	*Paris*	*Paris fargesii var. petiolata*
Melanthiaceae	*Paris*	*Paris polyphylla var. latifolia*
Melanthiaceae	*Veratrum*	*Veratrum grandiflorum*
Melanthiaceae	*Ypsilandra*	*Ypsilandra thibetica*
Menispermaceae	*Cyclea*	*Cyclea racemosa*
Menispermaceae	*Stephania*	*Stephania sinica*
Moraceae	*Ficus*	*Ficus sarmentosa var. henryi*
Nartheciaceae	*Aletris*	*Aletris glandulifera*
Nartheciaceae	*Aletris*	*Aletris stenoloba*
Nitrariaceae	*Nitraria*	*Nitraria tangutorum*
Nitrariaceae	*Peganum*	*Peganum multisectum*
Nyctaginaceae	*Oxybaphus*	*Oxybaphus himalaicus var. chinensis*
Nyssaceae	*Camptotheca*	*Camptotheca acuminata*
Nyssaceae	*Davidia*	*Davidia involucrata*
Oleaceae	*Fraxinus*	*Fraxinus baroniana*
Oleaceae	*Osmanthus*	*Osmanthus venosus*
Oleaceae	*Forsythia*	*Forsythia giraldiana*
Oleaceae	*Forsythia*	*Forsythia mira*
Oleaceae	*Forsythia*	*Forsythia suspensa*

Oleaceae	*Fraxinus*	*Fraxinus depauperata*
Oleaceae	*Fraxinus*	*Fraxinus odontocalyx*
Oleaceae	*Ligustrum*	*Ligustrum expansum*
Oleaceae	*Ligustrum*	*Ligustrum henryi*
Oleaceae	*Ligustrum*	*Ligustrum leucanthum*
Oleaceae	*Ligustrum*	*Ligustrum lucidum*
Oleaceae	*Ligustrum*	*Ligustrum pedunculare*
Oleaceae	*Ligustrum*	*Ligustrum quihoui*
Oleaceae	*Ligustrum*	*Ligustrum sinense var. myrianthum*
Oleaceae	*Ligustrum*	*Ligustrum strongylophyllum*
Oleaceae	*Osmanthus*	*Osmanthus armatus*
Oleaceae	*Osmanthus*	*Osmanthus fragrans*
Oleaceae	*Syringa*	*Syringa komarowii subsp. Reflexa*
Oleaceae	*Syringa*	*Syringa komarowii*
Oleaceae	*Syringa*	*Syringa pinnatifolia*
Oleaceae	*Syringa*	*Syringa sweginzowii*
Oleaceae	*Syringa*	*Syringa tomentella*
Oleaceae	*Syringa*	*Syringa villosa*
Oleaceae	*Syringa*	*Syringa yunnanensis*
Onagraceae	*Circaea*	*Circaea glabrescens*
Onagraceae	*Epilobium*	*Epilobium subcoriaceum*
Orchidaceae	*Bulbophyllum*	*Bulbophyllum chondriophorum*
Orchidaceae	*Bulbophyllum*	*Bulbophyllum henanense*
Orchidaceae	*Calanthe*	*Calanthe emeishanica*
Orchidaceae	*Changnienia*	*Changnienia amoena*
Orchidaceae	*Cypripedium*	*Cypripedium fasciolatum*
Orchidaceae	*Cypripedium*	*Cypripedium franchetii*
Orchidaceae	*Cypripedium*	*Cypripedium taibaiense*
Orchidaceae	*Dendrobium*	*Dendrobium flexicaule*
Orchidaceae	*Galearis*	*Galearis tschiliensis*
Orchidaceae	*Goodyera*	*Goodyera wolongensis*
Orchidaceae	*Habenaria*	*Habenaria fargesii*
Orchidaceae	*Myrmechis*	*Myrmechis chinensis*
Orchidaceae	*Neofinetia*	*Neofinetia richardsiana*
Orchidaceae	*Neottia*	*Neottia oblata*
Orchidaceae	*Neottia*	*Neottia puberula var. maculata*
Orchidaceae	*Nothodoritis*	*Nothodoritis zhejiangensis*
Orchidaceae	*Oberonia*	*Oberonia sinica*
Orchidaceae	*Tipularia*	*Tipularia szechuanica*
Orchidaceae	*Amitostigma*	*Amitostigma monanthum*

Orchidaceae	*Androcorys*	*Androcorys ophioglossoides*
Orchidaceae	*Bulbophyllum*	*Bulbophyllum chondriophorum*
Orchidaceae	*Bulbophyllum*	*Bulbophyllum henanense*
Orchidaceae	*Calanthe*	*Calanthe emeishanica*
Orchidaceae	*Changnienia*	*Changnienia amoena*
Orchidaceae	*Cymbidium*	*Cymbidium serratum*
Orchidaceae	*Cypripedium*	*Cypripedium fasciolatum*
Orchidaceae	*Cypripedium*	*Cypripedium franchetii*
Orchidaceae	*Cypripedium*	*Cypripedium henryi*
Orchidaceae	*Cypripedium*	*Cypripedium taibaiense*
Orchidaceae	*Dendrobium*	*Dendrobium flexicaule*
Orchidaceae	*Galearis*	*Galearis tschiliensis*
Orchidaceae	*Gastrochilus*	*Gastrochilus formosanus*
Orchidaceae	*Goodyera*	*Goodyera wolongensis*
Orchidaceae	*Habenaria*	*Habenaria fargesii*
Orchidaceae	*Habenaria*	*Habenaria glaucifolia*
Orchidaceae	*Habenaria*	*Habenaria szechuanica*
Orchidaceae	*Hemipilia*	*Hemipilia crassicalcarata*
Orchidaceae	*Hemipilia*	*Hemipilia flabellata*
Orchidaceae	*Hemipilia*	*Hemipilia henryi*
Orchidaceae	*Herminium*	*Herminium ophioglossoides*
Orchidaceae	*Ischnogyne*	*Ischnogyne mandarinorum*
Orchidaceae	*Liparis*	*Liparis angustioblonga*
Orchidaceae	*Liparis*	*Liparis fargesii*
Orchidaceae	*Liparis*	*Liparis pauliana*
Orchidaceae	*Myrmechis*	*Myrmechis chinensis*
Orchidaceae	*Neofinetia*	*Neofinetia richardsiana*
Orchidaceae	*Neottia*	*Neottia chenii*
Orchidaceae	*Neottia*	*Neottia oblata*
Orchidaceae	*Neottia*	*Neottia puberula var. maculata*
Orchidaceae	*Neottia*	*Neottia taibaishanensis*
Orchidaceae	*Neottia*	*Neottia wardii*
Orchidaceae	*Nothodoritis*	*Nothodoritis zhejiangensis*
Orchidaceae	*Oberonia*	*Oberonia sinica*
Orchidaceae	*Oreorchis*	*Oreorchis fargesii*
Orchidaceae	*Oreorchis*	*Oreorchis nana*
Orchidaceae	*Pleione*	*Pleione bulbocodioides*
Orchidaceae	*Ponerorchis*	*Ponerorchis limprichtii*
Orchidaceae	*Tipularia*	*Tipularia szechuanica*
Orobanchaceae	*Pedicularis*	*Pedicularis bicolor*
Orobanchaceae	*Pedicularis*	*Pedicularis odontochila*
Orobanchaceae	*Orobanche*	*Orobanche mongolica*

Orobanchaceae	*Orobanche*	*Orobanche ombrochares*
Orobanchaceae	*Brandisia*	*Brandisia hancei*
Orobanchaceae	*Euphrasia*	*Euphrasia pectinata subsp. sichuanica*
Orobanchaceae	*Pedicularis*	*Pedicularis bicolor*
Orobanchaceae	*Pedicularis*	*Pedicularis chinensis*
Orobanchaceae	*Pedicularis*	*Pedicularis curvituba subsp. provotii*
Orobanchaceae	*Pedicularis*	*Pedicularis davidii*
Orobanchaceae	*Pedicularis*	*Pedicularis decora*
Orobanchaceae	*Pedicularis*	*Pedicularis dissecta*
Orobanchaceae	*Pedicularis*	*Pedicularis giraldiana*
Orobanchaceae	*Pedicularis*	*Pedicularis muscicola*
Orobanchaceae	*Pedicularis*	*Pedicularis nasturtiifolia*
Orobanchaceae	*Pedicularis*	*Pedicularis odontochila*
Orobanchaceae	*Pedicularis*	*Pedicularis plicata*
Orobanchaceae	*Pedicularis*	*Pedicularis resupinata subsp. galeobdolon*
Orobanchaceae	*Pedicularis*	*Pedicularis resupinata subsp. lasiophylla*
Orobanchaceae	*Pedicularis*	*Pedicularis rudis*
Orobanchaceae	*Pedicularis*	*Pedicularis shansiensis*
Orobanchaceae	*Pedicularis*	*Pedicularis torta*
Orobanchaceae	*Rehmannia*	*Rehmannia piasezkii*
Paeoniaceae	*Paeonia*	*Paeonia ostii*
Paeoniaceae	*Paeonia*	*Paeonia suffruticosa*
Paeoniaceae	*Paeonia*	*Paeonia jishanensis*
Paeoniaceae	*Paeonia*	*Paeonia rockii*
Paeoniaceae	*Paeonia*	*Paeonia rockii subsp. taibaishanica*
Paeoniaceae	*Paeonia*	*Paeonia mairei*
Papaveraceae	*Corydalis*	*Corydalis anethifolia*
Papaveraceae	*Corydalis*	*Corydalis yanhusuo*
Papaveraceae	*Meconopsis*	*Meconopsis quintuplinervia var. glabra*
Papaveraceae	*Corydalis*	*Corydalis adunca*
Papaveraceae	*Corydalis*	*Corydalis fargesii*
Papaveraceae	*Corydalis*	*Corydalis giraldii*
Papaveraceae	*Corydalis*	*Corydalis linarioides*
Papaveraceae	*Corydalis*	*Corydalis tomentella*
Papaveraceae	*Corydalis*	*Corydalis wilsonii*
Papaveraceae	*Corydalis*	*Corydalis acuminata*
Papaveraceae	*Corydalis*	*Corydalis anethifolia*
Papaveraceae	*Corydalis*	*Corydalis chingii*

Papaveraceae	*Corydalis*	*Corydalis hemsleyana*
Papaveraceae	*Corydalis*	*Corydalis jingyuanensis*
Papaveraceae	*Corydalis*	*Corydalis nanwutaishanensis*
Papaveraceae	*Corydalis*	*Corydalis pseudoincisa*
Papaveraceae	*Corydalis*	*Corydalis shensiana*
Papaveraceae	*Corydalis*	*Corydalis ternatifolia*
Papaveraceae	*Corydalis*	*Corydalis trisecta*
Papaveraceae	*Corydalis*	*Corydalis gamosepala*
Papaveraceae	*Corydalis*	*Corydalis yanhusuo*
Papaveraceae	*Corydalis*	*Corydalis caudata*
Papaveraceae	*Corydalis*	*Corydalis cheilanthifolia*
Papaveraceae	*Corydalis*	*Corydalis saxicola*
Papaveraceae	*Dicranostigma*	*Dicranostigma leptopodum*
Papaveraceae	*Eomecon*	*Eomecon chionantha*
Papaveraceae	*Hylomecon*	*Hylomecon japonica var. subincisa*
Papaveraceae	*Meconopsis*	*Meconopsis oliveriana*
Papaveraceae	*Meconopsis*	*Meconopsis quintuplinervia*
Papaveraceae	*Meconopsis*	*Meconopsis quintuplinervia var. glabra*
Papaveraceae	*Stylophorum*	*Stylophorum lasiocarpum*
Papaveraceae	*Stylophorum*	*Stylophorum sutchuenense*
Pentaphylacaceae	*Cleyera*	*Cleyera lipingensis*
Pentaphylacaceae	*Eurya*	*Eurya alata*
Pentaphylacaceae	*Eurya*	*Eurya brevistyla*
Pentaphylacaceae	*Eurya*	*Eurya obtusifolia*
Phrymaceae	*Mimulus*	*Mimulus szechuanensis*
Phyllanthaceae	*Bischofia*	*Bischofia polycarpa*
Pinaceae	*Abies*	*Abies chensiensis*
Pinaceae	*Abies*	*Abies fargesii*
Pinaceae	*Larix*	*Larix potaninii var. chinensis*
Pinaceae	*Picea*	*Picea asperata*
Pinaceae	*Picea*	*Picea brachytyla*
Pinaceae	*Picea*	*Picea meyeri*
Pinaceae	*Picea*	*Picea neoveitchii*
Pinaceae	*Picea*	*Picea wilsonii*
Pinaceae	*Pinus*	*Pinus bungeana*
Pinaceae	*Pinus*	*Pinus massoniana*
Pinaceae	*Pinus*	*Pinus tabuliformis*
Pinaceae	*Pinus*	*Pinus taiwanensis*
Pinaceae	*Pseudotsuga*	*Pseudotsuga sinensis*
Pinaceae	*Tsuga*	*Tsuga chinensis*
Pinaceae	*Larix*	*Larix gmelinii var. principis-rupprechtii*

Pinaceae	*Pinus*	*Pinus tabuliformis var. henryi*
Pinaceae	*Pseudolarix*	*Pseudolarix amabilis*
Piperaceae	*Piper*	*Piper bambusifolium*
Pittosporaceae	*Pittosporum*	*Pittosporum heterophyllum*
Pittosporaceae	*Pittosporum*	*Pittosporum rehderianum*
Pittosporaceae	*Pittosporum*	*Pittosporum truncatum*
Plantaginaceae	*Pseudolysimachion*	*Pseudolysimachion linariifolium subsp. dilatatum*
Plantaginaceae	*Veronica*	*Veronica fargesii*
Plantaginaceae	*Veronica*	*Veronica henryi*
Plantaginaceae	*Veronica*	*Veronica rockii*
Plantaginaceae	*Veronica*	*Veronica tsinglingensis*
Plantaginaceae	*Veronica*	*Veronica vandellioides*
Plantaginaceae	*Veronicastrum*	*Veronicastrum latifolium*
Plantaginaceae	*Veronicastrum*	*Veronicastrum stenostachyum*
Poaceae	*Psathyrostachys*	*Psathyrostachys huashanica*
Poaceae	*Achnatherum*	*Achnatherum chinense*
Poaceae	*Achnatherum*	*Achnatherum chingii*
Poaceae	*Achnatherum*	*Achnatherum henryi*
Poaceae	*Agropyron*	*Agropyron mongolicum*
Poaceae	*Agrostis*	*Agrostis arisan-montana*
Poaceae	*Agrostis*	*Agrostis hugoniana*
Poaceae	*Aristida*	*Aristida triseta*
Poaceae	*Arundinaria*	*Arundinaria fargesii*
Poaceae	*Bambusa*	*Bambusa emeiensis*
Poaceae	*Bromus*	*Bromus plurinodis*
Poaceae	*Chimonobambusa*	*Chimonobambusa angustifolia*
Poaceae	*Chimonobambusa*	*Chimonobambusa purpurea*
Poaceae	*Cleistogenes*	*Cleistogenes caespitosa*
Poaceae	*Cleistogenes*	*Cleistogenes mucronata*
Poaceae	*Cleistogenes*	*Cleistogenes polyphylla*
Poaceae	*Deschampsia*	*Deschampsia cespitosa subsp. ivanovae*
Poaceae	*Deyeuxia*	*Deyeuxia conferta*
Poaceae	*Deyeuxia*	*Deyeuxia effusiflora*
Poaceae	*Elymus*	*Elymus barbicallus*
Poaceae	*Elymus*	*Elymus ciliaris var. lasiophyllus*
Poaceae	*Elymus*	*Elymus dahuricus var. cylindricus*
Poaceae	*Elymus*	*Elymus grandis*
Poaceae	*Elymus*	*Elymus hondae*
Poaceae	*Elymus*	*Elymus pendulinus subsp. multiculmis*
Poaceae	*Elymus*	*Elymus pendulinus subsp.*

		pubicaulis
Poaceae	*Elymus*	*Elymus serotinus*
Poaceae	*Elymus*	*Elymus shandongensis*
Poaceae	*Elymus*	*Elymus sinicus var. medius*
Poaceae	*Elymus*	*Elymus sinicus var. medius*
Poaceae	*Elymus*	*Elymus strictus*
Poaceae	*Fargesia*	*Fargesia decurvata*
Poaceae	*Fargesia*	*Fargesia dracocephala*
Poaceae	*Fargesia*	*Fargesia nitida*
Poaceae	*Fargesia*	*Fargesia qinlingensis*
Poaceae	*Fargesia*	*Fargesia spathacea*
Poaceae	*Festuca*	*Festuca fascinata*
Poaceae	*Festuca*	*Festuca sinensis*
Poaceae	*Helictotrichon*	*Helictotrichon delavayi*
Poaceae	*Helictotrichon*	*Helictotrichon leianthum*
Poaceae	*Indocalamus*	*Indocalamus bashanensis*
Poaceae	*Indocalamus*	*Indocalamus latifolius*
Poaceae	*Melica*	*Melica przewalskyi*
Poaceae	*Melica*	*Melica radula*
Poaceae	*Pennisetum*	*Pennisetum longissimum*
Poaceae	*Pennisetum*	*Pennisetum shaanxiense*
Poaceae	*Phyllostachys*	*Phyllostachys heteroclada*
Poaceae	*Phyllostachys*	*Phyllostachys sulphurea var. viridis*
Poaceae	*Piptatherum*	*Piptatherum tibeticum*
Poaceae	*Pleioblastus*	*Pleioblastus amarus*
Poaceae	*Poa*	*Poa faberi*
Poaceae	*Poa*	*Poa faberi var. longifolia*
Poaceae	*Poa*	*Poa szechuensis var. debilior*
Poaceae	*Psathyrostachys*	*Psathyrostachys huashanica*
Poaceae	*Shibataea*	*Shibataea chinensis*
Poaceae	*Spodiopogon*	*Spodiopogon tainanensis*
Poaceae	*Stipa*	*Stipa penicillata*
Poaceae	*Stipa*	*Stipa przewalskyi*
Poaceae	*Trisetum*	*Trisetum henryi*
Poaceae	*Trisetum*	*Trisetum pauciflorum*
Polygonaceae	*Rheum*	*Rheum tanguticum var. tanguticum/Rheum tanguticum*
Polygonaceae	*Antenoron*	*Antenoron filiforme var. neofiliforme*
Polygonaceae	*Atraphaxis*	*Atraphaxis manshurica*
Polygonaceae	*Fallopia*	*Fallopia aubertii*
Polygonaceae	*Fallopia*	*Fallopia cynanchoides*

Polygonaceae	*Polygonum*	*Polygonum darrisii*
Polygonaceae	*Polygonum*	*Polygonum runcinatum var. sinense*
Polygonaceae	*Polygonum*	*Polygonum sparsipilosum var. hubertii*
Polygonaceae	*Polygonum*	*Polygonum sparsipilosum var. hubertii*
Polygonaceae	*Polygonum*	*Polygonum suffultum var. pergracile*
Polygonaceae	*Rheum*	*Rheum hotaoense*
Polygonaceae	*Rheum*	*Rheum officinale*
Polygonaceae	*Rheum*	*Rheum palmatum*
Polygonaceae	*Rheum*	*Rheum tanguticum*
Polypodiaceae	*Drynaria*	*Drynaria baronii*
Polypodiaceae	*Loxogramme*	*Loxogramme salicifolia*
Polypodiaceae	*Lemmaphyllum*	*Lemmaphyllum diversum*
Polypodiaceae	*Lemmaphyllum*	*Lemmaphyllum drymoglossoides*
Polypodiaceae	*Lepisorus*	*Lepisorus albertii*
Polypodiaceae	*Lepisorus*	*Lepisorus angustus*
Polypodiaceae	*Lepisorus*	*Lepisorus bicolor*
Polypodiaceae	*Lepisorus*	*Lepisorus contortus*
Polypodiaceae	*Lepisorus*	*Lepisorus crassipes*
Polypodiaceae	*Lepisorus*	*Lepisorus likiangensis*
Polypodiaceae	*Lepisorus*	*Lepisorus loriformis*
Polypodiaceae	*Lepisorus*	*Lepisorus macrosphaerus*
Polypodiaceae	*Lepisorus*	*Lepisorus marginatus*
Polypodiaceae	*Lepisorus*	*Lepisorus thaipaiensis*
Polypodiaceae	*Neolepisorus*	*Neolepisorus ovatus*
Polypodiaceae	*Selliguea*	*Selliguea conjuncta*
Polypodiaceae	*Selliguea*	*Selliguea majoensis*
Polypodiaceae	*Selliguea*	*Selliguea shensiensis*
Polypodiaceae	*Polypodiodes*	*Polypodiodes amoena var. amoena/Polypodiodes amoena*
Polypodiaceae	*Polypodiodes*	*Polypodiodes chinensis*
Polypodiaceae	*Pyrrosia*	*Pyrrosia calvata*
Polypodiaceae	*Pyrrosia*	*Pyrrosia davidii*
Polypodiaceae	*Pyrrosia*	*Pyrrosia drakeana*
Primulaceae	*Primula*	*Primula filchnerae*
Primulaceae	*Primula*	*Primula rupestris*
Primulaceae	*Primula*	*Primula sinensis*
Primulaceae	*Androsace*	*Androsace engleri*
Primulaceae	*Androsace*	*Androsace laxa*

Primulaceae	*Lysimachia*	*Lysimachia crista-galli*
Primulaceae	*Lysimachia*	*Lysimachia pseudohenryi*
Primulaceae	*Lysimachia*	*Lysimachia auriculata*
Primulaceae	*Lysimachia*	*Lysimachia crispidens*
Primulaceae	*Lysimachia*	*Lysimachia pentapetala*
Primulaceae	*Lysimachia*	*Lysimachia silvestrii*
Primulaceae	*Lysimachia*	*Lysimachia stenosepala*
Primulaceae	*Primula*	*Primula alsophila*
Primulaceae	*Primula*	*Primula conspersa*
Primulaceae	*Primula*	*Primula filchnerae*
Primulaceae	*Primula*	*Primula giraldiana*
Primulaceae	*Primula*	*Primula handeliana*
Primulaceae	*Primula*	*Primula huashanensis*
Primulaceae	*Primula*	*Primula knuthiana*
Primulaceae	*Primula*	*Primula obconica*
Primulaceae	*Primula*	*Primula odontocalyx*
Primulaceae	*Primula*	*Primula rupestris*
Primulaceae	*Primula*	*Primula scopulorum*
Primulaceae	*Primula*	*Primula sinensis*
Primulaceae	*Primula*	*Primula stenocalyx*
Primulaceae	*Primula*	*Primula woodwardii*
Pteridaceae	*Adiantum*	*Adiantum davidii*
Pteridaceae	*Adiantum*	*Adiantum refractum*
Pteridaceae	*Adiantum*	*Adiantum erythrochlamys*
Pteridaceae	*Adiantum*	*Adiantum fimbriatum*
Pteridaceae	*Adiantum*	*Adiantum capillus-veneris*
Pteridaceae	*Adiantum*	*Adiantum myriosorum*
Pteridaceae	*Adiantum*	*Adiantum pedatum*
Pteridaceae	*Coniogramme*	*Coniogramme intermedia*
Pteridaceae	*Coniogramme*	*Coniogramme rosthornii*
Pteridaceae	*Coniogramme*	*Coniogramme sinensis*
Pteridaceae	*Coniogramme*	*Coniogramme suprapilosa*
Pteridaceae	*Coniogramme*	*Coniogramme wilsonii*
Pteridaceae	*Paragymnopteris*	*Paragymnopteris bipinnata var. auriculata*
Pteridaceae	*Paragymnopteris*	*Paragymnopteris delavayi*
Pteridaceae	*Pteris*	*Pteris actiniopteroides*
Pteridaceae	*Aleuritopteris*	*Aleuritopteris argentea*
Pteridaceae	*Aleuritopteris*	*Aleuritopteris duclouxii*
Pteridaceae	*Aleuritopteris*	*Aleuritopteris niphobola*
Pteridaceae	*Onychium*	*Onychium japonicum*
Pteridaceae	*Onychium*	*Onychium moupinense*
Pteridaceae	*Cheilanthes*	*Cheilanthes brausei*
Pteridaceae	*Aleuritopteris*	*Aleuritopteris albofusca*

Ranunculaceae	*Aconitum*	*Aconitum taipeicum*
Ranunculaceae	*Callianthemum*	*Callianthemum taipaicum*
Ranunculaceae	*Coptis*	*Coptis chinensis*
Ranunculaceae	*Helleborus*	*Helleborus thibetanus*
Ranunculaceae	*Hepatica*	*Hepatica henryi*
Ranunculaceae	*Aconitum*	*Aconitum cannabifolium*
Ranunculaceae	*Aconitum*	*Aconitum carmichaeli var. pubescens*
Ranunculaceae	*Aconitum*	*Aconitum flavum*
Ranunculaceae	*Aconitum*	*Aconitum henryi*
Ranunculaceae	*Aconitum*	*Aconitum pendulum*
Ranunculaceae	*Aconitum*	*Aconitum sinomontanum*
Ranunculaceae	*Aconitum*	*Aconitum taipeicum*
Ranunculaceae	*Aconitum*	*Aconitum tanguticum*
Ranunculaceae	*Adonis*	*Adonis sutchuenensis*
Ranunculaceae	*Anemone*	*Anemone rivularis var. flore-minore*
Ranunculaceae	*Anemone*	*Anemone taipaiensis*
Ranunculaceae	*Anemone*	*Anemone tomentosa*
Ranunculaceae	*Aquilegia*	*Aquilegia ecalcarata*
Ranunculaceae	*Aquilegia*	*Aquilegia incurvata*
Ranunculaceae	*Aquilegia*	*Aquilegia oxysepala var. kansuensis*
Ranunculaceae	*Aquilegia*	*Aquilegia yabeana*
Ranunculaceae	*Callianthemum*	*Callianthemum taipaicum*
Ranunculaceae	*Clematis*	*Clematis apiifolia var. argentilucida*
Ranunculaceae	*Clematis*	*Clematis armandii var. farquhariana*
Ranunculaceae	*Clematis*	*Clematis chinensis var. vestita*
Ranunculaceae	*Clematis*	*Clematis dasyandra*
Ranunculaceae	*Clematis*	*Clematis fruticosa var. lobata*
Ranunculaceae	*Clematis*	*Clematis grandidentata*
Ranunculaceae	*Clematis*	*Clematis gratopsis*
Ranunculaceae	*Clematis*	*Clematis henryi*
Ranunculaceae	*Clematis*	*Clematis henryi var. ternata*
Ranunculaceae	*Clematis*	*Clematis kirilowii*
Ranunculaceae	*Clematis*	*Clematis nannophylla*
Ranunculaceae	*Clematis*	*Clematis obscura*
Ranunculaceae	*Clematis*	*Clematis otophora*
Ranunculaceae	*Clematis*	*Clematis pashanensis*
Ranunculaceae	*Clematis*	*Clematis pashanensis var. latisepala*
Ranunculaceae	*Clematis*	*Clematis peterae*

Ranunculaceae	*Clematis*	*Clematis peterae var. trichocarpa*
Ranunculaceae	*Clematis*	*Clematis pogonandra*
Ranunculaceae	*Clematis*	*Clematis potaninii*
Ranunculaceae	*Clematis*	*Clematis pseudootophora*
Ranunculaceae	*Clematis*	*Clematis puberula var. ganpiniana*
Ranunculaceae	*Clematis*	*Clematis puberula var. tenuisepala*
Ranunculaceae	*Clematis*	*Clematis shensiensis*
Ranunculaceae	*Clematis*	*Clematis tomentella*
Ranunculaceae	*Clematis*	*Clematis uncinata var. coriacea*
Ranunculaceae	*Coptis*	*Coptis chinensis*
Ranunculaceae	*Delphinium*	*Delphinium anthriscifolium var. majus*
Ranunculaceae	*Delphinium*	*Delphinium giraldii*
Ranunculaceae	*Delphinium*	*Delphinium grandiflorum var. leiocarpum*
Ranunculaceae	*Delphinium*	*Delphinium grandiflorum var. gilgianum*
Ranunculaceae	*Delphinium*	*Delphinium henryi*
Ranunculaceae	*Delphinium*	*Delphinium hirticaule*
Ranunculaceae	*Delphinium*	*Delphinium honanense*
Ranunculaceae	*Delphinium*	*Delphinium honanense var. piliferum*
Ranunculaceae	*Delphinium*	*Delphinium potaninii*
Ranunculaceae	*Delphinium*	*Delphinium siwanense*
Ranunculaceae	*Delphinium*	*Delphinium taipaicum*
Ranunculaceae	*Dichocarpum*	*Dichocarpum fargesii*
Ranunculaceae	*Dichocarpum*	*Dichocarpum franchetii*
Ranunculaceae	*Helleborus*	*Helleborus thibetanus*
Ranunculaceae	*Hepatica*	*Hepatica henryi*
Ranunculaceae	*Kingdonia*	*Kingdonia uniflora*
Ranunculaceae	*Ranunculus*	*Ranunculus dielsianus var. suprasericeus*
Ranunculaceae	*Ranunculus*	*Ranunculus nematolobus*
Ranunculaceae	*Ranunculus*	*Ranunculus petrogeiton*
Ranunculaceae	*Ranunculus*	*Ranunculus sinovaginatus*
Ranunculaceae	*Ranunculus*	*Ranunculus tanguticus var. dasycarpus*
Ranunculaceae	*Thalictrum*	*Thalictrum brevisericeum*
Ranunculaceae	*Thalictrum*	*Thalictrum fargesii*
Ranunculaceae	*Thalictrum*	*Thalictrum foeniculaceum*

Ranunculaceae	*Thalictrum*	*Thalictrum ichangense*
Ranunculaceae	*Thalictrum*	*Thalictrum oligandrum*
Ranunculaceae	*Thalictrum*	*Thalictrum przewalskii*
Ranunculaceae	*Thalictrum*	*Thalictrum robustum*
Ranunculaceae	*Thalictrum*	*Thalictrum shensiense*
Ranunculaceae	*Thalictrum*	*Thalictrum tenue*
Ranunculaceae	*Thalictrum*	*Thalictrum uncinulatum*
Ranunculaceae	*Trollius*	*Trollius buddae*
Ranunculaceae	*Trollius*	*Trollius farreri*
Ranunculaceae	*Berchemia*	*Berchemia polyphylla var. leioclada*
Ranunculaceae	*Berchemia*	*Berchemia sinica*
Ranunculaceae	*Berchemia*	*Berchemia yunnanensis*
Rhamnaceae	*Rhamnella*	*Rhamnella martinii*
Rhamnaceae	*Rhamnus*	*Rhamnus arguta*
Rhamnaceae	*Rhamnus*	*Rhamnus arguta var. velutina*
Rhamnaceae	*Rhamnus*	*Rhamnus bungeana*
Rhamnaceae	*Rhamnus*	*Rhamnus dumetorum*
Rhamnaceae	*Rhamnus*	*Rhamnus esquirolii*
Rhamnaceae	*Rhamnus*	*Rhamnus hemsleyana*
Rhamnaceae	*Rhamnus*	*Rhamnus heterophylla*
Rhamnaceae	*Rhamnus*	*Rhamnus hupehensis*
Rhamnaceae	*Rhamnus*	*Rhamnus iteinophylla*
Rhamnaceae	*Rhamnus*	*Rhamnus leptophylla*
Rhamnaceae	*Rhamnus*	*Rhamnus rosthornii*
Rhamnaceae	*Rhamnus*	*Rhamnus rugulosa*
Rhamnaceae	*Rhamnus*	*Rhamnus rugulosa var. glabrata*
Rhamnaceae	*Rhamnus*	*Rhamnus sargentiana*
Rhamnaceae	*Rhamnus*	*Rhamnus tangutica*
Rhamnaceae	*Rhamnus*	*Rhamnus utilis var. hypochrysa*
Rhamnaceae	*Sageretia*	*Sageretia paucicostata*
Rhamnaceae	*Sageretia*	*Sageretia pycnophylla*
Rhamnaceae	*Sageretia*	*Sageretia subcaudata*
Rhamnaceae	*Ziziphus*	*Ziziphus jujuba var. inermis*
Rosaceae	*Sorbus*	*Sorbus tsinlingensis*
Rosaceae	*Sorbus*	*Sorbus yuana*
Rosaceae	*Amelanchier*	*Amelanchier sinica*
Rosaceae	*Amygdalus*	*Amygdalus davidiana*
Rosaceae	*Amygdalus*	*Amygdalus davidiana var. potaninii*
Rosaceae	*Amygdalus*	*Amygdalus kansuensis*
Rosaceae	*Amygdalus*	*Amygdalus tangutica*
Rosaceae	*Armeniaca*	*Armeniaca holosericea*

Rosaceae	*Armeniaca*	*Armeniaca vulgaris var. zhidanensis*
Rosaceae	*Cerasus*	*Cerasus clarofolia*
Rosaceae	*Cerasus*	*Cerasus conadenia*
Rosaceae	*Cerasus*	*Cerasus conradinae*
Rosaceae	*Cerasus*	*Cerasus discadenia*
Rosaceae	*Cerasus*	*Cerasus dolichadenia*
Rosaceae	*Cerasus*	*Cerasus humilis*
Rosaceae	*Cerasus*	*Cerasus polytricha*
Rosaceae	*Cerasus*	*Cerasus pseudocerasus*
Rosaceae	*Cerasus*	*Cerasus schneideriana*
Rosaceae	*Cerasus*	*Cerasus serrulata var. pubescens*
Rosaceae	*Cerasus*	*Cerasus setulosa*
Rosaceae	*Cerasus*	*Cerasus stipulacea*
Rosaceae	*Cerasus*	*Cerasus szechuanica*
Rosaceae	*Cerasus*	*Cerasus tatsienensis*
Rosaceae	*Cerasus*	*Cerasus tomentosa*
Rosaceae	*Chaenomeles*	*Chaenomeles cathayensis*
Rosaceae	*Chaenomeles*	*Chaenomeles sinensis*
Rosaceae	*Coluria*	*Coluria omeiensis var. nanzhengensis*
Rosaceae	*Cotoneaster*	*Cotoneaster acutifolius var. villosulus*
Rosaceae	*Cotoneaster*	*Cotoneaster apiculatus*
Rosaceae	*Cotoneaster*	*Cotoneaster dielsianus*
Rosaceae	*Cotoneaster*	*Cotoneaster dielsianus*
Rosaceae	*Cotoneaster*	*Cotoneaster divaricatus*
Rosaceae	*Cotoneaster*	*Cotoneaster gracilis*
Rosaceae	*Cotoneaster*	*Cotoneaster horizontalis var. perpusillus*
Rosaceae	*Cotoneaster*	*Cotoneaster moupinensis*
Rosaceae	*Cotoneaster*	*Cotoneaster multiflorus var. calocarpus*
Rosaceae	*Cotoneaster*	*Cotoneaster obscurus*
Rosaceae	*Cotoneaster*	*Cotoneaster salicifolius*
Rosaceae	*Cotoneaster*	*Cotoneaster salicifolius var. rugosus*
Rosaceae	*Cotoneaster*	*Cotoneaster silvestrii*
Rosaceae	*Cotoneaster*	*Cotoneaster tenuipes*
Rosaceae	*Cotoneaster*	*Cotoneaster zabelii*
Rosaceae	*Crataegus*	*Crataegus aurantia*
Rosaceae	*Crataegus*	*Crataegus hupehensis*
Rosaceae	*Crataegus*	*Crataegus kansuensis*

Rosaceae	*Crataegus*	*Crataegus shensiensis*
Rosaceae	*Crataegus*	*Crataegus wilsonii*
Rosaceae	*Exochorda*	*Exochorda giraldii*
Rosaceae	*Fragaria*	*Fragaria gracilis*
Rosaceae	*Fragaria*	*Fragaria moupinensis*
Rosaceae	*Fragaria*	*Fragaria nilgerrensis var. mairei*
Rosaceae	*Fragaria*	*Fragaria pentaphylla*
Rosaceae	*Geum*	*Geum japonicum var. chinense*
Rosaceae	*Maddenia*	*Maddenia incisoserrata*
Rosaceae	*Maddenia*	*Maddenia wilsonii*
Rosaceae	*Malus*	*Malus honanensis*
Rosaceae	*Malus*	*Malus hupehensis*
Rosaceae	*Malus*	*Malus kansuensis*
Rosaceae	*Malus*	*Malus kansuensis var. calva*
Rosaceae	*Malus*	*Malus transitoria*
Rosaceae	*Malus*	*Malus transitoria var. centralasiatica*
Rosaceae	*Malus*	*Malus yunnanensis var. veitchii*
Rosaceae	*Neillia*	*Neillia ribesioides*
Rosaceae	*Neillia*	*Neillia sinensis*
Rosaceae	*Osteomeles*	*Osteomeles schwerinae var. microphylla*
Rosaceae	*Padus*	*Padus brachypoda*
Rosaceae	*Padus*	*Padus obtusata*
Rosaceae	*Padus*	*Padus stellipila*
Rosaceae	*Padus*	*Padus velutina*
Rosaceae	*Padus*	*Padus wilsonii*
Rosaceae	*Photinia*	*Photinia beauverdiana var. brevifolia*
Rosaceae	*Photinia*	*Photinia parvifolia*
Rosaceae	*Photinia*	*Photinia schneideriana*
Rosaceae	*Photinia*	*Photinia villosa var. sinica*
Rosaceae	*Potentilla*	*Potentilla potaninii var. compsophylla*
Rosaceae	*Potentilla*	*Potentilla pseudosimulatrix*
Rosaceae	*Potentilla*	*Potentilla saundersiana var. caespitosa*
Rosaceae	*Potentilla*	*Potentilla simulatrix*
Rosaceae	*Potentilla*	*Potentilla sischanensis var. peterae*
Rosaceae	*Prinsepia*	*Prinsepia uniflora*
Rosaceae	*Prinsepia*	*Prinsepia uniflora var. serrata*

Rosaceae	*Pyracantha*	*Pyracantha angustifolia*
Rosaceae	*Pyracantha*	*Pyracantha atalantioides*
Rosaceae	*Pyracantha*	*Pyracantha fortuneana*
Rosaceae	*Pyrus*	*Pyrus phaeocarpa*
Rosaceae	*Pyrus*	*Pyrus serrulata*
Rosaceae	*Pyrus*	*Pyrus sinkiangensis*
Rosaceae	*Pyrus*	*Pyrus xerophila*
Rosaceae	*Rosa*	*Rosa banksiae var. normalis*
Rosaceae	*Rosa*	*Rosa banksiopsis*
Rosaceae	*Rosa*	*Rosa bella*
Rosaceae	*Rosa*	*Rosa bella var. nuda*
Rosaceae	*Rosa*	*Rosa caudata*
Rosaceae	*Rosa*	*Rosa caudata var. maxima*
Rosaceae	*Rosa*	*Rosa corymbulosa*
Rosaceae	*Rosa*	*Rosa cymosa var. puberula*
Rosaceae	*Rosa*	*Rosa davidii*
Rosaceae	*Rosa*	*Rosa filipes*
Rosaceae	*Rosa*	*Rosa giraldii*
Rosaceae	*Rosa*	*Rosa giraldii var. venulosa*
Rosaceae	*Rosa*	*Rosa glomerata*
Rosaceae	*Rosa*	*Rosa henryi*
Rosaceae	*Rosa*	*Rosa hugonis*
Rosaceae	*Rosa*	*Rosa moyesii*
Rosaceae	*Rosa*	*Rosa moyesii var. pubescens*
Rosaceae	*Rosa*	*Rosa multiflora var. cathayensis*
Rosaceae	*Rosa*	*Rosa omeiensis*
Rosaceae	*Rosa*	*Rosa prattii*
Rosaceae	*Rosa*	*Rosa primula*
Rosaceae	*Rosa*	*Rosa roxburghii f. normalis*
Rosaceae	*Rosa*	*Rosa rubus*
Rosaceae	*Rosa*	*Rosa sertata*
Rosaceae	*Rosa*	*Rosa setipoda*
Rosaceae	*Rosa*	*Rosa sweginzowii*
Rosaceae	*Rosa*	*Rosa sweginzowii var. glandulosa*
Rosaceae	*Rosa*	*Rosa willmottiana*
Rosaceae	*Rosa*	*Rosa xanthina*
Rosaceae	*Rosa*	*Rosa xanthina f. normalis*
Rosaceae	*Rubus*	*Rubus amabilis*
Rosaceae	*Rubus*	*Rubus bambusarum*
Rosaceae	*Rubus*	*Rubus cockburnianus*
Rosaceae	*Rubus*	*Rubus coreanus var. tomentosus*

Rosaceae	*Rubus*	*Rubus eucalyptus*
Rosaceae	*Rubus*	*Rubus eustephanos*
Rosaceae	*Rubus*	*Rubus flagelliflorus*
Rosaceae	*Rubus*	*Rubus flosculosus*
Rosaceae	*Rubus*	*Rubus forrestianus*
Rosaceae	*Rubus*	*Rubus grandipaniculatus*
Rosaceae	*Rubus*	*Rubus ichangensis*
Rosaceae	*Rubus*	*Rubus idaeopsis*
Rosaceae	*Rubus*	*Rubus innominatus*
Rosaceae	*Rubus*	*Rubus innominatus var. kuntzeanus*
Rosaceae	*Rubus*	*Rubus inopertus var. echinocalyx*
Rosaceae	*Rubus*	*Rubus lasiostylus*
Rosaceae	*Rubus*	*Rubus mesogaeus var. oxycomus*
Rosaceae	*Rubus*	*Rubus parkeri*
Rosaceae	*Rubus*	*Rubus pileatus*
Rosaceae	*Rubus*	*Rubus piluliferus*
Rosaceae	*Rubus*	*Rubus pinnatisepalus*
Rosaceae	*Rubus*	*Rubus playfairianus*
Rosaceae	*Rubus*	*Rubus pungens var. villosus*
Rosaceae	*Rubus*	*Rubus simplex*
Rosaceae	*Rubus*	*Rubus subcoreanus*
Rosaceae	*Rubus*	*Rubus subtibetanus*
Rosaceae	*Rubus*	*Rubus thibetanus*
Rosaceae	*Rubus*	*Rubus xanthocarpus*
Rosaceae	*Sibbaldia*	*Sibbaldia procumbens var. aphanopetala*
Rosaceae	*Sorbaria*	*Sorbaria arborea*
Rosaceae	*Sorbaria*	*Sorbaria arborea var. glabrata*
Rosaceae	*Sorbaria*	*Sorbaria arborea var. subtomentosa*
Rosaceae	*Sorbaria*	*Sorbaria kirilowii*
Rosaceae	*Sorbus*	*Sorbus discolor*
Rosaceae	*Sorbus*	*Sorbus folgneri*
Rosaceae	*Sorbus*	*Sorbus hemsleyi*
Rosaceae	*Sorbus*	*Sorbus hupehensis*
Rosaceae	*Sorbus*	*Sorbus koehneana*
Rosaceae	*Sorbus*	*Sorbus pohuashanensis*
Rosaceae	*Sorbus*	*Sorbus tapashana*
Rosaceae	*Sorbus*	*Sorbus tsinlingensis*
Rosaceae	*Sorbus*	*Sorbus yuana*
Rosaceae	*Spiraea*	*Spiraea blumei var. pubicarpa*

Rosaceae	*Spiraea*	*Spiraea chinensis*
Rosaceae	*Spiraea*	*Spiraea dasyantha*
Rosaceae	*Spiraea*	*Spiraea fritschiana var. angulata*
Rosaceae	*Spiraea*	*Spiraea henryi*
Rosaceae	*Spiraea*	*Spiraea hirsuta*
Rosaceae	*Spiraea*	*Spiraea hirsuta var. rotundifolia*
Rosaceae	*Spiraea*	*Spiraea japonica var. acuminata*
Rosaceae	*Spiraea*	*Spiraea japonica var. fortunei*
Rosaceae	*Spiraea*	*Spiraea longigemmis*
Rosaceae	*Spiraea*	*Spiraea miyabei var. glabrata*
Rosaceae	*Spiraea*	*Spiraea miyabei var. pilosula*
Rosaceae	*Spiraea*	*Spiraea mollifolia*
Rosaceae	*Spiraea*	*Spiraea mongolica*
Rosaceae	*Spiraea*	*Spiraea myrtilloides*
Rosaceae	*Spiraea*	*Spiraea ovalis*
Rosaceae	*Spiraea*	*Spiraea prostrata*
Rosaceae	*Spiraea*	*Spiraea prunifolia var. hupehensis*
Rosaceae	*Spiraea*	*Spiraea pubescens var. lasiocarpa*
Rosaceae	*Spiraea*	*Spiraea rosthornii*
Rosaceae	*Spiraea*	*Spiraea schneideriana*
Rosaceae	*Spiraea*	*Spiraea schneideriana var. amphidoxa*
Rosaceae	*Spiraea*	*Spiraea uratensis*
Rosaceae	*Spiraea*	*Spiraea veitchii*
Rosaceae	*Spiraea*	*Spiraea wilsonii*
Rosaceae	*Stranvaesia*	*Stranvaesia davidiana var. undulata*
Rubiaceae	*Damnacanthus*	*Damnacanthus officinarum*
Rubiaceae	*Emmenopterys*	*Emmenopterys henryi*
Rubiaceae	*Galium*	*Galium bungei var. angustifolium*
Rubiaceae	*Galium*	*Galium bungei var. hispidum*
Rubiaceae	*Galium*	*Galium crassifolium*
Rubiaceae	*Leptodermis*	*Leptodermis buxifolia*
Rubiaceae	*Leptodermis*	*Leptodermis pilosa*
Rubiaceae	*Leptodermis*	*Leptodermis potanini*
Rubiaceae	*Rubia*	*Rubia ovatifolia*
Rubiaceae	*Theligonum*	*Theligonum macranthum*
Rubiaceae	*Uncaria*	*Uncaria sinensis*

Rutaceae	*Citrus*	*Citrus japonica*
Rutaceae	*Zanthoxylum*	*Zanthoxylum molle*
Rutaceae	*Citrus*	*Citrus japonica*
Rutaceae	*Skimmia*	*Skimmia melanocarpa*
Rutaceae	*Zanthoxylum*	*Zanthoxylum armatum var. ferrugineum*
Rutaceae	*Zanthoxylum*	*Zanthoxylum bungeanum var. pubescens*
Rutaceae	*Zanthoxylum*	*Zanthoxylum dissitum*
Rutaceae	*Zanthoxylum*	*Zanthoxylum micranthum*
Rutaceae	*Zanthoxylum*	*Zanthoxylum molle*
Rutaceae	*Zanthoxylum*	*Zanthoxylum dimorphophyllum var. spinifolium*
Rutaceae	*Zanthoxylum*	*Zanthoxylum piasezkii*
Rutaceae	*Zanthoxylum*	*Zanthoxylum pilosulum*
Rutaceae	*Zanthoxylum*	*Zanthoxylum simulans*
Rutaceae	*Zanthoxylum*	*Zanthoxylum stenophyllum*
Rutaceae	*Zanthoxylum*	*Zanthoxylum undulatifolium*
Sabiaceae	*Meliosma*	*Meliosma cuneifolia*
Sabiaceae	*Meliosma*	*Meliosma cuneifolia var. glabriuscula*
Sabiaceae	*Meliosma*	*Meliosma flexuosa*
Sabiaceae	*Meliosma*	*Meliosma myriantha var. pilosa*
Sabiaceae	*Meliosma*	*Meliosma veitchiorum*
Sabiaceae	*Sabia*	*Sabia campanulata subsp. ritchieae*
Sabiaceae	*Sabia*	*Sabia schumanniana*
Sabiaceae	*Sabia*	*Sabia schumanniana subsp. pluriflora*
Salicaceae	*Populus*	*Populus pseudomaximowiczii*
Salicaceae	*Salix*	*Salix permollis*
Salicaceae	*Salix*	*Salix pingliensis*
Salicaceae	*Salix*	*Salix pseudopermollis*
Salicaceae	*Salix*	*Salix pseudotangii*
Salicaceae	*Salix*	*Salix yuhuangshanensis*
Salicaceae	*Carrierea*	*Carrierea calycina*
Salicaceae	*Poliothyrsis*	*Poliothyrsis sinensis*
Salicaceae	*Populus*	*Populus adenopoda*
Salicaceae	*Populus*	*Populus cathayana*
Salicaceae	*Populus*	*Populus cathayana var. latifolia*
Salicaceae	*Populus*	*Populus lasiocarpa*

Salicaceae	*Populus*	*Populus ningshanica*
Salicaceae	*Populus*	*Populus pseudomaximowiczii*
Salicaceae	*Populus*	*Populus pseudosimonii*
Salicaceae	*Populus*	*Populus purdomii*
Salicaceae	*Populus*	*Populus rotundifolia var. duclouxiana*
Salicaceae	*Populus*	*Populus simonii f. rhombifolia*
Salicaceae	*Populus*	*Populus szechuanica*
Salicaceae	*Populus*	*Populus tomentosa*
Salicaceae	*Populus*	*Populus tomentosa var. truncata*
Salicaceae	*Populus*	*Populus wilsonii*
Salicaceae	*Salix*	*Salix alfredii*
Salicaceae	*Salix*	*Salix bikouensis*
Salicaceae	*Salix*	*Salix biondiana*
Salicaceae	*Salix*	*Salix capitata*
Salicaceae	*Salix*	*Salix cathayana*
Salicaceae	*Salix*	*Salix chaenomeloides var. glandulifolia*
Salicaceae	*Salix*	*Salix characta*
Salicaceae	*Salix*	*Salix cheilophila*
Salicaceae	*Salix*	*Salix cupularis*
Salicaceae	*Salix*	*Salix fargesii*
Salicaceae	*Salix*	*Salix fargesii var. kansuensis*
Salicaceae	*Salix*	*Salix heterochroma*
Salicaceae	*Salix*	*Salix hylonoma*
Salicaceae	*Salix*	*Salix hypoleuca*
Salicaceae	*Salix*	*Salix hypoleuca var. platyphylla*
Salicaceae	*Salix*	*Salix koreensis var. pedunculata*
Salicaceae	*Salix*	*Salix linearistipularis*
Salicaceae	*Salix*	*Salix liouana*
Salicaceae	*Salix*	*Salix luctuosa*
Salicaceae	*Salix*	*Salix matsudana*
Salicaceae	*Salix*	*Salix matsudana f. pendula*
Salicaceae	*Salix*	*Salix paraplesia*
Salicaceae	*Salix*	*Salix permollis*
Salicaceae	*Salix*	*Salix pingliensis*
Salicaceae	*Salix*	*Salix polyclona*
Salicaceae	*Salix*	*Salix psammophila*
Salicaceae	*Salix*	*Salix pseudopermollis*
Salicaceae	*Salix*	*Salix pseudotangii*
Salicaceae	*Salix*	*Salix rehderiana*

Salicaceae	*Salix*	*Salix rhoophila*
Salicaceae	*Salix*	*Salix rosthornii*
Salicaceae	*Salix*	*Salix shihtsuanensis*
Salicaceae	*Salix*	*salix shihtsuanensis var. sessilis*
Salicaceae	*Salix*	*Salix sinica*
Salicaceae	*Salix*	*Salix sinica var. dentata*
Salicaceae	*Salix*	*Salix sinopurpurea*
Salicaceae	*Salix*	*Salix spathulifolia*
Salicaceae	*Salix*	*Salix taipaiensis*
Salicaceae	*Salix*	*Salix tangii*
Salicaceae	*Salix*	*Salix variegata*
Salicaceae	*Salix*	*Salix wangiana*
Salicaceae	*Salix*	*Salix wilsonii*
Salicaceae	*Salix*	*Salix yuhuangshanensis*
Santalaceae	*Buckleya*	*Buckleya graebneriana*
Santalaceae	*Buckleya*	*Buckleya henryi*
Santalaceae	*Viscum*	*Viscum diospyrosicola*
Santalaceae	*Viscum*	*Viscum fargesii*
Sapindaceae	*Acer*	*Acer cappadocicum subsp. sinicum*
Sapindaceae	*Acer*	*Acer fulvescens*
Sapindaceae	*Acer*	*Acer griseum*
Sapindaceae	*Acer*	*Acer miaotaiense*
Sapindaceae	*Acer*	*Acer pilosum*
Sapindaceae	*Acer*	*Acer tsinglingense*
Sapindaceae	*Acer*	*Acer buergerianum var. kaiscianense*
Sapindaceae	*Acer*	*Acer ceriferum*
Sapindaceae	*Acer*	*Acer davidii subsp. Grosseri*
Sapindaceae	*Acer*	*Acer erianthum*
Sapindaceae	*Acer*	*Acer henryi*
Sapindaceae	*Acer*	*Acer longipes*
Sapindaceae	*Acer*	*Acer maximowiczii*
Sapindaceae	*Acer*	*Acer oliverianum*
Sapindaceae	*Acer*	*Acer pictum subsp. Tricuspis*
Sapindaceae	*Acer*	*Acer pilosum var. stenolobum*
Sapindaceae	*Acer*	*Acer shenkanense*
Sapindaceae	*Acer*	*Acer sinense*
Sapindaceae	*Acer*	*Acer sterculiaceum subsp. Franchetii*
Sapindaceae	*Acer*	*Acer tataricum subsp. Theiferum*
Sapindaceae	*Dipteronia*	*Dipteronia sinensis*

Sapindaceae	*Aesculus*	*Aesculus chinensis*
Sapindaceae	*Aesculus*	*Aesculus chinensis var. wilsonii*
Sapindaceae	*Sapindus*	*Sapindus delavayi*
Saxifragaceae	*Bergenia*	*Bergenia scopulosa*
Saxifragaceae	*Chrysosplenium*	*Chrysosplenium taibaishanense*
Saxifragaceae	*Astilbe*	*Astilbe rivularis var. myriantha*
Saxifragaceae	*Bergenia*	*Bergenia scopulosa*
Saxifragaceae	*Chrysosplenium*	*Chrysosplenium biondianum*
Saxifragaceae	*Chrysosplenium*	*Chrysosplenium giraldianum*
Saxifragaceae	*Chrysosplenium*	*Chrysosplenium macrophyllum*
Saxifragaceae	*Chrysosplenium*	*Chrysosplenium microspermum*
Saxifragaceae	*Chrysosplenium*	*Chrysosplenium qinlingense*
Saxifragaceae	*Chrysosplenium*	*Chrysosplenium taibaishanense*
Saxifragaceae	*Saxifraga*	*Saxifraga aurantiaca*
Saxifragaceae	*Saxifraga*	*Saxifraga gemmigera*
Saxifragaceae	*Saxifraga*	*Saxifraga giraldiana*
Saxifragaceae	*Saxifraga*	*Saxifraga heleonastes*
Saxifragaceae	*Saxifraga*	*Saxifraga josephii*
Saxifragaceae	*Saxifraga*	*Saxifraga pseudohirculus*
Saxifragaceae	*Saxifraga*	*Saxifraga rufescens var. flabellifolia*
Saxifragaceae	*Saxifraga*	*Saxifraga rufescens var. flabellifolia*
Schisandraceae	*Illicium*	*Illicium henryi*
Schisandraceae	*Illicium*	*Illicium lanceolatum*
Schisandraceae	*Kadsura*	*Kadsura longipedunculata*
Schisandraceae	*Schisandra*	*Schisandra lancifolia*
Schisandraceae	*Schisandra*	*Schisandra propinqua subsp. sinensis*
Schisandraceae	*Schisandra*	*Schisandra sphenanthera*
Scrophulariaceae	*Scrophularia*	*Scrophularia stylosa*
Scrophulariaceae	*Buddleja*	*Buddleja albiflora*
Scrophulariaceae	*Buddleja*	*Buddleja alternifolia*
Scrophulariaceae	*Buddleja*	*Buddleja lindleyana*
Scrophulariaceae	*Buddleja*	*Buddleja nivea*
Scrophulariaceae	*Scrophularia*	*Scrophularia buergeriana var. tsinglingensis*
Scrophulariaceae	*Scrophularia*	*Scrophularia fargesii*
Scrophulariaceae	*Scrophularia*	*Scrophularia modesta*
Scrophulariaceae	*Scrophularia*	*Scrophularia stylosa*
Selaginellaceae	*Selaginella*	*Selaginella davidii*

Selaginellaceae	*Selaginella*	*Selaginella prostrata*
Selaginellaceae	*Selaginella*	*Selaginella sinensis*
Selaginellaceae	*Selaginella*	*Selaginella uncinata*
Simaroubaceae	*Ailanthus*	*Ailanthus giraldii*
Simaroubaceae	*Ailanthus*	*Ailanthus vilmoriniana*
Smilacaceae	*Smilax*	*Smilax cocculoides*
Smilacaceae	*Smilax*	*Smilax discotis*
Smilacaceae	*Smilax*	*Smilax glaucochina*
Smilacaceae	*Smilax*	*Smilax megalantha*
Smilacaceae	*Smilax*	*Smilax microphylla*
Smilacaceae	*Smilax*	*Smilax nigrescens*
Smilacaceae	*Smilax*	*Smilax polycolea*
Smilacaceae	*Smilax*	*Smilax riparia var. acuminata*
Smilacaceae	*Smilax*	*Smilax riparia var. pubescens*
Smilacaceae	*Smilax*	*Smilax scobinicaulis*
Smilacaceae	*Smilax*	*Smilax trachypoda*
Smilacaceae	*Smilax*	*Smilax vanchingshanensis*
Solanaceae	*Physochlaina*	*Physochlaina infundibularis*
Solanaceae	*Lycium*	*Lycium barbarum*
Solanaceae	*Physochlaina*	*Physochlaina infundibularis*
Staphyleaceae	*Staphylea*	*Staphylea holocarpa*
Styracaceae	*Sinojackia*	*Sinojackia xylocarpa*
Styracaceae	*Sinojackia*	*Sinojackia xylocarpa*
Styracaceae	*Styrax*	*Styrax bashanensis*
Styracaceae	*Styrax*	*Styrax confusus*
Styracaceae	*Styrax*	*Styrax hemsleyanus*
Styracaceae	*Styrax*	*Styrax japonicus var. nervillosus*
Styracaceae	*Styrax*	*Styrax odoratissimus*
Styracaceae	*Styrax*	*Styrax roseus*
Tamaricaceae	*Myricaria*	*Myricaria paniculata*
Tamaricaceae	*Myricaria*	*Myricaria platyphylla*
Tamaricaceae	*Tamarix*	*Tamarix austromongolica*
Tapisciaceae	*Tapiscia*	*Tapiscia sinensis*
Taxaceae	*Torreya*	*Torreya fargesii*
Taxaceae	*Cephalotaxus*	*Cephalotaxus sinensis*
Theaceae	*Camellia*	*Camellia cuspidata*
Thymelaeaceae	*Daphne*	*Daphne gemmata*
Thymelaeaceae	*Daphne*	*Daphne acutiloba*
Thymelaeaceae	*Daphne*	*Daphne gemmata*
Thymelaeaceae	*Daphne*	*Daphne giraldii*
Thymelaeaceae	*Daphne*	*Daphne tangutica*
Thymelaeaceae	*Daphne*	*Daphne tangutica var. wilsonii*
Thymelaeaceae	*Wikstroemia*	*Wikstroemia angustifolia*

Thymelaeaceae	*Wikstroemia*	*Wikstroemia ligustrina*
Thymelaeaceae	*Wikstroemia*	*Wikstroemia linoides*
Thymelaeaceae	*Wikstroemia*	*Wikstroemia micrantha*
Thymelaeaceae	*Wikstroemia*	*Wikstroemia pampaninii*
Thymelaeaceae	*Wikstroemia*	*Wikstroemia stenophylla*
Torricelliaceae	*Torricellia*	*Toricellia angulata*
Ulmaceae	*Ulmus*	*Ulmus elongata*
Ulmaceae	*Ulmus*	*Ulmus bergmanniana*
Ulmaceae	*Ulmus*	*Ulmus bergmanniana var. lasiophylla*
Ulmaceae	*Ulmus*	*Ulmus elongata*
Ulmaceae	*Ulmus*	*Ulmus glaucescens*
Ulmaceae	*Zelkova*	*Zelkova schneideriana*
Ulmaceae	*Zelkova*	*Zelkova sinica*
Urticaceae	*Elatostema*	*Elatostema stewardii*
Urticaceae	*Girardinia*	*Girardinia diversifolia subsp. triloba*
Urticaceae	*Pilea*	*Pilea pumila var. obtusifolia*
Urticaceae	*Pilea*	*Pilea receptacularis*
Violaceae	*Viola*	*Viola davidii*
Violaceae	*Viola*	*Viola hancockii*
Violaceae	*Viola*	*Viola kosanensis*
Violaceae	*Viola*	*Viola mongolica*
Violaceae	*Viola*	*Viola pekinensis*
Violaceae	*Viola*	*Viola pendulicarpa*
Violaceae	*Viola*	*Viola rupestris subsp. licentii*
Violaceae	*Viola*	*Viola sphaerocarpa*
Violaceae	*Viola*	*Viola stewardiana*
Violaceae	*Viola*	*Viola striatella*
Vitaceae	*Vitis*	*Vitis bashanica*
Vitaceae	*Ampelopsis*	*Ampelopsis bodinieri*
Vitaceae	*Ampelopsis*	*Ampelopsis bodinieri var. cinerea*
Vitaceae	*Ampelopsis*	*Ampelopsis chaffanjonii*
Vitaceae	*Ampelopsis*	*Ampelopsis delavayana*
Vitaceae	*Ampelopsis*	*Ampelopsis delavayana var. setulosa*
Vitaceae	*Ampelopsis*	*Ampelopsis humulifolia*
Vitaceae	*Ampelopsis*	*Ampelopsis megalophylla*
Vitaceae	*Cayratia*	*Cayratia japonica var. pseudotrifolia*
Vitaceae	*Cayratia*	*Cayratia oligocarpa*
Vitaceae	*Parthenocissus*	*Parthenocissus dalzielii*
Vitaceae	*Parthenocissus*	*Parthenocissus henryana*

Vitaceae	*Parthenocissus*	*Parthenocissus laetevirens*
Vitaceae	*Vitis*	*Vitis amurensis*
Vitaceae	*Vitis*	*Vitis bashanica*
Vitaceae	*Vitis*	*Vitis betulifolia*
Vitaceae	*Vitis*	*Vitis davidii*
Vitaceae	*Vitis*	*Vitis heyneana subsp. ficifolia*
Vitaceae	*Vitis*	*Vitis lanceolatifoliosa*
Vitaceae	*Vitis*	*Vitis piasezkii*
Vitaceae	*Vitis*	*Vitis romanetii*
Vitaceae	*Vitis*	*Vitis shenxiensis*
Vitaceae	*Vitis*	*Vitis silvestrii*
Vitaceae	*Vitis*	*Vitis wilsoniae*
Woodsiaceae	*Woodsia*	*Woodsia cycloloba*
Woodsiaceae	*Woodsia*	*Woodsia rosthorniana*
Woodsiaceae	*Woodsia*	*Woodsia shensiensis*
